# Mitochondrial protein OPA3 sustains cardiac function by regulating calcium handling in male mice

**DOI:** 10.1038/s41467-026-73991-4

**Published:** 2026-06-17

**Authors:** Na Geng, Taiwei Chen, Hao Li, Shan Lu, Yuehong Wang, Ling Gao, Donald M. Bers, Xiyuan Lu

**Affiliations:** 1 Department of Cardiology, Renji Hospital, School of Medicine, State Key Laboratory for Systems Medicine for Cancer, Shanghai Cancer Institute, Shanghai Jiao Tong University, Shanghai, China; 2 Institute for Regenerative Medicine, State Key Laboratory of Cardiology and Medical Innovation Center, Shanghai East Hospital, School of Medicine, Tongji University, Shanghai, China; 3 Nanjing Medical University, School of Pharmacy, Nanjing, China; 4 Department of Pharmacology, University of California-Davis, Davis, CA USA

**Keywords:** Cardiomyopathies, Mechanisms of disease, Mitochondria

## Abstract

Heart failure (HF) is a growing global health burden characterized by impaired cardiac contractility and progressive remodeling, driven in part by disrupted Ca^2+^ handling and mitochondrial dysfunction. However, the molecular mechanisms coordinating these processes remain incompletely understood. Here we showed that OPA3 was decreased in both human and murine HF. Cardiomyocyte-specific deletion of *Opa3* in male mice led to the progressive dilated cardiomyopathy (DCM), accompanied by impaired myocardial function, calcium cycling and mitochondria function. Mechanistically, OPA3 forms multimers that are required for its interaction with phospholamban (PLN), thereby maintaining sarcoplasmic reticulum (SR) Ca^2+^-ATPase (SERCA2a) activity and Ca^2+^ handling. OPA3 is localized to the mitochondrial outer membrane, and its absence impaired mitochondrial function. Cardiomyocyte-specific overexpression of *Opa3* improved cardiac dysfunction in both pressure overload- and doxorubicin-induced HF models. Our data define a critical role of OPA3-PLN-SERCA2a axis that regulates both mitochondria and SR function, representing a potential therapeutic target for HF.

## Abstract (web summary)

Heart failure is a major global disease, but the role of the mitochondrial protein OPA3 in cardiac dysfunction has remained unclear. Here, the authors show that OPA3 preserves heart function by regulating calcium handling via a PLN–SERCA2a pathway, and its loss leads to cardiomyopathy.

## Introduction

Heart failure (HF) is a major and growing public health challenge worldwide^1,2^. Despite substantial advances in pharmacological therapy and device-based interventions, including cardiac resynchronization therapy, HF continues to carry a high burden of morbidity and mortality^1^. The persistent residual risk underscores the need for deeper mechanistic insight and innovative therapeutic approaches. In particular, the ways in which cardiomyocyte organelle homeostasis and intracellular signaling networks progressively deteriorate during HF remain incompletely defined.

One prominent characteristic of HF is abnormal Ca^2+^ handling, which limits cardiac muscle contraction and relaxation and is linked to cardiac arrhythmias and adverse remodeling^3^. A critical contributing factor is decreased function of the sarcoplasmic reticulum Ca^2+^-ATPase (SERCA2a), due in part to stronger inhibition by unphosphorylated phospholamban (PLN), which reduces SERCA2a Ca^2+^ affinity^4^. Elevated diastolic cytosolic Ca^2+^ ([Ca]_i_) caused by SERCA2a deficiency could induce mitochondrial Ca^2+^ overload, mitochondrial dysfunction and impaired ATP synthesis, further exacerbating the progression HF^5^. Notably, communication between mitochondria and the endoplasmic/sarcoplasmic reticulum (ER/SR) can be important in modulating intracellular signal transduction, including Ca^2+^ handling, and energy production^6^. Studies have shown that abnormalities in the interactions between mitochondria and the ER/SR are associated with the occurrence of cardiovascular diseases such as ischemia-reperfusion injury and diabetic cardiomyopathy^7,8^. However, the molecular mechanisms underlying the coupling between SR and the mitochondria during the progression of HF remain unclear.

Optic atrophy (OPA) related gene family is grouped based on a shared clinical phenotype rather than molecular homology^9^. These genes have been identified as pivotal regulators in dominant optic atrophy, characterized by neuromuscular disorders^9^. To date, the recognized genes implicated in this condition include OPA1, OPA3, OPA4, OPA5, and OPA8, with OPA1 being by far the most extensively studied^9^. Research shows that OPA1 plays crucial roles in mitochondrial dynamics, mitochondrial membrane potential, calcium clearance, and oxidative phosphorylation^10^^,^^11^, thereby driving the development of diseases such as dilated cardiomyopathy (DCM)^12^^,^^13^, diabetic cardiomyopathy^14^^,^^15^, and ischemia-reperfusion injury^16^. The *Opa3* gene, initially identified in patients with Costeff optic-atrophy syndrome^17^, has been highlighted in recent research for its contributions to the regulation of mitochondrial function^18–20^ and its relevance in DCM^21–23^. However, the precise function of OPA3 in heart and the setting of HF remains unclear.

In this study, we aimed to elucidate the role of cardiac OPA3 in the pathogenesis of HF. Our findings demonstrated that cardiomyocyte-specific *Opa3* deficiency leads to the spontaneous development of DCM. Mechanistically, we discovered that OPA3 interacts with PLN to regulate SERCA2a, Ca^2+^ release and communication between the mitochondria and the SR, thereby sustaining mitochondrial function and Ca^2+^ homeostasis to preserve cardiac function. Overexpression of *Opa3* mitigated transverse aortic constriction (TAC)- or doxorubicin-induced cardiac dysfunction. Thus, our findings suggest that targeting OPA3 may represent a potential therapeutic strategy for the management of HF.

## Results

### Cardiomyocyte-derived OPA3 is downregulated in cardiomyopathy and HF

**Fig. 1:**
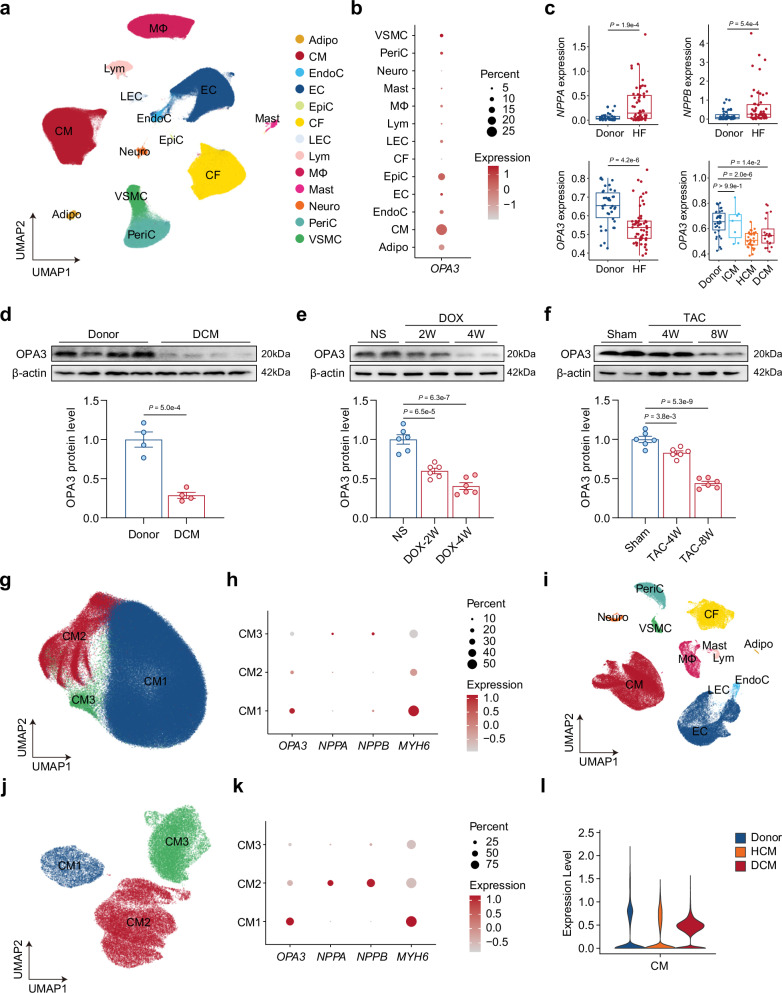
Cardiomyocyte-derived OPA3 is downregulated in cardiomyopathy and HF. **a** UMAP plot of cardiac nuclei from healthy donors and patients with cardiomyopathy including DCM, HCM, and ICM from the Broad Institute’s Single Cell Portal (SCP1303 and SCP1849). Adipo, adipocyte; CM, cardiomyocyte; EndoC, endocardial cell; EC, endothelial cell; EpiC, epicardial cell; CF, cardiac fibroblast; LEC, lymphatic endothelial cell; Lym, lymphocyte; MΦ, macrophage; Mast, mast cell; Neuro, neuron; PeriC, pericyte; VSMC, vascular smooth muscle cell. **b** Dot plot showing *OPA3* gene expression in cells (**a**). **c** Boxplot showing the CP10K of genes in cardiomyocytes (*n* = 38 for Donor, *n* = 57 for HF, *n* = 7 for ICM, *n* = 30 for HCM, *n* = 20 for DCM). Box plots show the median (center line), the 25th and 75th percentiles (box limits), and whiskers extending to the minima and maxima within 1.5 × the interquartile range (IQR). **d** OPA3 expressions in heart tissues from DCM patients and healthy donors (*n* = 4 per group). **e** OPA3 expressions in doxorubicin (DOX)-induced failing hearts and normal saline (NS) controls (*n* = 6 per group). **f** OPA3 expressions in TAC-induced failing hearts and Sham controls (*n* = 6 per group). **g** UMAP plot of three cardiomyocytes subclusters (SCP1303 and SCP1849). **h** Dot plot of gene expressions in three cardiomyocytes subclusters (SCP1303 and SCP1849). **i** UMAP plot of cardiac nuclei from healthy donors and patients with DCM and HCM from SCP1852 database. **j** UMAP plot of three cardiomyocytes subclusters (SCP1852). **k** Dot plot of gene expression in cardiomyocytes subclusters (SCP1852). **l** Violin plot showing *OPA3* gene expression in cardiomyocytes from indicated diseases (SCP1852). Data are expressed as mean ± SEM. Data in **c** were analyzed by the two-sided Mann-Whitney *U* test or Kruskal-Wallis tests followed by Dunn’s post hoc test, **d** by two-sided Student’s *t* test, **e**, **f** by one-way ANOVA followed by the Tukey post hoc test. DCM indicates dilated cardiomyopathy, HCM hypertrophic cardiomyopathy, HF heart failure, ICM ischemic cardiomyopathy, MYH6 myosin heavy chain 6, NPPA natriuretic peptide type A, NPPB natriuretic peptide type B, OPA3 optic atrophy 3, TAC transverse aortic constriction, UMAP uniform manifold approximation and projection.

To investigate whether OPA3 was involved in HF, we analyzed a large single-nucleus RNA sequencing (snRNA-seq) dataset containing 692,373 nuclei derived from two combined public databases^24,25^. This dataset included heart samples from patients with dilated cardiomyopathy (DCM), hypertrophic cardiomyopathy (HCM), and ischemic cardiomyopathy (ICM), as well as healthy donors. Our analysis revealed that, among all cell types, cardiomyocytes exhibited the highest proportion of *OPA3*-expressing cells (Fig. 1a, b and Supplementary Fig. 1a). Moreover, the *OPA3* expression in cardiomyocytes was significantly downregulated in HF, accompanied by the upregulation of *NPPA* and *NPPB* (Fig. 1c). This reduction in *OPA3* expression was observed in HCM and DCM, while no significant change was detected in ICM (Fig. 1c). We next examined OPA3 protein expression in the context of HF. OPA3 protein levels were markedly reduced in human HF hearts compared to healthy donor hearts (Fig. 1d and Supplementary Table 3). Similar decreases in OPA3 protein levels were observed in mouse models of HF, including both a doxorubicin-induced cardiomyopathy model (Fig. 1e) and a transverse aortic constriction (TAC)-induced HF model (Fig. 1f).

To further explore the relationship between cardiomyocyte-derived OPA3 expression and HF, we extracted cardiomyocyte data from the snRNA-seq dataset and divided them into three cardiomyocyte (CM) clusters: CM1, CM2, and CM3 (Fig. 1g). Among these, CM3 exhibited an HF phenotype, characterized by elevated *NPPA* and *NPPB* expression and reduced *MYH6* expression (Fig. 1h). Notably, CM3 cardiomyocytes also exhibited significantly lower *OPA3* expression compared to CM1 and CM2 (Fig. 1h). These findings were validated using another publicly available snRNA-seq dataset of congenital heart disease^26^, which demonstrated a strong correlation between cardiomyocyte-derived *OPA3* expression and HF (Fig. 1i–l and Supplementary Fig. 1b).

Since the mRNA level of *Opa3* was reduced in the failing hearts, we then investigated the potential transcriptional factors that may regulate *Opa3* expression using bioinformatics tools, including hTFtarget, Cistrome, JASPAR, and ENCODE. The intersection of multiple datasets identified two transcriptional factors, cAMP response element-binding protein (CREB) and RELA, as the most promising transcription factors in the regulation of *Opa3* (Supplementary Fig. 2a and Supplementary Table 4). Notably, *Creb* but not *Rela* was downregulated in heart tissues from HF compared to controls, as evidenced by a public RNA-seq database (Supplementary Fig. 2b). In addition, CREB has been implicated in the pathogenesis of DCM^27^. Thus, we aimed to evaluate whether CREB was involved in the transcriptional regulation of *Opa3* gene. Through JASPAR website (https://jaspar.genereg.net/)^28^, we found that the binding peak contained an evolutionarily conserved cAMP response element (CRE) site (Supplementary Fig. 2c). Subsequent chromatin immunoprecipitation assay confirmed the direct binding of CREB to the *Opa3* promoter region (−287 to −294) which was reduced in TAC vs. sham mouse hearts (Supplementary Fig. 2c−e). Functional analysis using a dual luciferase reporter gene assay demonstrated that the activity of *Opa3* luciferase reporter was enhanced by CREB co-expression, and this effect was abolished by the mutation of CREB binding site in the *Opa3* promoter (Supplementary Fig. 2f). Taken together, our results show that OPA3 is suppressed in HF, which is attributed to the reduced transcriptional activity of CREB.

### Cardiomyocyte-specific deficiency of Opa3 leads to progressive DCM and HF

**Fig. 2:**
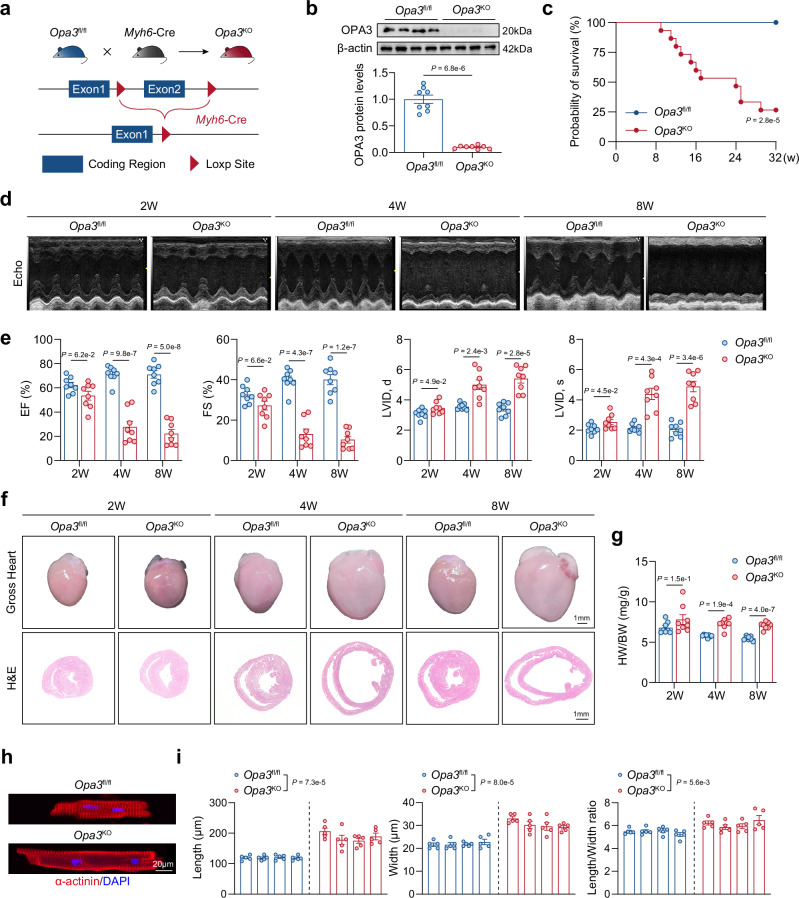
Cardiomyocyte-specific deficiency of *Opa3* leads to progressive DCM and HF. **a** Schematic diagram illustrating the strategy of cardiomyocyte-specific *Opa3* knockout mice construction. **b** Top, Representative western blots for OPA3 and β-actin in heart tissues from *Opa3*^fl/fl^ and *Opa3*^KO^ mice. Bottom, Quantification of protein expressions in indicated groups shown in **Top** (*n* = 8 per group). **c** Survival curves in indicated groups (*n* = 15 per group). Representative M-mode echocardiographic images (**d**) and corresponding measurements of EF, FS, LVID,d, and LVID,s (**e**) of *Opa3*^fl/fl^ and *Opa3*^KO^ mice at 2 weeks, 4 weeks, and 8 weeks of age (*n* = 8 per group). **f** Top, Representative gross images of hearts from *Opa3*^fl/fl^ and *Opa3*^KO^ mice at indicated time points (scale bar, 1 mm). Bottom, Representative images of H&E-stained heart sections in indicated groups (scale bar, 1 mm). **g** Ratios of heart weight to body weight (HW/BW) in indicated groups (*n* = 8 per group). **h** Representative immunofluorescence images of α-actinin in isolated AMCMs from *Opa3*^fl/fl^ and *Opa3*^KO^ mice at 8 weeks of age (scale bar, 20 μm). **i** Quantifications of cell length, width, and length/width ratio shown in (**h**) (5 cardiomyocytes per animal, 4 animals per group). Data are expressed as mean ± SEM. Data in **b**, **e**, **g** were analyzed by two-sided Student’s *t* test (equal variances) or Welch’s *t* test (unequal variances). Survival data in **c** were analyzed by the Kaplan-Meier method and compared using log-rank test. Data in **i** were analyzed by two-sided nested *t* test. AMCM indicates adult mouse cardiomyocyte, DCM dilated cardiomyopathy, EF ejection fraction, FS fractional shortening, H&E hematoxylin and eosin, HF heart failure, LVID,d, internal dimension of the left ventricle in diastole, LVID,s internal dimension of the left ventricle in systole, Opa3 optic atrophy 3.

To interrogate the role of OPA3 in cardiac pathology, we crossed *Opa3*^fl/fl^ mice with those carrying cardiomyocyte-specific Cre (*Myh6*-Cre) to generate cardiomyocyte-specific *Opa3* knockout mice (*Opa3*^KO^) (Fig. 2a and Supplementary Fig. 3). We observed a pronounced reduction of OPA3 protein levels in the hearts of *Opa3*^KO^ mice, indicating the successful deletion of the *Opa3* gene in cardiomyocytes (Fig. 2b). Notably, the deficiency of *Opa3* in cardiomyocytes resulted in a remarkable reduction in lifespan, with an increased mortality around 9–32 weeks (Fig. 2c). To determine whether the cause of death was attributed to HF, both *Opa3*^fl/fl^ and *Opa3*^KO^ mice were subjected to serial echocardiogram analyses to assess cardiac function at indicated times before death (2–8 weeks). Echocardiography revealed a progressive decline of systolic function with age in *Opa3*^KO^ mice compared to *Opa3*^fl/fl^ mice, as evidenced by reduced left ventricular ejection fraction (EF) and fractional shortening (FS), which was accompanied by increased left ventricular systolic and end-diastolic diameters, and decreased wall thickness, indicating DCM (Fig. 2d, e and Supplementary Table 5). In addition, consistent heart enlargement and increased heart weight to body weight ratios (HW/BW) were observed in *Opa3*^KO^ mice (Fig. 2f, g). Wheat germ agglutinin (WGA) staining showed a gradual increase in the cross-sectional area of cardiomyocytes in *Opa3*^KO^ mice (Supplementary Fig. 4a, b). Morphological analyses of isolated adult mouse cardiomyocytes (AMCMs) demonstrated significant eccentric hypertrophic remodeling, characterized by increased cell length, cell width, and an elevated length/width ratio in *Opa3*^KO^ mice compared to littermate controls (Fig. 2h, i). Progressive fibrosis was also observed in *Opa3*^KO^ mice as evidenced by Masson’s trichrome staining and Sirius Red staining (Supplementary Fig. 4a, c, d). In addition, the levels of biomarkers associated with HF such as ANP, BNP, and MYH7, as well as fibrosis, including Col1a2 and Col3a1, were gradually elevated in *Opa3*^KO^ mice compared to their littermate controls (Supplementary Fig. 4e, f). Collectively, our data provide convincing evidence that deficiency of *Opa3* in cardiomyocytes leads to distinct functional and histological characteristics of DCM, suggesting the pivotal role of OPA3 in HF pathogenesis.

### Adult cardiomyocyte-specific deficiency of *Opa3* Leads to DCM and exacerbates TAC-induced HF

To further determine the role of OPA3 in adult hearts, we generated inducible cardiomyocyte-specific *Opa3* knockout mice (*Opa3*^iKO^) by crossing *Opa3*^fl/fl^ mice with those carrying cardiomyocyte-specific MerCreMer (*Myh6*-MCM) (Supplementary Fig. 5a, b). Immunoblotting of heart tissue lysates confirmed efficient ablation of OPA3 protein in *Opa3*^iKO^ mice after tamoxifen administration (Supplementary Fig. 5c). Echocardiography revealed a significant systolic dysfunction in *Opa3*^iKO^ mice compared to *Opa3*^fl/fl^ and *Myh6*-MCM control mice, as evidenced by reduced EF and FS, increased left ventricular systolic and end-diastolic diameters, and decreased wall thickness (Supplementary Fig. 6a, b and Supplementary Table 6). In addition, *Opa3*^iKO^ mice exhibited significant heart enlargement, and the HW/BW ratios were higher in these mice than their littermate controls (Supplementary Fig. 6c, d). The pathological analyses, including WGA staining, Masson’s trichrome staining, Sirius Red staining, and morphological analyses, further confirmed that the absence of *Opa3* in adult cardiomyocytes resulted in significant dilation and fibrosis of the myocardium (Supplementary Fig. 6e–j). Moreover, levels of biomarkers associated with HF and fibrosis were elevated in *Opa3*^iKO^ mice compared to *Opa3*^fl/fl^ and *Myh6*-MCM control mice (Supplementary Fig. 6k, l). Notably, these functional and pathological changes were further exacerbated 8 weeks after TAC surgery (Supplementary Fig. 7a–j and Supplementary Table 7). Collectively, these results suggest that the absence of *Opa3* in adult cardiomyocytes recapitulates the progression of HF, which was exacerbated under the pathological condition of pressure overload.

### Cardiomyocyte-specific overexpression of *Opa3* ameliorates doxorubicin-induced cardiomyopathy

To investigate whether cardiomyocyte-specific overexpression of *Opa3* could mitigate cardiac dysfunction in a doxorubicin-induced cardiomyopathy model, we generated adeno-associated virus serotype 9 (AAV9) carrying a *cTnT* promoter to overexpress wild-type *Opa3* (AAV-*Opa3*) specifically in cardiomyocytes. AAV-*Opa3* and AAV-Vector were delivered into *Opa3*^fl/fl^ mice 2 weeks before doxorubicin administration to induce gene overexpression (Supplementary Fig. 8a–c). Under baseline conditions with normal saline administration, *Opa3* overexpression did not result in any significant changes in cardiac function or histology compared to vector controls (Supplementary Fig. 8d–j). However, the overexpression of *Opa3* effectively attenuated the doxorubicin-induced reductions in EF and FS, as well as the left ventricular dilation (Supplementary Fig. 8d, e and Supplementary Table 8). In addition, AAV-*Opa3* ameliorated cardiac enlargement and fibrosis induced by doxorubicin (Supplementary Fig. 8f–j). Overexpression of *Opa3* also resulted in a notable decrease in biomarkers associated with HF and fibrosis (Supplementary Fig. 8k, l). These findings demonstrate that cardiomyocyte-specific overexpression of *Opa3* markedly improves cardiac function and alleviates pathological remodeling in the context of doxorubicin-induced cardiomyopathy, highlighting its therapeutic potential.

### Cardiomyocyte-derived *Opa3* regulates pathways related to cardiac dysfunction

**Fig. 3:**
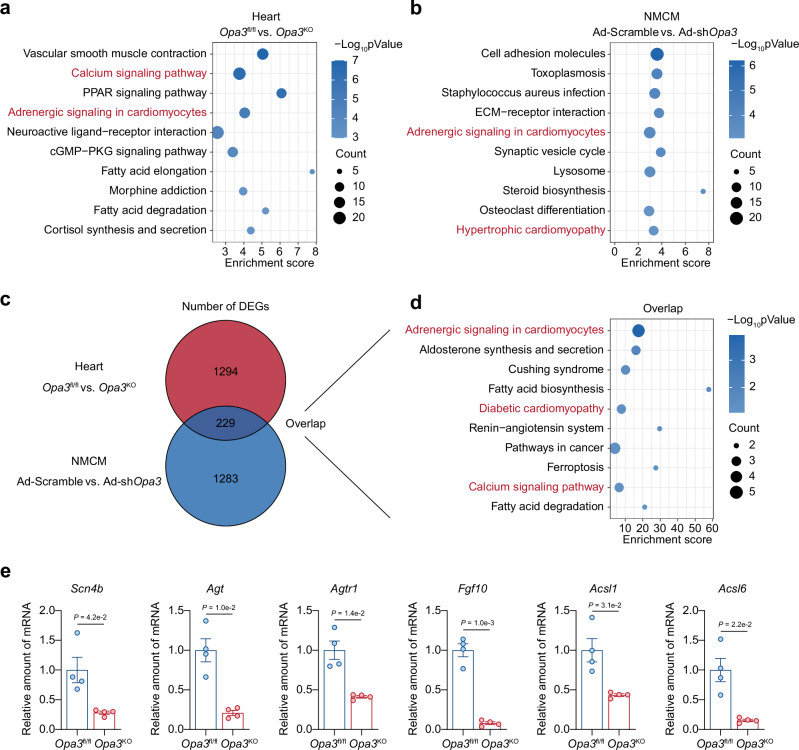
Cardiomyocyte-derived *Opa3* regulates pathways related to cardiac dysfunction. **a** The top 10 enriched KEGG pathways of downregulated DEGs in heart tissues from *Opa3*^KO^ mice compared to *Opa3*^fl/fl^ controls. **b** The top 10 enriched KEGG pathways of downregulated DEGs in NMCMs infected with adenovirus encoding scramble (Ad-Scramble) or shRNA targeting *Opa3* (Ad-sh*Opa3*) for 48 h. **c** Venn diagram showing the number of overlap DEGs shared by both heart and NMCM. **d** The top 10 enriched KEGG pathways of downregulated overlap DEGs shared by both heart and NMCM. **e** Relative mRNA expressions of genes related to cardiac function in heart tissues from *Opa3*^fl/fl^ vs. *Opa3*^KO^ mice (*n* = 4 per group). Data are expressed as mean ± SEM. Data in **a**, **b**, **d** were analyzed by one-sided hypergeometric test. Data in **e** were analyzed by two-sided Welch’s *t* test. DCM indicates dilated cardiomyopathy, DEG differentially expressed genes, KEGG Kyoto Encyclopedia Genes and Genomes, NMCM neonatal mouse cardiomyocyte, Opa3 optic atrophy 3.

To explore mechanisms involved in cardiac dysfunction after *Opa3* deficiency, we conducted comprehensive RNA-sequencing (RNA-seq) to assess early transcriptomic alterations in heart tissues of *Opa3*^KO^ and *Opa3*^fl/fl^ mice (4 weeks). We identified a total of 1523 differentially expressed genes (DEGs), with 975 being upregulated and 548 downregulated in *Opa3*^KO^ hearts (Supplementary Fig. 9a). Bioinformatic analysis showed that the upregulated DEGs were enriched in fibrosis-related pathways (including ECM-receptor interaction, focal adhesion, and TGF-beta signaling pathway) (Supplementary Fig. 9c), and downregulated DEGs were enriched in pathways related to cardiac function (calcium signaling pathway and adrenergic signaling in cardiomyocytes) (Fig. 3a). We also performed RNA-seq analysis on NMCMs infected with adenovirus encoding scramble (Ad-Scramble) or shRNA targeting *Opa3* (Ad-sh*Opa3*). A total of 1512 DEGs were identified, with 972 upregulated and 540 downregulated in Ad-sh*Opa3* (Supplementary Fig. 9b). Notably, the upregulated DEGs were enriched in inflammatory pathways (including TNF signaling pathway, cytokine-cytokine receptor interaction, and viral protein interaction with cytokine and cytokine receptor) (Supplementary Fig. 9d), while downregulated DEGs were enriched in pathways related to cardiac function (adrenergic signaling in cardiomyocytes and hypertrophic cardiomyopathy) (Fig. 3b). In addition, a comparison between the two datasets identified 229 overlapping DEGs in both heart tissues and NMCMs (Fig. 3c), which were significantly enriched in pathways that related to inflammation and cardiac function (Fig. 3d and Supplementary Fig. 9e). These findings were further confirmed by RT-qPCR (Fig. 3e and Supplementary Fig. 9f). Taken together, our results suggest that OPA3 plays a critical role in maintaining cardiac function by sustaining cellular function in cardiomyocytes.

### The absence of *Opa3* in cardiomyocytes Leads to Ca^2+^ handling deficiency

**Fig. 4:**
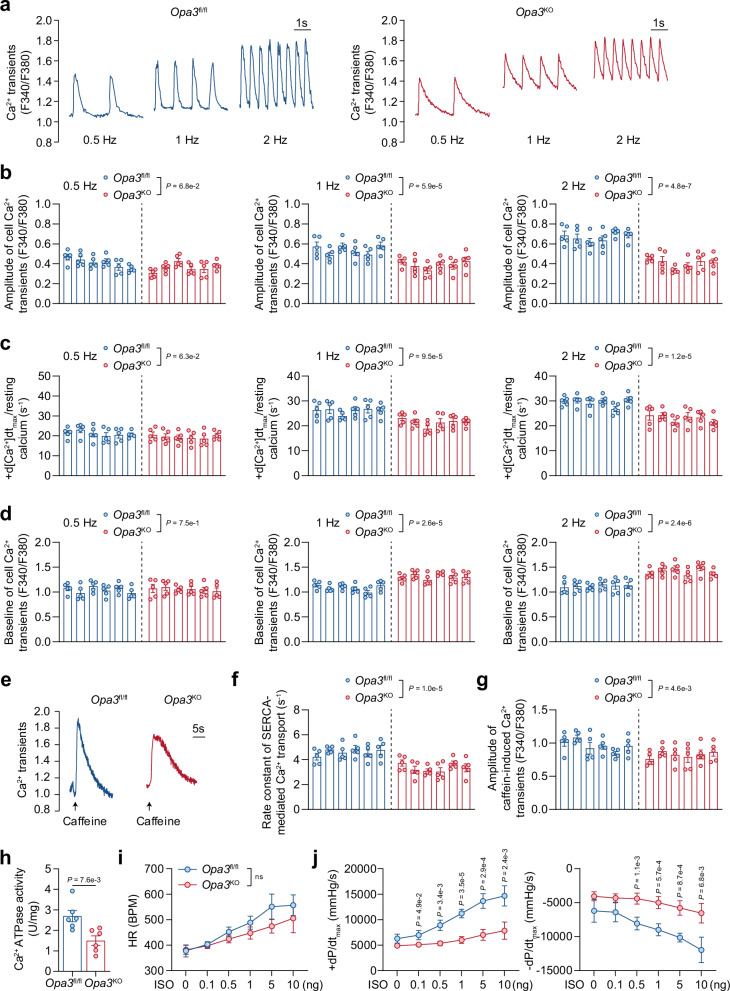
The absence of *Opa3* in cardiomyocytes leads to Ca^2+^ handling deficiency. **a**–**d** AMCMs were incubated with a Fura-2 AM and Ca^2+^ transients were recorded under continuous 0.5 Hz, 1 Hz, and 2 Hz electrical stimulation. **a** Representative traces of calcium transients in isolated AMCMs from *Opa3*^fl/fl^ and *Opa3*^KO^ mice at 8 weeks of age. Quantification of amplitude of Ca^2+^ transients (**b**), maximal ascending rate in cell contractile Ca^2+^ transients (**c**) and baseline of Ca^2+^ transients (**d**) shown in (**a**) (5 cardiomyocytes per animal, 6 animals per group). **e** Representative traces of Ca^2+^ transients during caffeine (10 mM)-induced contraction in NT solution in isolated AMCMs from *Opa3*^fl/fl^ and *Opa3*^KO^ mice at 8 weeks of age. Quantification of the rate constant of SERCA-mediated Ca^2+^ transport (**f**) and the amplitude of caffeine-induced Ca^2+^ transients (SR Ca^2+^ content, **g**) shown in (**e**) (5 cardiomyocytes per animal, 6 animals per group). **h** The Ca^2+^-ATPase activities in hearts from *Opa3*^fl/fl^ and *Opa3*^KO^ mice were assayed using the Ca^2+^-ATPase assay kit (*n* = 6 per group). The effects of increasing doses of isoproterenol (0.1 to 1.0 ng/mouse) on heart rate (**i**), ±dP/dt (**j**) in *Opa3*^fl/fl^ and *Opa3*^KO^ mice at 8 weeks of age (*n* = 5 per group). Data are expressed as mean ± SEM. Data in **b**–**d**, **f**, **g** were analyzed by two-sided nested *t* test. Data in **h** were analyzed by two-sided Student’s *t* test. Data in **i**, **j** were analyzed by a two-way repeated measures ANOVA followed by the Bonferroni multiple comparisons. AMCM indicates adult mouse cardiomyocyte, NT normal Na^+^/Ca^2+^ Tyrode’s, Opa3 optic atrophy 3, SERCA sarcoplasmic/endoplasmic reticulum Ca^2+^-ATPase, SR sarcoplasmic reticulum.

Based on the transcriptomic analysis showing a robust association between OPA3 and calcium signaling pathway, we sought to determine whether *Opa3* knockout in cardiomyocytes led to a deficiency of Ca^2+^ handling. To this end, we analyzed cell shortening and Ca^2+^ transients in isolated AMCMs from *Opa3*^fl/fl^ and *Opa3*^KO^ mice at 0.5 Hz, 1 Hz, and 2 Hz electrical stimulation. The amplitude of cell shortening and the maximum upstroke velocity of cell shortening were significantly reduced in AMCMs from *Opa3*^KO^ mice at 1 Hz and 2 Hz electrical stimulation compared to that from *Opa3*^fl/fl^ mice, indicating compromised cardiomyocyte contractility following *Opa3* deletion (Supplementary Fig. 10a–c). In parallel, the amplitude of Ca^2+^ transients and maximal rate of rise of Ca^2+^ transients were also diminished in AMCMs from *Opa3*^KO^ mice (Fig. 4a–c). Notably, the intracellular levels of diastolic Ca^2+^ were significantly elevated in the absence of *Opa3* (Fig. 4d). To evaluate SR Ca^2+^ content and function of Ca^2+^ transport by the SR/ER calcium ATPase (SERCA), we analyzed the amplitude and kinetics of Ca^2+^ release induced by caffeine. The rate constant of SERCA-mediated [Ca]_i_ decline was markedly decreased in AMCMs from *Opa3*^KO^ mice, suggesting a reduced Ca^2+^ reuptake of SR after *Opa3* ablation (Fig. 4e, f) and a reduction of SR Ca^2+^ content in AMCMs from *Opa3*^KO^ mice compared to *Opa3*^fl/fl^ counterparts (Fig. 4e, g). Furthermore, a decrease in Ca^2+^-ATPase activity was also observed after *Opa3* ablation (Fig. 4h).

To further elucidate the physiological consequences of *Opa3* deficiency, we performed dose-response experiments using the β-adrenergic agonist isoproterenol to assess its positive inotropic and lusitropic effects in vivo. Hemodynamic measurements showed that *Opa3* deficiency significantly impaired the positive inotropic ( + dP/dt) and lusitropic (–dP/dt) responses to β-adrenergic stimulation (Fig. 4i, j).

To further investigate the direct impact of *Opa3* deficiency on calcium handling, we isolated AMCMs from *Opa3*^fl/fl^ mice and infected them with adenovirus carrying scramble or shRNA targeting *Opa3* for 48 h. Like the findings in *Opa3*^KO^ mice, acute *Opa3* knockdown resulted in similar calcium handling defects in AMCMs of reduced contraction and Ca^2+^ transient amplitude, SR Ca^2+^ load and rate of SERCA2 transport (Supplementary Figs. 11 and 12). The absence of *Opa3* also reduced SR Ca^2+^ leak assessed by spontaneous ryanodine receptor (RyR) Ca^2+^ sparks (Supplementary Fig. 13), but that is likely due to the reduction in SR Ca^2+^ content, which by itself decreases Ca^2+^ spark frequency^29^. We further investigated the effect of OPA3 deficiency on mitochondrial calcium. Our data indicated that *Opa3* knockdown does not affect mitochondrial calcium uptake in Ca^2+^ buffered permeabilized cells, as demonstrated by the mitochondrial calcium sensor Mitycam (Supplementary Fig. 14a). This suggests that OPA3 does not directly influence mitochondrial calcium uniporter (MCU) activity. However, resting mitochondrial Ca^2+^ levels were elevated in *Opa3* knockdown cardiomyocytes (Supplementary Fig. 14b). In summary, these findings underscore the critical role of OPA3 in regulating Ca^2+^ handling in cardiomyocytes and highlight its importance in maintaining cardiomyocyte contractility and response to physiological stress.

### OPA3 interacts with SR-located protein phospholamban (PLN)

**Fig. 5:**
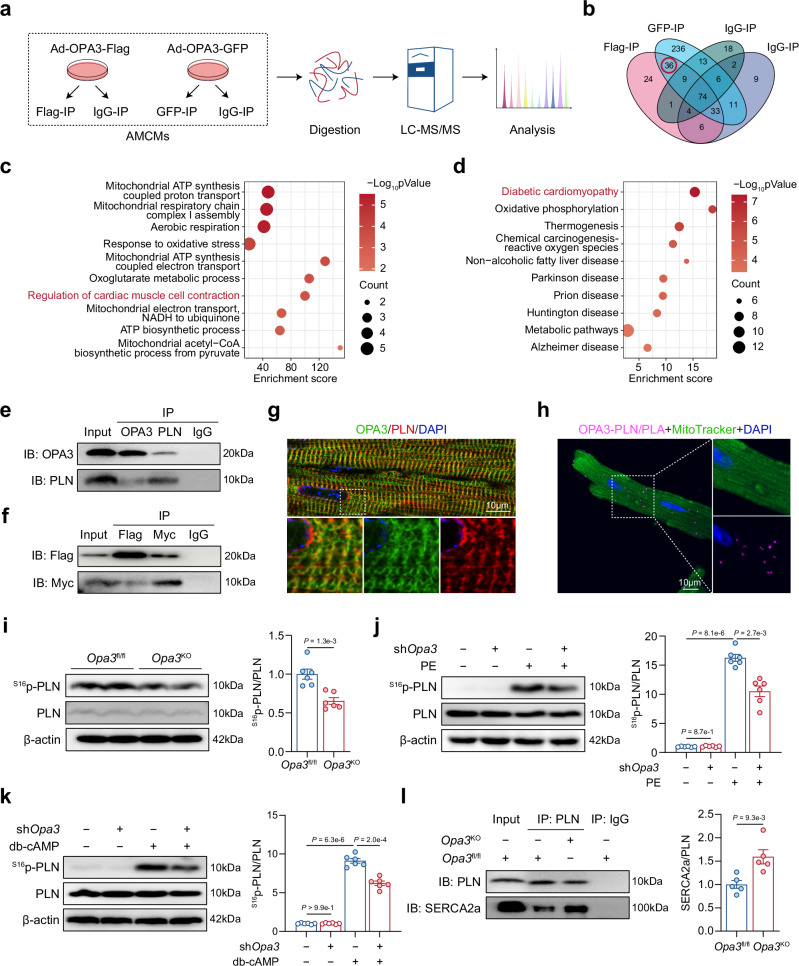
OPA3 interacts with SR-located protein phospholamban (PLN). **a** Schematic diagram depicting the experimental protocol for mass spectrometry. **b** The overlap number of proteins among Flag-IP, GFP-IP, and IgG-IP groups is shown by Venn diagram. The red circle indicates 36 proteins exclusively identified within the overlap of FLAG-IP and GFP-IP groups. The top 10 enriched biological processes from GO analysis (**c**) and the top 10 enriched KEGG pathways (**d**) of 36 overlap proteins shown in (**b**). **e** Coimmunoprecipitation of OPA3 and PLN in heart tissues. Endogenous OPA3 or PLN was immunoprecipitated by anti-OPA3 or anti-PLN antibody, respectively, using IgG as a negative control. **f** Coimmunoprecipitation of OPA3 and PLN in HEK293T cells co-transfected with OPA3-Flag and PLN-GFP plasmids. **g** Colocalization of OPA3 (green) and PLN (red) in heart sections (scale bars, 10 μm). Nuclei were counterstained with DAPI (blue). **h** The interaction between OPA3 and PLN was assessed by proximity ligation assay (PLA). Mitochondria were labeled with MitoTracker, and nuclei were counterstained with DAPI (scale bars, 10 μm). **i** PLN and its phosphorylation levels in PE-treated (50 nM for 48 h) AMCMs isolated from *Opa3*^fl/fl^ and *Opa3*^KO^ mice (*n* = 6 per group). PLN and its phosphorylation levels in PE-treated (50 nM, **j**) or db-cAMP-treated (10 μM, **k**) NMCMs that were infected with adenovirus encoding scramble (Scramble) or shRNA targeting *Opa3* (sh*Opa3*) for 48 h (*n* = 6 per group). **l** Interaction analysis between PLN and SERCA2a in heart tissues (*n* = 5 per group). Data are expressed as mean ± SEM. Data in **c**, **d** were analyzed by one-sided hypergeometric test. Data in **i**, **l** were analyzed by two-sided Student’s *t* test. Data in **j**, **k** were analyzed by Welch’s ANOVA test followed by a post-hoc analysis using the Tamhane T2 method. All the experiments in **e**–**h** were replicated independently at least three times. AMCM indicates adult mouse cardiomyocyte, GO Gene Ontology, IgG immunoglobulin G, IP immunoprecipitation, KEGG Kyoto Encyclopedia Genes and Genomes, NMCM neonatal mouse cardiomyocyte, OPA3 optic atrophy 3, PE phenylephrine, PLN phospholamban, SERCA2a sarcoplasmic/endoplasmic reticulum Ca^2+^-ATPase 2a, SR sarcoplasmic reticulum.

To dissect the molecular mechanisms underlying OPA3 function, we screened potential OPA3-interacting proteins using co-immunoprecipitation (co-IP) combined with mass spectrometry analysis. Specifically, isolated AMCMs were infected with adenoviruses encoding Flag-tagged or GFP-tagged OPA3, followed by immunoprecipitation using anti-Flag (Flag-IP) or anti-GFP (GFP-IP) antibodies, with IgG (IgG-IP) serving as an isotype control (Fig. 5a). Our analysis identified 36 proteins that uniquely presented in the overlap of the Flag-IP and GFP-IP groups (Fig. 5b and Supplementary Table 9). Further interrogation of these overlapping proteins revealed their significant enrichment in pathways that related to cardiac function (mitochondrial function and cardiac muscle cell contraction) (Fig. 5c) and biological processes that linked to cardiomyopathy (Fig. 5d). Notably, PLN, a SR-located protein implicated in the pathogenesis of DCM^30^, emerged as a potential target of OPA3 among overlapping proteins. To verify the association between OPA3 and PLN, we conducted experiments addressing both endogenous and exogenous protein interactions. As expected, an interaction between endogenous OPA3 and PLN was observed in heart tissues (Fig. 5e). Co-IP assay in HEK293T cells that overexpressed exogenous OPA3-Flag and PLN-Myc substantiated their interaction (Fig. 5f). In addition, further analysis confirmed their colocalization in AMCMs (Fig. 5g). We measured the colocalized OPA3-PLN fluorescence signal and normalized it to the total OPA3 fluorescence intensity within cardiomyocytes. This analysis revealed that approximately 64.89% of total OPA3 is engaged in interaction with PLN in situ, whereas the remaining fraction of OPA3 exhibits putative mitochondrial localization (Supplementary Fig. 15a). Further, proximity ligation assays (PLA) in conjunction with mitochondrial staining demonstrated that the OPA3-PLN interaction occurs at the mitochondrial edge (Fig. 5h), providing stronger evidence for the relevance of this interaction. Given the pivotal role of PLN in modulating SERCA2a activity^31^, we examined the expression levels of phosphorylated PLN after *Opa3* deletion. Our results showed that the absence of *Opa3* led to a significant decline in PLN phosphorylation, indicating stronger functional inhibition of SERCA2a activity (Fig. 5i). Similar results were observed in NMCMs under stimulation with both phenylephrine (PE) and dibutyryl-cAMP (db-cAMP), a cAMP analog that activates protein kinase A (PKA). *Opa3* knockdown resulted in a significant reduction in PE- or db-cAMP-induced PLN phosphorylation (Fig. 5j, k). In parallel, phosphorylation of ryanodine receptor 2 (RyR2) was also decreased following Opa3 deletion (Supplementary Fig. 15b). To exclude the possibility that the diminished PLN phosphorylation is attributed to reduced ATP content, isolated NMCMs were supplemented pyruvate to enhance ATP production (Supplementary Fig. 15c). Our results showed that pyruvate supplementation restored the RyR2 phosphorylation levels (Supplementary Fig. 15d). However, the PLN phosphorylation remained unchanged upon pyruvate supplementation in *Opa3* knockdown NMCMs (Supplementary Fig. 15d). These results suggest that the impairment of PLN phosphorylation is not solely due to a global energy deficit but may involve a more specific mechanism. Given that PLN regulates SR Ca^2+^ uptake by forming a complex with SERCA2a^32^, we therefore explored the relationship among PLN, SERCA2a, and OPA3. Notably, the absence of *Opa3* significantly enhanced the interaction between PLN and SERCA2a, aligning with the observed reduction in PLN phosphorylation (Fig. 5l). However, no direct interaction between OPA3 and SERCA2a was detected (Supplementary Fig. 15e, f). Taken together, we conclude that OPA3 enhances SR Ca^2+^ uptake via an interaction with PLN that limits the ability of PLN to inhibit SERCA2 function.

### OPA3 predominantly localizes to the outer mitochondrial membrane and regulates mitochondria-SR communication

**Fig. 6:**
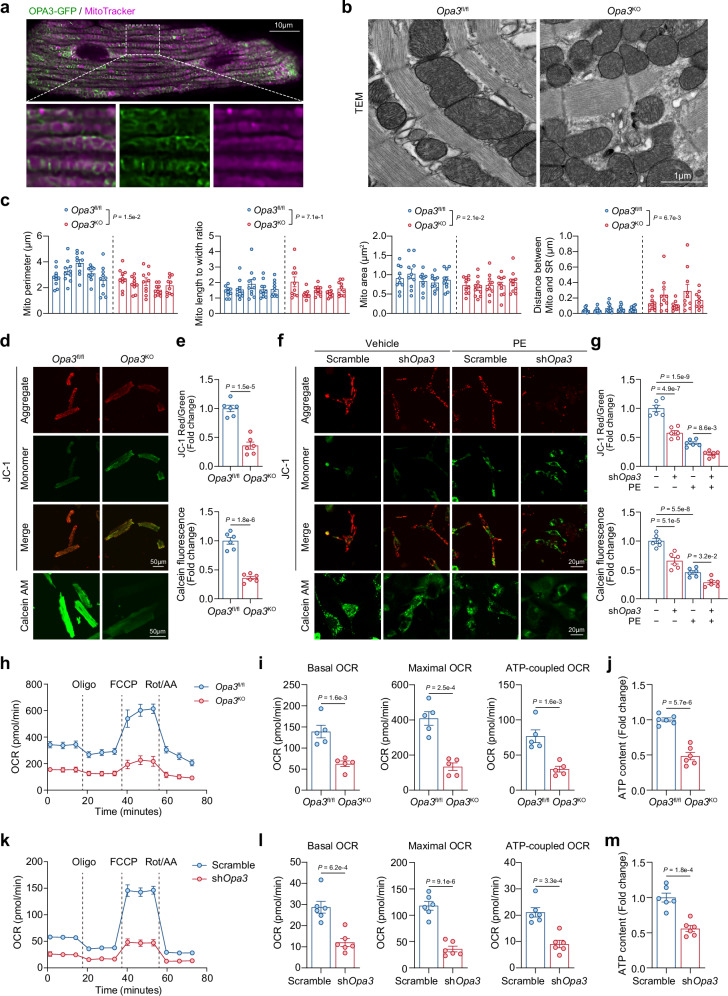
OPA3 predominantly localizes to the outer mitochondrial membrane and regulates mitochondria-SR communication. **a** MitoTraker staining in isolated AMCMs from *Opa3*^fl/fl^ mice after infected with adenovirus encoding OPA3-GFP fusion protein for 48 h (scale bar, 10 μm). The experiment was replicated independently at least three times. **b** Transmission electron micrographs (TEM) of ventricular tissue from *Opa3*^fl/fl^ and *Opa3*^KO^ mice at 8 weeks of age (scale bars, 1 μm). **c** Quantifications of mitochondrial perimeter, mitochondrial length to width ratio, mitochondrial area, and distance between mitochondrial and SR shown in (**b**) (*n* = 10 per animal, 5 animals per group). Representative images (**d**) and quantification (**e**) of mitochondrial ∆Ψm assessed by JC-1 staining and mPTP opening by calcein AM/CoCl_2_ staining in isolated AMCMs from *Opa3*^fl/fl^ and *Opa3*^KO^ mice (*n* = 6 per group, scale bar, 50 μm). Representative images (**f**) and quantification (**g**) of mitochondrial ∆Ψm assessed by JC-1 staining and mPTP opening by calcein AM/CoCl_2_ staining in isolated NMCMs (*n* = 6 per group, scale bar, 20 μm). Analysis (**h**) and quantification (**i**) of mitochondrial OCR in isolated AMCMs from *Opa3*^fl/fl^ and *Opa3*^KO^ mice (*n* = 5 per group). The dash lines indicate the sequential addition of oligomycin (Oligo), FCCP, and Rot/AA. **j** ATP content of isolated AMCMs from *Opa3*^fl/fl^ and *Opa3*^KO^ mice (*n* = 6 per group). Analysis (**k**) and quantification (**l**) of mitochondrial OCR in isolated NMCMs infected with adenovirus encoding scramble (Ad-Scramble) or shRNA targeting *Opa3* (Ad-sh*Opa3*) for 48 h (*n* = 6 per group). **m** ATP content in NMCMs of each group (*n* = 6 per group). Data are expressed as mean ± SEM. Data in **c** were analyzed by two-sided nested *t* test. Data in **e**, **i**, **j**, **l**, **m** were analyzed by two-sided Student’s *t* test. Data in **g** were analyzed by one-way ANOVA followed by the Tukey post hoc test. AA indicates antimycin A, AMCM adult mouse cardiomyocyte, ATP adenosine triphosphate, FCCP carbonyl cyanide 4-(trifluoromethoxy) phenylhydrazone, IgG immunoglobulin G, mPTP mitochondrial permeability transition pore, NMCM neonatal mouse cardiomyocyte, OCR oxygen consumption rate, OPA3 optic atrophy 3, PE phenylephrine, Rot rotenone, SR sarcoplasmic reticulum.

We next identified the subcellular localization of OPA3 in cardiomyocytes. Cryo-immunogold electron microscopy demonstrated that OPA3 was mainly located in mitochondria in adult hearts (Supplementary Fig. 16a). Additionally, subcellular fractionation followed by western blot analysis was performed to further determine the specific localization of OPA3 protein. This approach revealed a pronounced presence of OPA3 mainly in the mitochondrial fraction, with lower presence in the cytosolic fraction and an absence in nuclear extracts (Supplementary Fig. 16b). Confocal microscopy further corroborated these findings, showing predominant mitochondrial localization of OPA3, particularly within the outer membrane in AMCMs (Fig. 6a) and H9C2 cells (Supplementary Fig. 16c, d). In addition, the regions where OPA3 signals do not overlap with PLN may represent putative mitochondrial localization (Fig. 5g). Based on the above findings, we sought to ascertain whether OPA3 was positioned in the mitochondrial outer membrane (MOM), mitochondrial inner membrane (MIM) or the intermembrane space (IMS) of mitochondria by subjecting mitochondrial fractions to proteinase K. Similar to the MOM marker protein mitochondrial translocase of the outer membrane 20 (TOM20), OPA3 exhibited susceptibility to proteinase K digestion, indicating its presence within the MOM (Supplementary Fig. 16e). By contrast, the IMS component mitochondrial intermembrane space import and assembly protein 40 (MIA40) and the MIM protein OPA1 remained unaffected unless we solubilized membranes with detergent (Supplementary Fig. 16e). Considering the distinct localizations of OPA3 in mitochondria and PLN/SERCA2a in the SR, we next assessed the impact of OPA3 on mitochondrial-SR communication. Transmission electron microscopy (TEM) analyses of *Opa3*^KO^ heart tissues demonstrated an increased number of mitochondria and prevalence of smaller mitochondria relative to their littermate controls, whereas no significant alterations in cristae morphology were observed (Fig. 6b, c). Furthermore, an increase in the mean distance between SR and the outer mitochondrial membrane was observed in *Opa3*^KO^ heart tissues (Fig. 6b, c). Moreover, an upregulation of endoplasmic reticulum stress markers, including activating transcription factor 4 (ATF4) and C/EBP homologous protein (CHOP), was detected in heart tissues from *Opa3*^KO^, indicating induction of ER/SR stress (Supplementary Fig. 17).

We then investigated whether the *Opa3* deficiency was involved in mitochondrial dysfunction. First, mitochondrial membrane potential (ΔΨm) was assessed using JC-1, a fluorescent dye sensitive to the ΔΨm variation. The ratio of red/green JC-1 fluorescence was significantly decreased in AMCMs isolated from *Opa3*^KO^ mice compared to that from *Opa3*^fl/fl^ mice, indicating the occurrence of mitochondrial depolarization after *Opa3* deletion (Fig. 6d, e and Supplementary Fig. 18a). Subsequent calcein AM/CoCl_2_ staining revealed a marked increase of mitochondrial permeability transition pore (mPTP) opening in AMCMs from *Opa3*^KO^ mice (Fig. 6d, e). Similar mitochondrial dysfunction was observed in NMCMs infected with Ad-sh*Opa3*, as evidenced by reduced ΔΨm and increased mPTP opening, which was further exacerbated after PE stimulation (Fig. 6f, g and Supplementary Fig. 18b). In addition, oxygen consumption rate (OCR) was determined using Seahorse XF-96 Metabolic Flux analyzer. Our data revealed that the deficiency of *Opa3* reduced the OCR in both AMCMs and NMCMs (Fig. 6h–l), accompanied by reduced ATP production (Fig. 6j, m). Additionally, mitochondrial dysfunction was also observed in AMCMs isolated from *Opa3*^iKO^ after TAC surgery (Supplementary Fig.19a–e). Taken together, these data demonstrate that OPA3 is localized to the MOM in cardiomyocytes and consistent with a significant role of OPA3 on mitochondrial-SR communication.

### OPA3 multimerization is required for its interaction with pln and maintenance of mitochondria-SR homeostasis

**Fig. 7:**
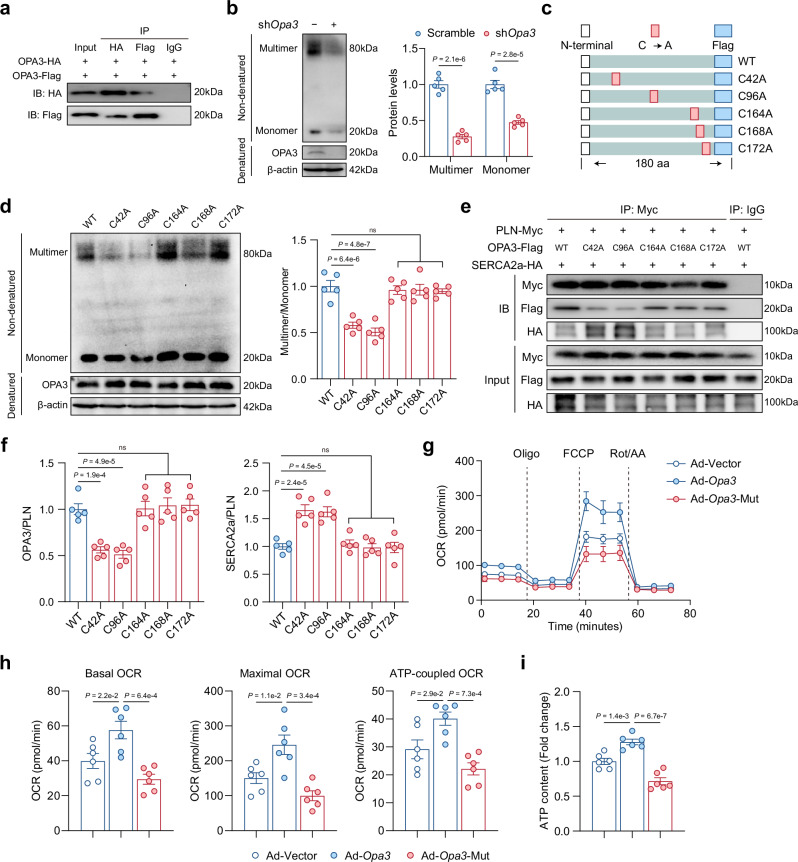
OPA3 multimerization is required for its interaction with PLN and maintenance of mitochondria-SR homeostasis. **a** Multimerization of OPA3 in HEK-293T cells transfected with OPA3-Flag- and HA-tagged OPA3 plasmids. Exogenous OPA3-Flag or OPA3-HA were immunoprecipitated by anti-Flag or anti-HA antibody, respectively, using IgG as a negative control. The experiment was replicated independently at least three times. **b** Representative western blots (Left) and quantification (Right) of OPA3 monomer and multimer in native protein extraction from NMCMs. Cells were infected with adenovirus encoding scramble (Scramble) or shRNA targeting *Opa3* (sh*Opa3*) for 48 h (*n* = 5 per group). **c** Schematic diagram illustrating the construction of the wild-type (WT) OPA3 plasmid and mutated plasmids carrying single cysteine-to-alanine mutation at each cysteine site. **d** Representative western blots (*Left*) and quantification (*Right*) of multimer/monomer ratio in native protein extraction from HEK293T cells. Cells were transfected with WT or serial mutated OPA3 plasmids for 48 h (*n* = 5 per group). **e, f** Coimmunoprecipitation of the WT OPA3, Mutated OPA3, SERCA2a-HA and PLN-Myc in HEK293T cells co-transfected with indicated plasmids. Exogenous PLN was immunoprecipitated by anti-Myc antibody, using IgG as a negative control (*n* = 5 per group). Analysis (**g**) and quantification (**h**) of mitochondrial OCR in indicated groups (*n* = 6 per group). The dash lines indicate the sequential addition of oligomycin (Oligo), FCCP, and Rot/AA. **i** ATP content in NMCMs of each group (*n* = 6 per group). Data are expressed as mean ± SEM. Data in **b** were analyzed by two-sided Student’s *t* test. Data in **d**, **f**, **h**, **i** were analyzed by one-way ANOVA followed by the Tukey post hoc test. AA indicates antimycin A, ATP adenosine triphosphate, FCCP carbonyl cyanide 4-(trifluoromethoxy) phenylhydrazone, IgG immunoglobulin G, NMCM neonatal mouse cardiomyocyte, OCR oxygen consumption rate, OPA3 optic atrophy 3, PLN phospholamban, Rot rotenone, SERCA2a sarcoplasmic/endoplasmic reticulum Ca^2+^-ATPase 2a, SR sarcoplasmic reticulum.

PLN exists as an equilibrium between pentamers and monomers that can bind to and inhibit SERCA2s^33–35^. In light of our observed OPA3-PLN association, we aimed to test whether OPA3 similarly forms a multimeric structure. The co-IP assays, followed by western blot analyses using HEK293T cells co-transfected with OPA3-Flag and OPA3-HA, indicated that OPA3 is present in a multimeric form (Fig. 7a). The multimeric nature of OPA3 protein was further validated by non-denatured PAGE, showing *Opa3* knockdown markedly diminishing both monomeric and multimeric OPA3 forms (possibly a tetramer), indicating the specificity of the detected bands (Fig. 7b). Predictive modeling via AlphaFold 2^36^ further supported this possible tetramerization, presenting tetrameric structure of OPA3 with high confidence scores, indicating a strong accuracy of predicted model (Supplementary Fig. 20a).

Multimers are sometimes linked by covalent bonds, such as disulfide bonds^37^. Thus, we first determined whether OPA3 multimers were sensitive to the reducing agent. We observed that OPA3 exhibited a significantly reduced ability to form multimers when treated with dithiothreitol (DTT), indicating that OPA3 multimers are stabilized by disulfide bonds (Supplementary Fig. 20b). To explore whether the multimerization of OPA3 is required for its biological function, we introduced single cysteine-to-alanine mutations at each cysteine site (C42A, C96A, C164A, C168A, and C172A) (Fig. 7c). Notably, OPA3 carrying C42A or C96A mutation showed a reduced capacity to form multimers (Fig. 7d) and exhibited diminished interaction with PLN (Fig. 7e, f). Furthermore, these more monomeric OPA3 mutants led to increased PLN-SERCA2a interaction (Fig. 7e, f) that would limit SR Ca^2+^-ATPase function. Subsequently, adenovirus encoding *Opa3* harboring both C42A and C96A mutations (*Opa3*-Mut) was constructed and introduced into NMCMs for further investigation. As expected, the overexpression of *Opa3* exhibited a significant increase in OCR, whereas *Opa3*-Mut showed a notable decline (Fig. 7g, h). Similarly, the *Opa3* overexpression enhanced ATPase activity, while the opposite effect was observed in *Opa3*-Mut (Fig. 7i). Moreover, the overexpression of *Opa3* significantly elevated PLN phosphorylation in NMCMs following PE stimulation, whereas *Opa3*-Mut inhibited this effect (Supplementary Fig. 21). Collectively, these data demonstrate that the multimerization of OPA3 is required for its interaction with PLN that promotes SERCA2a function (by limiting PLN binding) and for optimal mitochondrial respiration.

### OPA3 multimerization is required for its protective effect against TAC-induced HF and mitochondria-SR homeostasis

**Fig. 8:**
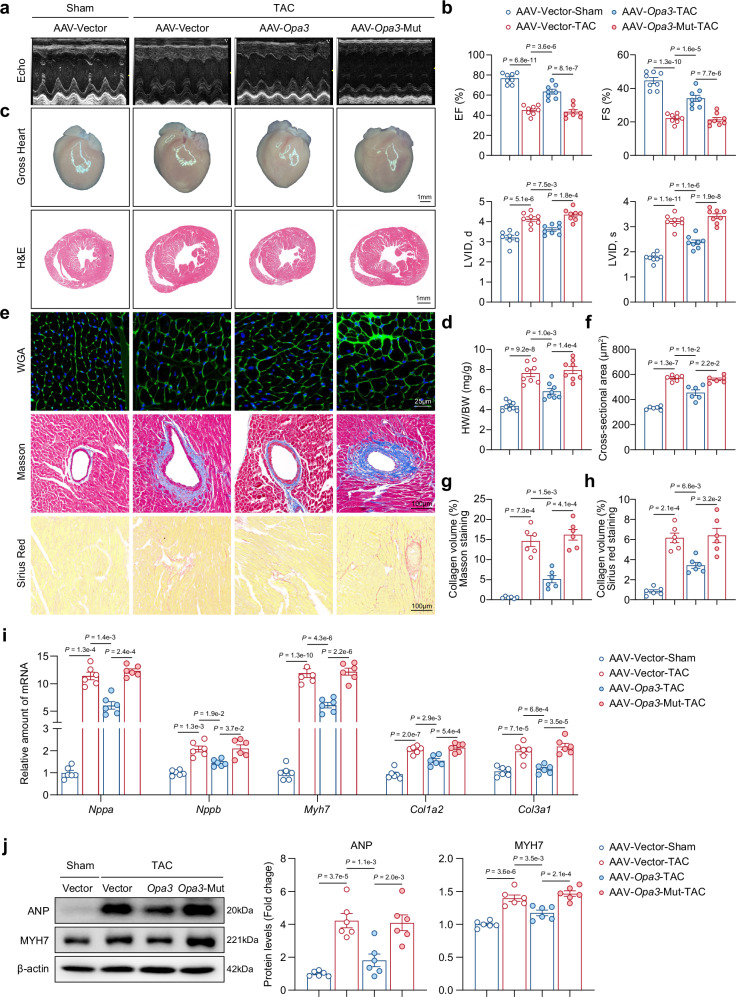
OPA3 multimerization is required for its protective effect against TAC-induced HF and mitochondria-SR homeostasis. Representative M-mode echocardiographic images (**a**) and corresponding measurements of EF, FS, LVID,d, and LVID,s (**b**) in *Opa3*^fl/fl^ mice received AAV-Vector, AAV-*Opa3*, and AAV-*Opa3*-Mut at 8 weeks after sham or TAC surgery (*n* = 8 per group). **c** Top, Representative gross images of hearts from indicated groups (scale bar, 1 mm). Bottom, Representative images of H&E-stained heart sections in indicated groups (scale bar, 1 mm). **d** Ratios of heart weight to body weight (HW/BW) in indicated groups (*n* = 8 per group). **e** Representative images of heart tissues stained with WGA, Masson trichrome, and Sirius red in indicated groups (scale bar, 25 μm for WGA; 100 μm for Masson trichrome and Sirius red). **f** Quantification of mean cardiomyocyte cross-section area of WGA-stained sections in (**e**) (*n* = 6 per group). Quantification of fibrotic area (%) determined by Masson trichome staining (**g**) (*n* = 6 per group) and Sirius red staining (**h**) of heart sections presented in (**e**) (*n* = 6 per group). **i** Relative gene expression in the heart tissues from indicated groups (*n* = 6 per group). **j** Left, Representative western blot and quantification of ANP and MYH7 in the heart tissues from indicated groups (n = 6 per group). Data are expressed as mean ± SEM. Data in **b**, **d**, **g**, **h**, **j** were analyzed by one-way ANOVA followed by the Tukey post hoc test. Data in **f**–**h**, **i** were analyzed by Welch’s ANOVA test followed by a post-hoc analysis using the Tamhane T2 method. AAV indicates adeno-associated viruses, Col1a2 collagen type I alpha 2 chain, Col3a1 collagen type III alpha 1 chain, EF ejection fraction, FS fractional shortening, H&E hematoxylin and eosin, HF heart failure, LVID,d internal dimension of the left ventricle in diastole, LVID,s internal dimension of the left ventricle in systole, Myh7 myosin heavy chain 7, Nppa (Anp) natriuretic peptide type A, Nppb (Bnp) natriuretic peptide type B, OPA3 optic atrophy 3, SR sarcoplasmic reticulum, TAC transverse aortic constriction, WGA wheat germ agglutinin.

To test whether OPA3 multimerization is required to protect against TAC-induced HF, we generated adeno-associated virus serotype 9 (AAV9) carrying a *cTnT* promoter to overexpress either wild-type *Opa3* (AAV-*Opa3*) or its mutant carrying C42A and C96A mutation (AAV-*Opa3*-Mut) specifically within cardiomyocytes. AAV-*Opa3* or AAV-*Opa3*-Mut was delivered into *Opa3*^fl/fl^ mice to induce gene overexpression 2 weeks before TAC surgery. We found that overexpression of AAV-*Opa3* limited the TAC-induced reduction in EF and FS, as well as the left ventricular dilation, whereas the AAV-*Opa3*-Mut failed to elicit significant improvement (Fig. 8a, b and Supplementary Table 10). Moreover, AAV-*Opa3* ameliorated the cardiac enlargement, cardiomyocyte hypertrophy, and fibrosis induced by TAC, a benefit not observed with the mutant variant (Fig. 8c–h). Furthermore, AAV-*Opa3* resulted in a notable decrease in biomarkers associated with HF and fibrosis, while AAV-*Opa3*-Mut showed no significant alteration vs. the control AAV-vector TAC animals (Fig. 8i, j).

We also tested whether in vivo overexpression of *Opa3* in TAC mice improved mitochondrial function within AMCMs. We found that overexpression of *Opa3* reduced mitochondrial depolarization and mPTP opening induced by TAC, a protective effect not evident in AAV-*Opa3*-Mut (Supplementary Fig. 22a, b). Additionally, AAV-*Opa3* limited the decline in OCR and ATP generation induced by TAC, while AAV-*Opa3*-Mut showed no significant improvement (Supplementary Fig. 22c–e). AAV-*Opa3* also diminished the increase in ER/SR stress proteins and prevented the decline in PLN phosphorylation levels after TAC, whereas AAV-*Opa3*-Mut showed minimal changes (Supplementary Fig. 23). Moreover, a decrease in the mitochondrial-associated membrane (MAM) protein mitofusin 2 (MFN2)^38^ was detected in heart tissues post-TAC, which was restored by AAV-*Opa3* but not AAV-*Opa3*-Mut (Supplementary Fig. 23).

Finally, we tested whether OPA3 was involved in TAC-induced deficiency of cardiomyocyte Ca^2+^ handling and contraction. Remarkable reductions in both the amplitude of cell shortening and the maximum upstroke velocity of cell shortening were observed in AMCMs isolated from the control TAC group (*Opa3*^fl/fl^-AAV-Vector-TAC) compared to sham group (*Opa3*^fl/fl^-AAV-Vector-Sham), which were exacerbated in the *Opa3*^iKO^-AAV-Vector-TAC group (Supplementary Fig. 24a–c). However, these adverse changes were reversed by *Opa3* overexpression (*Opa3*^fl/fl^-AAV-*Opa3*-TAC) but not *Opa3* mutant overexpression (*Opa3*^fl/fl^-AAV-*Opa3*-Mut-TAC) (Supplementary Fig. 24a–c). Parallel results were seen in Ca^2+^ transient analysis (Supplementary Fig. 24d–k). That is, the control TAC group (*Opa3*^fl/fl^-AAV-Vector-TAC) exhibited elevated diastolic [Ca^2+^]_i_ lower Ca^2+^ transient amplitudes and kinetics and lower SR Ca^2+^-ATPase activity and SR Ca^2+^ load vs. their sham group (*Opa3*^fl/fl^-AAV-Vector-Sham). All of these negative TAC effects were exacerbated in *Opa3* KO mice subjected to TAC (*Opa3*^iKO^-AAV-Vector-TAC), largely unimproved when the more monomeric OPA3 mutant was expressed (*Opa3*^fl/fl^-AAV-*Opa3*-Mut-TAC), but largely reversed toward the sham control when normal OPA3 was overexpressed (*Opa3*^fl/fl^-AAV-*Opa3*-TAC).

Taken together, these results collectively underscore that the multimerization of OPA3 is required for its protective effect against TAC-induced HF and mitochondrial and SR function.

## Discussion

In the present work, we identified a role of cardiac OPA3 in the pathogenesis of HF. Several key observations were made. First, the expression level of OPA3 was downregulated in failing heart tissues from both humans and mice, which was regulated by CREB. Second, cardiomyocyte-specific *Opa3* deficiency caused defects in SR Ca^2+^ handling and mitochondrial function, which may contribute to the development of HF. Third, OPA3 protein can multimerize, is localized to the outer mitochondrial membrane and associates with the SR protein PLN (particularly when OPA3 is multimerized). OPA3 alters both SR Ca^2+^ handling and mitochondrial function, possibly via mitochondria-SR communication. Finally, we showed that cardiomyocyte-specific overexpression of *Opa3* suppressed doxorubicin- and TAC-induced HF and the associated mitochondrial and SR dysfunction. Collectively, these findings offer the evidence for a regulatory role of OPA3 in cardiac myocytes and HF.

OPA gene family primarily reflects a shared clinical phenotype rather than structural or functional homology. Indeed, OPA family members differ substantially in their molecular architecture and cellular roles. For instance, OPA1 is well-documented for its critical role in maintaining mitochondrial homeostasis and cardiac function^10,12,14^. OPA1 contains a GTPase domain that facilitates nucleotide-dependent dimerization, driving conformational changes and oligomer stabilization essential for MIM fusion^39^. Imbalanced OPA1 processing leads to mitochondrial fragmentation and disruption of cardiac metabolism, which have been implicated in progression of DCM^12^. In contrast, OPA3 exhibits minimal sequence homology with OPA1 and lacks a GTPase domain, and its biological functions have remained far less understood. Our findings reveal that OPA3 also undergoes multimerization, a mechanism fundamentally distinct from the GTPase-driven oligomerization of OPA1. There was also controversy regarding the localization of OPA3 to the MIM vs. MOM in earlier studies^18,19^. OPA3 was initially thought to be located in the MIM based on sequence predictions^19^, but our research demonstrated that OPA3 mainly localized to the MOM in cardiomyocyte mitochondria, aligning with prior observations in HeLa cells^18^. Taken together, the distinction between OPA1 and OPA3 provides important context for interpreting the role of OPA3 uncovered in our study and highlights the need to consider OPA3 as a functionally independent regulator in cardiac physiology rather than as a structural or evolutionary analogue of OPA1.

Ca^2+^ is fundamental for excitation-contraction coupling in cardiomyocytes^40–42^, which can be regulated by the L-type Ca^2+^ channel, ryanodine receptor 2, and SERCA2a^32^. Defects in Ca^2+^ handling proteins are common features of HF^43^. Here, we showed that the cardiomyocyte-specific *Opa3* ablation led to impaired Ca²⁺ handling. To determine whether these defects represent a primary consequence of OPA3 loss or a secondary effect of the HF phenotype, we performed acute knockdown of OPA3 in isolated AMCMs from *Opa3*^fl/fl^ mice in the absence of systemic hemodynamic stress. This approach was sufficient to alter Ca²⁺ transients and SERCA2a activity, indicating that Ca^2+^ dysregulation arises directly from OPA3 deficiency and occurs prior to the development of overt heart failure. Prior research has linked the alteration of SERCA2a activity to the onset of DCM^44^. Heterozygous knockout of SERCA2 has been shown to accelerate the progression of HF induced by TAC^45^, whereas increasing SERCA2a levels in the myocardium alleviated cardiac dysfunction^46^. Although gene therapy aimed at enhancing SERCA2a activity showed promise in the management of patients with HF^47^, the inadequate restoration of SERCA2a in failing human hearts may have undermined the success of a SERCA2a gene therapy in a phase 2 clinical trial^48^. SERCA2a activity is known to be modulated by interactions with various partners, particularly by PLN^31^, which has also been implicated in the pathogenesis of DCM^30,49^. Specifically, PLN reduces the affinity of SERCA2a for Ca^2+^ under basal conditions. Upon sympathetic stimulation, PLN is phosphorylated by PKA, relieving the inhibitory effect and facilitating cardiac relaxation^34^. Inhibition of PLN by antisense oligonucleotides improves cardiac function in cardiomyopathy, indicating PLN as a potential therapeutic target in HF^50^. Our mechanistic analysis of OPA3 interactome revealed a close interaction between OPA3 and PLN. *Opa3* deficiency led to diminished PLN phosphorylation, enhanced interaction between PLN and SERCA2a, and thereby reduced SERCA2a activity. Collectively, our findings identified OPA3 as a critical PLN regulator stabilizing SERCA2a and normal Ca^2+^ handling in cardiomyocytes via interacting with PLN, suggesting a critical role of OPA3 in cardiomyocyte function.

PLN exists as both monomers and oligomers (pentamers), with the monomeric form directly binding to and inhibiting SERCA2a activity^51,52^. PLN phosphorylation, shifts the PLN equilibrium from monomers toward pentamers, thereby activating the SR Ca^2+^ pump^33^. Indeed, shifting PLN toward pentamers enhances SERCA2a function and cardiac function^34^. However, the molecular mechanisms governing the PLN/SERCA2a interaction have not been fully elucidated. Here, we show that OPA3 interacts with PLN and that interaction relies on OPA3 multimerization. We found that mutations at two critical OPA3 cysteine residues (C42A and C96A) impaired the OPA3 multimerization and weakened its interaction with PLN, leading to an increased PLN-SERCA2a interaction and reduced SERCA2a activity. Our findings unveil a tertiary structure of OPA3 and underscore its involvement in the regulatory dynamics of the PLN/SERCA2a complex, thus emphasizing the critical function of OPA3’s structural configuration in modulating cardiac Ca^2+^ handling and function.

Emerging evidence highlights that mitochondria-SR crosstalk is important for sustaining the normal integrated function of cardiomyocytes and for normal ATP production and Ca^2+^ signaling^53^. In HF, mitochondria experience structural alterations, leading to the detachment of intermyofibrillar mitochondria from the cytoskeleton and SR^54^. This aligns with our observations that spontaneous HF, due to *Opa3* deficiency, exhibited abnormally small mitochondria, disorganized myofibrils, and increased distance between mitochondria and SR. We observed that *Opa3* was involved in the regulation of MFN2, a key component of mitochondrial dynamics and MAM implicated in mitochondria-ER contacts and Ca^2+^ homeostasis^38,55^. Further, mutations in PLN are reported to be associated with dysregulation of the ER/mitochondria compartment, yet the specific mechanisms remain unclear^56^. Our research demonstrated that *Opa3* deficiency disrupted both mitochondria and SR function in cardiomyocytes, resulting in compromised mitochondrial function and Ca^2+^ handling, together with elevated ER/SR stress. Remarkably, these adverse effects were mitigated by overexpression of *Opa3*, an outcome not replicated with a multimerization-deficient form of OPA3 in the setting of HF. Thus, our results indicate that OPA3 may play a vital role in facilitating mitochondria-SR crosstalk, potentially through interaction with the SR protein PLN.

In summary, our study reveals that OPA3 is a critical functional mediator in mitochondria-SR communication within cardiomyocytes. Disruption of OPA3 function compromised its association with PLN and reduced SERCA2a activity and impaired SR and mitochondrial homeostasis, characterized by reduced ATP generation and Ca^2+^ handling, ultimately contributing to HF. These insights offer therapeutic potential for OPA3 in the management of HF.

## Methods

### Human studies

This study was conducted in accordance with the Declaration of Helsinki. Analysis of human heart tissues was approved by the ethics committee of Nanjing First Hospital of Nanjing Medical University (approval no. KY20190404-03-KS-01). We collected failing human heart tissue samples from the left ventricle of DCM patients who accepted heart transplants. Normal heart tissues were obtained from non-failing donors. Written informed consent was obtained from all the patients.

### Animal studies

Animal experiments were conducted with approval from the Animal Ethics Committee of Renji Hospital and followed the National Institutes of Health Guidelines for the Care and Use of Laboratory Animals. All animals were kept at 24 °C ± 2 °C, humidity of 40% ± 5%, under a constant 12-h light/dark cycle with free access to diet and water. Animals were monitored daily for general health status, including body weight, activity, posture, and signs of distress. Mice exhibiting severe distress, rapid weight loss (>20%), impaired mobility, or signs of advanced heart failure were euthanized. At the experimental endpoint, mice were anesthetized in an induction chamber using 2% isoflurane mixed with oxygen prior to sacrifice.

*Opa3*^fl/fl^ mice on the C57BL/6 J genetic background were constructed by Cyagen Biosciences Inc., with exon 2 of the *Opa3* gene being flanked by loxP sites. Cardiomyocyte-specific *Opa3* knockout male mice (*Opa3*^KO^) were generated by crossing *Opa3*^fl/fl^ mice with those carrying cardiomyocyte-specific Cre (*Myh6*-Cre). And inducible cardiomyocyte-specific *Opa3* knockout mice (*Opa3*^iKO^) were established by crossing *Opa3*^fl/fl^ mice with those carrying cardiomyocyte-specific MerCreMer (*Myh6*-MCM). To activate MCM, 6-week-old mice were administrated with tamoxifen intraperitoneally at a dosage of 30 mg/kg/day for five consecutive days.

Transverse aortic constriction (TAC) procedure was performed as follows^57^. Briefly, 8-week-old mice were anesthetized with a mixture of 2% isoflurane/oxygen in an induction chamber. Then the mice were subjected to ligation of the transverse aorta between the innominate and left common carotid arteries with a 6-0 suture against a 27 G needle, which was immediately removed after ligation. The identical procedure without constriction served as the sham surgery.

To investigate the therapeutic role of *Opa3* overexpression in HF, we constructed adeno-associated virus (AAV) serotype 9 vectors containing the cardiac troponin T promoter to drive the expression of wild-type *Opa3* (AAV-*Opa3*) or mutated *Opa3* (AAV-*Opa3*-Mut). 6-week-old male mice were then injected with a total of 5 × 10^11^ viral particles through tail vein to induce *Opa3* overexpression in cardiomyocytes. The overexpression efficacy of *Opa3* was confirmed by western blot 14 d after injection.

To minimize potential bias, mice were randomly assigned to experimental groups using SPSS-generated random numbers prior to treatment. All animal procedures were conducted by experienced investigators who were blinded to group allocation during both experimentation and data collection. No animals were excluded from the study unless clear evidence of technical failure was identified. Sample sizes were determined based on prior experimental experience and established literature. In this study, we primarily used male mice to reduce variability associated with hormonal fluctuations. However, this represents a limitation of the present work, as potential sex-specific differences were not evaluated. Future studies incorporating both male and female mice will be necessary to determine whether OPA3 exerts sex-dependent effects in the heart.

### Echocardiography measurements

To assess the cardiac function of the mice, echocardiography measurements were conducted by an echocardiographic imaging system (Vevo 2100; Visual Sonic, Canada) at indicated times. The mice were anesthetized with 1.5–2% isoflurane, and their body temperature was maintained at 37 °C during the procedure. Measurements were taken from three cardiac cycles and then averaged to ensure accuracy. Image measurement and analysis were performed by researchers who were blinded to the treatment.

### Hemodynamic Measurements

Mice were anesthetized with 1.5–2% isoflurane, and the right carotid artery was carefully isolated and cannulated with a 1.4 French micro-manometer (Millar Instruments, Houston, TX). The catheter was gently inserted into the left ventricle (LV) to measure and record LV pressure, maximal ascending and descending rates of LV pressure ( ± dP/dt), and heart rate (HR) using the PowerLab System (AD Instruments Pty Ltd., USA). Measurements were obtained at baseline and following administration of the β-adrenergic receptor agonist isoproterenol (0.1–10 ng).

### Histological analysis

Hearts were fixed in 4% paraformaldehyde and embedded in paraffin. Paraffin-embedded heart sections were stained with hematoxylin and eosin (H&E) for histopathology and with Sirius red or Masson’s Trichrome to clarify collagen deposition. Stained sections were digitized using a NanoZoomer S360 Digital slide scanner (Hamamatsu, Japan) for subsequent analysis. To calculate cardiomyocyte cross-sectional areas, the slides were stained with FITC-conjugated wheat germ agglutinin (WGA, Invitrogen, USA). Images were captured using a fluorescence microscope and analyzed with Image J software (Version 1.48 v, NIH, USA).

For immunofluorescence staining, heart sections were subjected to deparaffinization, rehydration, and antigen retrieval using citrate buffer. Following this, the sections were blocked with 5% bovine serum albumin (BSA) and incubated overnight at 4 °C with primary antibodies against OPA3 and PLN. Then, the sections were incubated with Alexa Fluor-conjugated secondary antibodies (Invitrogen, CA, USA) for 1 h, followed by nuclear staining with DAPI (Beyotime Biotechnology, China) for 5 min at room temperature. Super-resolution imaging was performed using AX with NSPARC microscope (Nikon, Japan).

### Cryo-immunogold electron microscopy

Mouse heart tissues were processed for cryo-immunogold electron microscopy using the Tokuyasu method. Briefly, heart tissues were rapidly excised and fixed in 2% paraformaldehyde and 0.5% glutaraldehyde in 0.1 M PBS (pH 7.2–7.4) for 4 h at 4 °C. After washing, samples were sequentially infused with 2% and 10% gelatin at 37 °C, followed by infusion with 2.3 M sucrose in PBS (three 20-min incubations and overnight at 4 °C). Tissue blocks were mounted and rapidly frozen in liquid nitrogen. Ultrathin sections (80 nm) were cut at −110 to −120 °C and collected using formvar/carbon coated specimen grid. For immunolabeling, sections were blocked with blocking solution (5% bovine serum albumin, 2% goat serum and 0.1% cold water fish gelatin in PBS) for 20 min at room temperature, and incubated with primary antibodies for 1 h at 37 °C, followed by incubation with gold-conjugated secondary antibody for 1 h at room temperature. Sections were post-fixed with 1% glutaraldehyde, washed, and contrasted with methyl cellulose/uranyl acetate (9:1) on ice. Grids were air-dried and examined using a transmission electron microscope.

### Transmission Electron Microscopy (TEM)

Mice were anesthetized and sacrificed, and heart tissues were immediately separated, fixed, stained, and sliced into ultrathin sections (70 nm) sections. The samples were mounted on a grid and imaged using Talos™ L120C TEM (Thermo Fisher Scientific, USA). TEM morphometric analysis of mitochondria and the mitochondria-SR distance was performed using ImageJ/FIJI (NIH).

### Cell isolation and culture

Adult mouse cardiomyocytes (AMCMs) were isolated from both *Opa3*^fl/fl^ and *Opa3*^KO^ mice via a Langendorff-based protocol. In brief, mice were anesthetized and euthanized, and their chests were cut open to expose the heart. The aortic arch was cannulated using a 20 G needle and perfused with type II collagenase (LS004176; Worthington Biochemical Corp., USA) solution (1 mg/ml) for 10 min. The digested ventricles were pulled into 1-mm pieces using fine-tip forceps and gently dissociated by pipetting. The cells were then subjected to sequential centrifugation at 20 *g* for 3 min each to gradually reintroduce calcium and achieve physiological calcium levels. The enriched cardiomyocytes were resuspended in prewarmed plating media and seeded onto laminin-coated (5 μg/ml) culture plates in a humidified tissue culture incubator (37 °C, 5% CO_2_).

Neonatal mouse cardiomyocytes (NMCMs) were isolated from 1-day-old neonatal C57BL/6 J mice (Jiesijie Laboratory Animal Center, Shanghai, China). In brief, hearts from neonatal mice were excised and dissociated in 0.125% trypsin (LT1720; Bioagrio, USA) at 4 °C overnight. Subsequently, the heart tissues underwent sequential digestion in type II collagenase (1 mg/ml, LS004176; Worthington Biochemical Corp., USA) at 37 °C. The cells were then collected and seeded in DMEM medium (SB-C002; Share-Bio, China) containing 10% fetal bovine serum for 1 h to reduce the nonmyocytes. Unattached cells (NMCMs) were collected and transferred to new culture plates for further experiments.

### Plasmid and recombinant adenoviruses construction

Plasmids encoding wild-type *Opa3*, mutated *Opa3*, *Atp2a2*, *Pln*, and *Creb* were cloned from mouse cDNAs and ligated to pcDNA3.1 vector. To generate the *Opa3* promoter reporter plasmid, mouse *Opa3* promoter region [Chr7:18960314–18962514 (+), GRCm39], which contained putative CREB binding site, was amplified from mouse genomic DNA and inserted into the pGL3-Basic vector to construct a firefly reporter. The pRL-TK vector (Renilla luciferase) from Promega served as an internal control to normalize transfection efficiency.

Adenoviruses encoding mouse GFP-tagged or Flag-tagged *Opa3* (both wild-type and C42/96 A mutated) were generated by Obio Technology Corporation (Shanghai, China). Adenoviruses encoding scramble or shRNA targeting *Opa3* were purchased from Hanbio Technology Corporation (Shanghai, China). The knockdown target sequence is available in Supplementary Table 1.

### Luciferase assay

Transfection for dual-luciferase reporter assay was performed in HEK293T cells. Luciferase activity was detected by the Dual-Luciferase Reporter Assay System (Promega, USA) according to the manufacturer’s instructions. Briefly, HEK293T cells were transfected with pGL3-*Opa3* reporter and pRL-TK-renilla vector, along with pcDNA3-*Creb* for 48 h. Cells were then harvested for firefly and renilla luciferase activity detection on Cytation 3 Cell Imaging Multi-Mode Reader (BioTek, USA). Renilla luciferase activity (pRL-TK) was used as an internal control for transfection efficiency. Results were expressed as fold changes of control group.

### Reverse transcription quantitative polymerase chain reaction (RT-qPCR)

Total RNA of cells or heart tissues was extracted using RNAiso Plus (9109; Takara, Japan). The extracted RNA was reverse transcribed to cDNA using HiScript III RT SuperMix (R323-01; Vazyme Biotech Co.,Ltd, China). Gene expression levels were quantified by qPCR, using ChamQ Universal SYBR qPCR Master Mix (Q711-03; Vazyme Biotech Co., Ltd, China) and LightCycler® 480 System (Roche, Germany). The 2^-(ΔΔCt)^ method was used to analyze the relative changes in gene expression. Primers used in qPCR are listed in Supplementary Table 1.

### Western blot

Total proteins from cells or heart tissues were extracted using a complete lysis buffer (Boster Biological Technology Co., Ltd., USA). Subcellular fractions, including proteins from mitochondria, cytosol, and nuclei, were isolated using the Tissue Mitochondria Isolation Kit (C3606; Beyotime Biotechnology, China) and Nuclear and Cytoplasmic Protein Extraction Kit (P0027; Beyotime Biotechnology, China). For denatured samples, proteins were separated by 7.5–15% SDS-PAGE gels (Epizyme Biotech, China) and electro-transferred to 0.2 μm or 0.45 μm PVDF membranes (Millipore, USA) according to the molecular weight of the target proteins, using Prestained Protein Ladder (SB-WB009; Share-Bio, China or WJ103; Epizyme Biotech, China) as a molecular weight marker. After blocking with 5% skim milk in TBST at room temperature, the membranes were probed with specific antibodies. Protein bands were detected using HRP-conjugated secondary antibodies (Jackson ImmunoResearch, USA) and ECL detection reagent (SB-WB012; Share-Bio, China). Band images were captured by ChemiDoc^TM^ Touch Imaging System (BIO-RAD, USA). Densitometric analysis of the protein bands was conducted using Image J software (Version 1.48 v, NIH, USA), with normalization to the loading controls. For non-denatured samples, proteins were extracted using a native lysis buffer (R0030; Solarbio, China) and then electrophoresed using the native PAGE gel system (C631101, Sangon Biotech, China). The subsequent steps were consistent with those described for denatured samples.

Due to the wide range of molecular weights of target proteins and non-specific bands observed with some antibodies, certain blots were generated from different gels to ensure accurate detection. Consistent loading controls were used across all blots within each experiment to maintain reliability and comparability. The antibodies used in this study are listed in Supplementary Table 2.

### Proteinase K assay

The mitochondrial fraction of heart tissues was isolated using the Tissue Mitochondria Isolation Kit (C3606; Beyotime Biotechnology, China). The fraction was then washed with IB buffer (220 mM mannitol, 70 mM sucrose, 10 mM HEPES/KOH, pH 7.6, 1 mM MgCl_2_, 1 mM DTT, and 1 mM EDTA). Proteinase K (10 μg/ml) was added to the samples in IB buffer, followed by incubation on ice for 30 min. Protease activity was terminated by the addition of SDS-PAGE sample buffer.

### Mito-tracker staining

To directly visualize mitochondria in AMCMs, the MitoTracker Red CMXRos (40741ES50; Yeasen, China) was used to detect mitochondria in accordance with the manufacturer’s instructions. Cells were incubated with MitoTracker Red CMXRo (200 nM) for 30 min at 37 °C. The morphology of mitochondria of cells was photographed by a confocal laser scanning microscope FV3000 series (Olympus, Japan).

### Determination of mitochondrial membrane potential (ΔΨm)

ΔΨm was assessed using JC-1 staining (ab113850; Abcam, USA). For AMCMs, cells were incubated with JC-1 dye (2 μM) or in culture medium for 30 min at 37 °C in the dark. For NMCMs, cells underwent initial infection with adenovirus carrying scramble or shRNA targeting *Opa3* for 48 h. Post-infection, cells were treated with phenylephrine (PE, 50 nM) or vehicle for additional 48 h. Following this, NMCMs were also stained with JC-1 dye (2 μM) in culture medium for 30 min at 37 °C in the dark. Fluorescence intensity in each group was analyzed by a confocal laser scanning microscope FV3000 series (Olympus, Japan).

### Determination of mitochondrial permeability transition pore (mPTP) Opening

mPTP opening were assessed using calcein AM/CoCl_2_ staining (C2009S; Beyotime Biotechnology, China). For AMCMs, cells were incubated with calcein-AM (2 μM) for 30 min at 37 °C in the dark, then washed twice with PBS. Subsequently, cells were incubated with CoCl_2_ (5 mM) in culture medium for an additional 30 min at 37 °C to quench calcein-AM fluorescence. For NMCMs, cells were first infected with adenovirus carrying scramble or shRNA targeting *Opa3* for 48 h, followed by treatment with PE (50 nM) or vehicle for an additional 48 h before staining. Fluorescence intensity in each group was analyzed by a confocal laser scanning microscope FV3000 series (Olympus, Japan).

### Measurement of the oxygen consumption rate (OCR)

The OCR was analyzed using a Seahorse XF96 analyzer (Agilent, USA), according to the manufacturer’s instructions. In brief, for NMCMs, cells were infected with indicated adenoviruses before being seeded (50,000 cells/well) in an XF96-well microplate (102601-100; Agilent, USA). These cells were further treated with PE (50 nM) for 48 h. For AMCMs, cells were isolated and seeded (3500 cells/well) in an XF96-well microplate. The cell-free wells served as the background control. The culture medium was replaced with Agilent Seahorse XF Media (Agilent, USA) supplemented with 1 mM pyruvate, 2 mM glutamine, and 10 mM glucose prior to the assay, and the cells were incubated for 1 h in the CO_2_-free incubator at 37 °C. During OCR assay, cells were sequentially exposed to 2 μM oligomycin, 1 μM FCCP, and 0.5 μM rotenone/antimycin. After the measurement, supernatants were carefully aspirated and proteins from each well were extracted. OCR values were then normalized to the total protein content of each well.

### Measurement of cell shortening and Ca^2+^ transients

AMCMs were evaluated for Ca^2+^ handling capacity by using the IonOptix Ca^2+^ recording system^58^. Briefly, we took the additional step of incubating AMCMs with 10 μM Fura-2 AM (Ca^2+^ indicator) for 20 minutes. After three washes with normal Tyrode’s solution (NT) solution containing 1.8 mM Ca^2+^, the cell suspension was collected. Additionally, 50 μL of the suspension was placed in a perfusion chamber containing 250 μL of Tyrode’s solution at a temperature of 25 °C. AMCMs were then subjected to electrical stimulation at different frequencies (0.5 Hz, 1 Hz, 2 Hz) to record cell shortening function and Ca^2+^ transient curves. These procedures enabled the collection of crucial data, including the amplitude of cell shortening/cell resting length, the maximum upstroke velocity of cell shortening ( + dL/dt_max_)/cell resting length, the amplitude of Ca^2+^ transient, and the maximal ascending rate in cell contractile Ca^2+^ transients ( + d[Ca^2+^]/dt_max_)/resting calcium, which could be analyzed by using specialized software. For the caffeine-induced cardiomyocyte Ca^2+^ transient experiment, the cardiomyocytes superfused with NT were paced at 1 Hz and Ca^2+^ transients were recorded. Thereafter, the stimulation was stopped and 10 mM caffeine dissolved in NT was immediately applied. The rate constant of decline of the electrically activated Ca^2+^ transients (rTOTAL) reflects the rate of combined transport of Ca^2+^ by SERCA2a, plasma membrane Na^+^/Ca^2+^ exchanger (NCX) and plasma membrane Ca^2+^-ATPase (PMCA). The rate constant of decline of the Ca^2+^ transient initiated by caffeine reflects the rate of combined transport of Ca^2+^ by NCX and PMCA through sarcolemma (rSL). In order to estimate the rate of Ca^2+^ transport by SERCA2a, rSL was subtracted from rTOTAL. In addition, the amplitude of caffeine-induced Ca^2+^ transients (SR Ca^2+^ content) was estimated from the amplitude of caffein-evoked Ca^2+^ transients in myocytes superfused with 0Na0Ca solution.

### Ca^2+^ spark and transient measurements

AMCMs were isolated and loaded with Fluo-4 AM dye (5 μM) for 30 min. Ca^2+^ transients were recorded by field stimulation at 0.5 Hz in normal Tyrode’s buffer. SR Ca^2+^ load was assessed by measuring the amplitude of Ca^2+^ transients induced by application of caffeine (10 mM). Images were captured using a confocal microscopy (Nikon, Japan) in line scan mode with excitation at 488 nm and emission at >505 nm. Image analysis was performed using ImageJ software and Sparkmaster plugin^59^.

### Ca^2+^ ATPase activity assay

The activity of Ca^2+^-ATPase was measured using the Ca^2+^-ATPase assay kit (A070-4; Jiancheng Bioengineering Institute, China). Heart tissues or cells were homogenized in physiological saline, centrifuged, and the supernatant was collected for subsequent analysis. The samples were then assayed according to the manufacturer’s instructions. Enzyme activity was expressed as U/mg protein.

### ATP content assay

ATP levels of NMCMs or AMCMs were determined using an Enhanced ATP Assay Kit (S0027, Beyotime Biotechnology, China). The cells were lysed, centrifuged, and the supernatant was collected for subsequent determination. The detection solution was added to a 96-well plate and incubated at room temperature for 5 min prior to ATP detection. The supernatant was then added to the plate and ATP content was detected using Cytation 3 Cell Imaging Multi-Mode Reader (BioTek, USA).

### Mitochondrial Ca^2+^ measurement

Cardiomyocytes expressing adenovirus-mediated Mitycam (Adeno-Mitycam) were used for mitochondrial Ca^2+^ measurements. Adenoviral gene transduction was performed at a multiplicity of infection of 500 virus particles per cell (vp/cell), followed by appropriate culture to ensure stable expression of the indicator. Cells were incubated in a Na^+^-free, Ca^2+^-buffered solution to establish a controlled intracellular ionic environment. To eliminate the contribution of SR Ca^2+^ handling, cells were pretreated with 5 μM thapsigargin to inhibit SR Ca^2+^-ATPase activity, thereby blocking both SR Ca^2+^ uptake and release. Mitycam fluorescence was excited at 488 nm and imaged using an Olympus fluorescence imaging system. Mitochondrial Ca^2+^ changes were quantified based on dynamic variations in fluorescence intensity and expressed in a normalized form. Spatial distribution of signals was visualized using pseudocolor images labeled “F”. In addition, fluorescence signals were extracted from selected regions of interest (ROIs) to generate time-dependent traces for quantitative assessment of mitochondrial Ca^2+^ dynamics^60^.

### RNA sequencing (RNA-seq) and bioinformatics analysis

Heart tissues and cells were collected, and total RNA was extracted. Transcriptome libraries construction and sequencing were performed by OE Biotech Co. Ltd. (Shanghai, China). Briefly, RNA integrity was initially assessed using the Agilent 2100 Bioanalyzer (Agilent Technologies, USA). The samples with RNA Integrity Number (RIN) ≥ 7 were subjected to the subsequent analysis. The libraries were constructed using VAHTS Universal RNA-seq Library Prep Kit for Illumina (Vazyme Biotech Co.,Ltd, China) according to the manufacturer’s instructions. Then these libraries were sequenced on the Illumina sequencing platform Illumina NovaSeq 6000 and 150 bp paired-end reads were generated.

Raw sequence reads were trimmed and quality-filtered using Trimmomatic^61^. Clean reads were aligned against the mouse genome (GRCm38) using hisat2^62^, and the read counts of genes were obtained by htseq-count^63^. Differentially expressed genes (DEGs) were identified using the DESeq2 package. To assess the impact of *Opa3* depletion on biological pathways, Kyoto Encyclopedia of Genes and Genomes (KEGG) enrichment analysis was conducted in R, utilizing the hypergeometric distribution for statistical assessment.

For cell type-specific expression analysis of the *Opa3* gene in human failing hearts, single-nucleus RNA sequencing (snRNA-seq) datasets of cardiac nuclei from healthy donors and patients with cardiomyopathy including DCM, hypertrophic cardiomyopathy (HCM), and ischemic cardiomyopathy (ICM) were downloaded from the Broad Institute’s Single Cell Portal (https://singlecell.broadinstitute.org/singlecell) under project ID SCP1303, SCP1849 and SCP1852. The dataset was processed using the Seurat (Version 5). Cells were categorized into clusters using UMAP dimensionality reduction, with cell types identified by established marker genes. Cell types in different clusters were defined by canonical cell markers. The *Opa3* gene expression in cardiomyocytes from both healthy donors and patients with cardiomyopathy was visualized using box plot, dot plot, and violin plot.

### Chromatin immunoprecipitation (ChIP)

ChIP assays were conducted using SimpleChIP® Plus Enzymatic Chromatin IP Kit (9005; Cell Signaling Technology, USA) according to manufacturer’s protocol. Briefly, cells were fixed by 1% formaldehyde for 10 min at room temperature to facilitate protein-DNA crosslinking. The reaction was stopped using a 0.125 M glycine solution for 5 min. The extracted chromatin was subsequently digested with micrococcal nuclease for 20 min at 37 °C, followed by sonication for fragmentation. The fragmented chromatin was then immunoprecipitated using CREB antibody or IgG antibody at 4 °C overnight. After DNA purification, the DNA purity was checked using the NanoPhotometer® spectrophotometer (IMPLEN, CA, USA) and the DNA concentration was measured using Qubit® DNA Assay Kit in Qubit® 2.0 Flurometer (Life Technologies, CA, USA). The purified DNA was used for qPCR (with specially designed primers). The primers for ChIP-qPCR are presented in Supplementary Table 1.

### Co-immunoprecipitation (Co-IP)

For the detection of endogenous protein interaction, heart tissues or AMCMs were used for protein extraction. For detection of exogenous protein interaction, HEK293T cells were transfected with indicated plasmids for 48 h prior to protein extraction. Then, the protein was extracted using NP-40 cell lysis buffer and subjected to incubation with specific antibodies at 4 °C overnight. The protein-antibody complexes were then purified by 20 µl of protein A/G magnetic beads (SB-PR001; Share-Bio, China) at 4 °C for 2 h, centrifuged and washed with Phosphate Buffered Saline (PBS) solution with the detergent Tween 20 (0.05%) for five times. The immunoprecipitated protein was further analyzed by western blot.

### Immunoprecipitation-mass Spectrometry (IP/MS)

AMCMs were infected with GFP-tagged or Flag-tagged *Opa3* overexpressing adenovirus for 48 h. IP was performed as described above. Following electrophoresis, the gels were stained with Coomassie Brilliant Blue (Beyotime, China), and the bands of interest were excised for analysis via liquid chromatography-mass spectrometry (LC-MS), which was carried out by OE Biotech Co., Ltd. (Shanghai, China). The functional implications of the proteins interacting with OPA3 were explored using KEGG enrichment and Gene Ontology (GO) analysis performed in R.

### Proximity-Ligation Assay (PLA)

Proximity-ligation assay was performed using NaveniFlex Cell Red kit (Navinci, Sweden) according to the manufacturer’s protocol. Briefly, AMCMs were fixed, permeabilized, and blocked before incubation with primary antibodies against PLN and OPA3 (1:100 in Naveni Diluent) for 60 min at 37 °C. After washing, cells were incubated with NaveniBody M1 and R2 probes (1:40) for 60 min at 37 °C. Reaction 1 was then applied by adding Naveni Buffer 1 and Enzyme 1 (1:40) and incubating for 30 min at 37 °C. Reaction 2 was performed in the dark using NaveniFlex Cell Buffer 2 Red and Enzyme 2 (1:40) for 90 min at 37 °C. Slides were then washed and counterstained with Mito-Tracker Green for fixed cells (C1048; Beyotime Biotechnology, China) and DAPI (Beyotime Biotechnology, China). Images were acquired using a confocal laser scanning microscope FV3000 series (Olympus, Japan).

### Statistics and reproducibility

All statistical analyses were conducted using GraphPad Prism 8.0.1. The Shapiro-Wilk test was applied to assess the normality of the data distribution initially. Comparisons between 2 groups were performed by the Student *t* test (equal variances) or Welch *t* test (unequal variances) for normally distributed variables. Non-normally distributed variables were analyzed using the Mann-Whitney *U* test. For multiple comparisons involving more than 2 groups, the Brown-Forsythe test was applied to assess variance homogeneity. If the data passed the equal variance test, a one-way ANOVA analysis was used followed by the Tukey post hoc test; otherwise, a Welch ANOVA test was performed followed by the Tamhane T2 method. Two-way ANOVA followed by Tukey multiple comparison test was used for comparing differences between >2 groups with two factors. Non-normally distributed variables were analyzed using Kruskal-Wallis tests followed by Dunn’s post hoc test. For hierarchically structured datasets, comparative analyses were performed using either nested Student *t* tests or one-way ANOVA. Survival rates were determined by the Kaplan-Meier method, and differences were assessed using a stratified log-rank test. Unless indicated otherwise, parametric data are expressed as mean ± SEM and nonparametric data are represented as median (interquartile range). All experiments were independently repeated at least three times using biological replicates (derived from independent experimental units or subjects). A value of *P* < 0.05 was considered statistically significant.

### Reporting summary

Further information on research design is available in the Nature Portfolio Reporting Summary linked to this article.

## Supplementary information

Supplementary Information

Reporting Summary

Transparent Peer Review file

## Source data

Source Data

## Data Availability

The RNA-seq data generated in this study have been deposited in National Center for Biotechnology Information (NCBI) under the project ID PRJNA1460849. Publicly available single-nucleus transcriptomic data of human cardiomyopathy are available through the Broad Institute’s Single Cell Portal (https://singlecell.broadinstitute.org/single_cell) under project ID SCP1303, SCP1849, and SCP1852. Public transcriptomic data from DCM murine model and corresponding controls are available on Gene Expression Omnibus under project ID GSE168610. All data supporting the findings of this study are available in the article, in the Supplementary Information, and in the Source Data file. Source data are provided with this paper.
