# Supplementary material for: Mitochondrial protein OPA3 sustains cardiac function by regulating calcium handling in male mice
Source: Nat Commun. 2026 Jun 17;17:7665. doi: 10.1038/s41467-026-73991-4 (PMC13434718; doi:10.1038/s41467-026-73991-4)
Supplement: Supplementary file 1 [file 41467_2026_73991_MOESM1_ESM.pdf]

Supplementary Figures

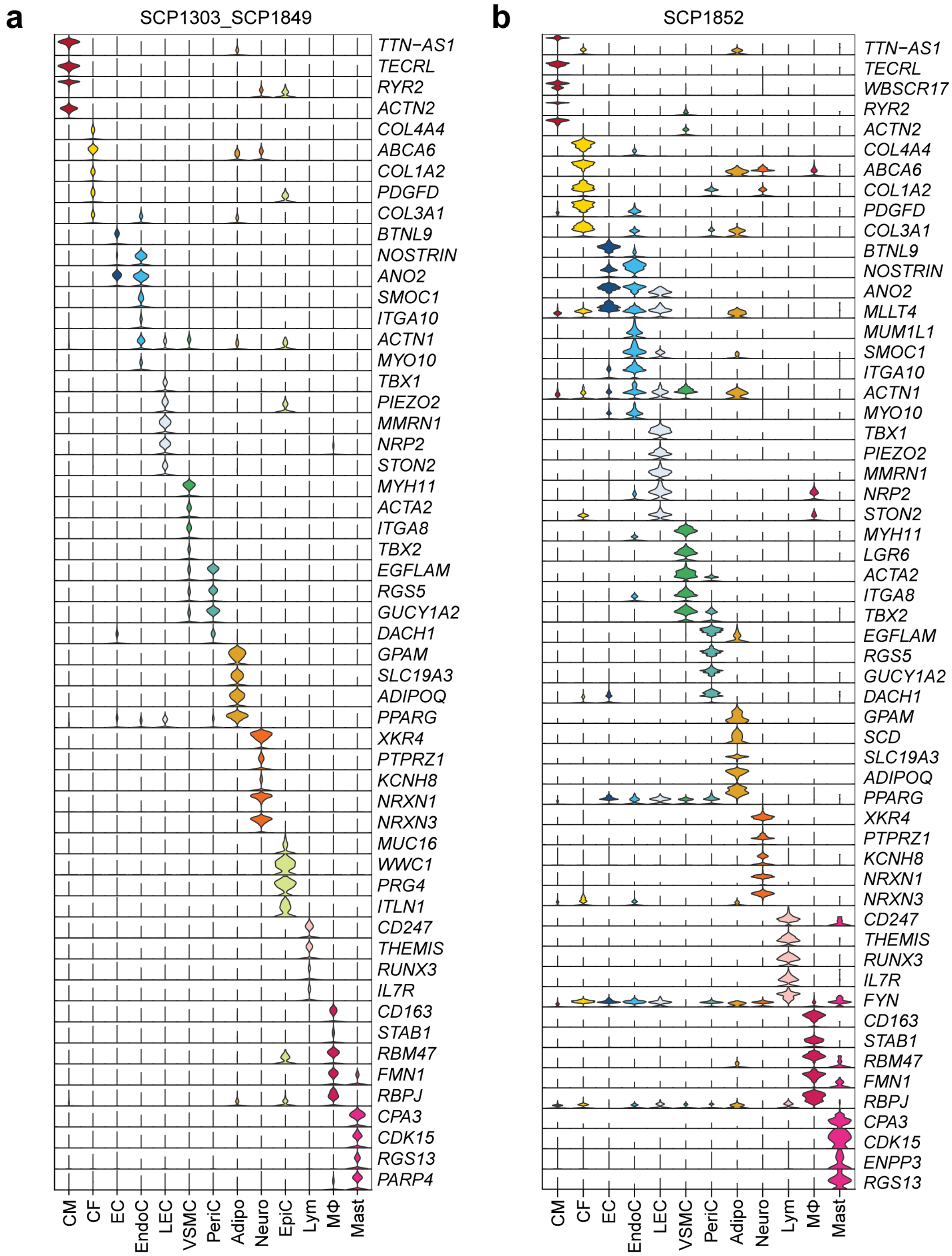

Supplementary Fig. 1. The expression distribution of selected signature genes.

a, The columns of the violin plot represent the 13 cell types identified in **Fig. 1a** and the rows represent the selected signature genes. **b**, The columns of the violin plot represent the 12 cell types identified in **Fig. 1i** and the rows represent the selected signature genes.

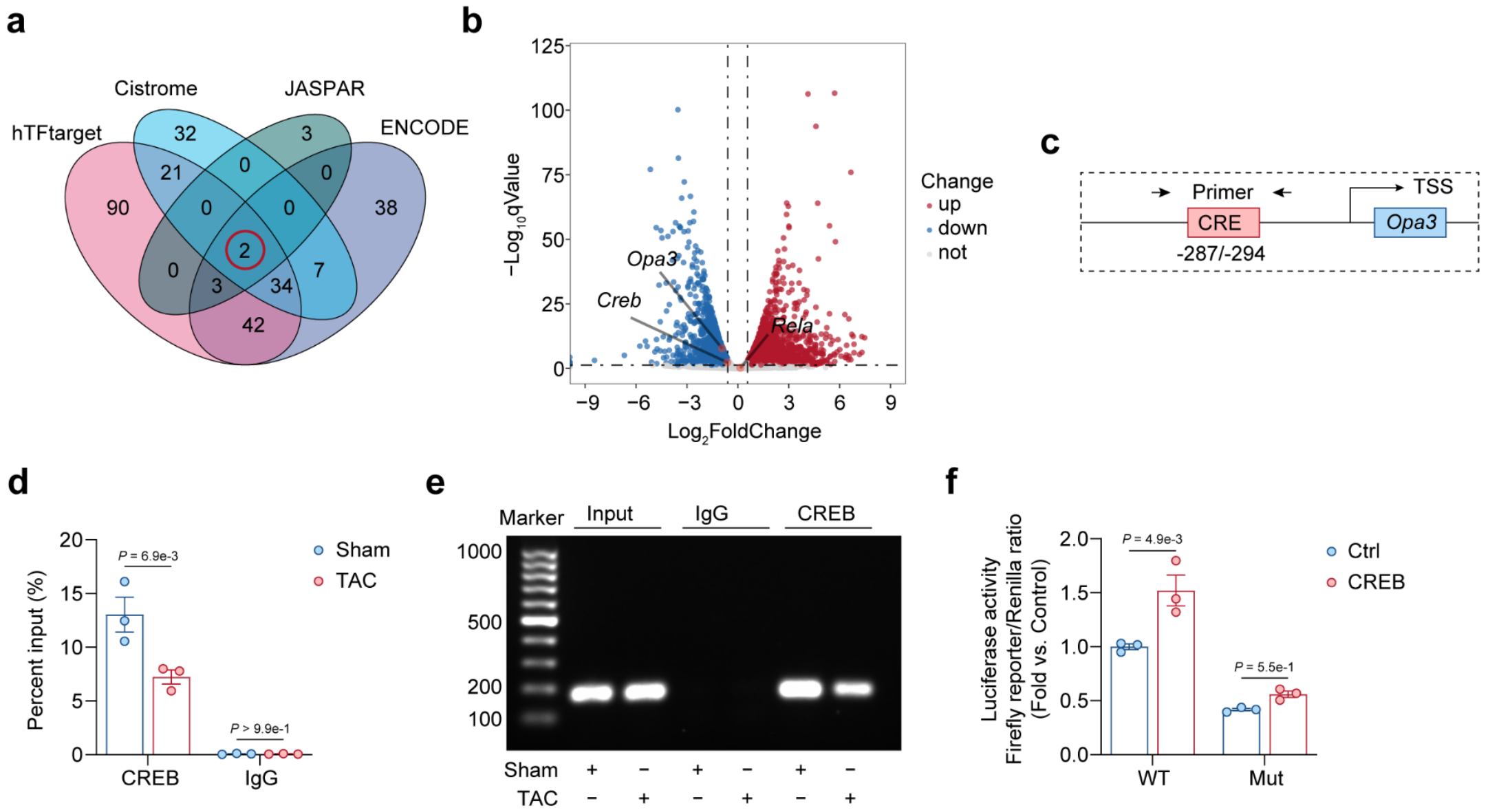

Supplementary Fig. 2. CREB is identified as a direct regulator of OPA3.

a, Venn diagram showing intersection of predicted transcription factors via hTFtarget, Cistrome, JASPAR, and ENCODE databases. **b**, Volcano plot revealing the magnitude and significance of altered genes in heart tissues from DCM and corresponding controls (GSE168610). DEGs were defined as genes with a Benjamini-Hochberg adjusted *P* <0.05 and $|\log_2(\text{fold change})| \geq 0.58$. **c**, Schematic diagram showing the conserved CRE site, as determined by online JASPAR analysis tool, within *Opa3* gene promoter. The position of primer pair (arrowheads) flanking the CRE site in *Opa3* promoter is indicated. **d** and **e**, ChIP-qPCR was performed for CREB or IgG occupancy at *Opa3* promoter fragments containing the CRE site in indicated groups (n=3 per group). **f**, *Opa3* transcriptional activity was analyzed in HEK293T cells co-transfected with overexpression plasmid for CREB and luciferase reporter driven by indicated promoter. pRL-TK-renilla plasmid was used as an internal control for transfection efficiency (n=3 per group). Data are expressed as mean ± SEM. Data in **d**, **f** were analyzed by two-way ANOVA followed by the Tukey post hoc test. CRE indicates cAMP response element; CREB, cAMP response element-binding protein; DCM, dilated cardiomyopathy; DEG, differentially expressed genes; OPA3, optic atrophy 3.

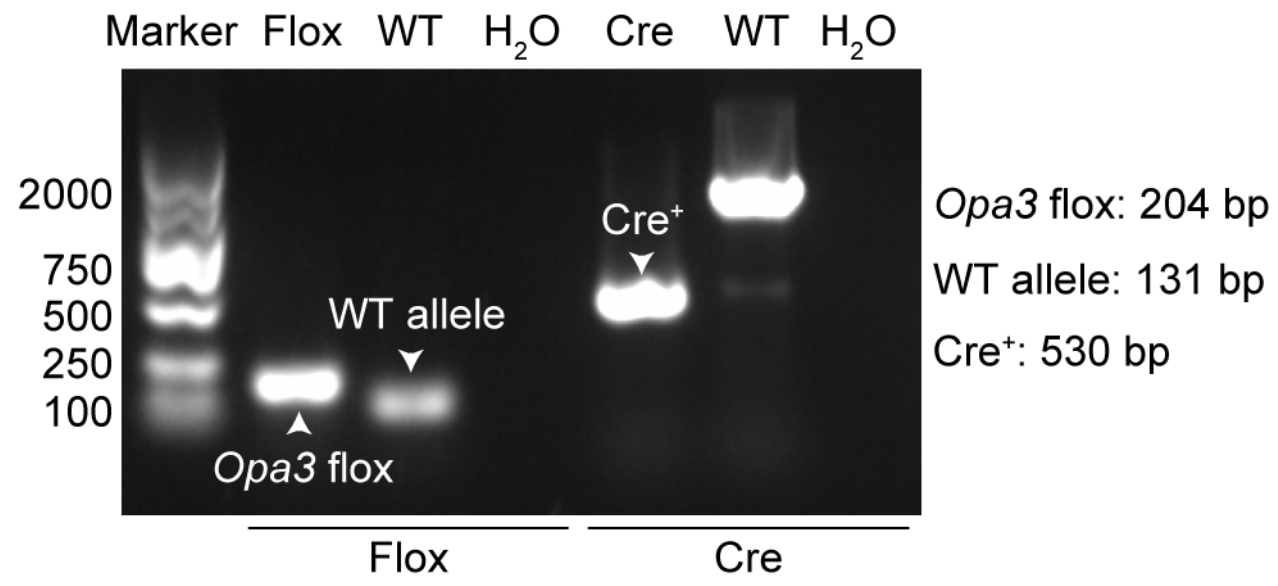

Supplementary Fig. 3. Construction of cardiomyocyte-specific *Opa3* knockout mouse strain.

Validation of the effective inactivation of OPA3 in the cardiomyocyte-specific *Opa3* knockout strain. PCR analysis was performed for the confirmation of successful gene editing. The 530 bp band represents *Myh6*-Cre. The 204 bp represents the mutation produced by insertion of Loxp sites, while the 131 bp represents the wild type. Opa3 indicates optic atrophy 3; PCR, polymerase chain reaction.

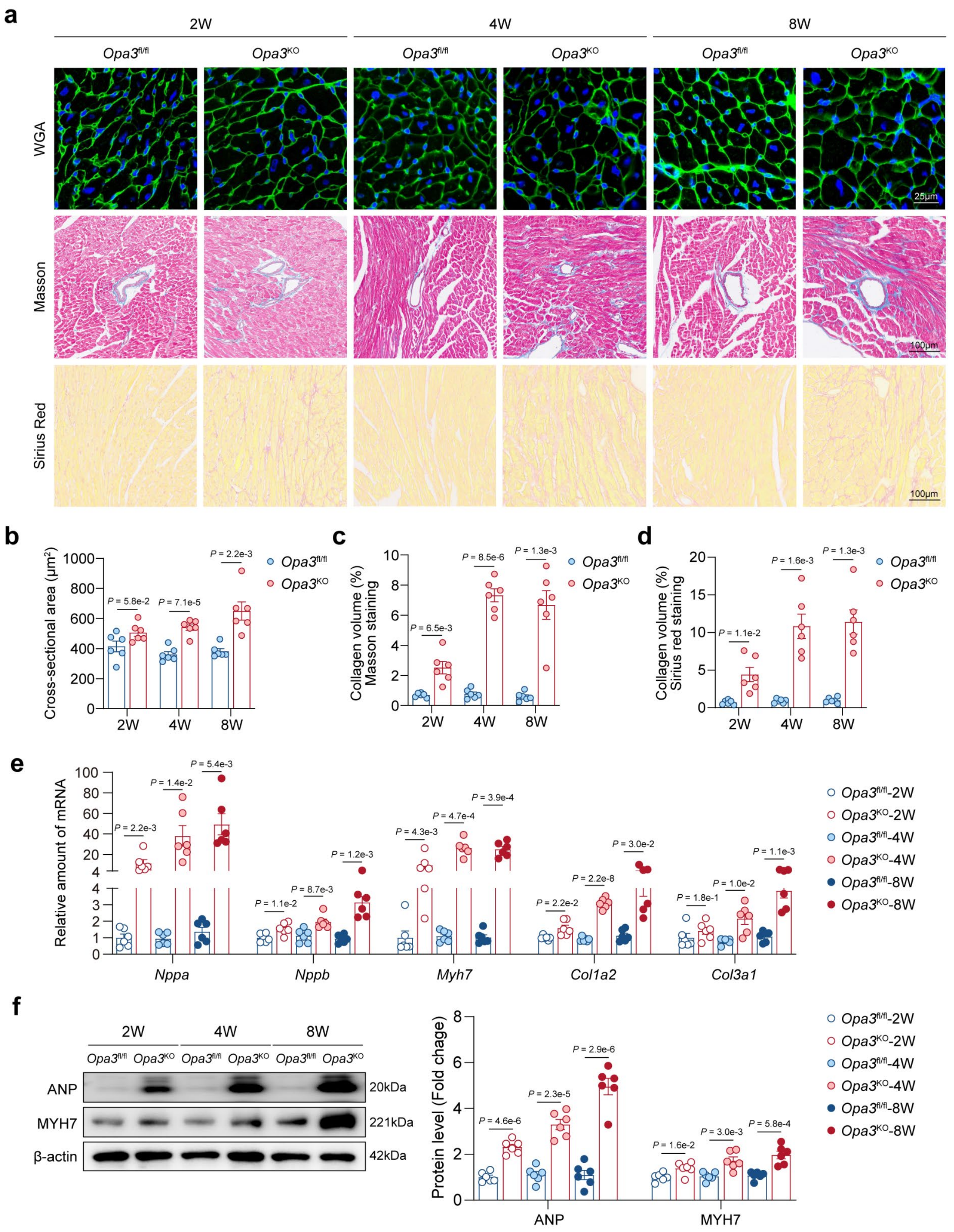

Supplementary Fig. 4. Cardiomyocyte-specific deficiency of *Opa3* exhibited typical histological characteristics of DCM.

a, Representative images of heart tissues stained with WGA, Masson trichrome, and Sirius red from *Opa3*$^{fl/fl}$ and *Opa3*^{KO} mice at 2 weeks, 4 weeks, and 8 weeks of age (scale bar, 25 μm for WGA; 100 μm for Masson trichrome and Sirius red). **b**, Quantification of mean cardiomyocyte cross-section area of WGA-stained sections in (**a**) (n=6 per group). Data were analyzed by two-sided Student's *t* test or Mann-Whitney

U test. **c** and **d**, Quantification of fibrotic area (%) as determined by Masson trichome staining (**c**) (n=6 per group) and Sirius red staining of heart sections (**d**) presented in (**a**) (n=6 per group). Data were analyzed by two-sided Welch's *t* test. **e**, Relative mRNA expression of *Nppa*, *Nppb*, *Myh7*, *Col1a2*, and *Col3a1* in the heart tissues from $Opa3^{fl/fl}$ and $Opa3^{KO}$ mice at indicated time points (n=6 per group). Data were analyzed by two-sided Student's *t* test (equal variances), Welch's *t* test (unequal variances), or the Mann-Whitney *U* test. **f**, **Left**, Representative western blots for ANP, MYH7, and β-actin in the heart tissues from $Opa3^{fl/fl}$ and $Opa3^{KO}$ mice at indicated time points. **Right**, Quantification of protein expressions in indicated groups shown in **Left** (n=6 per group). Data were analyzed by two-sided Student's *t* test. Data are expressed as mean ± SEM. Col1a2 indicates collagen type I alpha 2 chain; Col3a1, collagen type III alpha 1 chain; DCM, dilated cardiomyopathy; Myh7, myosin heavy chain 7; Nppa (Anp), natriuretic peptide type A; Nppb (Bnp), natriuretic peptide type B; Opa3, optic atrophy 3; WGA, wheat germ agglutinin.

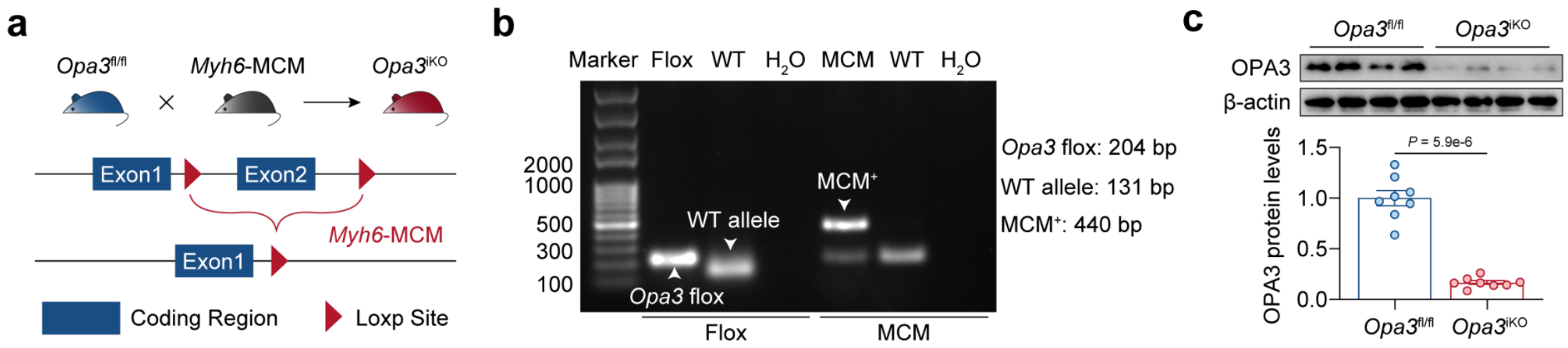

Supplementary Fig. 5. Construction of inducible cardiomyocyte-specific *Opa3* knockout mouse strain.

a, Schematic diagram illustrating the strategy of inducible cardiomyocyte-specific *Opa3* knockout mice construction. **b**, Validation of the effective inactivation of OPA3 in the cardiomyocyte-specific *Opa3* knockout strain. PCR analysis was performed for the confirmation of successful gene editing. The 440 bp band represents *Myh6*-MCM. The 204 bp represents the mutation produced by insertion of Loxp sites, while the 131 bp represents the wild type. **c**, **Top**, Representative western blots for OPA3 and β-actin in heart tissues from *Opa3*$^{fl/fl}$ and *Opa3*^{iKO} mice at 14 days after 5-day consecutive tamoxifen injections (40 mg/kg/d). **Bottom**, Quantification of protein expressions in indicated groups shown in **Top** (n=8 per group). Data were analyzed by two-sided Welch's *t* test. Data are expressed as mean ± SEM. MCM indicates MerCreMer; Opa3, optic atrophy 3; PCR, polymerase chain reaction.

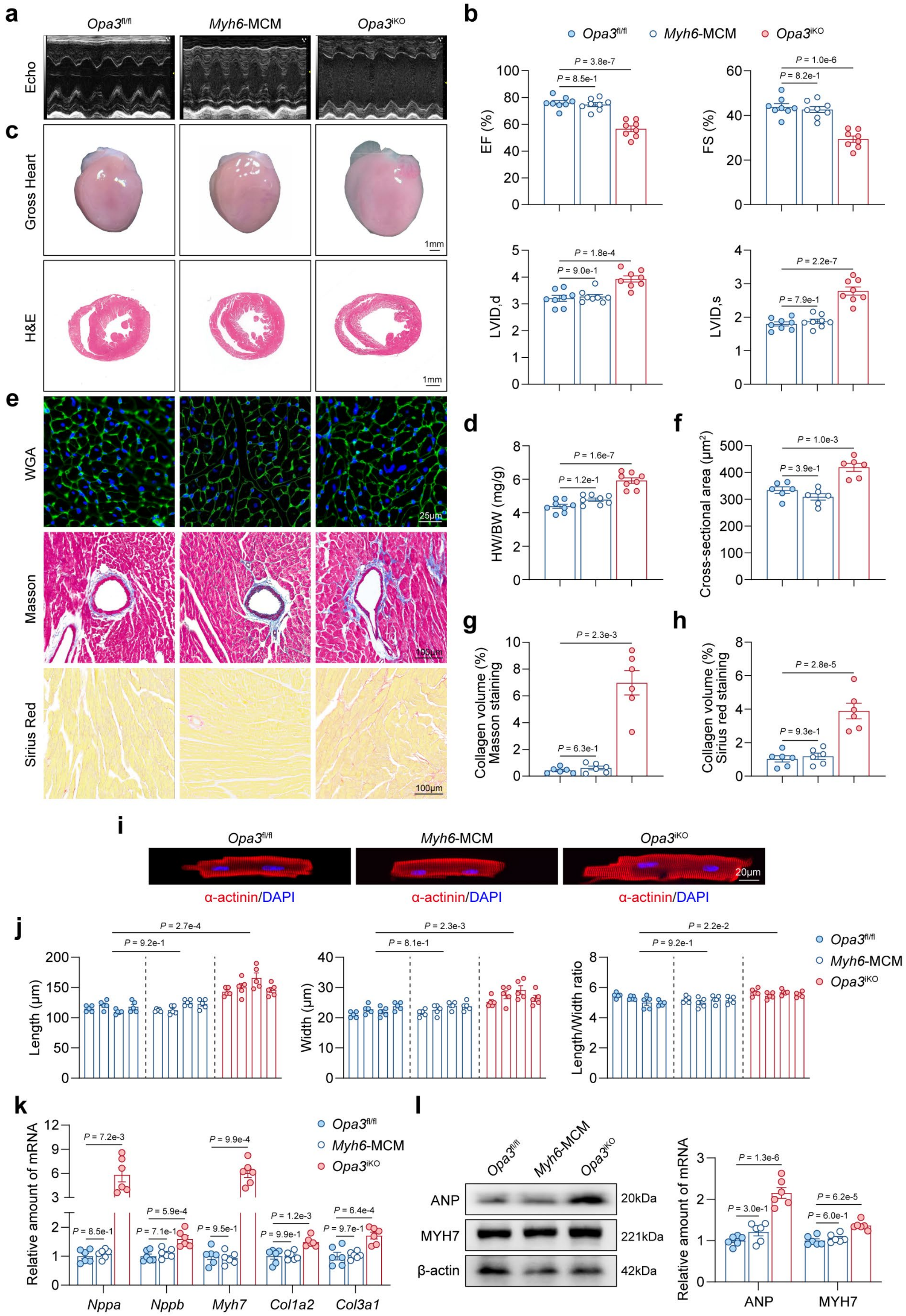

Supplementary Fig. 6. Adult cardiomyocyte-specific deficiency of *Opa3* leads to DCM.

a and **b**, Representative M-mode echocardiographic images (**a**) and corresponding

measurements of EF, FS, LVID,d, and LVID,s (**b**) of *Opa3*$^{fl/fl}$, *Myh6*-MCM, and *Opa3*^{iKO} mice 2 weeks after five consecutive days of tamoxifen administration (n=8 per group). **c**, Representative gross images of hearts and H&E-stained heart sections from indicated groups (scale bar, 1 mm). **d**, Ratios of heart weight to body weight (HW/BW) in indicated groups (n=8 per group). **e**, Representative images of heart tissues stained with WGA, Masson trichrome, and Sirius red from indicated groups (scale bar, 25 μm for WGA; 100 μm for Masson trichrome and Sirius red). **f**, Quantification of mean cardiomyocyte cross-section area of WGA-stained sections in (**e**) (n=6 per group). **g** and **h**, Quantification of fibrotic area (%) as determined by Masson trichome staining (**g**) (n=6 per group) and Sirius red staining (**h**) of heart sections presented in (**e**) (n=6 per group). **i**, Representative immunofluorescence images of α-actinin in isolated AMCMs from *Opa3*$^{fl/fl}$, *Myh6*-MCM, and *Opa3*^{iKO} 2 weeks after five consecutive days of tamoxifen administration (scale bar, 20 μm). **j**, Quantifications of cell length, width, and length/width ratio shown in (**i**) (5 cardiomyocytes per animal, 4 animals per group). **k**, Relative gene expression in the heart tissues from indicated groups (n=6 per group). **l**, Western blots and quantification of ANP and MYH7 in indicated groups (n=6 per group). Data are expressed as mean ± SEM. Data in **b**, **d**, **f**, **g**, **h**, **k**, **l** were analyzed by one-way ANOVA followed by the Tukey post hoc test. Data in **j** were analyzed by nested one-way ANOVA followed by the Tukey post hoc test. Col1a2 indicates collagen type I alpha 2 chain; Col3a1, collagen type III alpha 1 chain; DCM, dilated cardiomyopathy; EF, ejection fraction; FS, fractional shortening; H&E, hematoxylin and eosin; LVID,d, internal dimension of the left ventricle in diastole; LVID,s, internal dimension of the left ventricle in systole; MCM, MerCreMer; Myh7, myosin heavy chain 7; Nppa (Anp), natriuretic peptide type A; Nppb (Bnp), natriuretic peptide type B; Opa3, optic atrophy 3; WGA, wheat germ agglutinin.

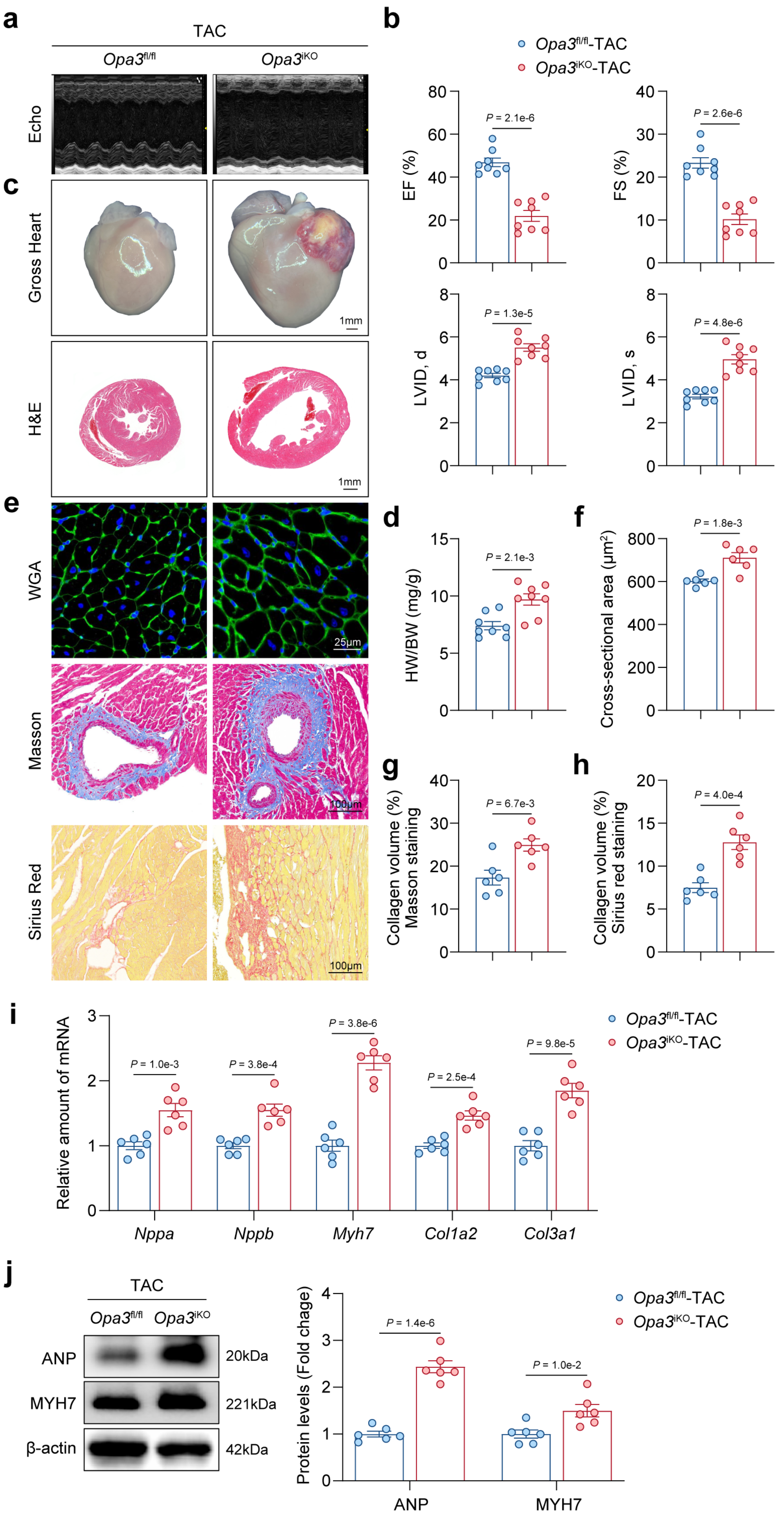
a
TAC
Opa3fl/fl
Opa3iKO
Echo
c
Gross Heart
H&E
1mm
1mm
e
WGA
Masson
Sirius Red
25μm
100μm
100μm
b
Opa3fl/fl-TAC
Opa3iKO-TAC
EF (%)
P = 2.1e-6
FS (%)
P = 2.6e-6
LVID, d
P = 1.3e-5
LVID, s
P = 4.8e-6
d
HW/BW (mg/g)
P = 2.1e-3
f
Cross-sectional area (μm²)
P = 1.8e-3
g
Collagen volume (%)
Masson staining
P = 6.7e-3
h
Collagen volume (%)
Sirius red staining
P = 4.0e-4
i
Relative amount of mRNA
P = 1.0e-3
P = 3.8e-4
P = 3.8e-6
P = 2.5e-4
P = 9.8e-5
Nppa
Nppb
Myh7
Col1a2
Col3a1
Opa3fl/fl-TAC
Opa3iKO-TAC
j
TAC
Opa3fl/fl
Opa3iKO
ANP
MYH7
β-actin
20kDa
221kDa
42kDa
Protein levels (Fold chage)
P = 1.4e-6
P = 1.0e-2
ANP
MYH7
Opa3fl/fl-TAC
Opa3iKO-TAC

Supplementary Fig. 7. Adult cardiomyocyte-specific deficiency of *Opa3* exacerbates TAC-induced HF.

a and **b**, Representative M-mode echocardiographic images (**a**) and corresponding measurements of EF, FS, LVID,d, and LVID,s (**b**) of *Opa3*$^{fl/fl}$ and *Opa3*^{iKO} mice at 8 weeks after TAC surgery (n=8 per group). **c**, **Top**, Representative gross images of hearts from *Opa3*$^{fl/fl}$ and *Opa3*^{iKO} mice at 8 weeks after TAC surgery (scale bar, 1 mm). **Bottom**, Representative images of H&E-stained heart sections in indicated groups (scale bar, 1 mm). **d**, Ratios of heart weight to body weight (HW/BW) in indicated groups (n=8 per group). **e**, Representative images of heart tissues stained with WGA, Masson trichrome, and Sirius red from *Opa3*$^{fl/fl}$ and *Opa3*^{iKO} mice at 8 weeks after TAC surgery (scale bar, 25 μm for WGA; 100 μm for Masson trichrome and Sirius red). **f**, Quantification of mean cardiomyocyte cross-section area of WGA-stained sections in (**e**) (n=6 per group). **g** and **h**, Quantification of fibrotic area (%) as determined by Masson trichome staining (**g**) (n=6 per group) and Sirius red staining of heart sections (**h**) presented in (**e**) (n=6 per group). **i**, Relative mRNA expression of *Nppa*, *Nppb*, *Myh7*, *Col1a2*, and *Col3a1* in the heart tissues from indicated groups (n=6 per group). **j**, **Left**, Representative western blot for ANP, MYH7, and β-actin in the heart tissues from indicated groups. **Right**, Quantification of protein expression in indicated groups shown in **Left** (n=6 per group). Data are expressed as mean ± SEM. Data in **b**, **d**, **f**, **g**, **h**, **i**, **j** were analyzed by two-sided Student's *t* test. Col1a2 indicates collagen type I alpha 2 chain; Col3a1, collagen type III alpha 1 chain; EF, ejection fraction; FS, fractional shortening; H&E, hematoxylin and eosin; HF, heart failure; LVID,d, internal dimension of the left ventricle in diastole; LVID,s, internal dimension of the left ventricle in systole; Myh7, myosin heavy chain 7; Nppa (Anp), natriuretic peptide type A; Nppb (Bnp), natriuretic peptide type B; Opa3, optic atrophy 3; TAC, transverse aortic constriction; WGA, wheat germ agglutinin.

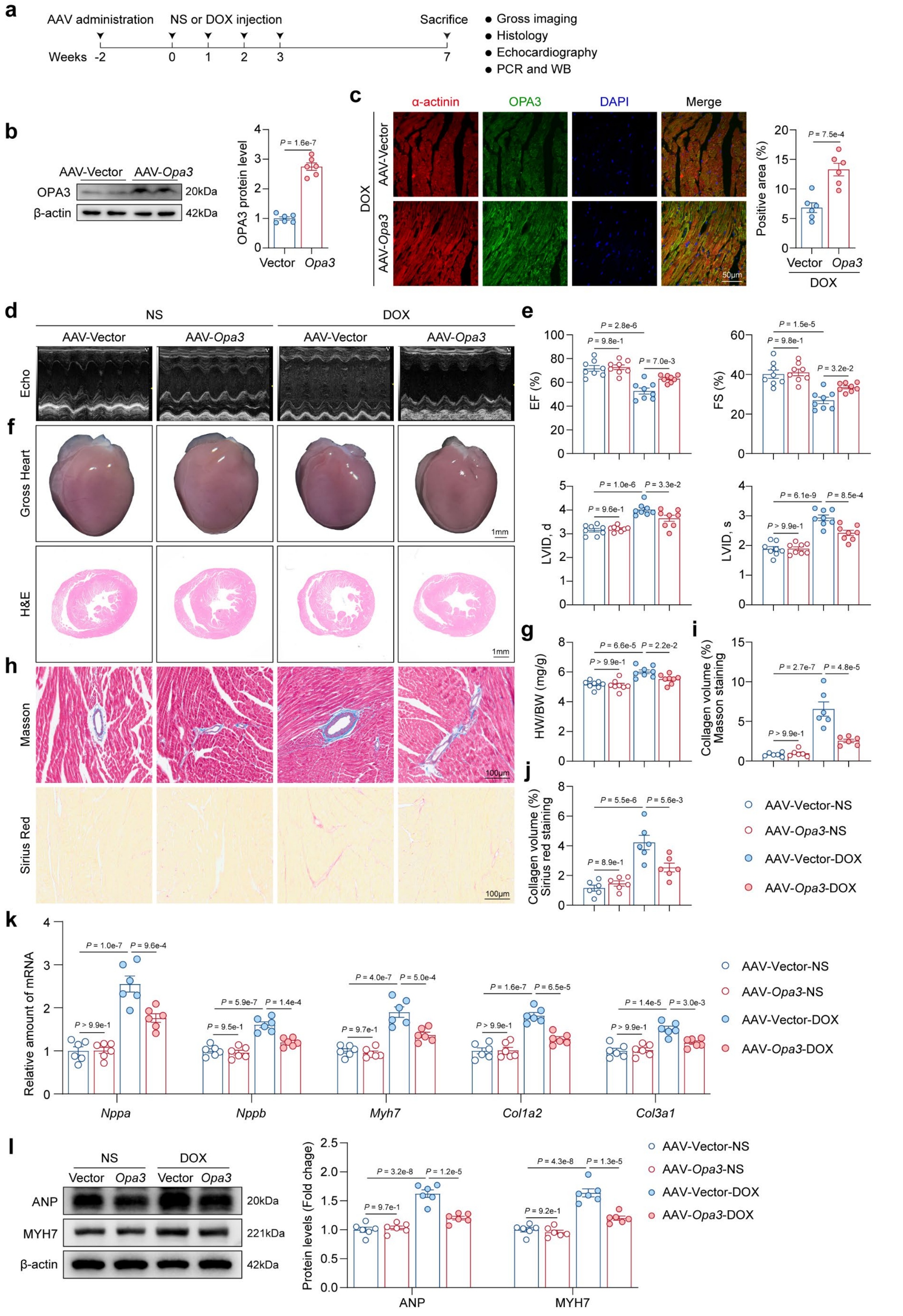

Supplementary Fig. 8. Cardiomyocyte-specific overexpression of Opa3 ameliorates DOX-induced cardiomyopathy.

a, Schematic diagram illustrating the timeline for AAV administration and DOX

induction. **b**, **Left**, Representative western blots and quantification of OPA3 in *Opa3*$^{fl/fl}$ mice treated with AAV-Vector or AAV-*Opa3* (n=6 per group). **c**, Colocalization of OPA3 (green) and α-actinin (red) in heart sections (n=6 per group, scale bars, 50 μm). Nuclei were counterstained with DAPI (blue). **d** and **e**, Representative M-mode echocardiographic images (**d**) and corresponding measurements of EF, FS, LVID,d, and LVID,s (**e**) in indicated groups (n=8 per group). **f**, Representative gross images of hearts and H&E-stained heart sections from indicated groups (scale bar, 1 mm). **g**, Ratios of heart weight to body weight (HW/BW) in indicated groups (n=8 per group). **h**, Representative images of heart tissues stained with Masson trichrome and Sirius red from indicated groups (scale bar, 100 μm). **i** and **j**, Quantification of fibrotic area (%) as determined by Masson trichome staining (**i**) and Sirius red staining (**j**) of heart sections presented in (**G**) (n=6 per group). **k**, Relative gene expression in indicated groups (n=6 per group). **l**, Representative western blots and quantification of ANP and MYH7 in the heart tissues from indicated groups (n=6 per group). Data are expressed as mean ± SEM. Data in **b**, **c** were analyzed by two-sided Student's *t* test. Data **e**, **g**, **i**, **j**, **k**, **l** were analyzed by one-way ANOVA followed by the Tukey post hoc test. AAV indicates adeno-associated viruses; Col1a2, collagen type I alpha 2 chain; Col3a1, collagen type III alpha 1 chain; DOX, doxorubicin; EF, ejection fraction; FS, fractional shortening; H&E, hematoxylin and eosin; LVID,d, internal dimension of the left ventricle in diastole; LVID,s, internal dimension of the left ventricle in systole; Myh7, myosin heavy chain 7; Nppa (Anp), natriuretic peptide type A; Nppb (Bnp), natriuretic peptide type B; Opa3, optic atrophy 3.

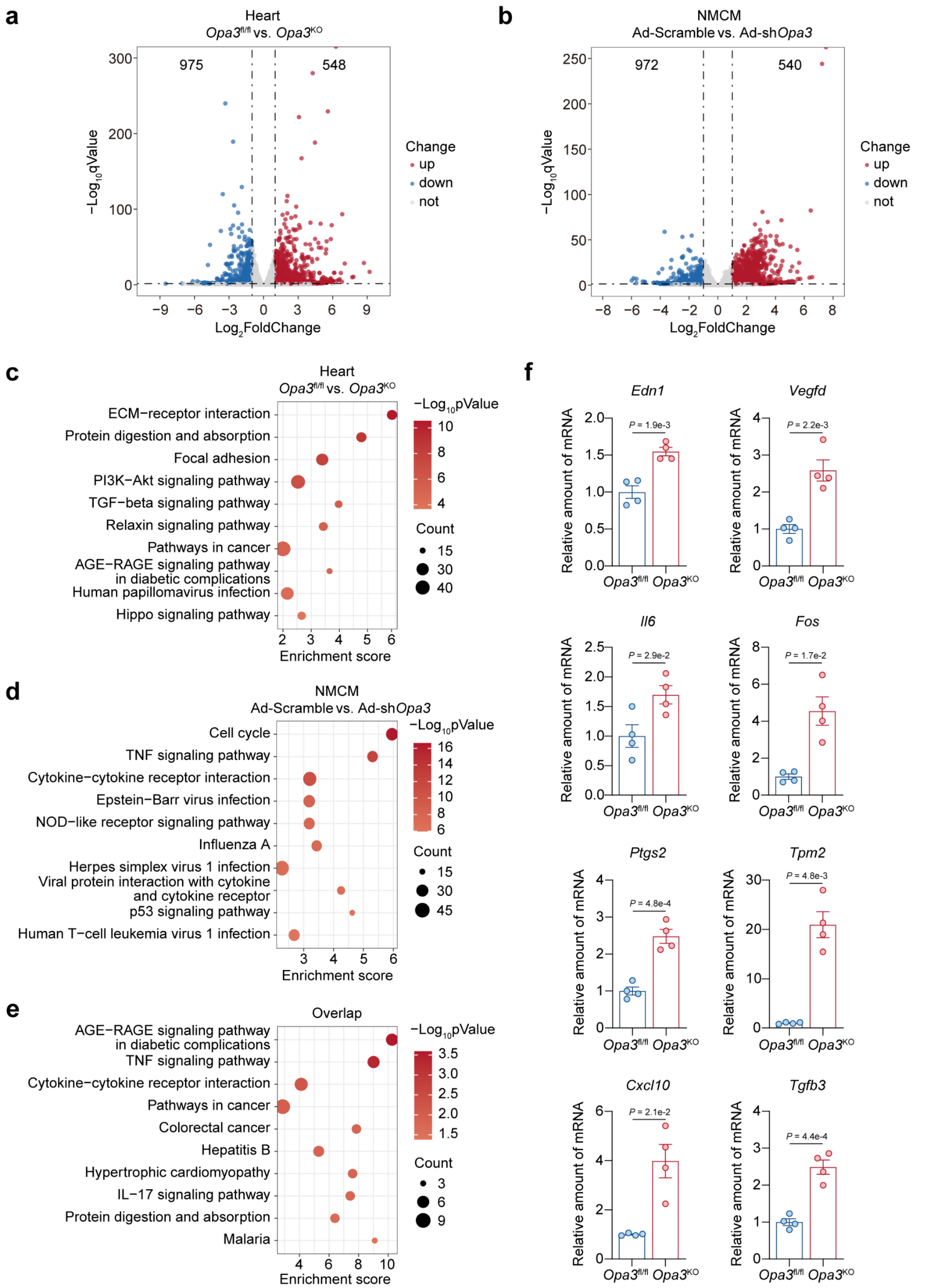

Supplementary Fig. 9. Cardiomyocytes-derived *Opa3* regulates pathways related to inflammatory response and cardiomyopathy.

a, Volcano plot revealing the magnitude and significance of altered genes in heart tissues from *Opa3*$^{fl/fl}$ vs. *Opa3*^{KO} groups at 4 weeks of age (n=4 per group). DEGs were

defined as genes with a Benjamini-Hochberg adjusted P <0.05 and $|\log_2$(fold change)$|\geq 1$. **b**, Volcano plot reveals the magnitude and significance of altered genes in NMCMs infected with adenovirus encoding scramble (Ad-Scramble) or shRNA targeting *Opa3* (Ad-sh*Opa3*) for 48 h (n=5 per group). DEGs were defined as genes with a Benjamini-Hochberg adjusted P <0.05 and $|\log_2$(fold change)$|\geq 1$. **c**, The top 10 enriched KEGG pathways of upregulated DEGs in heart tissues from $Opa3^{KO}$ mice compared with $Opa3^{fl/fl}$ controls. **d**, The top 10 enriched KEGG pathways of upregulated DEGs in NMCMs infected with Ad-sh*Opa3* compared with Ad-Scramble controls. **e**, The top 10 enriched KEGG pathways of upregulated overlap DEGs shared by both heart and NMCM. **f**, Relative mRNA expression of genes related to inflammatory response and cardiomyopathy in heart tissues from $Opa3^{fl/fl}$ vs. $Opa3^{KO}$ mice (n=4 per group). Data are expressed as mean ± SEM. Data in **c**, **d**, **e** were analyzed by one-sided hypergeometric test. Data in **f** were analyzed by two-sided Student's t test (equal variances) or Welch's t test (unequal variances). DEG indicates differentially expressed genes; KEGG, Kyoto Encyclopedia Genes and Genomes; NMCM, neonatal mouse cardiomyocyte; Opa3, optic atrophy 3.

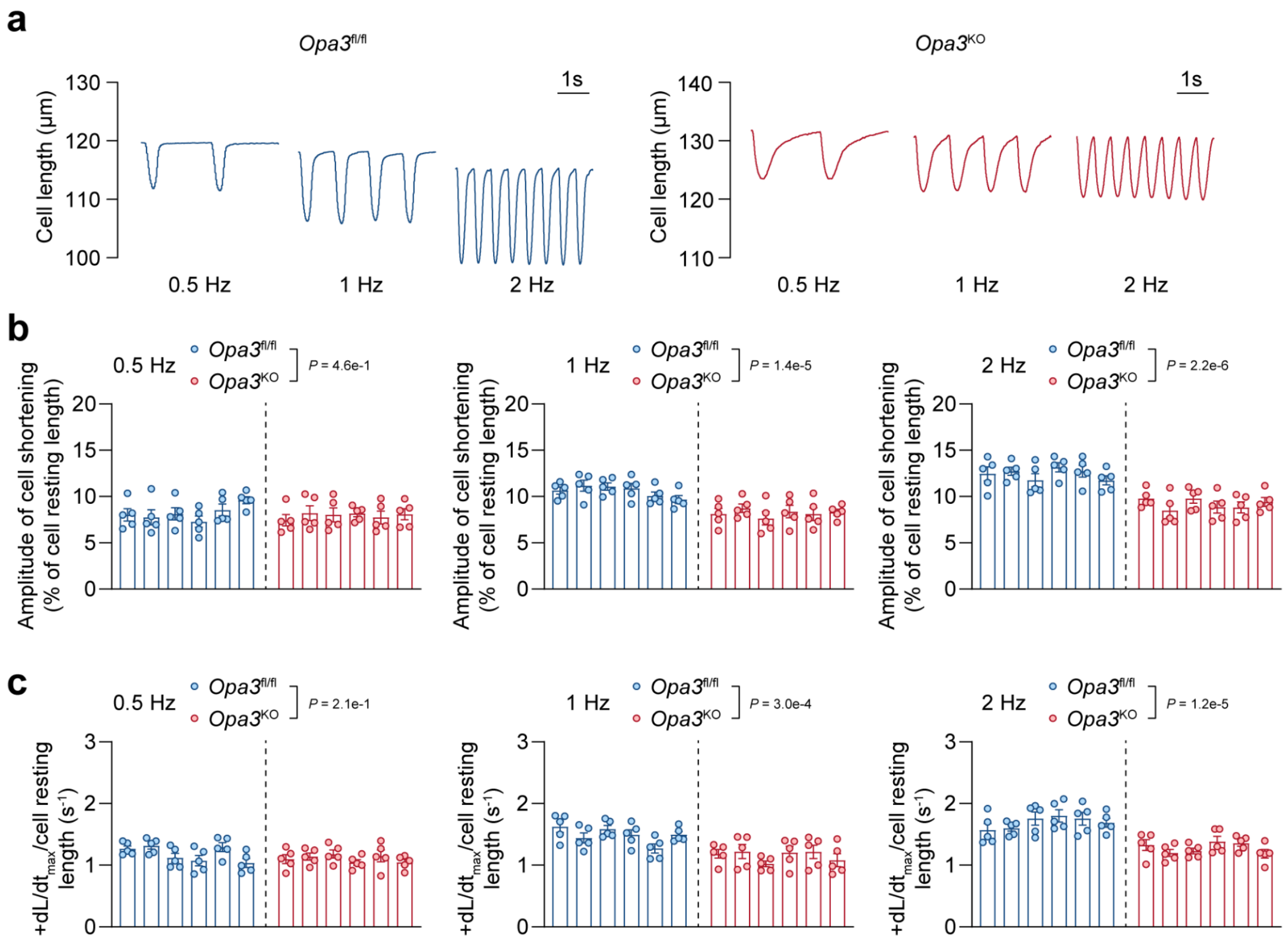

Supplementary Fig. 10. The absence of *Opa3* in cardiomyocytes exhibited an abnormal contraction phenotype.

a–**c**, AMCMs were incubated with a Fura-2 AM and cell shortening were recorded under continuous 0.5 Hz, 1 Hz, and 2 Hz electrical stimulation. **a**, Representative traces of cell shortening in isolated AMCMs from *Opa3*$^{fl/fl}$ and *Opa3*^{KO} mice at 8 weeks of age. **b** and **c**, Quantification of the amplitude of cell shortening (**b**) and the maximum upstroke velocity of cell shortening (**c**) shown in (**a**) (5 cardiomyocytes per animal, 6 animals per group). Data are expressed as mean ± SEM. Data in **b**, **c** were analyzed by two-sided nested *t* test. AMCM indicates adult mouse cardiomyocyte; Opa3, optic atrophy 3.

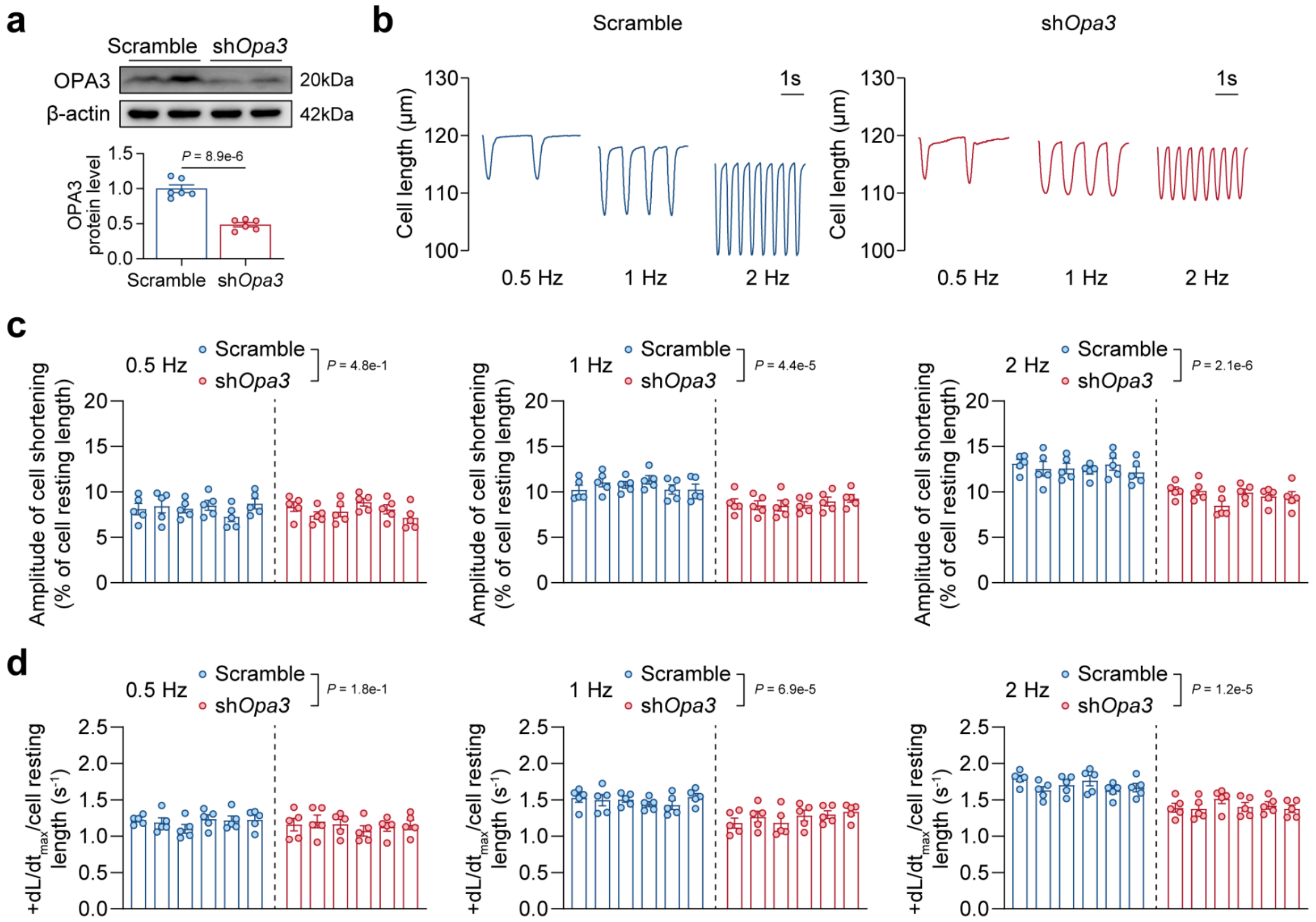

Supplementary Fig. 11. The knockdown of *Opa3* in cardiomyocytes exhibited an abnormal contraction phenotype.

a, Representative western blots (**Top**) and quantification (**Bottom**) of OPA3 and β-actin from isolated AMCMs. AMCMs were infected with adenovirus encoding scramble (Scramble) or shRNA targeting *Opa3* (sh*Opa3*) for 48 h (n=6 per group). **b–d**, AMCMs were incubated with a Fura-2 AM and cell shortening were recorded under continuous 0.5 Hz, 1 Hz, and 2 Hz electrical stimulation. **b**, Representative traces of cell shortening in isolated AMCMs infected with adenovirus encoding scramble (Scramble) or shRNA targeting *Opa3* (sh*Opa3*) for 48 h. **c** and **d**, Quantification of the amplitude of cell shortening (**c**) and the maximum upstroke velocity of cell shortening (**d**) shown in (**b**) (5 cardiomyocytes per animal, 6 animals per group). Data are expressed as mean ± SEM. Data in **a** were analyzed by two-sided Student's *t* test. Data in **c**, **d** were analyzed by two-sided nested *t* test. AMCM indicates adult mouse cardiomyocyte; Opa3, optic atrophy 3.

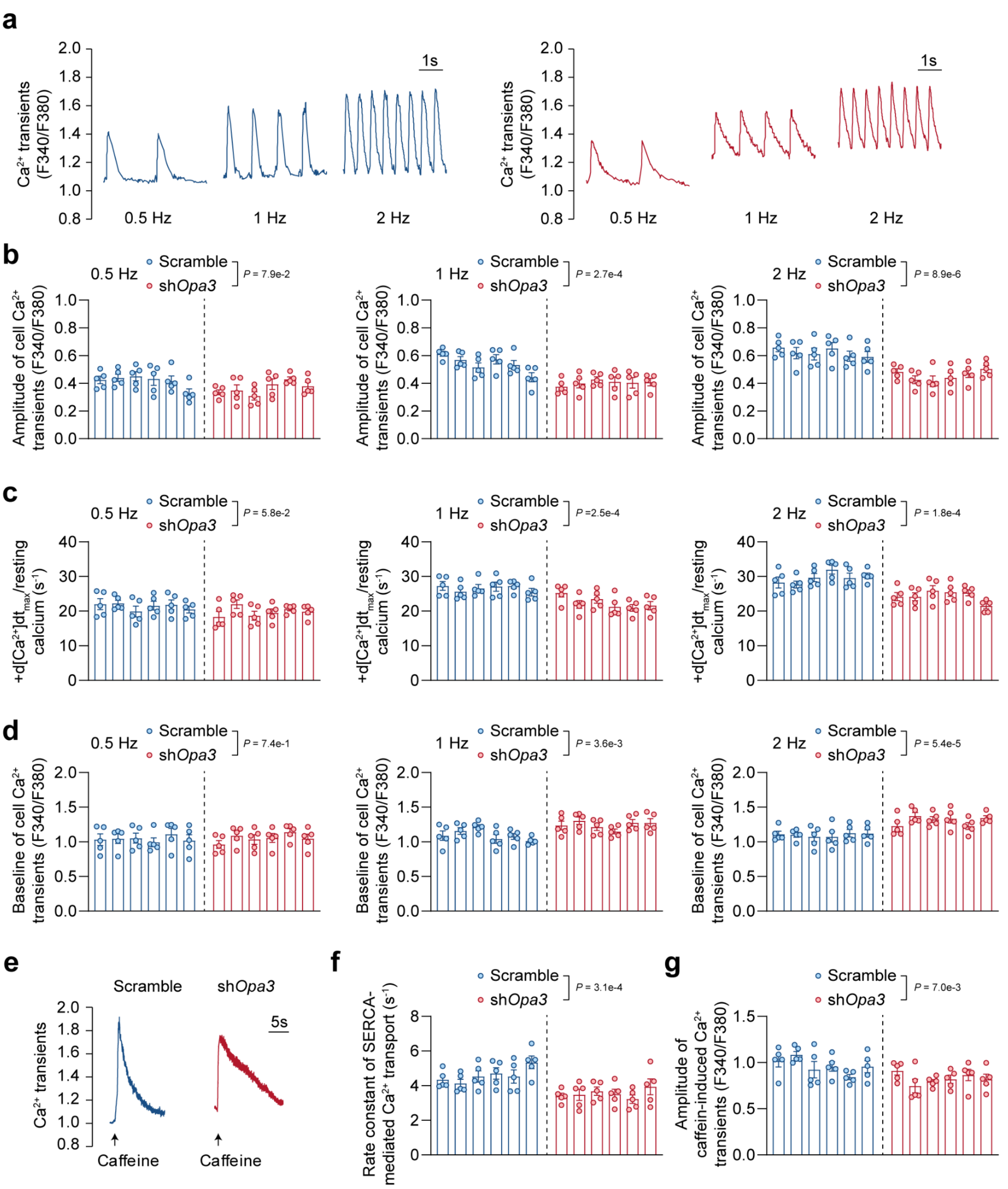

Supplementary Fig. 12. The knockdown of *Opa3* in cardiomyocytes leads to Ca^{2+} handling deficiency.

a–**d**, AMCMs were incubated with a Fura-2 AM and Ca^{2+} transients were recorded under continuous 0.5 Hz, 1 Hz, and 2 Hz electrical stimulation. **a**, Representative traces of calcium transients in isolated AMCMs infected with adenovirus encoding scramble (Scramble) or shRNA targeting *Opa3* (sh*Opa3*) for 48 h. **b**–**d**, Quantification of amplitude of Ca^{2+} transients (**b**), maximal ascending rate in cell contractile Ca^{2+} transients (**c**) and baseline of Ca^{2+} transients (**d**) shown in (**a**) (5 cardiomyocytes per animal, 6 animals per group). **e**, Representative traces of Ca^{2+} transients during caffeine

(10 mM)-induced contraction in NT solution in indicated groups. **f** and **g**, Quantification of the rate constant of SERCA-mediated Ca^{2+} transport (**f**) and the amplitude of caffeine-induced Ca^{2+} transients (SR Ca^{2+} content, **g**) shown in (**e**) (5 cardiomyocytes per animal, 6 animals per group). Data are expressed as mean ± SEM. Data in **b**, **c**, **d**, **f**, **g** were analyzed by two-sided nested *t* test. AMCM indicates adult mouse cardiomyocyte; NT, normal Na^{+}/Ca^{2+} Tyrode's; Opa3, optic atrophy 3; SERCA, sarcoplasmic/endoplasmic reticulum Ca^{2+}-ATPase; SR, sarcoplasmic reticulum.

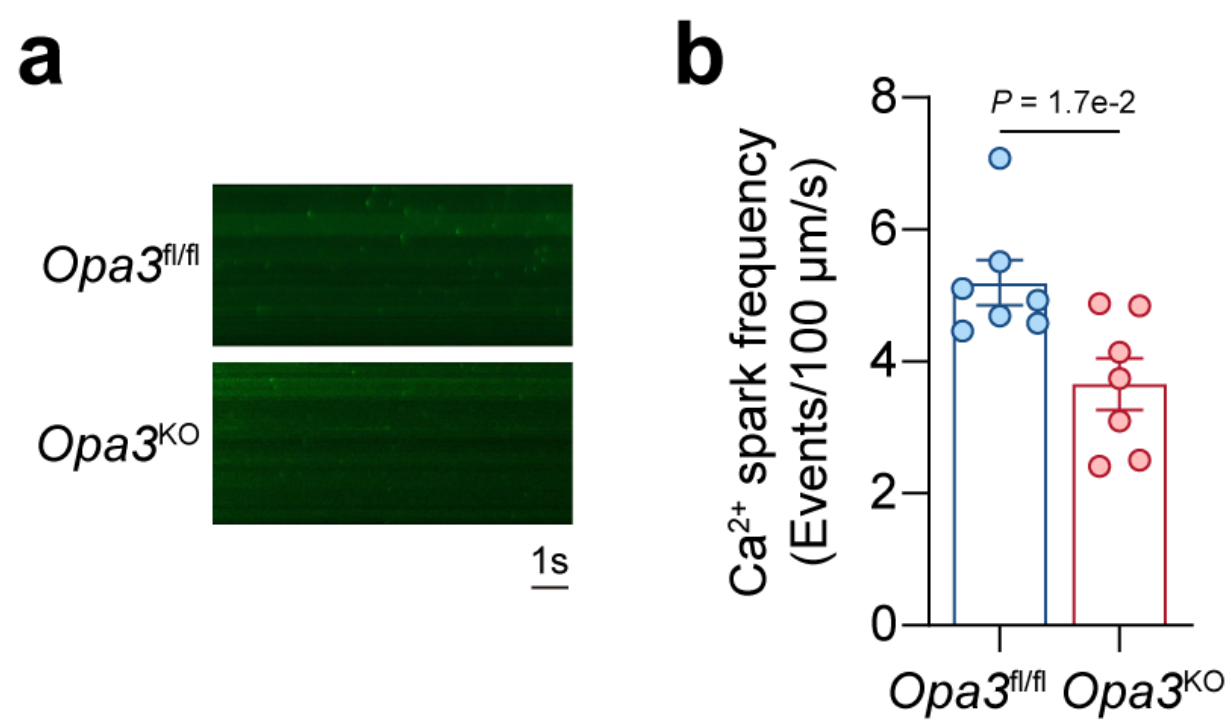

Supplementary Fig. 13. Deficiency of *Opa3* decreases SR Ca^{2+} leak.

a, Line scan images show Ca^{2+} sparks of indicated groups after 2 Hz stimulation. **b**, Quantification of Ca^{2+} sparks frequency in (**a**). Data were analyzed by two-sided Mann-Whitney *U* test. Data are expressed as mean ± SEM. Opa3 indicates optic atrophy 3; SR, sarcoplasmic reticulum.

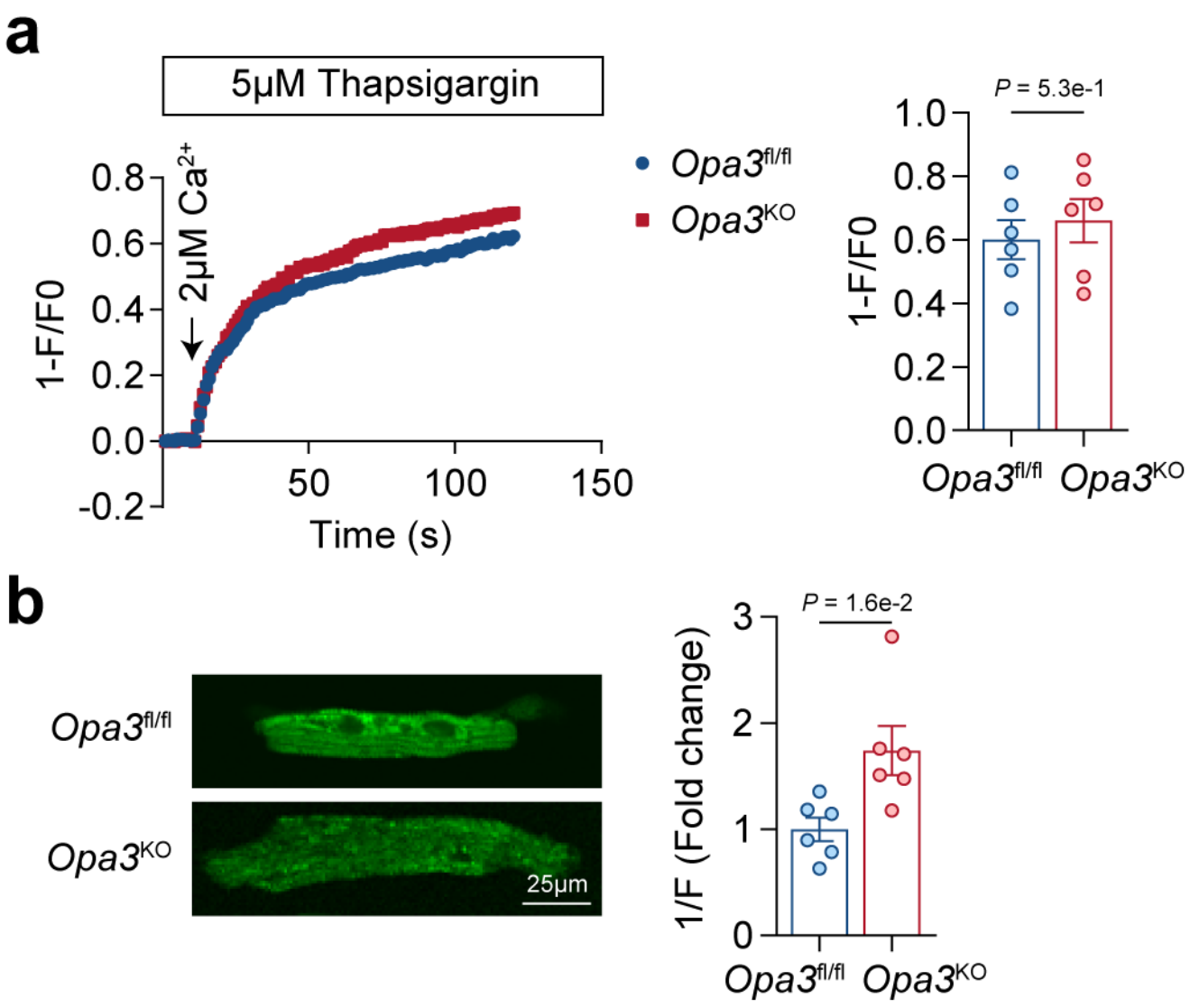

Supplementary Fig. 14. Mitochondrial Ca^{2+} uptake in cardiomyocytes.

a, **Left**, Representative traces of normalized Mitycam signal in isolated AMCMs from *Opa3*$^{fl/fl}$ and *Opa3*^{KO} mice. **Right**, Quantification of the amplitude of fluorescence in response to changes of Ca^{2+}. **b,** Representative image (**Left**) and quantification (**Right**) of Mitycam in resting isolated AMCMs. Data are expressed as mean ± SEM. Data in **a**, **b** were analyzed by two-sided Student's *t* test. AMCM indicates adult mouse cardiomyocyte.

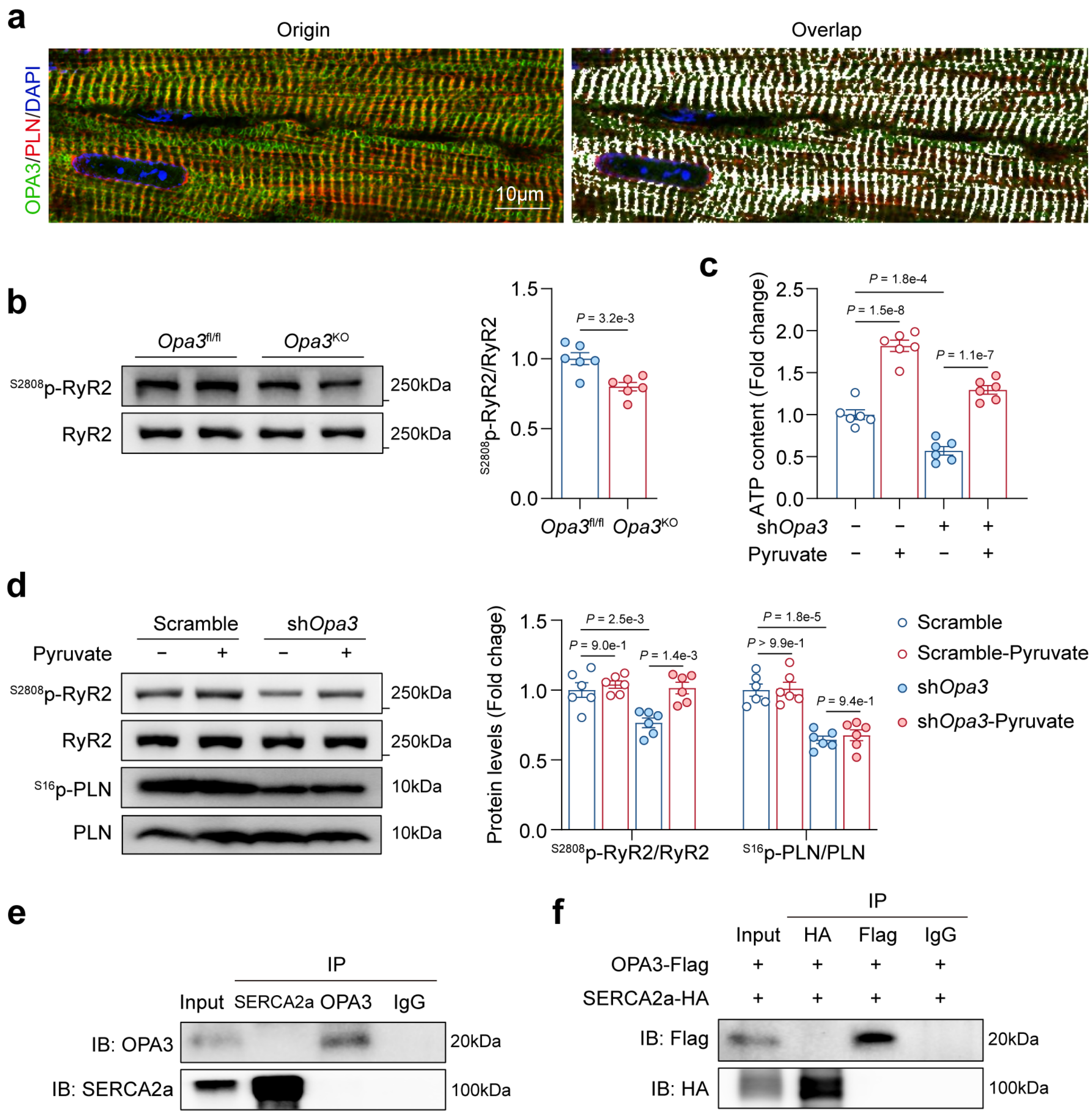

Supplementary Fig. 15. The role of OPA3 in PKA signaling pathway.

a, Representative images of the colocalization of OPA3 (green) and PLN (red) in heart sections (scale bars, 10 μm). Nuclei were counterstained with DAPI (blue). The upper panels show the original fluorescence images, and the lower panels display the colocalized OPA3-PLN regions (white) identified using *FIJI* software. Colocalized OPA3-PLN signals were quantified and normalized to total OPA3 fluorescence intensity to estimate the fraction of OPA3 associated with PLN *in situ*. **b**, Representative western blots and quantification of RyR2 and its phosphorylation in PE-treated (50 nM for 48 h) AMCMs isolated from *Opa3*$^{\mathrm{fl/fl}}$ and *Opa3*^{KO} mice (n=6 per group). **c**, ATP content was determined by ATP Assay Kit (n=6 per group). **d**, Representative western blots in indicated groups. NMCMs were infected with adenovirus encoding scramble (Scramble) or shRNA targeting *Opa3* (sh*Opa3*) for 48 h. NMCMs were then

supplemented with pyruvate (20 mM) for 30 min and treated with db-cAMP (10 μM). **e**, Coimmunoprecipitation of OPA3 and SERCA2a in heart tissues. Endogenous OPA3 or SERCA2a was immunoprecipitated by anti-OPA3 antibody or anti-SERCA2a antibody, respectively, using IgG as a negative control. **f**, Coimmunoprecipitation of OPA3 and SERCA2a in HEK293T cells co-transfected with OPA3-Flag and SERCA2a-HA plasmids. Exogenous OPA3-Flag or SERCA2a-HA was immunoprecipitated by anti-Flag antibody or anti-HA antibody, respectively, using IgG as a negative control. Data are expressed as mean ± SEM. Data in **b** were analyzed by two-sided Student's *t* test. Data in **c**, **d** were analyzed by one-way ANOVA followed by the Tukey post hoc test. AMCMs indicates adult mouse cardiomyocytes; NMCMs, neonatal mouse cardiomyocytes; OPA3, optic atrophy 3; PKA, protein kinase A; PLN, phospholamban; RyR2, ryanodine receptor 2; SERCA, sarcoplasmic/endoplasmic reticulum Ca^{2+}-ATPase.

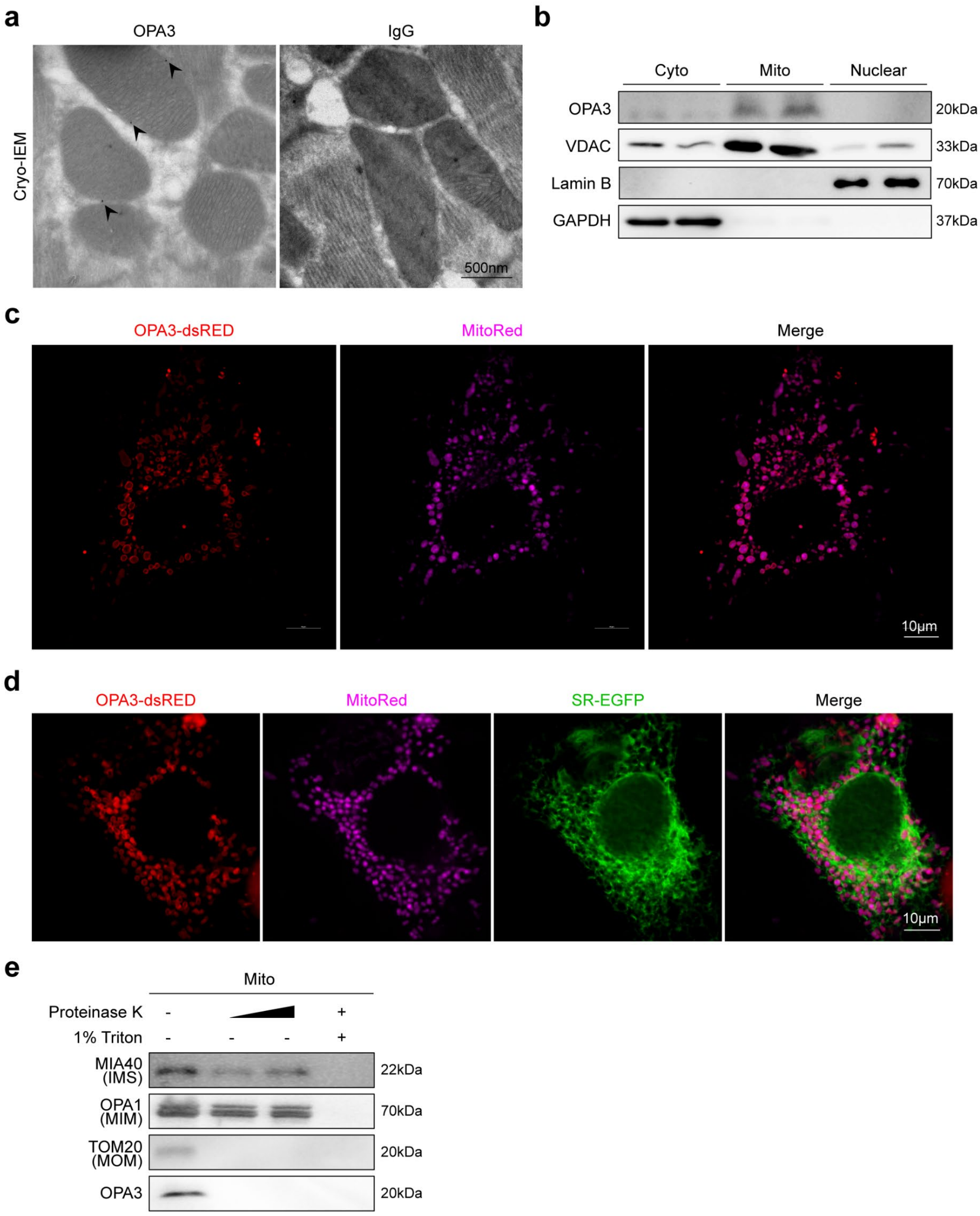

Supplementary Fig. 16. OPA3 localizes to the outer mitochondrial membrane.

a, Cryo-immunogold electron microscopy (Cryo-IEM) of mouse heart tissues was performed using 12-nm gold particles. Arrows indicate OPA3 labeling. **b**, Representative western blot for OPA3 in cytosolic (Cyto), mitochondrial (Mito), and nuclear (Nuclear) fractions from heart tissues. GAPDH, VDAC, and Lamin B were used as markers for cytosolic, mitochondrial, and nuclear loading controls, respectively. **c**, Representative images of MitoRed staining in H9C2 cells infected with adenovirus

encoding OPA3-dsRED fusion protein for 48 h (scale bar, 10 μm). **d**, Representative images of MitoRed staining in H9C2 cells infected with adenovirus encoding OPA3-dsRED fusion protein and transfected with and SR-EGFP probe mRNA for 24 h (scale bar, 10 μm). **e**, Representative western blot for proteinase K protection assay. Specifically, isolated mitochondria were treated with 10 μg/ml proteinase K for 30 min in the absence or presence of 1% Triton X-100. GAPDH indicates glyceraldehyde-3-phosphate dehydrogenase; OPA3, optic atrophy 3; SR, sarcoplasmic reticulum; VDAC, voltage-dependent anion channel.

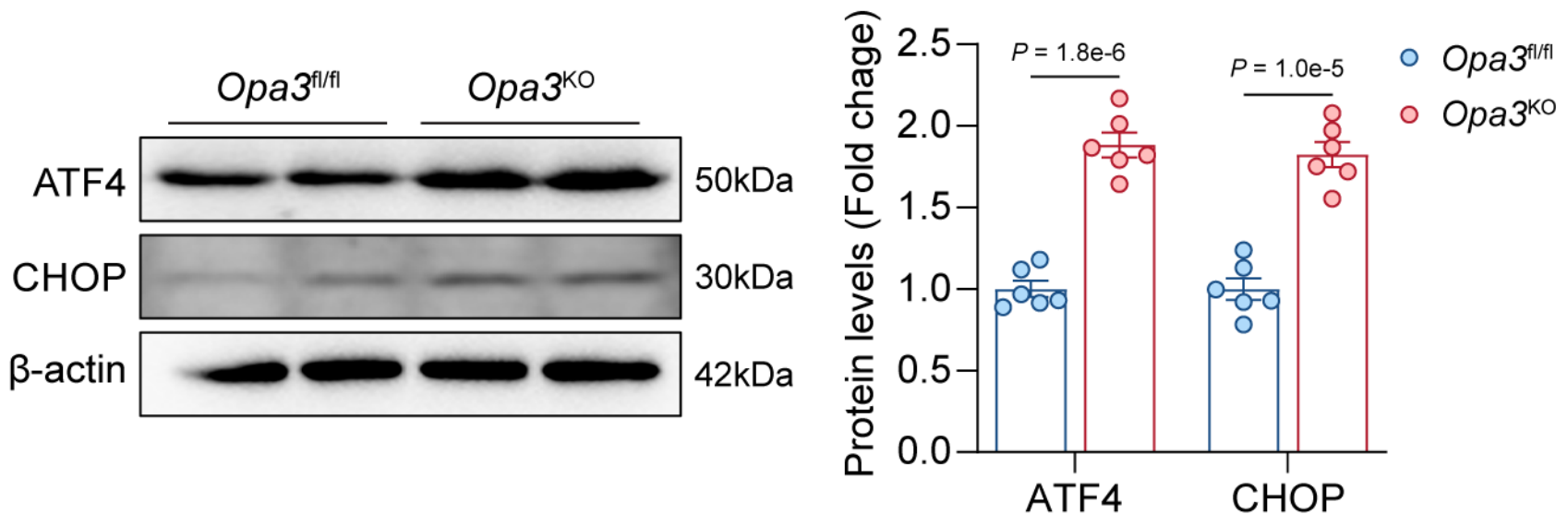

Supplementary Fig. 17. The role of OPA3 in SR stress.

Left, Representative western blots for SR stress-related proteins. **Right**, Quantification of protein expression in indicated groups shown in **Left** (n=6 per group). Data were analyzed by two-sided Student's *t* test. Data are expressed as mean ± SEM. OPA3 indicates optic atrophy 3; SR, sarcoplasmic reticulum.

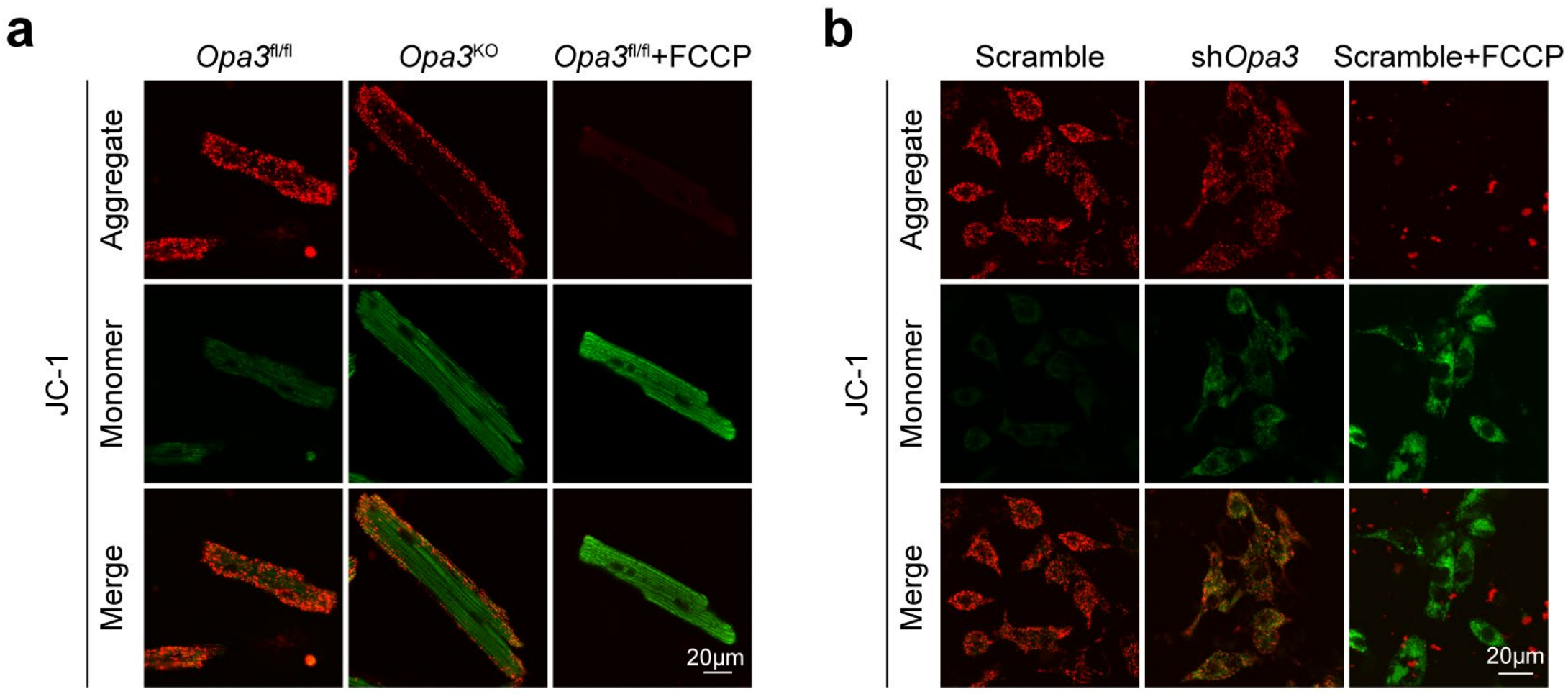

Supplementary Fig. 18. ***Opa3*** **deficiency impairs mitochondrial function.**

a, Representative images of mitochondrial ΔΨm assessed by JC-1 staining in isolated AMCMs from *Opa3*$^{fl/fl}$ and *Opa3*^{KO} mice (scale bar, 20 μm). FCCP was used as a positive control. **b**, Representative images of mitochondrial ΔΨm assessed by JC-1 staining in isolated NMCMs (scale bar, 20 μm). Specifically, the NMCMs were infected with adenovirus encoding scramble (Scramble) or shRNA targeting *Opa3* (sh*Opa3*) for 48 h. FCCP was used as a positive control. AMCM indicates adult mouse cardiomyocyte; FCCP, carbonyl cyanide 4-(trifluoromethoxy) phenylhydrazone; NMCM, neonatal mouse cardiomyocyte; OPA3, optic atrophy 3.

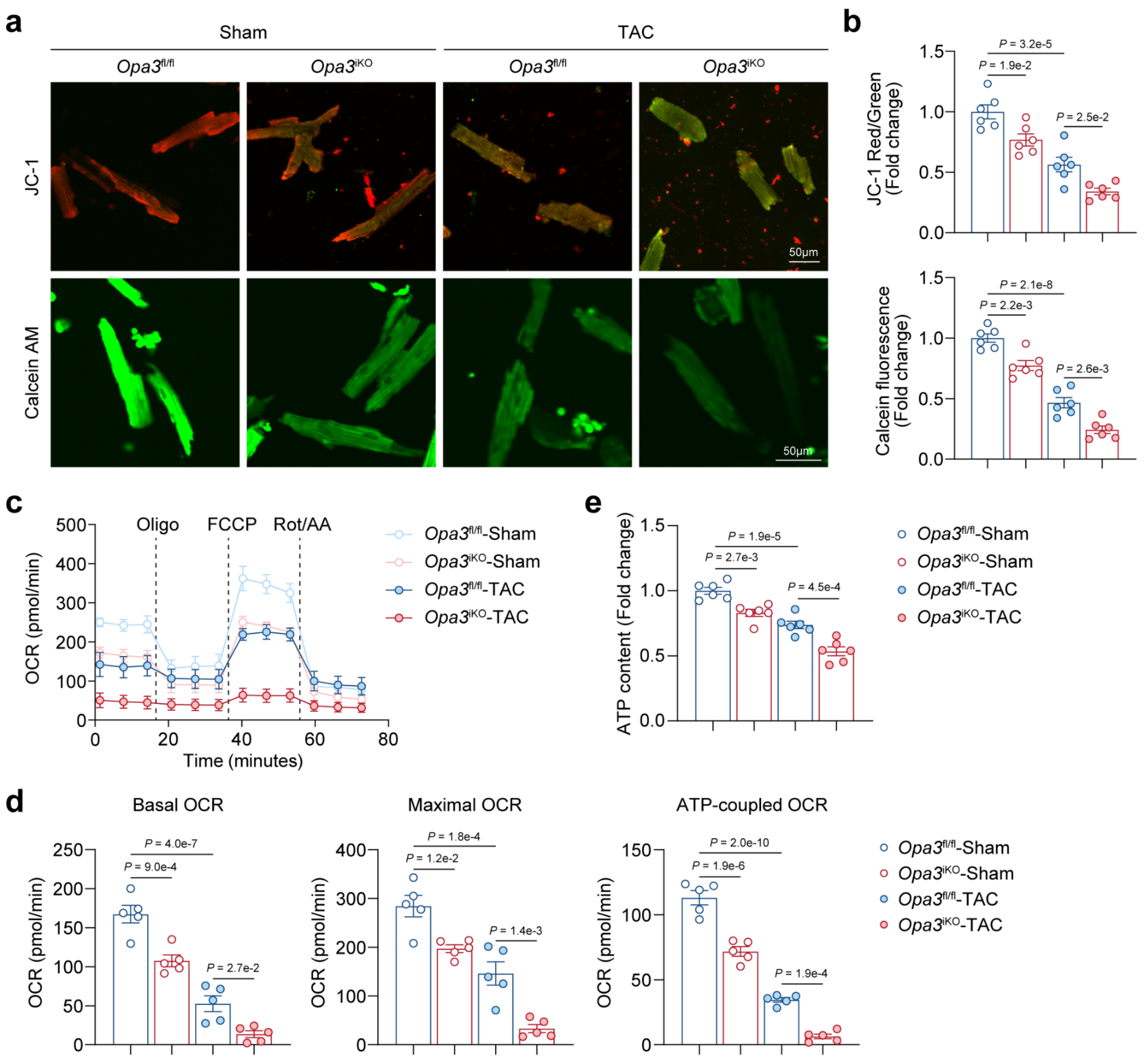

Supplementary Fig. 19. Adult cardiomyocyte-specific deficiency of *Opa3* exacerbates TAC-induced mitochondrial dysfunction of cardiomyocytes.

a, Representative images of mitochondrial ΔΨm assessed by JC-1 staining and mPTP opening by calcein AM/$CoCl_2$ staining in isolated AMCMs from *Opa3*$^{fl/fl}$ and *Opa3*^{iKO} mice at 8 weeks after sham or TAC surgery (scale bar, 50 μm). **b**, Quantification of JC-1 staining and calcein AM/$CoCl_2$ staining in indicated groups (n=6 per group). **c** and **d**, Analysis (**c**) and quantification (**d**) of mitochondrial OCR in isolated AMCMs from *Opa3*$^{fl/fl}$ and *Opa3*^{iKO} mice at 8 weeks after sham or TAC surgery (n=5 per group). The dash lines indicate the sequential addition of oligomycin (Oligo), FCCP, and Rot/AA. **e**, ATP content of isolated AMCMs from *Opa3*$^{fl/fl}$ and *Opa3*^{iKO} mice at 8 weeks after sham or TAC surgery was determined by ATP Assay Kit (n=6 per group). Data are expressed as mean ± SEM. Data in **b**, **d**, **e** were analyzed by one-way ANOVA followed by the Tukey post hoc test. AA indicates antimycin A; AMCM, adult mouse cardiomyocyte; ATP, adenosine triphosphate; FCCP, carbonyl cyanide 4-

(trifluoromethoxy) phenylhydrazone; mPTP, mitochondrial permeability transition pore; OCR, oxygen consumption rate; Opa3, optic atrophy 3; Rot, rotenone; TAC, transverse aortic constriction.

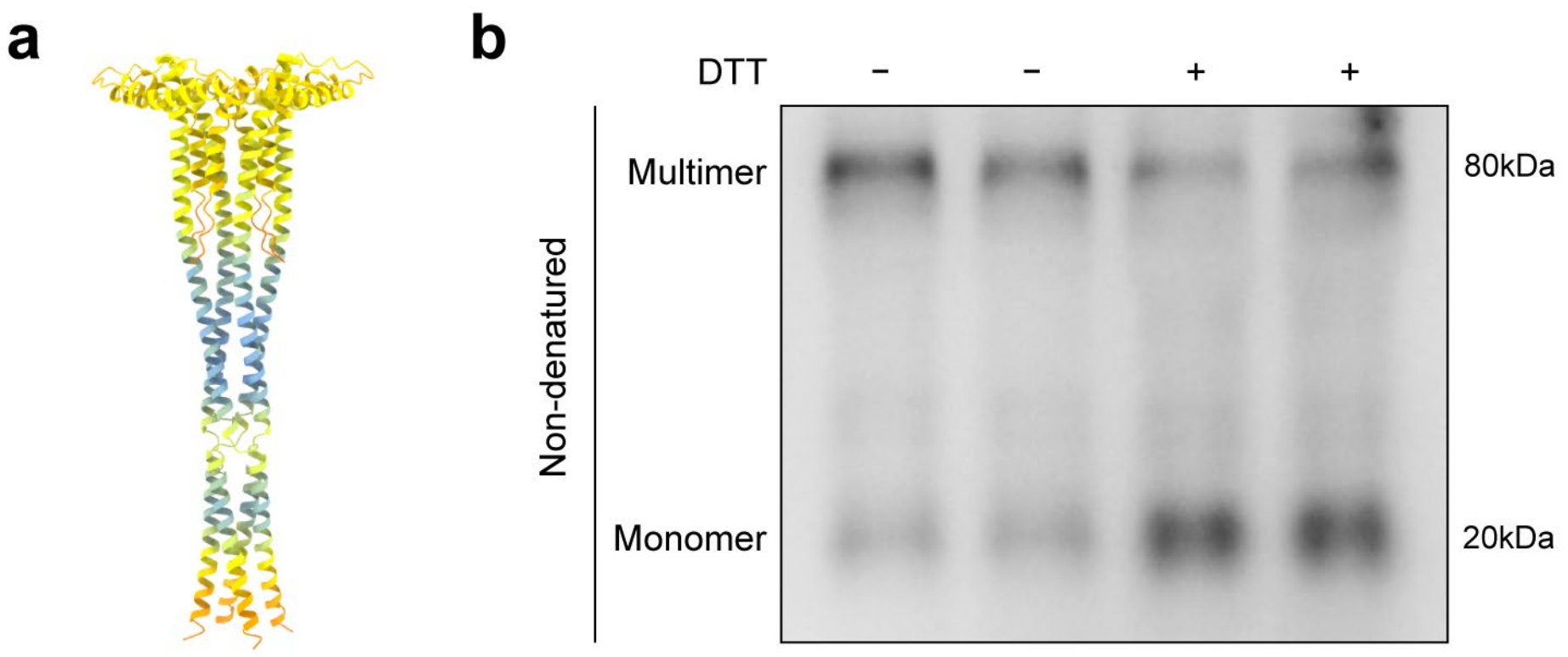

Supplementary Fig. 20. The multimerization of OPA3.

a, Structure of OPA3 as predicted by AlphaFold 2. The colors correspond to model per-residue confidence (pLDDT): light blue, confident (90 > pLDDT > 70), yellow, low (70 > pLDDT > 50), orange, very low (pLDDT < 50). **b**, Representative western blots of OPA3 monomer and multimer in native protein extraction from NMCMs. Protein extracts were analyzed in the presence or absence of DTT. DTT indicates dithiothreitol; NMCM, neonatal mouse cardiomyocyte; OPA3, optic atrophy 3.

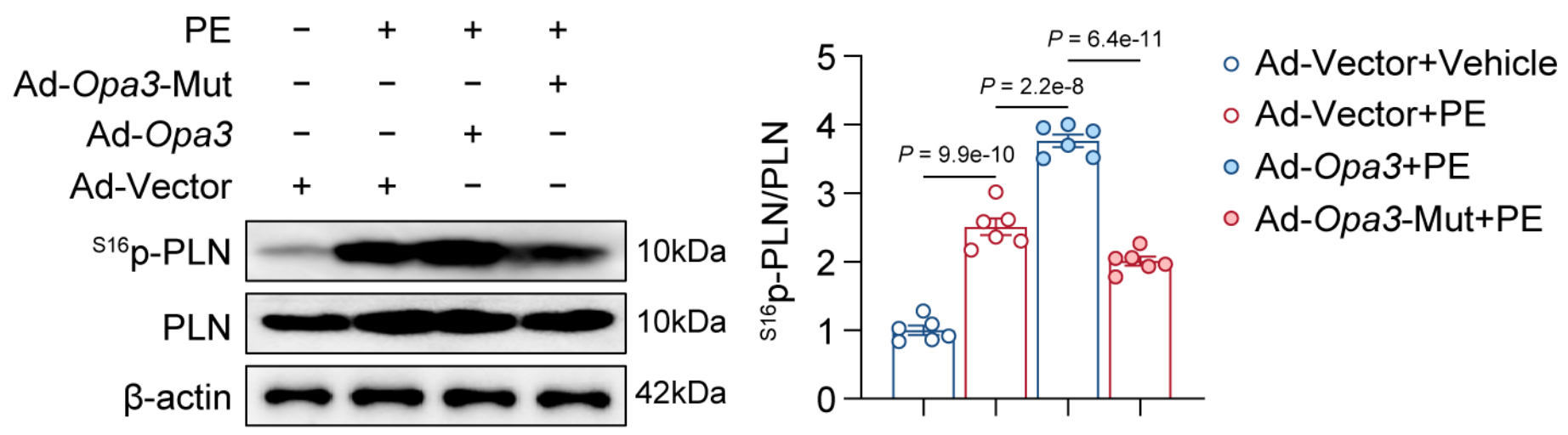

Supplementary Fig. 21. The role OPA3 multimerization in PE-induced PLN phosphorylation.

Representative western blots and quantification for PLN and its phosphorylation levels in indicated groups. NMCMs were infected with adenoviruses encoding wild-type *Opa3* or its mutation for 48 h and treated with 50 nM PE for 1 h (n=6 per group). Data were analyzed by one-way ANOVA followed by the Tukey post hoc test. Data are expressed as mean ± SEM. NMCM indicates neonatal mouse cardiomyocyte; OPA3, optic atrophy 3; PE, phenylephrine; PLN, phospholamban.

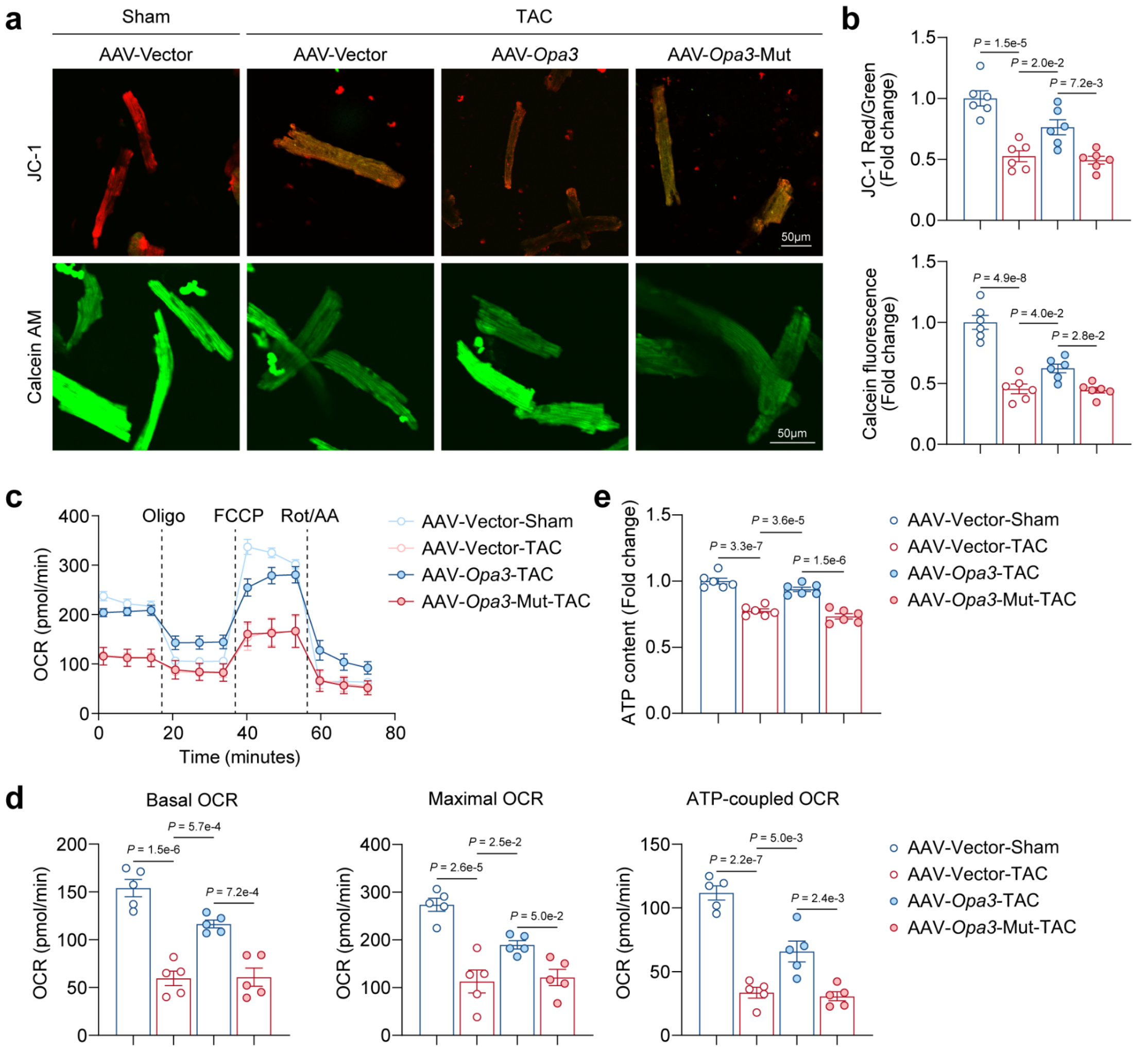

Supplementary Fig. 22. OPA3 multimerization is required for its protective effect against TAC-induced mitochondrial dysfunction of cardiomyocytes.

a, Representative images of mitochondrial ΔΨm assessed by JC-1 staining and mPTP opening by calcein AM/$CoCl_2$ staining in isolated AMCMs from mice received AAV-Vector, AAV-*Opa3*, and AAV-*Opa3*-Mut at 8 weeks after sham or TAC surgery (scale bar, 50 μm). **b**, Quantification of JC-1 staining and calcein AM/$CoCl_2$ staining in indicated groups (n=6 per group). **c** and **d**, Analysis (**c**) and quantification (**d**) of mitochondrial OCR in isolated AMCMs from indicated groups (n=5 per group). The dash lines indicate the sequential addition of oligomycin (Oligo), FCCP, and Rot/AA. **e**, ATP content of isolated AMCMs from indicated groups was determined by ATP Assay Kit (n=6 per group). Data are expressed as mean ± SEM. Data **b**, **d**, **e** were analyzed by one-way ANOVA followed by the Tukey post hoc test. AA indicates antimycin A; AAV, adeno-associated viruses; AMCM, adult mouse cardiomyocyte; ATP, adenosine triphosphate; FCCP, carbonyl cyanide 4-(trifluoromethoxy)

phenylhydrazone; mPTP, mitochondrial permeability transition pore; OCR, oxygen consumption rate; OPA3, optic atrophy 3; Rot, rotenone; TAC, transverse aortic constriction.

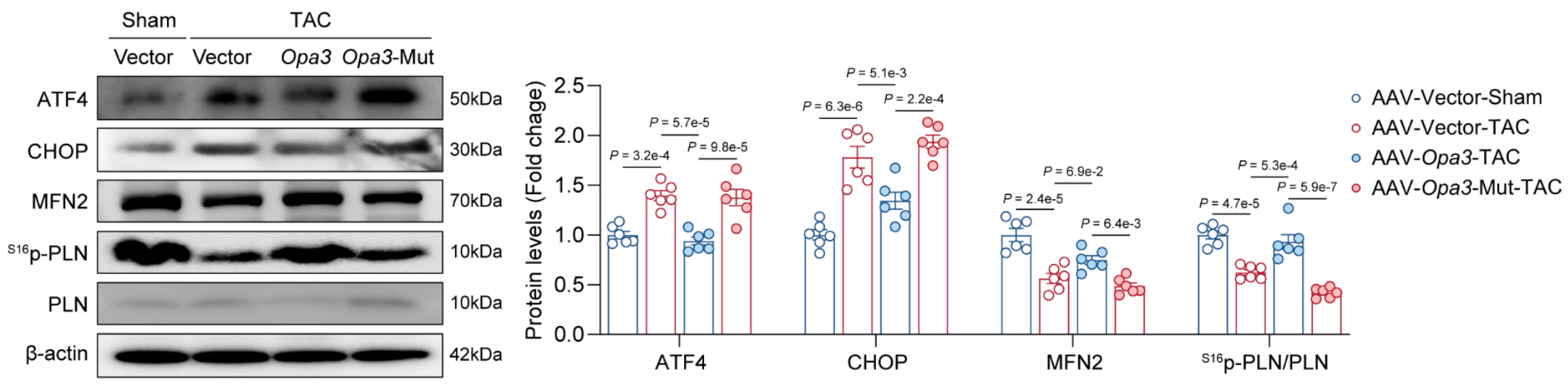

Supplementary Fig. 23. OPA3 multimerization is required for its protective effect against TAC-induced SR dysfunction of cardiomyocytes.

Representative western blots and quantification for SR stress-related proteins in indicated groups (n=6 per group). Data were analyzed by one-way ANOVA followed by the Tukey post hoc test. Data are expressed as mean ± SEM. OPA3 indicates optic atrophy 3; SR, sarcoplasmic reticulum; TAC, transverse aortic constriction.

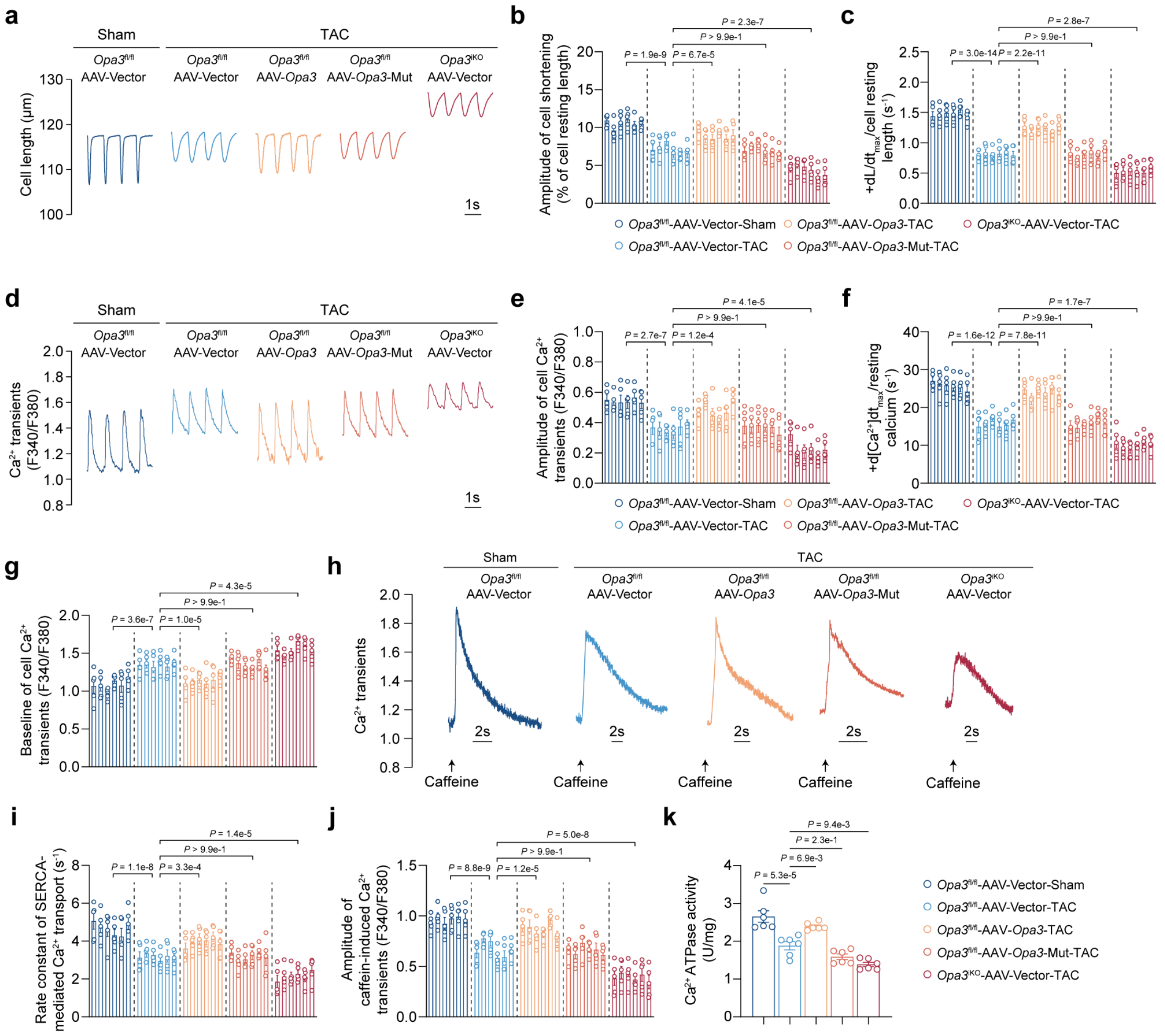

Supplementary Fig. 24. OPA3 multimerization is required for its protective effect against TAC-induced Ca^{2+} handling deficiency.

a–**g**, AMCMs were incubated with a Fura-2 AM; then, cell shortening and Ca^{2+} transients were recorded under 1 Hz electrical stimulation. **a**, Representative traces of cell shortening in isolated AMCMs from mice received AAV-Vector, AAV-*Opa3*, AAV-*Opa3*-Mut and *Opa3*^{iKO} mice at 8 weeks after sham or TAC surgery. **b** and **d**, Quantification of amplitude of cell shortening (**b**) and the maximum upstroke velocity of cell shortening (**c**) shown in (**a**) (5 cardiomyocytes per animal, 6 animals per group). **d**, Representative traces of calcium transients in isolated AMCMs from indicated groups. **e**–**g**, Quantification of amplitude of Ca^{2+} transients (**e**), maximal ascending rate in cell contractile Ca^{2+} transients (**f**) and baseline of Ca^{2+} transients (**g**) shown in (**d**) (5 cardiomyocytes per animal, 6 animals per group). **h**, Representative traces of Ca^{2+} transients during caffeine (10 mM)-induced contraction in NT solution in isolated AMCMs from indicated groups. **i** and **j**, Quantification of the rate constant of SERCA-mediated Ca^{2+} transport (**i**) and the amplitude of caffeine-induced Ca^{2+} transients (SR

Ca^{2+} content, **j**) shown in (**h**) (5 cardiomyocytes per animal, 6 animals per group). **k**, The Ca^{2+}-ATPase activities in isolated AMCMs from indicated groups were assayed using the Ca^{2+}-ATPase assay kit (n=6 per group). Data are expressed as mean ± SEM. Data in **b**, **c**, **e**, **f**, **g**, **i**, **j** were analyzed by nested one-way ANOVA followed by the Tukey post hoc test. Data in **k** were analyzed by one-way ANOVA followed by the Tukey post hoc test. AAV indicates adeno-associated viruses; AMCM, adult mouse cardiomyocyte; NT, normal Na^{+}/Ca^{2+} Tyrode's; OPA3, optic atrophy 3; SERCA, sarcoplasmic/endoplasmic reticulum Ca^{2+}-ATPase; SR, sarcoplasmic reticulum; TAC, transverse aortic constriction.

Supplementary Tables

Supplementary Table 1. Primers and siRNA used in this study.

Gene	Primer	Sequence	Application
Scn4b	Forward	TAACGGCTCATCGATCCTGC	RT-qPCR
	Reverse	CCCGTCGATGAGAATCCTGG	RT-qPCR
Agt	Forward	CTGGGGACCGCGTATACATC	RT-qPCR
	Reverse	AGGCTCGAACGTTGACTCTG	RT-qPCR
Agtr1a	Forward	AAAGGCCAAGTCGCACTCAA	RT-qPCR
	Reverse	CTCAGAACAAGACGCAGGCT	RT-qPCR
Fgf10	Forward	GGAGATGTCCGCTGGAGAAG	RT-qPCR
	Reverse	GCTGTTGATGGCTTTGACGG	RT-qPCR
Acsl1	Forward	CGCTTCCACAGATGATGTGC	RT-qPCR
	Reverse	GGGAACCACAGGGAAGATGG	RT-qPCR
Acsl6	Forward	TCGGACTTGGGCCAGTTTTT	RT-qPCR
	Reverse	CACCGTCCTCCACTTCTTCC	RT-qPCR
Edn1	Forward	TCTTCCAGGTCCAAGCGTTC	RT-qPCR
	Reverse	GGTGAGCGCACTGACATCTA	RT-qPCR
Vegfd	Forward	ACTGCAGTTGCTTTGAGTGC	RT-qPCR
	Reverse	CTCCTGGCTGTAGAGTCCCT	RT-qPCR
Il6	Forward	GAGGATACCACTCCCAACAGACC	RT-qPCR
	Reverse	AAGTGCATCATCGTTGTTCATACA	RT-qPCR
Fos	Forward	CGGGTTTCAACGCCGACTA	RT-qPCR
	Reverse	TTGGCACTAGAGACGGACAGA	RT-qPCR
Ptgs2	Forward	TGAGCAACTATTCCAAACCAGC	RT-qPCR
	Reverse	GCACGTAGTCTTCGATCACTATC	RT-qPCR
Tpm2	Forward	GAAGCCCAAGCGGACAAGTA	RT-qPCR
	Reverse	TCCTCGCTGATGGCCTTGTA	RT-qPCR
Cxcl10	Forward	CCAAGTGCTGCCGTCATTTTC	RT-qPCR
	Reverse	GGCTCGCAGGGATGATTTCAA	RT-qPCR
Tgfb3	Forward	GGACTTCGGCCACATCAAGA	RT-qPCR
	Reverse	ATAGGGGACGTGGGTCATCA	RT-qPCR
Nppa	Forward	AACCTGCTAGACCACCTGGA	RT-qPCR
	Reverse	ATCTATCGGAGGGGTCCCAG	RT-qPCR
Nppb	Forward	GGAGAACACGGCATCATTGC	RT-qPCR
	Reverse	TCCAGCAGCTGCATCTTGAA	RT-qPCR
Myh7	Forward	AGGTCTGGCTCTGAGCATTC	RT-qPCR
	Reverse	CGGCACAAGCATGCGTATTT	RT-qPCR
Col1a2	Forward	CACCTACAGCACCCTTGTGG	RT-qPCR
	Reverse	GGGAGGTCTTGGTGGTTTTG	RT-qPCR
Col3a1	Forward	CAGGCCAGTGGCAATGTAAAGA	RT-qPCR
	Reverse	CTCATTGCCTTGCGTGTTTGATA	RT-qPCR

Opa3 promoter (mouse)	Forward	ATGGGCAGGGAAATCGACTC	ChIP-qPCR
	Reverse	ACGTCCGTACGTTGGGTATG	ChIP-qPCR
Opa3 siRNA	Sense	5’-CCAGACUCAGCAGCGCAAUAATT-3’	Knockdown
	Antisense	5’-UUAUUGCGCUGCUGAGUCUGGTT-3’	Knockdown

Supplementary Table 2. Antibodies used in this study.

Antibody	Sourse	Identifier
OPA3	Proteintech	15638-1-AP
β-actin	Share-Bio	SB-AB0035
Flag	Sigma	F1804
HA	Cell Signaling Technology	3724S
MYC	Proteintech	16286-1-AP
PLN	Invitrogen	MA3-922
PLN	Abcam	Ab219626
^{s16}p-PLN	Abclonal	AP0907
SERCA	Share-Bio	CY6682
ANP	Santa Cruz	Sc-515701
MYH7	Abways	CY10011
VDAC	Abcam	ab14734
LaminB	Abways	AB0054
GAPDH	Share-Bio	SB-AB0037
TOM20	Abcam	ab56783
OPA1	Abcam	ab157457
ATF4	Proteintech	60035-1-lg
CHOP	Proteintech	15204-1-AP
MFN2	Proteintech	12186-1-AP
lgG(Rb)	Cell Signaling Technology	2729S
lgG(Ms)	Santa Cruz	sc-2025
MIA40	proteintech	21090-1-AP
RyR	Invitrogen	PA5-87416
Phospho-RyR2	Invitrogen	PA5-105712

Supplementary Table 3. Information of donors with non-failing hearts and DCM patients.

	NF (n=4)	DCM (n=4)	*P* value
Age (years)	54.50 (51.75–67.00)	67.50 (57.75-77.25)	0.1143
Male gender (n, %)	4 (100)	4 (100)	>0.99
DCM (n, %)	0 (0)	4 (100)	0.0286
AF (n, %)	0 (0)	3 (75)	0.1429
ICM (n, %)	0 (0)	0 (0)	>0.99
CHD (n, %)	0 (0)	0 (0)	>0.99

Data are expressed as median (IQR) or n (%). Two-sided Mann-Whitney *U* test was used for variables that were not normally distributed. Comparisons of categorical variables were conducted with Fisher's exact test. AF indicates atrial fibrillation; CHD, congenital heart disease; DCM, dilated cardiomyopathy; ICM, ischemic cardiomyopathy; IQR, interquartile range; NF, non-failing.

Supplementary Table 4. Predicted transcription factors.

hTFtarget	ENCODE	Cistrome	Jaspar	Intersection
SPI1	ARID3A	H2AZ	ARID3A	CREB1
CTCF	ATF1	NIPBL	CREB1	RELA
BCL6	ATF2	CBX5	YY1	
BRD3	ATF3	SP1	NFIC	
BRD4	BACH1	RAD21	RELA	
CBFB	BATF	EP300	PAX2	
CDK9	BCL11A	GLI2	SOX10	
CEBPB	BCL3	BRD4	SOX17	
CREB1	BCLAF1	E2F2		
E2F1	BHLHE40	SIN3A		
EBF1	BRCA1	TEAD4		
EGR1	CBX3	FOXM1		
ELF1	CCNT2	NANOG		
EP300	CEBPB	HDAC2		
ERG	CEBPD	SMC3		
ETS1	CEBPZ	NR3C1		
FLI1	CHD1	E2F8		
FOS	CHD2	ZNF518A		
GABPA	CHD4	ELF3		
GATA1	CREB1	POLR2A		
HDAC1	CREBBP	RELA		
HDAC2	CTCF	FOSL2		
IRF3	CUX1	GABPA		
IRF4	E2F4	ZNF589		
KLF1	E2F6	SRF		
MAX	EBF1	JUN		
MAZ	EGR1	EGR1		
MED1	ELF1	BCL3		
MYB	ELK1	ATF2		
MYC	ELK4	E2F5		
MYH11	EP300	CBX3		
NFYA	ETS1	VDR		
NFYB	EZH2	DAXX		
PAX5	FOS	ZNF687		
PBX3	FOSL2	PML		
POLR2A	FOXA1	L3MBTL2		
POU2F1	FOXA2	BRD2		
POU2F2	FOXM1	CTBP2		
RELA	FOXP2	NR2F2		
REST	GABPA	PRAME		
RUNX1	GATA2	TRAF7		

RUNX3	GATA3	ELF1
SP1	GTF2B	FAIRE
SPIB	GTF2F1	ZNF143
SRF	H2AFZ	PKNOX1
STAG1	HCFC1	PHF2
STAT1	HDAC1	USF1
STAT3	HDAC2	IRF4
STAT4	HDAC6	STAT3
TAF1	HMGN3	POU5F1
TCF12	HNF4A	MED1
TCF3	HNF4G	NR1H2
USF1	IRF1	HDAC1
YY1	IRF3	ELK3
NOTCH1	IRF4	FOXA1
RBPJ	JUN	ATF1
CLOCK	JUND	GABP
ESR1	KAT2A	TGIF2
ESR2	KDM1A	H2AFZ
HIF1A	KDM4A	MAX
ATF1	KDM5A	TRIM28
ATF3	KDM5B	SP4
BHLHE40	MAFF	MYBL2
CBX3	MAFK	MBD1_isoform2
E2F4	MAX	MYC
E2F6	MAZ	PHF8
HCFC1	MBD4	CHD2
IRF1	MTA3	SOX2
JUN	MXI1	SSU72
JUND	MYBL2	CEBPB
KDM5B	MYC	IRF1
MAFK	MYOG	KLF5
MXI1	NELFE	E2F7
NR2F2	NFE2	SS18
NRF1	NFIC	CBX1
PBX1	NFYA	E2F4
PHF8	NFYB	HBP1
PRAME	NR2F2	CTCF
RAD21	NRF1	USF2
RFX5	PAX5	SMC1A
SP2	PBX3	BCLAF1
SUZ12	PHF8	PRKDC
TBL1XR1	PML	MECP2
ZBTB7A	POLR2A	TCF7
ZNF143	POU2F2	FLI1

CREBBP	RBBP5	TET2
SIN3A	RCOR1	RNF2
AR	RELA	ZNF766
ARNT	REST	CREB1
BRCA1	RFX5	TFAP2A
FOSL1	RUNX3	DNMT3A
FOXA1	SAP30	E2F1
FOXM1	SETDB1	PTTG1
PGR	SIN3A	RCOR1
TCF7L2	SIRT6	KDM2B
TFAP2A	SIX5	TFDP1
TFAP2C	SMC3	
XBP1	SP1	
EHF	SP2	
GATAD1	SP4	
JMJD6	SRF	
SMC3	STAT1	
USF2	STAT3	
BARX2	STAT5A	
E2F3	TAF1	
E2F7	TAF7	
ELF2	TAL1	
ESRRA	TBL1XR1	
GLI2	TBP	
GMEB2	TCF12	
HES4	TCF7L2	
HINFP	TEAD4	
HNF4A	TRIM28	
HOXA1	UBTF	
HOXA4	USF1	
HOXA6	USF2	
JUNB	WHSC1	
KLF3	WRNIP1	
KLF5	YY1	
LYL1	ZBTB33	
MECOM	ZBTB7A	
MED12	ZC3H11A	
NFAT5	ZKSCAN1	
NIPBL	ZMIZ1	
RBCK1	ZNF143	
RFX2	ZNF384	
RXRA		
SMC1A		
TAF3		

TAL1
TFDP1
VEZF1
ZC3H8
ZNF280D
ATF2
CTBP2
FOXA2
FOXH1
FOXP1
GATA4
NANOG
OTX2
POU5F1
PRDM14
RBBP5
SAP30
SMAD1
SP4
TCF4
ZFP42
PPARG
GTF2I
KLF4
MAFB
PRKDC
RCOR1
EZH1
BRD2
ELK4
NCOR1
OGT
RNF2
SUMO2
TBL1X
TRIM28
ZNF263
ARID3A
CEBPZ
FOSL2
HNF4G
NFE2
ARRB1
LMNB1

NR3C1
RBL2
SIX5
WDR5
KDM4C
DDX5
DUX4
KAT5
KLF9
MITF
NRIP1
SMARCA4
CBX8
ZNF92
BRD7
NFIC
ELK3
GATA2
EWSR1

Supplementary Table 5. Echocardiographic analysis of cardia function in *Opa3*$^{fl/fl}$ and *Opa3*^{KO} mice.

	Opa3$^{fl/fl}$ 2 w (n=8)	*Opa3*^{KO} 2 w (n=8)	*Opa3*$^{fl/fl}$ 4 w (n=8)	*Opa3*^{KO} 4 w (n=8)	*Opa3*$^{fl/fl}$ 8 w (n=8)	*Opa3*^{KO} 8 w (n=8)
HR (BPM)	394±40	374±20^{ns}	463±31	421±55^{ns}	421±43	385±70^{ns}
EF (%)	62.28±6.46	53.54±10.34^{ns}	71.14±6.70	27.65±13.36***	71.05±8.81	22.15±9.76***
FS (%)	32.64±4.47	27.21±6.26^{ns}	39.94±5.20	13.13±6.85***	40.04±7.13	10.32±4.74***
LVID,d (mm)	3.10±0.30	3.47±0.39*	3.56±0.21	5.00±0.90**	3.41±0.41	5.41±0.84***
LVID,s (mm)	2.09±0.30	2.54±0.49*	2.14±0.28	4.40±1.07***	2.06±0.45	4.88±0.98***
LVAW,d (mm)	0.62±0.11	0.63±0.10^{ns}	0.80±0.09	0.55±0.10***	0.87±0.09	0.57±0.11***
LVAW,s (mm)	0.77±0.12	0.82±0.11^{ns}	1.07±0.12	0.65±0.13***	1.10±0.16	0.58±0.13***
LVPW,d (mm)	0.51±0.05	0.57±0.07^{ns}	0.77±0.13	0.50±0.15**	0.80±0.19	0.52±0.16**
LVPW,s (mm)	0.76±0.10	0.73±0.07^{ns}	1.22±0.27	0.52±0.17***	1.08±0.20	0.62±0.22***

Data are expressed as mean ± SD. Data were analyzed by two-sided Student's *t* test (equal variances) or Welch's *t* test (unequal variances). Opa3 indicates optic atrophy 3; HR, heart rate; BPM, beats per minute; EF, ejection fraction; FS, fractional shortening; LVID,d, internal dimension of the left ventricle in diastole; LVID,s, internal dimension of the left ventricle in systole; LVAW,d, end diastolic left ventricular anterior wall thickness; LVAW,s, end systolic left ventricular anterior wall thickness; LVPW,d, end diastolic left ventricular posterior wall thickness; LVPW,s, end systolic left ventricular posterior wall thickness. $^{*}P < 0.05$, $^{**}P < 0.01$, $^{***}P < 0.001$, ns, not significant compared with *Opa3*$^{fl/fl}$.

Supplementary Table 6. Echocardiographic analysis of cardia function in adult cardiomyocyte-specific *Opa3* deficiency mice and their littermate controls.

	Opa3$^{fl/fl}$ (n=8)	*Myh6*-MCM (n=8)	*Opa3*^{iKO} (n=8)
HR (BPM)	454±71	449±42^{ns}	470±43^{ns}
EF (%)	76.13±4.36	74.77±4.28^{ns}	56.68±6.14***
FS (%)	43.86±4.14	42.66±3.82^{ns}	29.37±3.97***
LVID,d (mm)	3.21±0.31	3.28±0.23^{ns}	3.93±0.32***
LVID,s (mm)	1.80±0.17	1.88±0.18^{ns}	2.78±0.34***
LVAW,d (mm)	0.90±0.08	0.84±0.08^{ns}	0.75±0.05**
LVAW,s (mm)	1.13±0.11	1.13±0.06^{ns}	0.91±0.08***
LVPW,d (mm)	0.87±0.14	0.84±0.08^{ns}	0.68±0.06**
LVPW,s (mm)	1.09±0.13	1.07±0.07^{ns}	0.88±0.06***

Data are expressed as mean ± SD. Data were analyzed by one-way ANOVA followed by the Tukey post hoc test. Opa3 indicates optic atrophy 3; MCM, MerCreMer; HR, heart rate; BPM, beats per minute; EF, ejection fraction; FS, fractional shortening; LVID,d, internal dimension of the left ventricle in diastole; LVID,s, internal dimension of the left ventricle in systole; LVAW,d, end diastolic left ventricular anterior wall thickness; LVAW,s, end systolic left ventricular anterior wall thickness; LVPW,d, end diastolic left ventricular posterior wall thickness; LVPW,s, end systolic left ventricular posterior wall thickness. $^{**}P < 0.01$, $^{***}P < 0.001$, ns, not significant compared with *Opa3*$^{fl/fl}$.

Supplementary Table 7. Echocardiographic analysis of cardia function in adult cardiomyocyte-specific *Opa3* deficiency mice and their littermate controls after TAC surgery.

	Opa3$^{fl/fl}$-TAC (n=8)	*Opa3*^{iKO}-TAC (n=8)
HR (BPM)	450±67	414±35^{ns}
EF (%)	46.87±5.72	21.93±7.16***
FS (%)	23.31±3.45	10.20±3.48***
LVID,d (mm)	4.20±0.28	5.51±0.49***
LVID,s (mm)	3.23±0.31	4.96±0.61***
LVAW,d (mm)	1.04±0.09	0.86±0.10**
LVAW,s (mm)	1.24±0.07	0.99±0.13***
LVPW,d (mm)	1.02±0.13	0.83±0.12***
LVPW,s (mm)	1.30±0.16	0.91±0.15***

Data are expressed as mean ± SD. Data were analyzed by two-sided Student's *t* test (equal variances) or Welch's *t* test (unequal variances). Opa3 indicates optic atrophy 3; TAC, transverse aortic constriction; HR, heart rate; BPM, beats per minute; EF, ejection fraction; FS, fractional shortening; LVID,d, internal dimension of the left ventricle in diastole; LVID,s, internal dimension of the left ventricle in systole; LVAW,d, end diastolic left ventricular anterior wall thickness; LVAW,s, end systolic left ventricular anterior wall thickness; LVPW,d, end diastolic left ventricular posterior wall thickness; LVPW,s, end systolic left ventricular posterior wall thickness. $^{**}P < 0.01$, $^{***}P < 0.001$, ns, not significant compared with *Opa3*$^{fl/fl}$-TAC.

Supplementary Table 8. Echocardiographic analysis of cardia function in mice with cardiomyocyte-specific *Opa3* overexpression in a doxorubicin-induced cardiomyopathy model.

	AAV-Vector-NS (n=8)	AAV-*Opa3*-NS (n=8)	AAV-Vector-DOX (n=8)	AAV-*Opa3*-DOX (n=8)
HR (BPM)	401±46	400±55	431±45^{ns}	465±42^{ns}
EF (%)	71.70±6.75	72.79±5.56	52.73±7.12***	63.11±3.01$^{\#\#}$
FS (%)	40.14±5.86	41.02±4.68	26.89±4.59***	33.53±2.18$^{\#}$
LVID,d (mm)	3.15±0.21	3.21±0.12	4.02±0.25***	3.65±0.37$^{\#}$
LVID,s (mm)	1.89±0.23	1.89±0.18	2.94±0.27***	2.42±0.25$^{\#\#\#}$
LVAW,d (mm)	0.88±0.09	0.90±0.08	0.55±0.10***	0.70±0.05$^{\#\#}$
LVAW,s (mm)	1.12±0.12	1.12±0.14	0.72±0.09***	1.02±0.14$^{\#\#\#}$
LVPW,d (mm)	0.84±0.09	0.85±0.13	0.52±0.12***	0.67±0.07$^{\#}$
LVPW,s (mm)	1.16±0.10	1.17±0.10	0.73±0.11***	0.88±0.07$^{\#}$

Data are expressed as mean ± SD. Data were analyzed by one-way ANOVA followed by the Tukey post hoc test. Opa3 indicates optic atrophy 3; AAV, adeno-associated viruses; NS, normal saline; DOX, doxorubicin; HR, heart rate; BPM, beats per minute; EF, ejection fraction; FS, fractional shortening; LVID,d, internal dimension of the left ventricle in diastole; LVID,s, internal dimension of the left ventricle in systole; LVAW,d, end diastolic left ventricular anterior wall thickness; LVAW,s, end systolic left ventricular anterior wall thickness; LVPW,d, end diastolic left ventricular posterior wall thickness; LVPW,s, end systolic left ventricular posterior wall thickness. $^{***}P < 0.001$, ns, not significant compared with AAV-Vector-NS; $^{\#}P < 0.05$, $^{\#\#}P < 0.01$, $^{\#\#\#}P < 0.001$, ns, not significant compared with AAV-Vector-DOX.

Supplementary Table 9. 36 proteins that uniquely presented in the overlap of the Flag-IP and GFP-IP groups.

Confidence	Accession	Protein	Abundance
High	Q505D7	Opa3	32420832
Medium	E9Q0B5	Fcgbp	11386831
High	Q99M74	Krt82	3452357
High	Q91VA7	Idh3b	1240014
High	Q9CZ13	Uqcrc1	1093275
High	J3QMG3	Vdac3	1034871
High	P61014	Pln	1026592
High	P50446	Krt6a	981015.3
High	Q60597	Ogdh	946810
High	Q8VDN2	Atp1a1	934060.8
High	D3YUM1	Ndufv1	785676.4
High	Q9CQC7	Ndufb4	568950.5
Medium	Q6ZQ06	Cep162	476065.2
High	Q9QXX4	Slc25a13	475765.3
High	Q08857	Cd36	455322.3
High	E9PZ67	Casq2	431584.8
High	Q9Z2Z6	Slc25a20	415851.8
High	O08749	Dld	411279.7
High	Q9D1G3	Hhatl	368046.5
High	A0A0U1RPT5	Aldoa	361868.7
High	F6QPR1	Phb2	345903.3
High	F6RBR6	Ndufa12	326000.4
High	A0A1L1SRG5	Cryab	318066.6
High	Q60936	Coq8a	315490
High	Q9ET78	Jph2	247059.5
High	B1AU25	Aifm1	192081.6
High	Q791V5	Mtch2	188252.2
High	A0A0R4J083	Acadl	172294.8
High	P03930	Mtatp8	171290.8
High	Q9CPQ8	Atp5mg	161630.1
Medium	Q8BKZ9	Pdhx	121520.9
High	Q8K3J1	Ndufs8	116252.4
High	Q9CQZ5	Ndufa6	111796.1
Medium	P63276	Rps17	99139.9
High	A2AI89	Phka1	95159.04
High	P11499	Hsp90ab1	90164.61

Supplementary Table 10. Echocardiographic analysis of cardia function in mice with cardiomyocyte-specific *Opa3* overexpression in a TAC-induced cardiomyopathy model.

	AAV-Vector-Sham (n=8)	AAV-Vector-TAC (n=8)	AAV-*Opa3*-TAC (n=8)	AAV-*Opa3*-Mut-TAC (n=8)
HR (BPM)	466±44	432±37	460±52^{ns}	435±50^{ns}
EF (%)	76.73±5.25	45.08±4.62	63.51±6.92***	43.43±5.99$^{###}$
FS (%)	44.62±5.04	22.17±2.65	34.01±5.17***	21.36±3.53$^{###}$
LVID,d (mm)	3.21±0.32	4.14±0.31	3.61±0.25**	4.34±0.29$^{###}$
LVID,s (mm)	1.77±0.17	3.22±0.26	2.38±0.25***	3.42±0.28$^{###}$
LVAW,d (mm)	0.76±0.07	0.99±0.05	0.86±0.10*	0.99±0.06$^{#}$
LVAW,s (mm)	1.03±0.08	1.23±0.09	1.10±0.07*	1.23±0.09$^{#}$
LVPW,d (mm)	0.76±0.06	0.99±0.12	0.78±0.10***	0.93±0.07$^{##}$
LVPW,s (mm)	1.02±0.09	1.19±0.07	1.00±0.10**	1.16±0.10$^{#}$

Data are expressed as mean ± SD. Data were analyzed by one-way ANOVA followed by the Tukey post hoc test. Opa3 indicates optic atrophy 3; TAC, transverse aortic constriction; AAV, adeno-associated viruses; HR, heart rate; BPM, beats per minute; EF, ejection fraction; FS, fractional shortening; LVID,d, internal dimension of the left ventricle in diastole; LVID,s, internal dimension of the left ventricle in systole; LVAW,d, end diastolic left ventricular anterior wall thickness; LVAW,s, end systolic left ventricular anterior wall thickness; LVPW,d, end diastolic left ventricular posterior wall thickness; LVPW,s, end systolic left ventricular posterior wall thickness. $^{*}P < 0.05$, $^{**}P < 0.01$, $^{***}P < 0.001$, ns, not significant compared with AAV-Vector-TAC; $^{#}P < 0.05$, $^{##}P < 0.01$, $^{###}P < 0.001$, ns, not significant compared with AAV-*Opa3*-TAC.
