# Supplementary material for: Mitochondrial protein OPA3 sustains cardiac function by regulating calcium handling in male mice
Source: Nat Commun. 2026 Jun 17;17:7665. doi: 10.1038/s41467-026-73991-4 (PMC13434718; doi:10.1038/s41467-026-73991-4)
Supplement: Supplementary file 2 [file 41467_2026_73991_MOESM2_ESM.pdf]

nature portfolio

Corresponding author(s): Xiyuan Lu

Last updated by author(s): Mar 20, 2026

Reporting Summary

Nature Portfolio wishes to improve the reproducibility of the work that we publish. This form provides structure for consistency and transparency in reporting. For further information on Nature Portfolio policies, see our Editorial Policies and the Editorial Policy Checklist.

Statistics

For all statistical analyses, confirm that the following items are present in the figure legend, table legend, main text, or Methods section.

n/a	Confirmed	
☐	☒	The exact sample size (*n*) for each experimental group/condition, given as a discrete number and unit of measurement
☐	☒	A statement on whether measurements were taken from distinct samples or whether the same sample was measured repeatedly
☐	☒	The statistical test(s) used AND whether they are one- or two-sided<br>*Only common tests should be described solely by name; describe more complex techniques in the Methods section.*
☐	☒	A description of all covariates tested
☐	☒	A description of any assumptions or corrections, such as tests of normality and adjustment for multiple comparisons
☐	☒	A full description of the statistical parameters including central tendency (e.g. means) or other basic estimates (e.g. regression coefficient) AND variation (e.g. standard deviation) or associated estimates of uncertainty (e.g. confidence intervals)
☐	☒	For null hypothesis testing, the test statistic (e.g. *F*, *t*, *r*) with confidence intervals, effect sizes, degrees of freedom and *P* value noted<br>*Give P values as exact values whenever suitable.*
☒	☐	For Bayesian analysis, information on the choice of priors and Markov chain Monte Carlo settings
☒	☐	For hierarchical and complex designs, identification of the appropriate level for tests and full reporting of outcomes
☒	☐	Estimates of effect sizes (e.g. Cohen's *d*, Pearson's *r*), indicating how they were calculated

Our web collection on statistics for biologists contains articles on many of the points above.

Software and code

Policy information about availability of computer code

Data collection	Bulk RNA-seq data were generated using the Illumina NovaSeq 6000 platform following standard library preparation and sequencing protocols.
Data analysis	Softwares: Trimmomatic (v0.39), HISAT2 (v2.1.0), StringTie (v2.1.3b), htseq-count (v0.11.2)<br>R packages: DESeq2 (1.22.2),Seurat (v5.0.1),ggplot2 (v3.5.1)<br><br>Other software: GraphPad Prism (v8.4.3)

For manuscripts utilizing custom algorithms or software that are central to the research but not yet described in published literature, software must be made available to editors and reviewers. We strongly encourage code deposition in a community repository (e.g. GitHub). See the Nature Portfolio guidelines for submitting code & software for further information.

Data

Policy information about availability of data

All manuscripts must include a data availability statement. This statement should provide the following information, where applicable:

- Accession codes, unique identifiers, or web links for publicly available datasets
- A description of any restrictions on data availability
- For clinical datasets or third party data, please ensure that the statement adheres to our policy

The RNA-seq data generated in this study have been deposited in National Center for Biotechnology Information (NCBI) under the project ID PRJNA1460849 (https://www.ncbi.nlm.nih.gov/bioproject/PRJNA1460849). Publicly available single-nucleus transcriptomic data of human cardiomyopathy are available through the Broad Institute's Single Cell Portal (https://singlecell.broadinstitute.org/singlecell) under project ID SCP1303, SCP1849, and SCP1852. Public transcriptomic data from DCM murine model and corresponding controls are available on Gene Expression Omnibus under project ID GSE168610 (https://www.ncbi.nlm.nih.gov/geo/query/acc.cgi?acc=GSE168610). All data supporting the findings of this study are available in the article, in the Supplementary Information, and in the Source Data file.

Research involving human participants, their data, or biological material

Policy information about studies with human participants or human data. See also policy information about sex, gender (identity/presentation), and sexual orientation and race, ethnicity and racism.

Reporting on sex and gender	All donors included in this study were male. No sex-based analysis was performed due to the limited sample size.
Reporting on race, ethnicity, or other socially relevant groupings	Information on race, ethnicity, or other socially relevant groupings was not collected, as these variables are not relevant to the biological processes examined.
Population characteristics	Human heart tissue samples were obtained from patients with dilated cardiomyopathy (DCM) undergoing heart transplantation (n = 4) and from non-failing donor hearts (n = 4). Baseline characteristics, including age, sex, and comorbidities, are summarized in Supplementary Table 3. All participants were male. Data are presented as median (interquartile range) or number (percentage).
Recruitment	Failing heart tissues were collected from patients with DCM undergoing heart transplantation at Nanjing First Hospital of Nanjing Medical University. Non-failing heart tissues were obtained from donor hearts deemed unsuitable for transplantation.
Ethics oversight	This study was conducted in accordance with the Declaration of Helsinki. The use of human heart tissue samples was approved by the Ethics Committee of Nanjing First Hospital of Nanjing Medical University (approval no. KY20190404-03-KS-01). Written informed consent was obtained from all patients or their legal representatives.

Note that full information on the approval of the study protocol must also be provided in the manuscript.

Field-specific reporting

Please select the one below that is the best fit for your research. If you are not sure, read the appropriate sections before making your selection.

[x] Life sciences [ ] Behavioural & social sciences [ ] Ecological, evolutionary & environmental sciences

For a reference copy of the document with all sections, see nature.com/documents/nr-reporting-summary-flat.pdf

Life sciences study design

All studies must disclose on these points even when the disclosure is negative.

Sample size	No formal statistical power calculation was performed to predetermine sample size. For each experiment, sample sizes were determined with based on similar studies previously published in the field, where such sample sizes are widely accepted. The chosen sample sizes were sufficient to produce consistent results across repeated experiments.
Data exclusions	No data were excluded from the analyses. All collected data points were included in the final analysis.
Replication	All experiments were performed using biological replicates derived from independent animals or independent primary cell isolations. Each experiment was independently repeated at least three times. The number of biological replicates (n) is specified in the corresponding figure legends.
Randomization	Animals were randomly assigned to experimental groups using a randomization method. For cell-based experiments, samples were allocated to groups without specific selection bias.
Blinding	Investigators were blinded to group allocation during data acquisition and analysis.

Reporting for specific materials, systems and methods

We require information from authors about some types of materials, experimental systems and methods used in many studies. Here, indicate whether each material, system or method listed is relevant to your study. If you are not sure if a list item applies to your research, read the appropriate section before selecting a response.

Materials & experimental systems

n/a	Involved in the study	
☐	☒	Antibodies
☐	☒	Eukaryotic cell lines
☒	☐	Palaeontology and archaeology
☐	☒	Animals and other organisms
☒	☐	Clinical data
☒	☐	Dual use research of concern
☒	☐	Plants

Methods

n/a	Involved in the study	
☒	☐	ChIP-seq
☒	☐	Flow cytometry
☒	☐	MRI-based neuroimaging

Antibodies

Antibodies used

Specificity; clone; manufacturer; identifier
anti-human/mouse OPA3; Proteintech; 15638-1-AP
anti-human/mouse β-Actin; Share-Bio; SB-AB0035
anti-Flag; Sigma;F1804
anti-HA;Cell Signaling Technology;3724S
anti-MYC; Proteintech;16286-1-AP
anti-mouse PLN;Invitrogen; MA3-922
anti-mouse PLN;Abcam; Ab219626
anti-mouse s16p-PLN;Abclonal; AP0907
anti-mouse SERCA; Share-Bio; CY6682
anti-mouse ANP;Santa Cruz; Sc-515701
anti-mouse MYH7;Abways; DY10011
anti-mouse VDAC;Abcam; ab14734
anti-mouse LaminB; Abways
anti-mouse GAPDH; Share-Bio; SB-AB0037
anti-mouse TOM20;Abcam; ab56783
anti-mouse OPA1; Abcam; ab157457
anti-mouse ATF4;Proteintech; 60035-1-lg
anti-mouse CHOP; Proteintech; 15204-1-AP
anti-mouse MFN2;Proteintech; 12186-1-AP
anti-lgG(Rb);Cell Signaling Technology;2729S
anti-lgG(Ms); Santa Cruz;sc-2025
anti-mouse MIA40;proteintech;21090-1-AP
anti-mouse RyR;Invitrogen;PA5-87416
anti-mouse Phospho-RyR2 (Ser2808) ;Invitrogen;PA5-105712

Validation

All antibodies are commercially available and validation statements can be found on the manufacturers' websites using the catalogue number.
anti-human/mouse OPA3 antibody (https://www.ptgcn.com/products/OPA3-Antibody-15638-1-AP.htm). 15638-1-AP targets OPA3 in WB, IHC, IF/ICC, IP, ELISA applications and shows reactivity with human, mouse, rat samples.
anti-human/mouse β-Actin antibody (https://www.share-bio.com/product/PNOSB-AB0035.html). The beta-Actin antibody in WB, IHC, IF/ICC applications and shows reactivity with human, mouse, rat samples.
anti-Flag antibody (https://www.sigmaaldrich.cn/CN/zh/product/sigma/f1804).The Flag antibody in WB, IP, IHC, IF/ICC applications and shows reactivity with recombinant protein samples.
anti-HA antibody (https://www.cellsignal.cn/products/primary-antibodies/ha-tag-c29f4-rabbit-mab/3724).The HA antibody in WB, IP, IHC, IF/ICC, Flow Cytometry applications and shows reactivity with recombinant protein samples.
anti-MYC antibody (https://www.ptgcn.com/products/MYC-tag-Antibody-16286-1-AP.htm).16286-1-AP targets MYC tag in WB, IHC, IF/ICC, IP, CoIP, ChIP, ELISA applications and shows reactivity with recombinant protein samples.
anti-PLN antibody (https://www.thermofisher.cn/cn/zh/antibody/product/Phospholamban-Antibody-clone-2D12-Monoclonal/MA3-922). MA3-922 has been successfully used in Western blot, immunofluorescence, immunohistochemistry and immunoprecipitation procedures and shows reactivity with dog, chicken, guinea pig, human, mouse, sheep, pig, rabbit, rat.
anti-PLN antibody (https://www.abcam.cn/products/primary-antibodies/phospholamban-antibody-epr21897-ab219626.html).The PLN antibody in WB, IHC-P, IP applications and shows reactivity with mouse, rat, human.
anti-s16p-PLN antibody (https://abclonal.com.cn/catalog/AP0907). The phospho-PLN-S16 antibody in WB, ELISA applications and shows reactivity with Human, Mouse, Rat.
anti-SERCA antibody (https://www.share-bio.com/product/PNOCY6682S.html).The SERCA antibody in WB;IHC;ICC/IF;IHF;FC

applications and shows reactivity with mouse, rat, human.
anti-ANP antibody (https://www.scbt.com/zh/p/anp-antibody-f-2).The ANP antibody in WB;IF;IP; ELISA applications and shows reactivity with mouse, rat, human.
anti-MYH7 antibody (https://www.abways.cn/ProductsStd/DY1011.html).The MYH7 antibody in WB;IHC applications and shows reactivity with mouse, rat, human.
anti-VDAC antibody (https://www.abcam.cn/products/primary-antibodies/vdac1porin--vdac3-antibody-20b12af2-ab14734.html?productWallTab=Abreviews).The VDAC antibody in WB;IICC/IF, Flow Cyt applications and shows reactivity with mouse, rat, human, cow.
anti-LaminB antibody (https://www.abways.cn/ProductsStd/AB0054.html).The LaminB antibody in WB;IHC;ICC/IF;IHF;IP applications and shows reactivity with mouse, rat, human.
anti-GAPDH antibody (https://www.share-bio.com/product/PNOSB-AB0037.html).The GAPDH antibody in WB;ICC/IF Flow Cyt applications and shows reactivity with mouse, rat, human, cow.
anti-TOM20 antibody (https://www.abcam.cn/products/primary-antibodies/tomm20-antibody-4f3-bsa-and-azide-free-ab56783.html).The TOM20 antibody in WB, IHC-P, Flow Cyt, IP, ICC/IF applications and shows reactivity with mouse, rat, human.
anti-OPA1 antibody (https://www.abcam.cn/products/primary-antibodies/opa1-antibody-epr11057b-ab157457.html).The OPA1 antibody in WB, IHC-P, IHC-Fr applications and shows reactivity with mouse, rat, human.
anti-ATF4 antibody (https://www.ptgcn.com/products/ATF4-Antibody-60035-1-Ig.htm).The ATF4 antibody in WB, IHC, IF/ICC, FC (Intra), ELISA applications and shows reactivity with human, mouse, rat.
anti-CHOP antibody (https://www.ptgcn.com/products/DDIT3-Antibody-15204-1-AP.htm).The CHOP antibody in WB, IHC, IF/ICC, FC (Intra), IP, ELISA applications and shows reactivity with human, mouse, rat.
anti-MFN2 antibody (https://www.ptgcn.com/products/MFN2-Antibody-12186-1-AP.htm).The MFN2 antibody in WB, IHC, IP, ELISA applications and shows reactivity with human, mouse, rat.
anti-Normal Rabbit IgG antibody (https://www.cellsignal.cn/products/primary-antibodies/normal-rabbit-igg/2729).The Normal Rabbit IgG antibody in IP, CHIP,CUT Tag applications.
anti-Normal Rabbit IgG antibody (https://www.cellsignal.cn/products/primary-antibodies/normal-rabbit-igg/2729).The Normal Rabbit IgG antibody in IP, WB,IHC applications.
anti- MIA40 antibody(https://www.ptgcn.com/products/CHCHD4-Antibody-21090-1-AP.htm).The MIA40 antibody in WB, IHC, IF/ICC, IP, ELISA applications and shows reactivity with human, mouse, rat.
anti-RyR2 antibody(https://www.thermofisher.cn/cn/zh/antibody/product/RyR2-Antibody-Polyclonal/PA5-87416).The RyR in WB,IHC,ELISA applications and shows reactivity with human, mouse.
anti-Phospho-RyR2 antibody(https://www.thermofisher.cn/cn/zh/antibody/product/Phospho-RyR2-Ser2808-Antibody-Polyclonal/PA5-105712).The Phospho-RyR in WB,IHC applications and shows reactivity with human, mouse, rat.

Eukaryotic cell lines

Policy information about cell lines and Sex and Gender in Research

Cell line source(s)	HEK293T cells (human embryonic kidney cells) and H9C2 rat cardiomyoblast cell line was purchased from ATCC (USA).
Authentication	No additional authentication was performed for the cell lines used in this study.
Mycoplasma contamination	All cell lines were routinely tested and confirmed to be negative for mycoplasma contamination.
Commonly misidentified lines (See ICLAC register)	No commonly misidentified cell lines were used in this study.

Animals and other research organisms

Policy information about studies involving animals; ARRIVE guidelines recommended for reporting animal research, and Sex and Gender in Research

Laboratory animals	Animal experiments were conducted with approval from the Animal Ethics Committee of Renji Hospital and followed the National Institutes of Health Guidelines for the Care and Use of Laboratory Animals. All animals were kept at 24°C ± 2°C, humidity of 40% ± 5%, under a constant 12-hour light/dark cycle with free access to diet and water. Animals were monitored daily for general health status, including body weight, activity, posture, and signs of distress. Mice exhibiting severe distress, rapid weight loss (>20%), impaired mobility, or signs of advanced heart failure were euthanized. At the experimental endpoint, mice were anesthetized in an induction chamber using 2% isoflurane mixed with oxygen prior to sacrifice.<br>Opa3fl/fl mice on the C57BL/6J genetic background were constructed by Cyagen Biosciences Inc., with exon 2 of the Opa3 gene being flanked by loxP sites. Cardiomyocyte-specific Opa3 knockout male mice (Opa3KO) were generated by crossing Opa3fl/fl mice with those carrying cardiomyocyte-specific Cre (Myh6-Cre). And inducible cardiomyocyte-specific Opa3 knockout mice (Opa3iKO) were established by crossing Opa3fl/fl mice with those carrying cardiomyocyte-specific MerCreMer (Myh6-MCM). To activate MCM, 6-week-old mice were administrated with tamoxifen intraperitoneally at a dosage of 30 mg/kg/day for five consecutive days.
Wild animals	This study did not involve wild animals.
Reporting on sex	In this study, we primarily used male mice to reduce variability associated with hormonal fluctuations. However, this represents a limitation of the present work, as potential sex-specific differences were not evaluated. Future studies incorporating both male and female mice will be necessary to determine whether OPA3 exerts sex-dependent effects in the heart.
Field-collected samples	The study did not involve samples collected from the field.
Ethics oversight	The protocols for randomized animal experiments were approved by the Institute's Animal Ethics Committee of Shanghai Jiao Tong University, School of Medicine, Renji Hospital and adhered to the NIH Guidelines for the Care and Use of Laboratory Animals.

Note that full information on the approval of the study protocol must also be provided in the manuscript.

Plants

Seed stocks	N/A
Novel plant genotypes	N/A
Authentication	N/A
