# Supplementary material for: Mitochondrial protein OPA3 sustains cardiac function by regulating calcium handling in male mice
Source: Nat Commun. 2026 Jun 17;17:7665. doi: 10.1038/s41467-026-73991-4 (PMC13434718; doi:10.1038/s41467-026-73991-4)
Supplement: Supplementary file 3 [file 41467_2026_73991_MOESM3_ESM.pdf]

nature portfolio

Peer Review File

Mitochondrial protein OPA3 sustains cardiac function by regulating calcium handling

Corresponding Author: Professor Xiyuan Lu

This file contains all reviewer reports in order by version, followed by all author rebuttals in order by version.

Version 1:

Reviewer comments:

Reviewer #1

(Remarks to the Author)
I believe the authors have done a commendable job in addressing the reviewers' comments. The additional supplementary figures are convincing and strengthen the manuscript. I have no further comments or questions.

Reviewer #2

(Remarks to the Author)
The authors have responded favorably to my previous concerns.
Only one issue requires attention. The images shown in supplementary Fig 13 does not have any detectable Ca sparks. Please replace with images where Ca sparks are clearly visible. Maybe eliminating the portion of the ca transients will allow enough contrast to see them.

I failed to see which RyR2 residue's phosphorylation was studied in the new experiments. These data should be added to the new version, either in the supplementary figure, or in the text.

Reviewer #3

(Remarks to the Author)
The authors have responded well to each of my points raised in the first review, carrying out additional experiments and altering the text. They also seem to have responded positively to the other reviewers' comments, and I believe have produced an improved manuscript.

Reviewer #4

(Remarks to the Author)
I appreciate the effort the authors have made to address my concerns and comments. I have no futher comments.

Open Access This Peer Review File is licensed under a Creative Commons Attribution 4.0 International License, which permits use, sharing, adaptation, distribution and reproduction in any medium or format, as long as you give appropriate credit to the original author(s) and the source, provide a link to the Creative Commons license, and indicate if changes were made.
In cases where reviewers are anonymous, credit should be given to 'Anonymous Referee' and the source.
The images or other third party material in this Peer Review File are included in the article's Creative Commons license, unless indicated otherwise in a credit line to the material. If material is not included in the article's Creative Commons license and your intended use is not permitted by statutory regulation or exceeds the permitted use, you will need to obtain permission directly from the copyright holder.
To view a copy of this license, visit https://creativecommons.org/licenses/by/4.0/

Detailed Response to the Reviewer #1

General comments:

In this manuscript, Geng and coworkers show that the Optic atrophy (OPA)-3 (OPA3) gene regulates mitochondrial function in cardiomyocytes and can participate in the pathogenetic mechanisms that lead to dilated cardiomyopathy (DCM). As requested by the Editor, I limit my comments to the overall relevance of the study and the gene transfer part, leaving to other reviewers the assessment of the mechanistic studies.

Response:

We sincerely thank you for taking the time to evaluate the overall relevance of our study and the gene transfer experiments. We have carefully followed your recommendations and made every possible effort to address these concerns. Our detailed responses are provided below.

1. There is ample evidence (and a strong rationale) for the involvement of OPA genes in the development of heart failure. Multiple studies (several of which also cited by the authors) show that these genes play important roles in mitochondrial function and that their dysregulation or downregulation leads to cardiac dysfunction. A connection between OPA1 and OPA3 with cardiomyopathy is well established in multiple animal species as well as in patients. Thus, there is not much surprise in the findings reported here in tissue specific and conditional knock out mice. I am afraid, however, that the finding that OPA3 is downregulated in DCM and can cause cardiomyopathy does not prove that it takes part in DCM pathogenesis in patients, as the authors wish to imply and also explicitly state. The authors are correct in stating that "the pathogenesis and underlying molecular mechanisms contributing to the onset of dilated cardiomyopathy (DCM) remain incompletely understood.". But the overall findings of this manuscript point to a correlation of heart failure with OPA3 downregulation, while they do not prove that OPA3 is casually linked to the pathogenesis of DCM. Should the authors have focused their attention to other OPA proteins, or to other proteins of the mitochondria, they would have most likely phenocopied the same results. This appears to me as an important conceptual flaw of this manuscript.

Response:

Thank you for this insightful and valuable comment. We fully agree with you that our current findings demonstrate a strong correlation between OPA3 downregulation and heart failure, but do not definitively establish a causal role for OPA3 in the pathogenesis

of human DCM. To address this important conceptual concern, we have carefully revised the **Introduction**, **Results**, and **Discussion** sections to avoid any overstatement. Specifically, we now use the term "heart failure" when referring to clinical observations and implications, while reserving "DCM" to describe phenotypic features observed in our murine models. The relevant revisions have been highlighted in the manuscript. We acknowledge that further investigations are required to elucidate whether reduced OPA3 expression directly contributes to disease progression in human DCM.

Nevertheless, we note that OPA3 has been strongly implicated in the development of DCM in animals. Evidence indicates that mutations in OPA3 cause DCM in cattle,[1] suggesting that genetic defects in OPA3 may predispose to DCM. In addition, we believe our data provide compelling evidence for a pathogenic role of OPA3 deficiency in driving cardiac dysfunction in experimental models. In both TAC- and doxorubicin-induced cardiomyopathy models, we observed a progressive reduction in OPA3 expression concomitant with the development of heart failure (Fig. 1e, f). Importantly, cardiomyocyte-specific ablation of *Opa3* resulted in chamber dilation and severe cardiac dysfunction (Fig. 2 and Supplementary Fig. 4, 6), whereas cardiomyocyte-specific overexpression of *Opa3* significantly attenuated the heart failure phenotype induced by TAC or doxorubicin administration (Fig. 8 and Supplementary Fig. 8). Taken together, these findings indicate that both acquired downregulation of OPA3 in cardiomyocytes and genetic disruption of OPA3 exacerbate cardiac dysfunction over time, thereby promoting DCM-like remodeling in animal models.

While we agree that multiple mitochondrial proteins are critical for maintaining mitochondrial integrity and their deficiency may cause similar cardiac dysfunction, we underscore that the underlying mechanisms may differ based on the specific localization and roles of these proteins. Specifically, our data suggest that OPA3 has a unique role in modulating sarcoplasmic reticulum (SR) calcium dynamics through its interaction with phospholamban (PLN) and indirect regulation of SERCA2a activity. This mechanism appears distinct from other mitochondrial factors such as OPA1, which is primarily involved in inner mitochondrial membrane fusion and cristae maintenance, but not known to directly influence SR-mitochondrial calcium cross-talk. Notably, OPA1 and OPA3 are not homologous and may have distinct cellular functions (Fig. R1 and R2). We have expanded the description to clearly outline these mechanistic distinctions, "OPA1 is well-documented for its critical role in maintaining mitochondrial homeostasis and cardiac function.[11, 13, 15] OPA1 contains a GTPase domain that facilitates nucleotide-dependent dimerization, driving conformational changes and oligomer stabilization essential for MIM fusion.[47] Imbalanced OPA1 processing leads to mitochondrial fragmentation and disruption of cardiac metabolism, which have been implicated in progression of DCM.[13] In contrast, OPA3 exhibits minimal sequence homology with OPA1 and lacks a GTPase domain, and its biological

functions have remained far less understood. Our findings reveal that OPA3 also undergoes multimerization, a mechanism fundamentally distinct from the GTPase-driven oligomerization of OPA1. There was also controversy regarding the localization of OPA3 to the MIM vs. MOM in earlier studies.[19, 20] OPA3 was initially thought to be located in the MIM based on sequence predictions,[20] but our research demonstrated that OPA3 mainly localized to the MOM in cardiomyocyte mitochondria, aligning with prior observations in HeLa cells.[19] Taken together, the distinction between OPA1 and OPA3 provides important context for interpreting the role of OPA3 uncovered in our study and highlights the need to consider OPA3 as a functionally independent regulator in cardiac physiology rather than as a structural or evolutionary analogue of OPA1." You can also refer to the **Discussion** section in Lines 797–816.

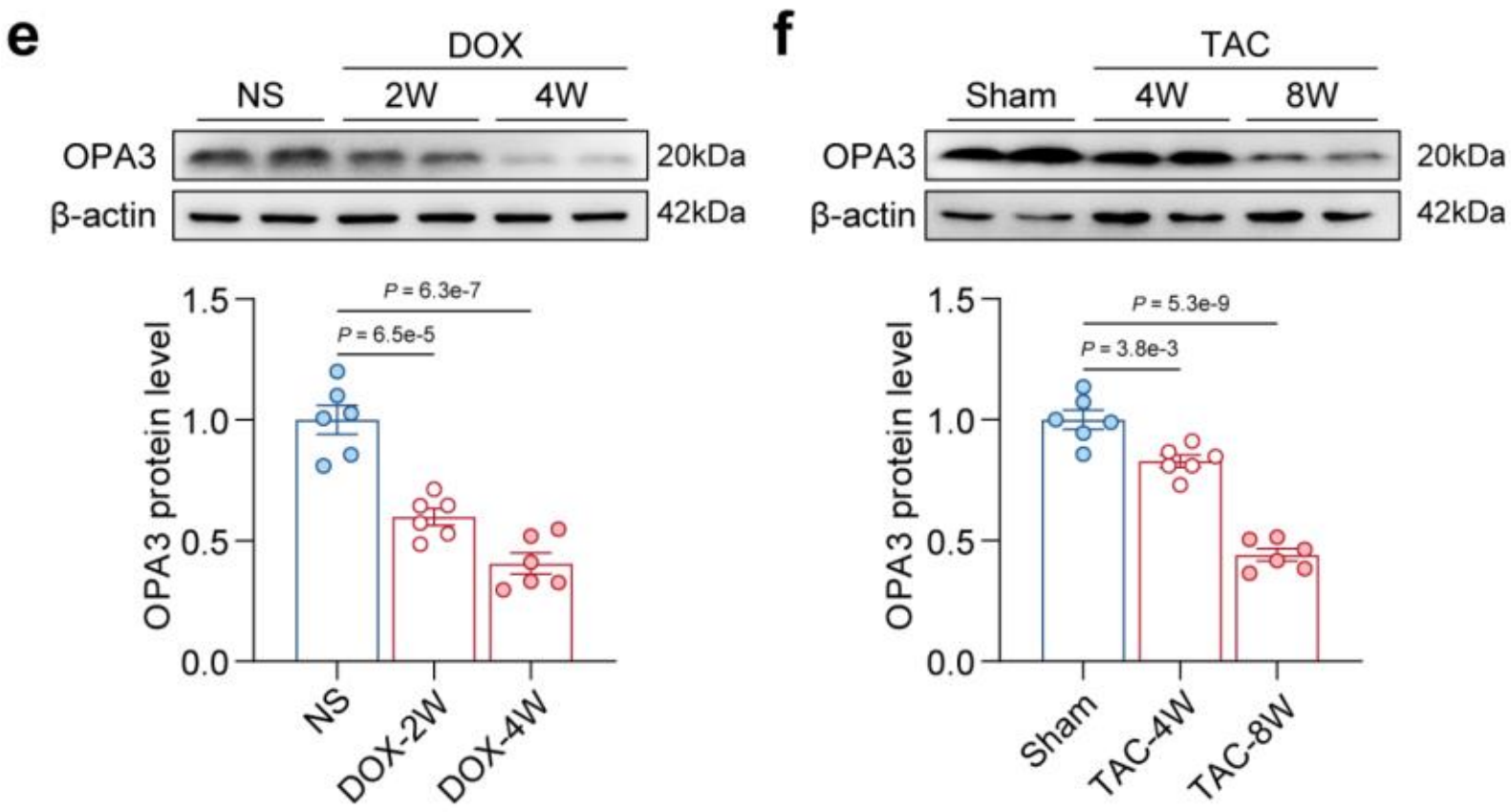

Fig. 1. Cardiomyocyte-derived OPA3 is downregulated in DOX-induced cardiomyopathy and TAC-induced HF.

e, **Top**, Representative western blots for OPA3 and β-actin in doxorubicin (DOX)-induced failing hearts and normal saline (NS) control heart tissues from mouse. **Bottom**, Quantification of protein expressions in indicated groups shown in Top (n=6 per group). Data were analyzed by one-way ANOVA followed by the Tukey post hoc test. **f**, **Top**, Representative western blots for OPA3 and β-actin in TAC-induced failing hearts and normal heart tissues (Sham) from mouse. **Bottom**, Quantification of protein expressions in indicated groups shown in Top (n=6 per group). Data were analyzed by one-way ANOVA followed by the Tukey post hoc test. HF indicates heart failure; OPA3, optic atrophy 3; TAC, transverse aortic constriction.

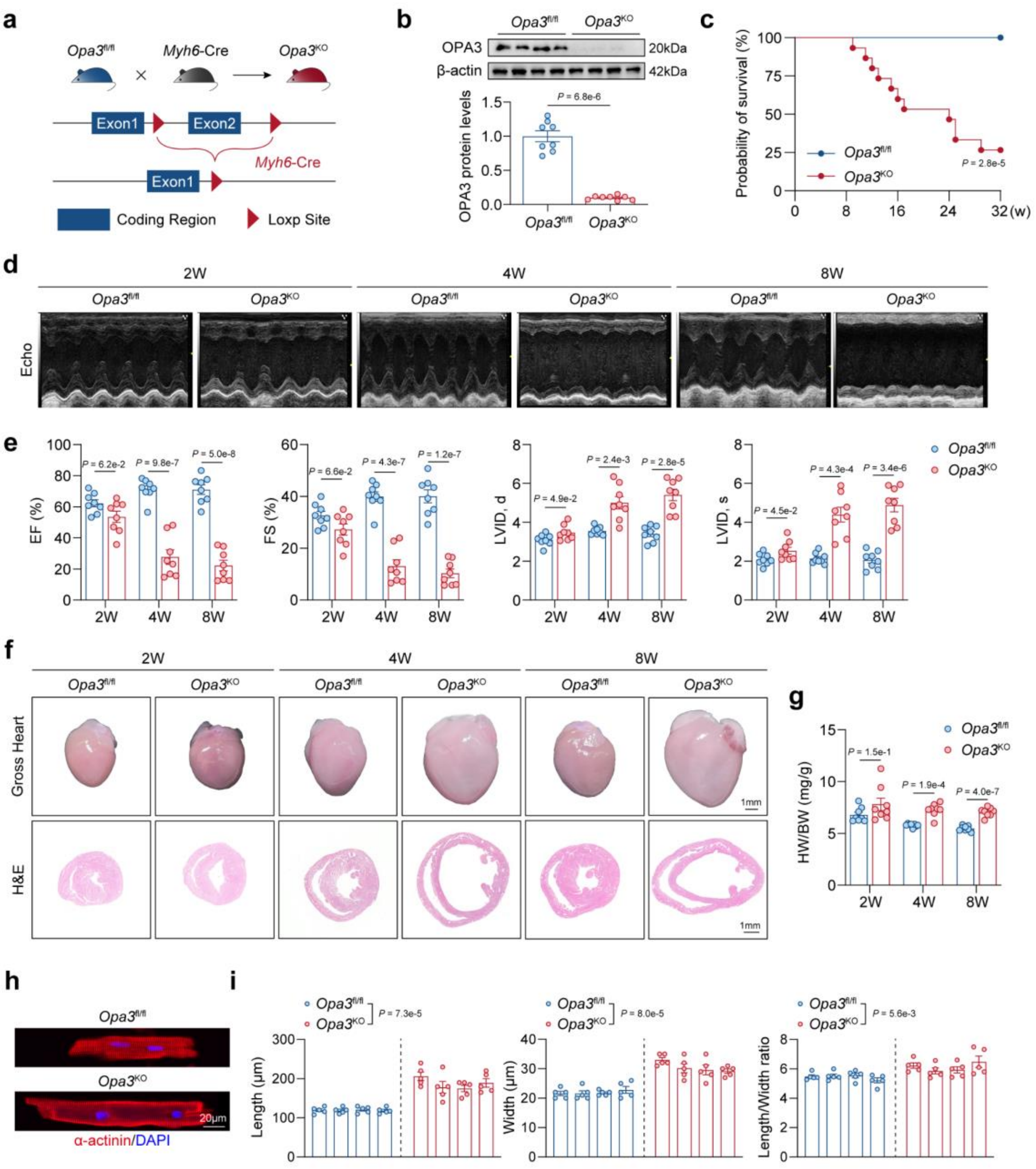

Fig. 2. Cardiomyocyte-specific deficiency of *Opa3* leads to progressive DCM and HF.

a, Schematic diagram illustrating the strategy of cardiomyocyte-specific *Opa3* knockout mice construction. **b**, **Top**, Representative western blots for OPA3 and β-actin in heart tissues from *Opa3*$^{fl/fl}$ and *Opa3*^{KO} mice. **Bottom**, Quantification of protein expressions in indicated groups shown in **Top** (n=8 per group). Data were analyzed by Welch's *t* test. **c**, Survival curves in indicated groups (n=15 per group). Survival data were analyzed by the Kaplan-Meier method and compared using log-rank test. **d** and **e**, Representative M-mode echocardiographic images (**d**) and corresponding measurements of EF, FS, LVID,d, and LVID,s (**e**) of *Opa3*$^{fl/fl}$ and *Opa3*^{KO} mice at 2 weeks, 4 weeks, and 8 weeks of age (n=8 per group). Data were analyzed by Student's *t* test (equal variances) or Welch's *t* test (unequal variances). **f**, **Top**, Representative gross images of hearts from *Opa3*$^{fl/fl}$ and *Opa3*^{KO} mice at indicated time points (scale bar, 1 mm). **Bottom**, Representative images of H&E-stained heart sections in indicated groups (scale bar, 1 mm). **g**, Ratios of heart weight to body weight (HW/BW) in indicated groups (n=8 per group).

Data were analyzed by Student's *t* test (equal variances) or Welch's *t* test (unequal variances). **h**, Representative immunofluorescence images of α-actinin in isolated AMCMs from *Opa3*$^{fl/fl}$ and *Opa3*^{KO} mice at 8 weeks of age (scale bar, 20 μm). **i**, Quantifications of cell length, width, and length/width ratio shown in (**h**) (5 cardiomyocytes per animal, 4 animals per group). Bars in the same color represent individual cells from different animals within the same group. Data were analyzed by nested *t* test. Data are expressed as mean ± SEM. AMCM indicates adult mouse cardiomyocyte; DCM, dilated cardiomyopathy; EF, ejection fraction; FS, fractional shortening; H&E, hematoxylin and eosin; HF, heart failure; LVID,d, internal dimension of the left ventricle in diastole; LVID,s, internal dimension of the left ventricle in systole; Opa3, optic atrophy 3.

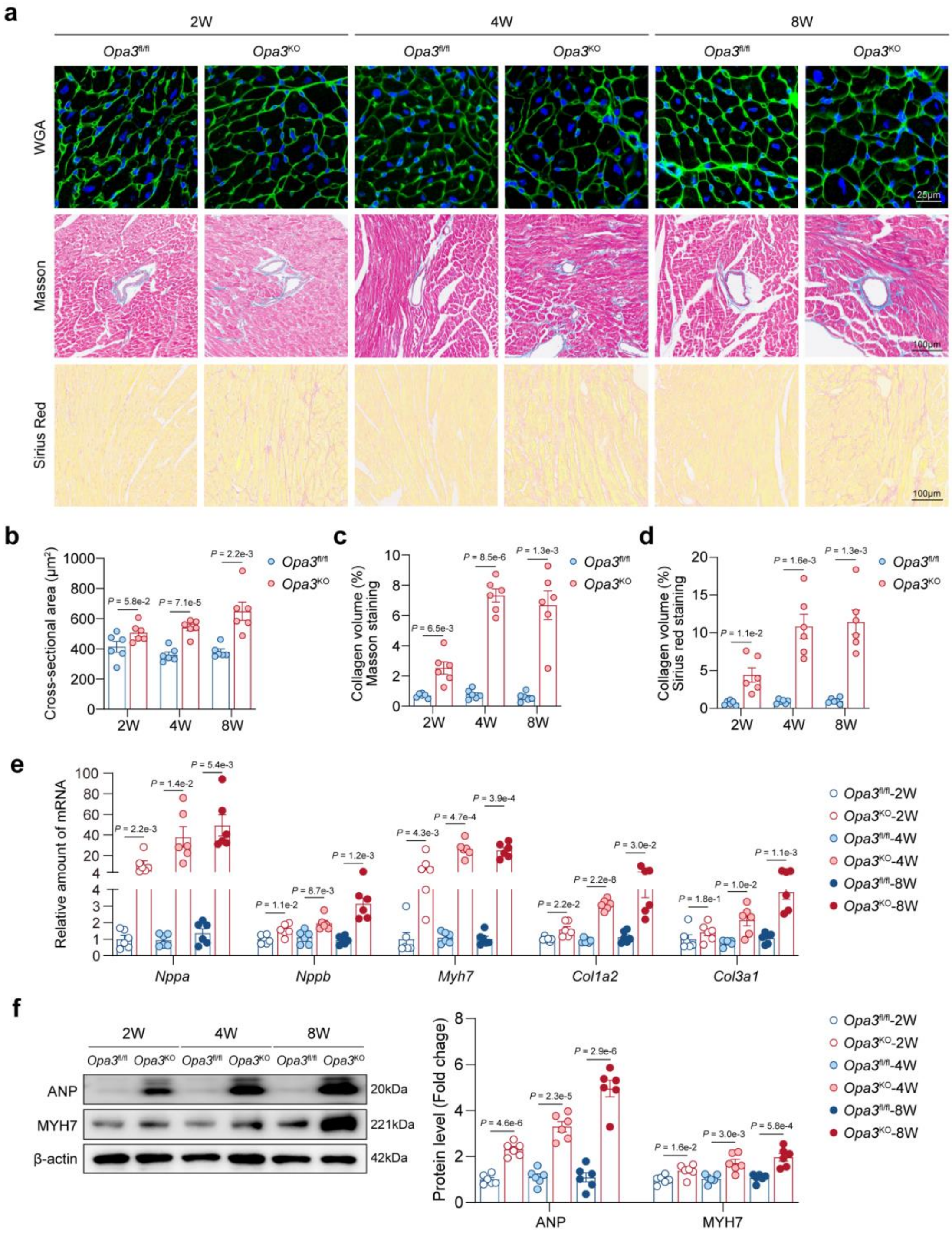

Supplementary Fig. 4. Cardiomyocyte-specific deficiency of *Opa3* exhibited typical histological characteristics of DCM.

a, Representative images of heart tissues stained with WGA, Masson trichrome, and Sirius red from *Opa3*$^{fl/fl}$ and *Opa3*^{KO} mice at 2 weeks, 4 weeks, and 8 weeks of age (scale bar, 25 μm for WGA; 100 μm for Masson trichrome and Sirius red). **b**, Quantification of mean cardiomyocyte cross-section area of WGA-stained sections in (**a**) (n=6 per group). Data were analyzed by Student's *t* test or Mann-Whitney *U* test. **c** and **d**, Quantification of fibrotic area (%) as determined by Masson trichome staining (**c**) (n=6 per group) and Sirius red staining of heart sections (**d**) presented in (**a**)

(n=6 per group). Data were analyzed by Welch's *t* test. **e**, Relative mRNA expression of *Nppa*, *Nppb*, *Myh7*, *Col1a2*, and *Col3a1* in the heart tissues from *Opa3*$^{fl/fl}$ and *Opa3*^{KO} mice at indicated time points (n=6 per group). Data were analyzed by Student's *t* test (equal variances), Welch's *t* test (unequal variances), or the Mann-Whitney *U* test. **f**, **Left**, Representative western blots for ANP, MYH7, and β-actin in the heart tissues from *Opa3*$^{fl/fl}$ and *Opa3*^{KO} mice at indicated time points. **Right**, Quantification of protein expressions in indicated groups shown in **Left** (n=6 per group). Data were analyzed by Student's *t* test. Data are expressed as mean ± SEM. Col1a2 indicates collagen type I alpha 2 chain; Col3a1, collagen type III alpha 1 chain; DCM, dilated cardiomyopathy; Myh7, myosin heavy chain 7; Nppa (Anp), natriuretic peptide type A; Nppb (Bnp), natriuretic peptide type B; Opa3, optic atrophy 3; WGA, wheat germ agglutinin.

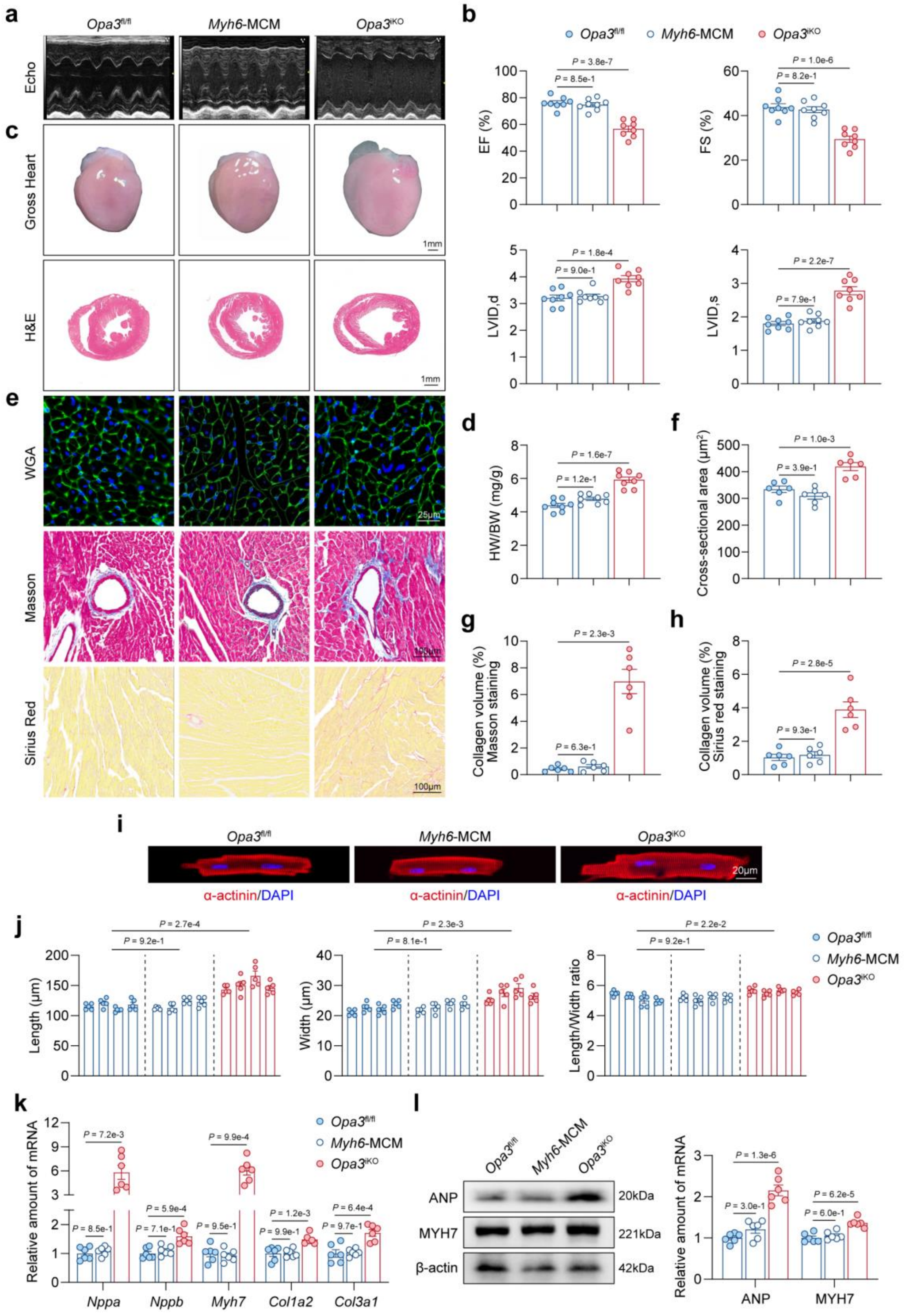

Supplementary Fig. 6. Adult cardiomyocyte-specific deficiency of *Opa3* leads to DCM.

a and **b**, Representative M-mode echocardiographic images (**a**) and corresponding measurements of EF, FS, LVID,d, and LVID,s (**b**) of *Opa3*$^{fl/fl}$, *Myh6*-MCM, and *Opa3*^{iKO} mice 2 weeks after five consecutive days of tamoxifen administration (n=8 per group). Data were

analyzed by one-way ANOVA followed by the Tukey post hoc test. **c**, **Top**, Representative gross images of hearts from indicated groups (scale bar, 1 mm). **Bottom**, Representative images of H&E-stained heart sections in indicated groups (scale bar, 1 mm). **d**, Ratios of heart weight to body weight (HW/BW) in indicated groups (n=8 per group). Data were analyzed by one-way ANOVA followed by the Tukey post hoc test. **e**, Representative images of heart tissues stained with WGA, Masson trichrome, and Sirius red from indicated groups (scale bar, 25 μm for WGA; 100 μm for Masson trichrome and Sirius red). **f**, Quantification of mean cardiomyocyte cross-section area of WGA-stained sections in (**e**) (n=6 per group). Data were analyzed by one-way ANOVA followed by the Tukey post hoc test. **g** and **h**, Quantification of fibrotic area (%) as determined by Masson trichome staining (**g**) (n=6 per group) and Sirius red staining of heart sections (**h**) presented in (**e**) (n=6 per group). Data were analyzed by one-way ANOVA followed by the Tukey post hoc test. **i**, Representative immunofluorescence images of α-actinin in isolated AMCMs from *Opa3*$^{fl/fl}$, *Myh6*-MCM, and *Opa3*^{iKO} 2 weeks after five consecutive days of tamoxifen administration (scale bar, 20 μm). **j**, Quantifications of cell length, width, and length/width ratio shown in (**i**) (5 cardiomyocytes per animal, 4 animals per group). Bars in the same color represent individual cells from different animals within the same group. Data were analyzed by nested one-way ANOVA followed by the Tukey post hoc test. **k**, Relative mRNA expression of *Nppa*, *Nppb*, *Myh7*, *Col1a2*, and *Col3a1* in the heart tissues from indicated groups (n=6 per group). Data were analyzed by one-way ANOVA followed by the Tukey post hoc test. **l**, **Top**, Representative western blot for ANP, MYH7, and β-actin in the heart tissues from indicated groups. **Bottom**, Quantification of protein expression in indicated groups shown in **Top** (n=6 per group). Data were analyzed by one-way ANOVA followed by the Tukey post hoc test. Data are expressed as mean ± SEM. Col1a2 indicates collagen type I alpha 2 chain; Col3a1, collagen type III alpha 1 chain; DCM, dilated cardiomyopathy; EF, ejection fraction; FS, fractional shortening; H&E, hematoxylin and eosin; LVID,d, internal dimension of the left ventricle in diastole; LVID,s, internal dimension of the left ventricle in systole; MCM, MerCreMer; Myh7, myosin heavy chain 7; Nppa (Anp), natriuretic peptide type A; Nppb (Bnp), natriuretic peptide type B; Opa3, optic atrophy 3; WGA, wheat germ agglutinin.

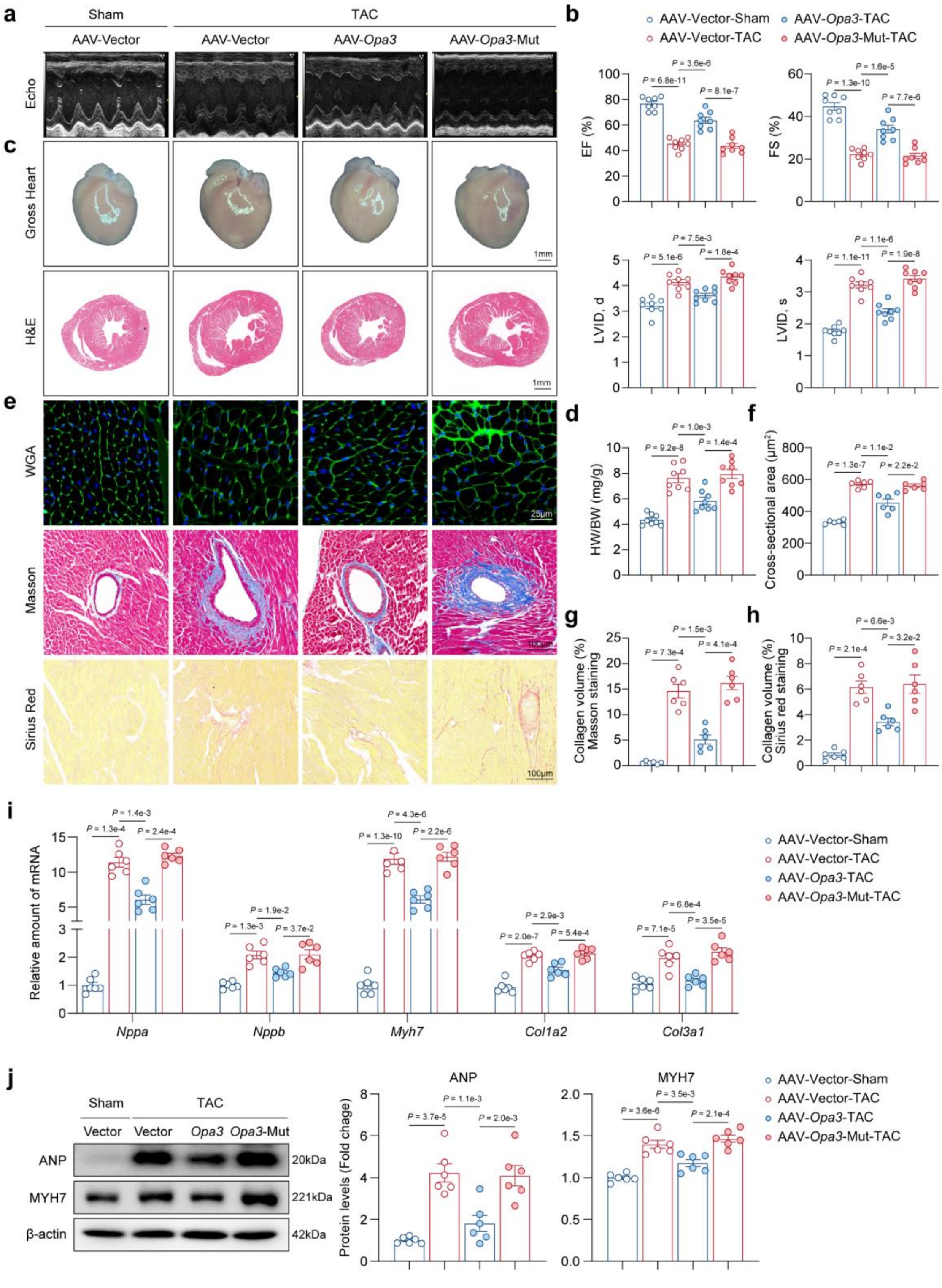

Fig. 8. OPA3 multimerization is required for its protective effect against TAC-induced HF and mitochondria-SR homeostasis.

a and **b**, Representative M-mode echocardiographic images (**a**) and corresponding measurements of EF, FS, LVID,d, and LVID,s (**b**) in *Opa3*$^{fl/fl}$ mice received AAV-Vector, AAV-*Opa3*, and AAV-*Opa3*-Mut at 8 weeks after sham or TAC surgery (n=8 per group). Data were analyzed by one-way ANOVA followed by the Tukey post hoc test. **c**, **Top**, Representative gross

images of hearts from indicated groups (scale bar, 1 mm). **Bottom**, Representative images of H&E–stained heart sections in indicated groups (scale bar, 1 mm). **d**, Ratios of heart weight to body weight (HW/BW) in indicated groups (n=8 per group). Data were analyzed by one-way ANOVA followed by the Tukey post hoc test. **e**, Representative images of heart tissues stained with WGA, Masson trichrome, and Sirius red from indicated groups (scale bar, 25 μm for WGA; 100 μm for Masson trichrome and Sirius red). **f**, Quantification of mean cardiomyocyte cross-section area of WGA-stained sections in (**e**) (n=6 per group). Data were analyzed by Welch's ANOVA test followed by a post-hoc analysis using the Tamhane T2 method. **g** and **h**, Quantification of fibrotic area (%) as determined by Masson trichome staining (**g**) (n=6 per group) and Sirius red staining of heart sections (**h**) presented in (**e**) (n=6 per group). Data were analyzed by Welch's ANOVA test followed by a post-hoc analysis using the Tamhane T2 method. **i**, Relative mRNA expression of *Nppa*, *Nppb*, *Myh7*, *Col1a2*, and *Col3a1* in the heart tissues from indicated groups (n=6 per group). Data were analyzed by one-way ANOVA followed by the Tukey post hoc test or Welch's ANOVA test followed by a post-hoc analysis using the Tamhane T2 method. **j**, **Left**, Representative western blot for ANP, MYH7, and β-actin in the heart tissues from indicated groups. **Right**, Quantification of protein expression in indicated groups shown in **Left** (n=6 per group). Data were analyzed by one-way ANOVA followed by the Tukey post hoc test. Data are expressed as mean ± SEM. AAV indicates adeno-associated viruses; Col1a2, collagen type I alpha 2 chain; Col3a1, collagen type III alpha 1 chain; EF, ejection fraction; FS, fractional shortening; H&E, hematoxylin and eosin; HF, heart failure; LVID,d, internal dimension of the left ventricle in diastole; LVID,s, internal dimension of the left ventricle in systole; Myh7, myosin heavy chain 7; Nppa (Anp), natriuretic peptide type A; Nppb (Bnp), natriuretic peptide type B; OPA3, optic atrophy 3; SR, sarcoplasmic reticulum; TAC, transverse aortic constriction; WGA, wheat germ agglutinin.

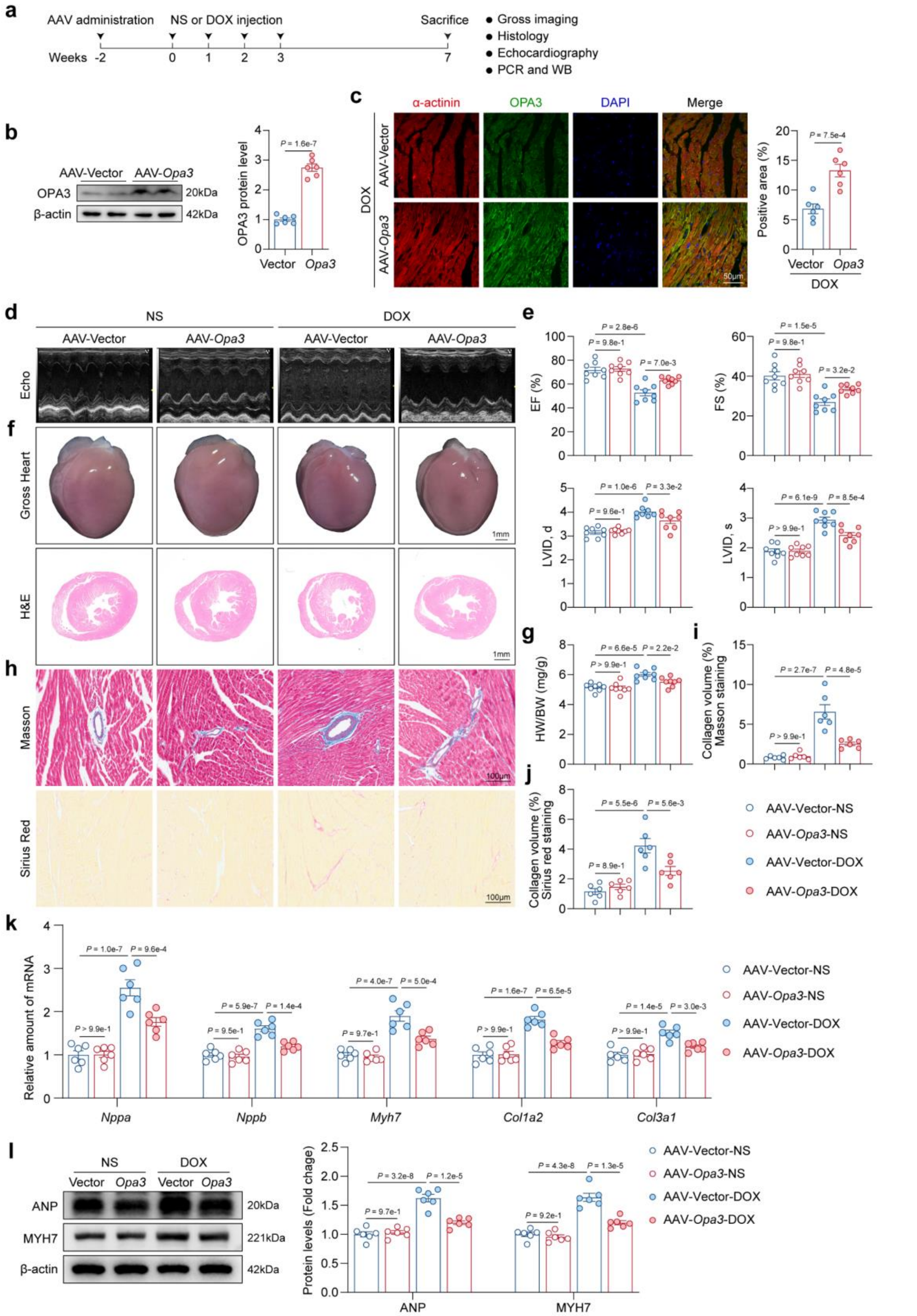

Supplementary Fig. 8. Cardiomyocyte-specific overexpression of Opa3 ameliorates DOX-induced cardiomyopathy.

a, Schematic diagram illustrating the timeline for AAV administration and DOX induction. **b**, **Left**, Representative western blots for OPA3 and β-actin in *Opa3*$^{fl/fl}$ mice treated with AAV-

Vector or AAV-*Opa3*. **Right**, Quantification of protein expressions in indicated groups shown in **Left** (n=6 per group). Data were analyzed by Student's *t* test. **c**, **Left**, Representative images of the colocalization of OPA3 (green) and α-actinin (red) in heart sections (scale bars, 50 μm). Nuclei were counterstained with DAPI (blue). **Right**, Quantification of OPA3 expressions in indicated groups shown in **Left** (n=6 per group). Data were analyzed by Student's *t* test. **d** and **e**, Representative M-mode echocardiographic images (**d**) and corresponding measurements of EF, FS, LVID,d, and LVID,s (**e**) in indicated groups (n=8 per group). Data were analyzed by one-way ANOVA followed by the Tukey post hoc test. **f**, **Top**, Representative gross images of hearts from indicated groups (scale bar, 1 mm). **Bottom**, Representative images of H&E-stained heart sections in indicated groups (scale bar, 1 mm). **g**, Ratios of heart weight to body weight (HW/BW) in indicated groups (n=8 per group). Data were analyzed by one-way ANOVA followed by the Tukey post hoc test. **h**, Representative images of heart tissues stained with Masson trichrome and Sirius red from indicated groups (scale bar, 100 μm). **i** and **j**, Quantification of fibrotic area (%) as determined by Masson trichome staining (**i**) (n=6 per group) and Sirius red staining of heart sections (**j**) presented in (**G**) (n=6 per group). Data were analyzed by one-way ANOVA followed by the Tukey post hoc test. **k**, Relative mRNA expression of *Nppa*, *Nppb*, *Myh7*, *Col1a2*, and *Col3a1* in the heart tissues from indicated groups (n=6 per group). Data were analyzed by one-way ANOVA followed by the Tukey post hoc test. **l**, **Left**, Representative western blot for ANP, MYH7, and β-actin in the heart tissues from indicated groups. **Right**, Quantification of protein expression in indicated groups shown in **Left** (n=6 per group). Data were analyzed by one-way ANOVA followed by the Tukey post hoc test. Data are expressed as mean ± SEM. AAV indicates adeno-associated viruses; Col1a2, collagen type I alpha 2 chain; Col3a1, collagen type III alpha 1 chain; DOX, doxorubicin; EF, ejection fraction; FS, fractional shortening; H&E, hematoxylin and eosin; LVID,d, internal dimension of the left ventricle in diastole; LVID,s, internal dimension of the left ventricle in systole; Myh7, myosin heavy chain 7; Nppa (Anp), natriuretic peptide type A; Nppb (Bnp), natriuretic peptide type B; Opa3, optic atrophy 3.

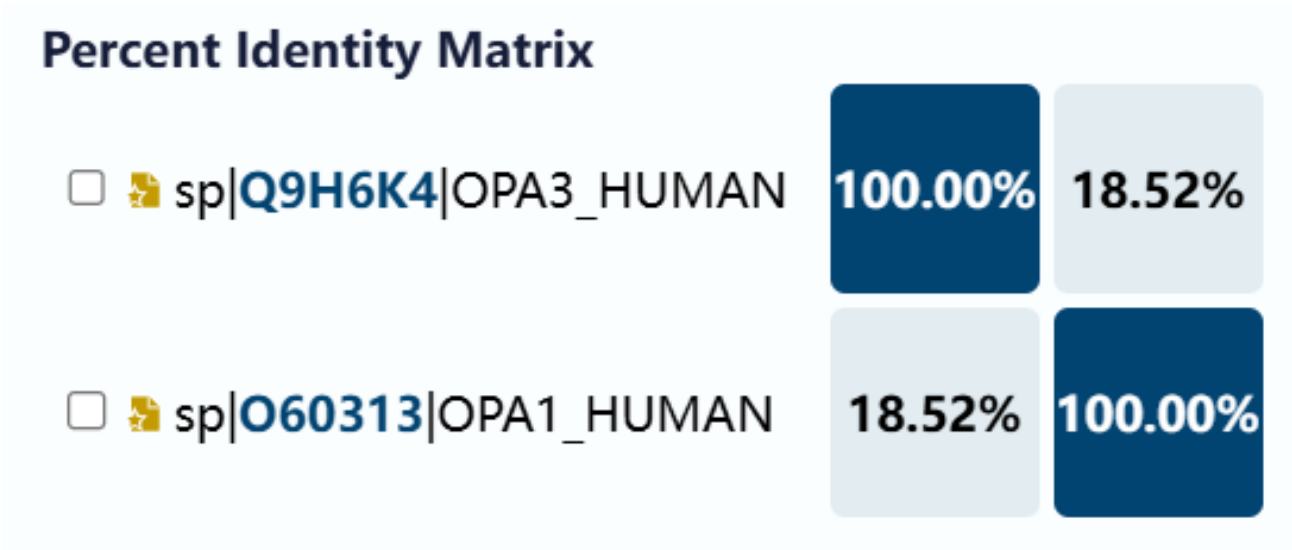

Fig. R1. Percent identity matrix of human OPA1 and OPA3 proteins from the UniProt database.

The matrix displays the sequence identity between human OPA1 (UniProt ID: O60313) and OPA3 (UniProt ID: Q9H6K4). OPA3 and OPA1 share 18.52% sequence identity, indicating limited sequence homology between the two proteins.

Percent Identity Matrix

sp|Q505D7|OPA3_MOUSE	100.00%	18.52%
sp|P58281|OPA1_MOUSE	18.52%	100.00%

Fig. R2. Percent identity matrix of mouse OPA1 and OPA3 proteins from the UniProt database.

The matrix displays the sequence identity between mouse OPA1 (UniProt ID: P58281) and OPA3 (UniProt ID: Q505D7). OPA3 and OPA1 share 18.52% sequence identity, indicating limited sequence homology between the two proteins.

References:

1. Horiuchi, N. et al. Detection of the nonsense mutation of OPA3 gene in Holstein Friesian cattle with dilated cardiomyopathy in Japan. *J. Vet. Med. Sci.* **77**, 1281-1283 (2015).

2. Directly consequent to the consideration above, the data presented do not allow the authors to state that "our findings suggest that targeting OPA3 may represent an effective therapeutic strategy for the management of DCM and subsequent HF.". The data only show that overexpression of OPA3 only very marginally improve cardiac function in very specific models of cardiomyopathy. The improvement after anthracycline administration appears to be statistically significant, but is quite modest (Extended Data Fig 8). More relevant, doxorubicin cardiomyopathy is primarily driven by mitochondrial dysfunction and is far from being representative of the vast majority anthracycline administration of DCM cases in patients, of which at least 50% (and probably many more) are genetically determined. Similar considerations also apply to using decompensation after TAC as a model for DCM (Figure 8). Should OPA3 gene delivery in more specific DCM animal models, or during HF in chronic ischemic conditions, be effective, this would be much more impactful for potential translation.

Response:

Thank you for these insightful comments. We agree that our current data, mainly derived from TAC- and doxorubicin-induced heart failure models, provide preliminary support for the therapeutic potential of OPA3, and are not sufficient to represent DCM cases in patients. Accordingly, the previous statement "targeting OPA3 may represent an effective therapeutic strategy for the management of DCM and subsequent HF" indeed overstated the translational implications, and we have revised the manuscript to more accurately reflect the scope of our findings, now emphasizing that our findings

"targeting OPA3 may represent a potential therapeutic strategy for the management of HF." Lines 64–65.

Importantly, the central aim of our study was not to model all etiologies of DCM but to delineate the mechanistic role of OPA3 in cardiac function, and to determine whether restoring OPA3 expression confers measurable functional benefit under cardiac stress. Thus, combined with your suggestion above, we extended our investigation to an ischemia/reperfusion (I/R) model, which is well established in our laboratory [1, 2] to further strengthen the translational implication of our findings. Our preliminary results showed that OPA3 overexpression significantly reduced the infarction area and improved cardiac function in 48 h post IR (Fig. R3). Regarding the modest degree of functional rescue observed upon AAV9-mediated OPA3 overexpression, we agree that this likely reflects the severity and specific pathophysiology of the doxorubicin-induced heart failure model, differences in disease progression across models, or potentially heterogeneous cardiomyocyte transduction efficiency as you suggested below. Nonetheless, the fact that restoration of OPA3 levels leads to measurable improvements in cardiac function supports its biological relevance. In addition, future studies will be directed toward evaluating OPA3 gene manipulation in genetic DCM models, including transgenic mice harboring defined pathogenic mutations associated with inherited DCM. In summary, we have softened our translational conclusions and provided new evidence from an I/R model to bolster the relevance of OPA3. We believe these observations address your valid concerns by presenting a more balanced interpretation and a stronger, multi-model foundation for future research.

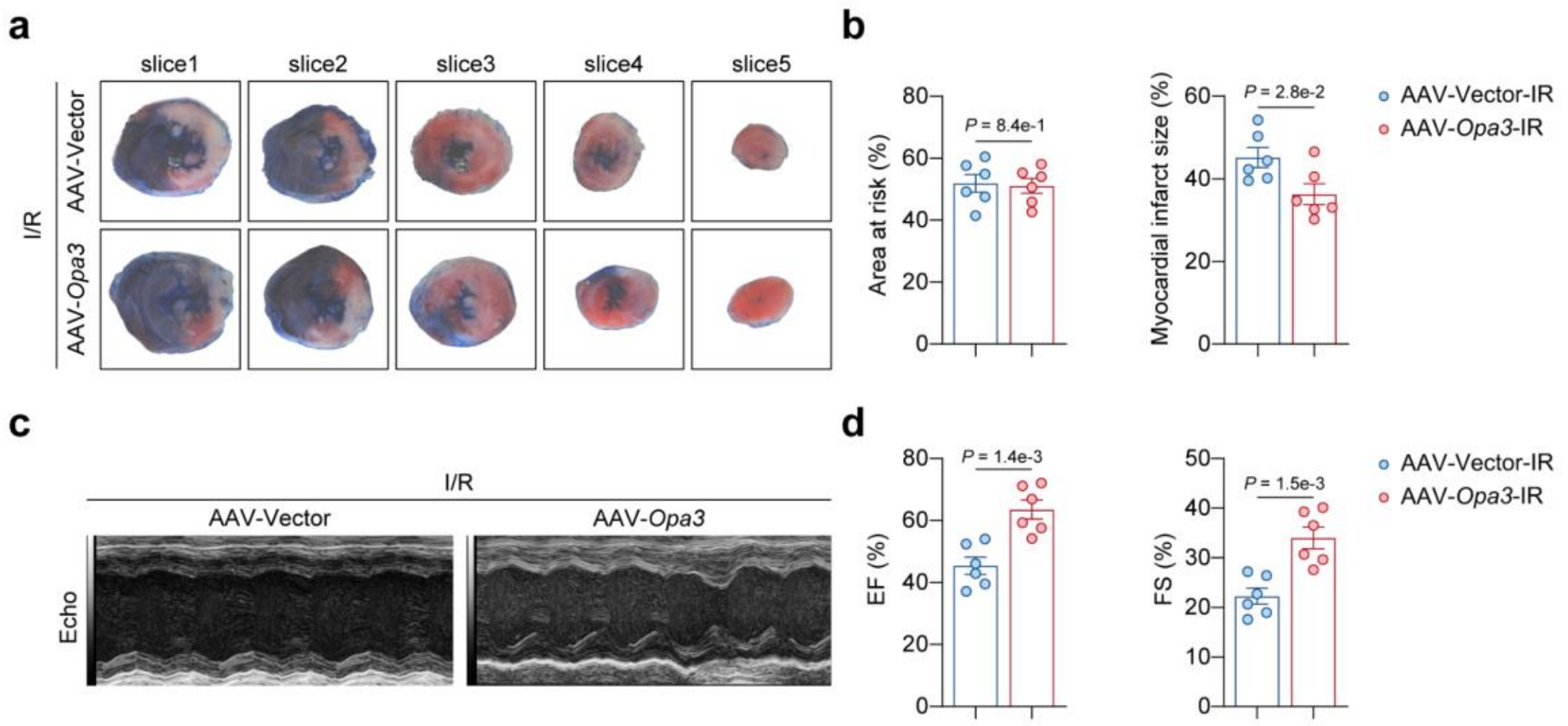

Fig. R3. Cardiomyocyte-specific overexpression of *Opa3* ameliorates I/R-induced HF.

a, Evans blue/TTC double staining of heart slices. **b**, Quantitative analysis of the area at risk and myocardial infarct size (n=6 per group). Data were analyzed by Student's *t* test. **c** and **d**, Representative M-mode echocardiographic images (**c**) and corresponding measurements of EF and FS in indicated groups (n=8 per group). Data were analyzed by Student's *t* test. Data are

presented as mean ± SEM. EF indicates ejection fraction; FS, fractional shortening; HF, heart failure; Opa3, optic atrophy 3.

References:

1. Zhao, Y. et al. Disruption of Circadian Rhythms by Shift Work Exacerbates Reperfusion Injury in Myocardial Infarction. *J Am Coll Cardiol* **79**, 2097-2115 (2022).

2. Qin, Z. et al. The nuclear receptor co-repressor 1 is a novel cardioprotective factor against acute myocardial ischemia-reperfusion injury. *J Mol Cell Cardiol* **166**, 50-62 (2022).

3. The authors use AAV9 vectors to express the OPA3 transgene under the control of the troponin T promoter in vivo. Protein overexpression is shown after gene transfer, but it is unclear what the efficiency of transduction is in terms of number of cardiomyocytes overexpressing the transgene. This is an important point, as OPA3 overexpression is expected to compensate mitochondrial function only in the transduced cells. In other terms, the authors should show whether expression is very high in few cells, or more moderately high in a large number of cells. Poor efficiency of transduction (namely, few transduced cardiomyocytes) could also explain why the functional effect of overexpressing OPA3 is relatively modest.

Response:

We thank you for raising this important point. In the revised manuscript, we performed immunofluorescence staining in both TAC- and DOX-induced HF model to show the OPA3 overexpression efficacy and spatial distribution within cardiomyocytes by co-staining of OPA3 with α-actinin in the heart. Our results show that OPA3 expression exhibited a heterogeneous distribution across cardiomyocytes rather than uniform expression (Fig. R4 and Supplementary Fig. 8c). Thus, these data clarify the extent and limitations of *in vivo* OPA3 gene transfer and provide a plausible explanation for the partial functional rescue observed in our models.

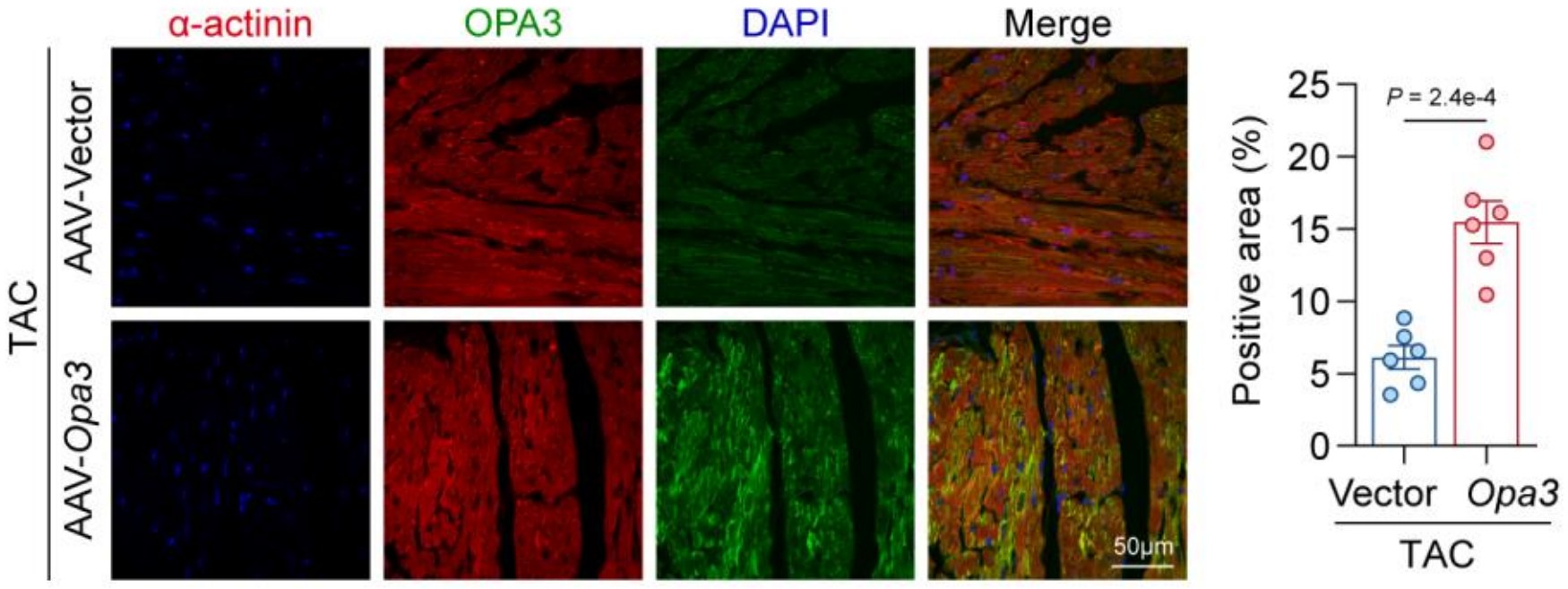

Fig. R4. Cardiomyocyte-specific overexpression of Opa3 ameliorates TAC-induced HF.

Left, Representative images of the colocalization of OPA3 (green) and α-actinin (red) in heart sections (scale bars, 50 μm). Nuclei were counterstained with DAPI (blue). **Right**, Quantification of OPA3 expressions in indicated groups shown in **Left** (n=6 per group). Data were analyzed by Student's *t* test. Data are expressed as mean ± SEM.

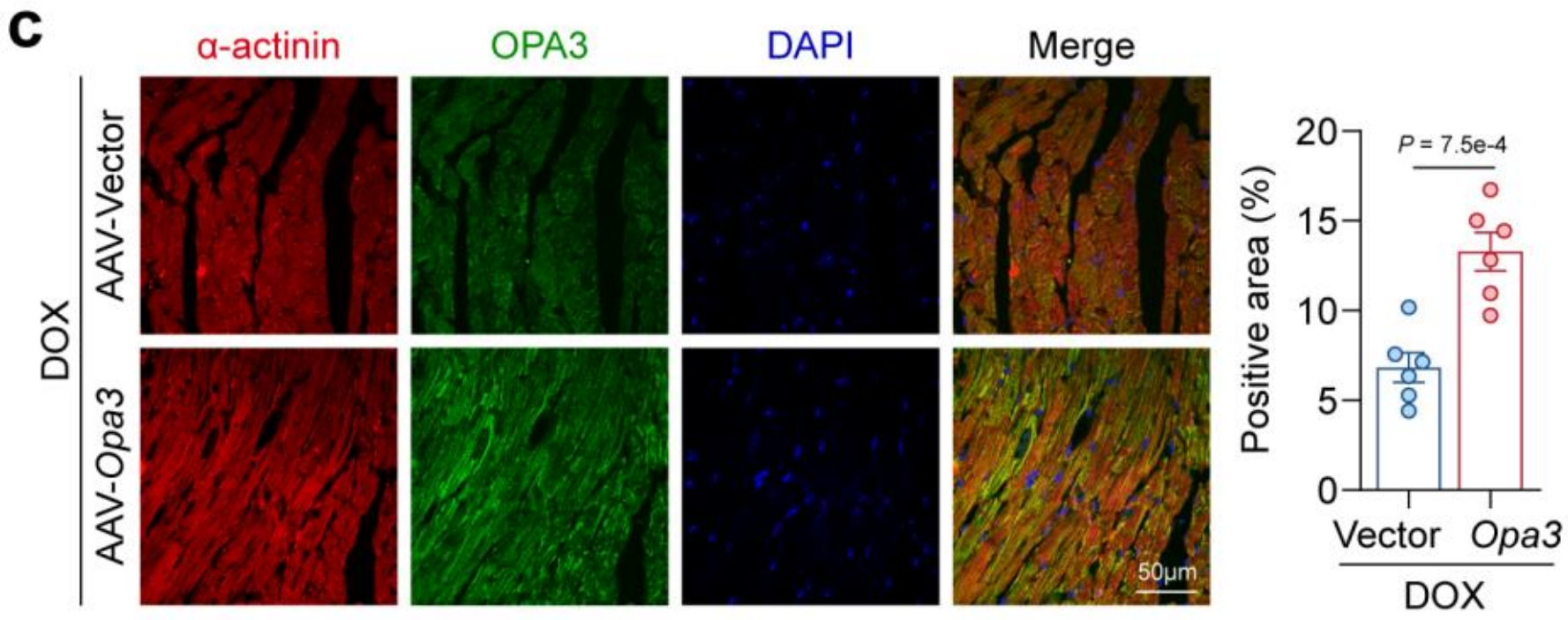

Supplementary Fig. 8. Cardiomyocyte-specific overexpression of Opa3 ameliorates DOX-induced cardiomyopathy.

c, **Left**, Representative images of the colocalization of OPA3 (green) and α-actinin (red) in heart sections (scale bars, 50 μm). Nuclei were counterstained with DAPI (blue). **Right**, Quantification of OPA3 expressions in indicated groups shown in **Left** (n=6 per group). Data were analyzed by Student's *t* test. Data are expressed as mean ± SEM.

4. Why did the authors use adenovirus (instead of AAV6) vectors to overexpress or knock down OPA3 in vitro?

Response:

Thank you for this question. In our study, adenoviral vectors were intentionally selected for *in vitro* experiments for several reasons. First, adenoviruses provide much higher transduction efficiency and a rapid onset of gene expression, typically within 24–48 h, which is essential for dissecting the direct and cell-autonomous effects of OPA3 manipulation in isolated cardiomyocytes. In contrast, AAV6 requires a substantially longer period to achieve robust expression, which makes it difficult to distinguish primary effects of OPA3 deficiency from secondary adaptations that occur during prolonged culture after transduction. Second, our *in vitro* experiments were designed to evaluate acute changes in mitochondrial function and calcium handling, independent of remodeling processes associated with heart failure. Adenoviral delivery enables precise temporal control and minimizes the confounding influence of chronic stress responses that may arise during the delayed expression phase of AAV-based vectors.

Detailed Response to the Reviewer #2

General comments:

This is a very complete and well-structured manuscript in which the authors explore the role of OPA3 in cardiac function. Using a broad range of models and techniques, they present several key findings: OPA3 is located in the outer mitochondrial membrane, its expression is reduced in human and animal models of heart failure, and its deletion leads to heart failure, a phenotype that is reversed by OPA3 overexpression. Molecular analyses show that OPA3 interacts with phospholamban (PLN), predominantly in its pentameric form, promoting its phosphorylation and enhancing SERCA2a function. OPA3 forms tetramers, which appear more functionally active. The authors identify specific amino acids involved in both tetramerization and PLN interaction. Additionally, they demonstrate that OPA3 contributes to mitochondrial function, ATP production, and interacts with mitofusins and MAMs, suggesting a role in ER–mitochondria communication.

Response:

We thank you for reviewing our manuscript and for your suggestions. We have carefully considered your feedback and made every effort to address these points thoroughly. Our detailed responses are provided below.

Major suggestions:

1. The extensive dataset convincingly supports a central role for OPA3 in mitochondrial function. However, the observed alterations in calcium handling could result either from direct effects via PLN interaction or indirectly from the heart failure phenotype due to mitochondrial dysfunction and reduced ATP production. A discussion on whether the calcium handling changes are primary to OPA3 deletion or secondary to the HF phenotype it induces would strengthen the manuscript.

Response:

Thanks for this insightful comment. To address whether the calcium handling changes are primary to OPA3 deletion or secondary to the HF phenotype, we have added additional description in the **Discussion** section as follows: "Defects in Ca^{2+} handling proteins are common features of HF.[51] Here, we showed that the cardiomyocyte-specific *Opa3* ablation led to impaired Ca^{2+} handling. To determine whether these defects represent a primary consequence of OPA3 loss or a secondary effect of the HF phenotype, we performed acute knockdown of OPA3 in isolated AMCMs from *Opa3*$^{fl/fl}$

mice in the absence of systemic hemodynamic stress. This approach was sufficient to alter Ca^{2+} transients and SERCA2a activity, indicating that Ca^{2+} dysregulation arises directly from OPA3 deficiency and occurs prior to the development of overt heart failure.”. You can also refer to Lines 819–826.

Minor suggestions:

1. Figure 4: Please clarify whether the Y-axis of the Ca^{2+} transients represents a fluorescence ratio.

Response:

The Y-axis of the Ca^{2+} transients represents the fluorescence ratio of F340/F380, which is a standard ratiometric measurement used to reflect intracellular calcium concentrations. The corresponding figures have been revised accordingly for clarity (Fig.4a and Supplementary Fig. 12a, 24d).

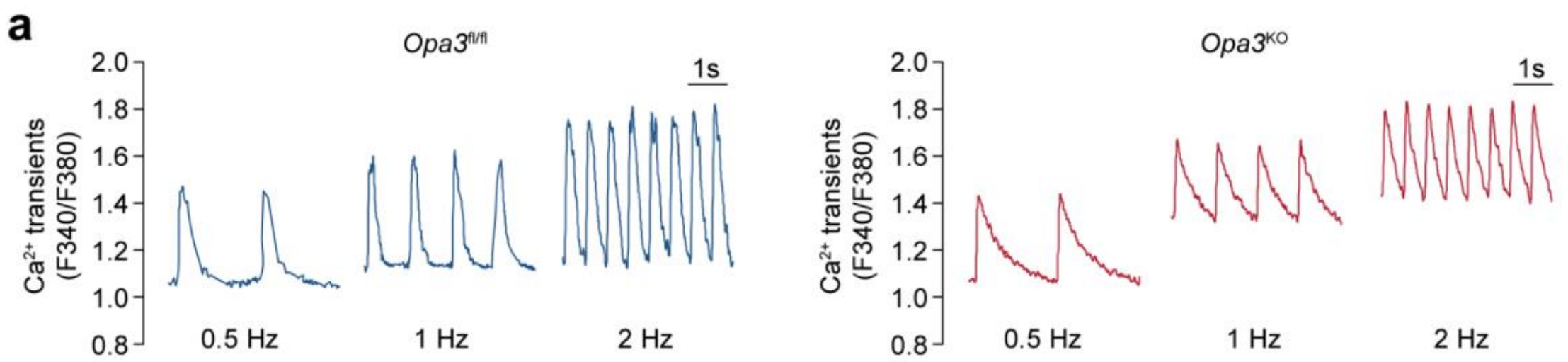

Fig. 4. The absence of *Opa3* in cardiomyocytes leads to Ca^{2+} handling deficiency.

a, Representative traces of calcium transients in isolated AMCMs from *Opa3*$^{fl/fl}$ and *Opa3*^{KO} mice at 8 weeks of age.

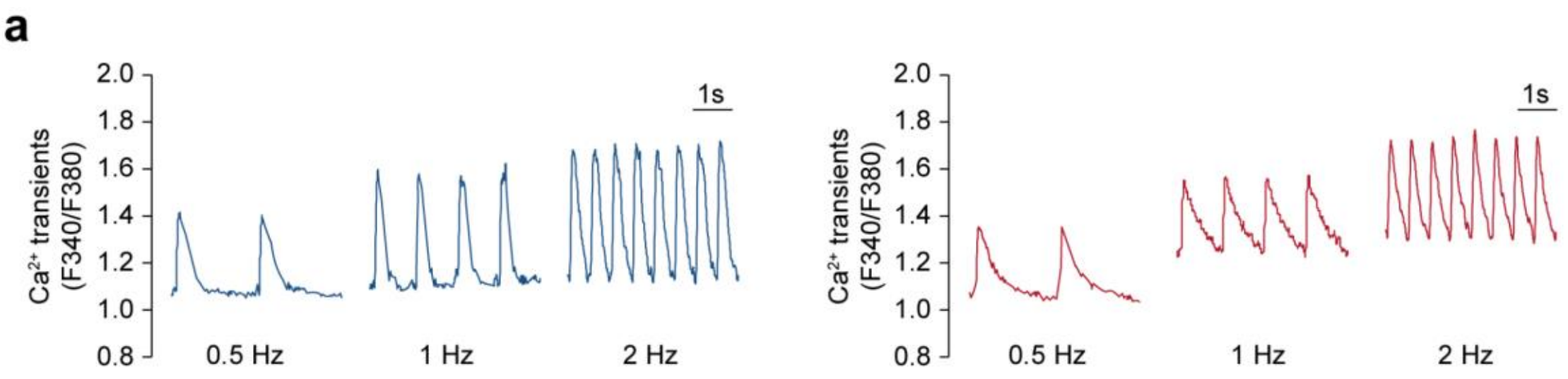

Supplementary Fig. 12. The knockdown of *Opa3* in cardiomyocytes leads to Ca^{2+} handling deficiency.

a, Representative traces of calcium transients in isolated AMCMs infected with adenovirus encoding scramble (Scramble) or shRNA targeting *Opa3* (sh*Opa3*) for 48 h.

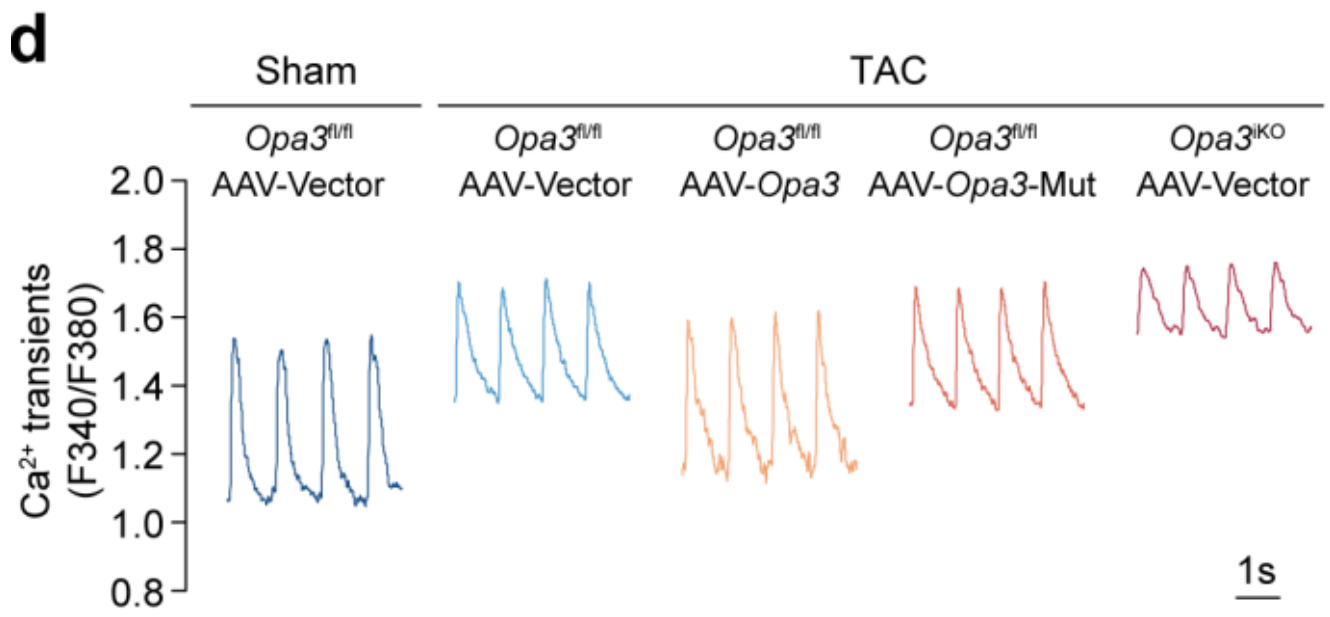

Supplementary Fig. 24. OPA3 multimerization is required for its protective effect against TAC-induced Ca^{2+} handling deficiency.

d, Representative traces of calcium transients in isolated AMCMs from indicated groups.

2. Figures 4 and 6: Are different bars in the same color from different animals? Since nested statistical tests are used, displaying a single column per group (possibly with dot color variations to denote individual animals) may make the figures clearer and more concise.

Response:

We are sorry for the confusion. Different bars in the same color represent individual cells derived from different animals within the same experimental group. To avoid potential visual confusion and to ensure accessibility for readers with color vision deficiency, we chose not to use dot color variations to denote individual animals within a single column (Fig. R5). Instead, we have added a clarification in the figure legend as follows: "Bars in the same color represent individual cells from different animals within the same group".

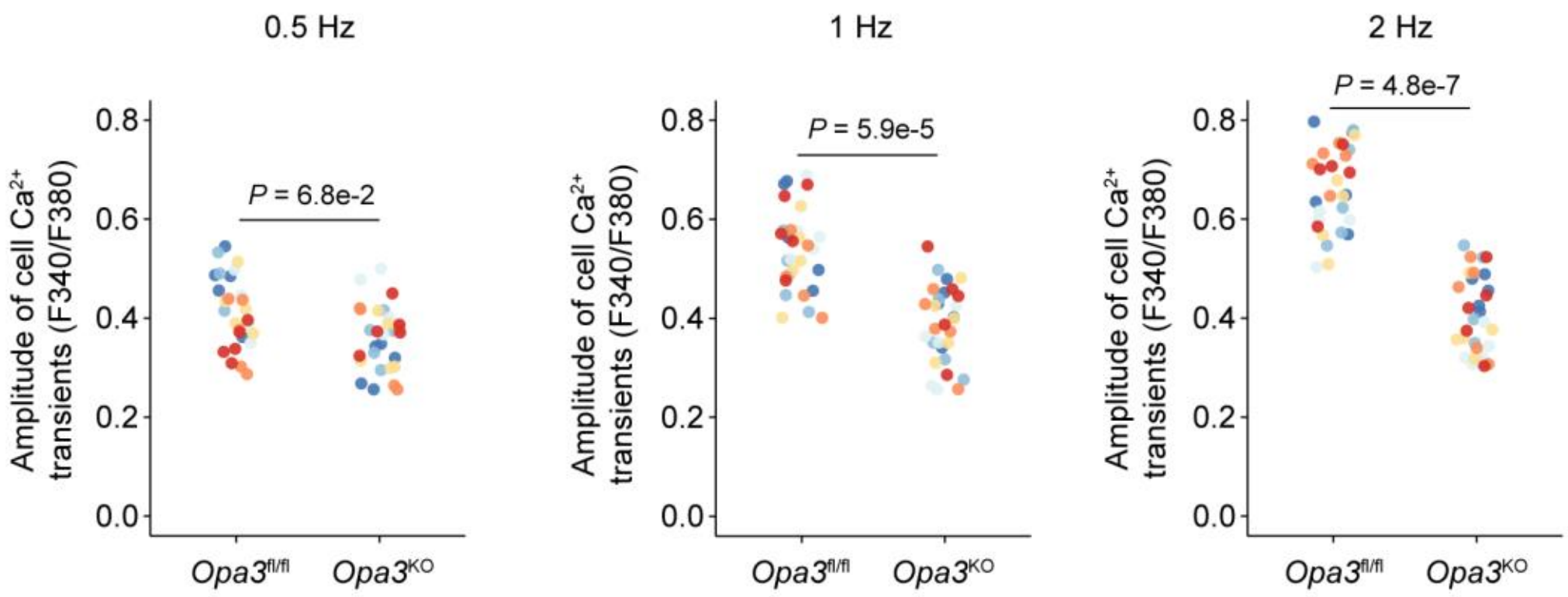

Fig. R5. The absence of *Opa3* in cardiomyocytes leads to Ca^{2+} handling deficiency.

Quantification of amplitude of Ca^{2+} transients (5 cardiomyocytes per animal, 6 animals per group). The same color in each column represents individual cells from the same animal, whereas different colors represent distinct animals within the same group. Data were analyzed by nested *t* test.

3. Mitochondrial fragmentation: You suggest that fragmentation upon OPA3 deletion is due to disrupted SR interaction. However, since OPA3 is located throughout the outer mitochondrial membrane, and PLN/SR are more localized, this may not fully explain the fragmentation. Could OPA3 also play a role in fusion-fission dynamics?

Response:

Thank you for this insightful comment. As suggested, we further examined mitochondrial dynamics following *Opa3* deletion. Our data show that loss of Opa3 significantly increases mitochondrial fission, as evidenced by upregulated FIS1 and DRP1, and reduces mitochondrial fusion, as indicated by decreased OPA1 and MFN2 expression (Fig. R6). These results support that the marked mitochondrial fragmentation observed in *Opa3*-deficient hearts is also associated with an imbalance in the fusion-fission dynamics. In addition, previous studies have demonstrated that mitochondria-ER/SR contact sites serve as important platforms for regulating mitochondrial dynamics.[1–3] Consistent with these findings, our discovery of an interaction between the mitochondrial protein OPA3 and the SR-resident protein PLN provides new mechanistic support that OPA3 may participate in mitochondrial dynamics partly through its role at mitochondria-SR contact sites. However, we acknowledge that OPA3 is broadly distributed along the outer mitochondrial membrane, and its effects on mitochondrial dynamics may not be exclusively mediated through SR interactions. It is therefore plausible that OPA3 also regulates mitochondrial fusion-fission dynamics through SR-independent mechanisms. Further studies will be required to delineate these possibilities and to define the precise molecular pathways by which OPA3 governs mitochondrial dynamics.

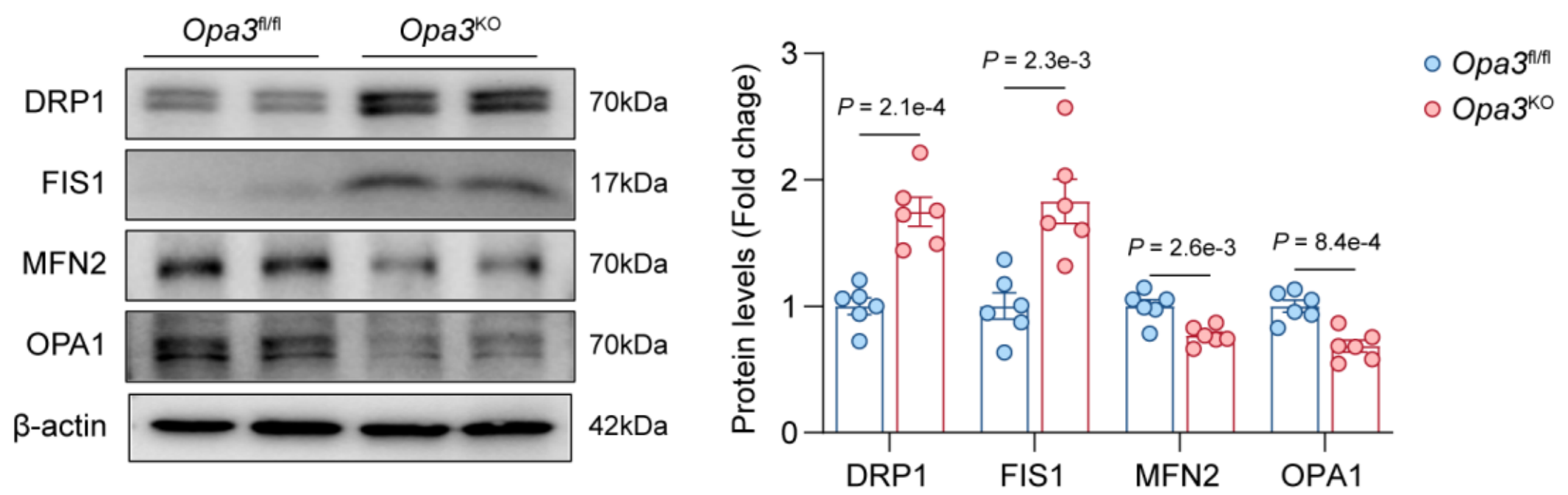

Fig. R6. The role of OPA3 in mitochondrial dynamics.

Left, Representative western blot for DRP1, FIS1, MFN2, OPA1 and β-actin in the heart tissues from indicated groups. **Right**, Quantification of protein expression in indicated groups shown in **Left** (n=6 per group). Data were analyzed by Student's *t* test. Data are expressed as mean ± SEM.

References:

1. Chen, C. et al. Mitochondria-associated endoplasmic reticulum membranes and myocardial ischemia: from molecular mechanisms to therapeutic strategies. *J. Transl. Med*. **23**, 277 (2025).

2. Mao, H., Chen, W., Chen, L. & Li, L. Potential role of mitochondria-associated endoplasmic reticulum membrane proteins in diseases. *Biochem. Pharmacol*. **199**, 115011 (2022).

3. Jiang, R.-Q., Li, Q.-Q. & Sheng, R. Mitochondria associated ER membranes and cerebral ischemia: Molecular mechanisms and therapeutic strategies. *Pharmacol. Res*. **191**, 106761 (2023).

4. OPA3/PLN stoichiometry: utilizing your data, can you estimate the proportion of total OPA3 that interacts with PLN or vice-versa?

Response:

Thank you for this important question. Based on our image-based quantification, we measured the colocalized OPA3-PLN fluorescence signal and normalized it to the total OPA3 fluorescence intensity within cardiomyocytes. This analysis revealed that approximately 64.89% of total OPA3 is engaged in interaction with PLN *in situ* (Supplementary Fig. 15a). This has been added to the revised Results section (Lines 633–637).

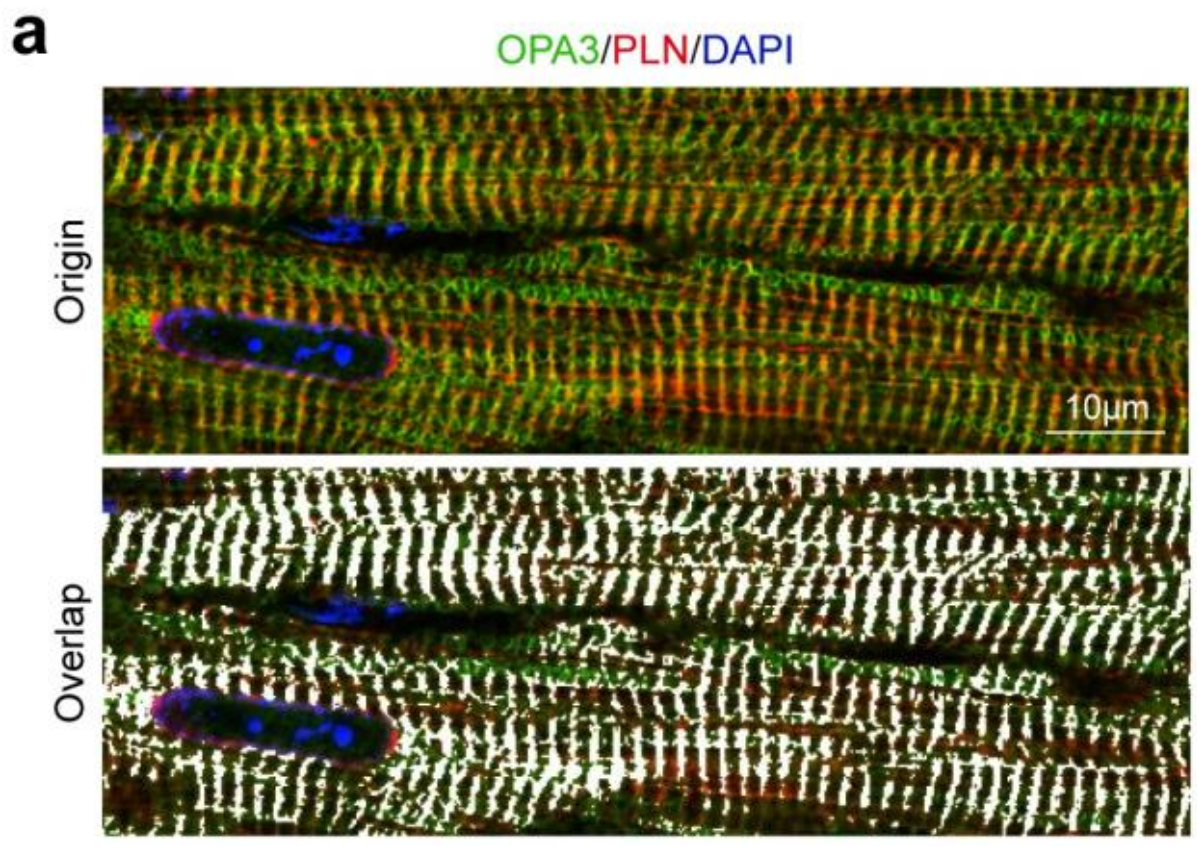

Supplementary Fig. 15. Lack of interaction between OPA3 and SERCA2a.

a, Representative images of the colocalization of OPA3 (green) and PLN (red) in heart sections (scale bars, 10 μm). Nuclei were counterstained with DAPI (blue). The upper panels show the original fluorescence images, and the lower panels display the colocalized OPA3-PLN regions (white) identified using *FIJI* software. Colocalized OPA3-PLN signals were quantified and normalized to total OPA3 fluorescence intensity to estimate the fraction of OPA3 associated

with PLN *in situ*. OPA3 indicates optic atrophy 3; SERCA, sarcoplasmic/endoplasmic reticulum Ca^{2+}-ATPase.

5. Lines 786–787: The sentence "Opa3 deficiency caused defects in SR Ca^{2+} handling, contraction, and mitochondrial function, resulting in progressive DCM..." may suggest a sequence of events that is not entirely demonstrated.

Response:

Thanks. To avoid overinterpretation, we have revised the sentence to: "cardiomyocyte-specific *Opa3* deficiency caused defects in SR Ca^{2+} handling and mitochondrial function, which may contribute to the development of HF." to avoid overstatement." (Lines 788–790).

6. Have you measured the phosphorylation levels of other proteins? The decrease in PLN phosphorylation in OPA3 KO might be a consequence of heart failure. Is phosphorylation of other proteins also reduced? Could the diminished effect of db-cAMP be due to altered PKA abundance or to reduced ATP levels?

Response:

Thank you for this insightful comment. We have performed a series of complementary experiments to fully address your concerns and presented as detailed below.

We first analyzed the phosphorylation levels of additional PKA downstream targets, including RyR2 and TnI in *Opa3*-deficient AMCMs. Our results showed that phosphorylation levels of RyR2 and TnI were also reduced upon *Opa3* deletion (Fig. R7a). To evaluate whether the reduced PLN phosphorylation after *Opa3* ablation reflects a secondary consequence of heart failure, we have previously isolated NMCMs and subjected them to *Opa3* knockdown, which does not involve heart failure, and showed that db-cAMP failed to effectively induce phosphorylation of PLN (Fig. 5k). Consistently, similar defects in PKA downstream target phosphorylation were observed in AMCMs isolated from $Opa3^{iKO}$ one week after tamoxifen administration, a time point at which no significant changes in heart failure markers were detected, including ANP and MYH7 (Fig. R7b). These findings further support the notion that the diminished phosphorylation of PKA downstream targets upon *Opa3* loss reflects an OPA3-dependent regulatory defect rather than a secondary effect of established heart failure.

To exclude the possibility that the diminished effect of db-cAMP was due to altered PKA abundance, we next examined total PKA protein levels. Notably, total PKA abundance remained unchanged in *Opa3* knockdown NMCMs (Fig. R7d), indicating

that OPA3 loss does not affect PKA expression but instead impairs PKA signaling at a functional level. To further dissect whether diminished PLN phosphorylation is attributed to reduced ATP content, isolated NMCMs were supplemented pyruvate to enhance ATP production (Fig. R7c).[1] Our results showed that pyruvate supplementation restored the RyR2 and TnI phosphorylation levels. However, the total PKA abundance and PLN phosphorylation remained unchanged upon pyruvate supplementation in *Opa3* knockdown NMCMs (Fig. R7d). These results suggest that the impairment of PLN phosphorylation is not solely due to a global energy deficit but may involve a more specific mechanism.

Taken together, our data reveal a previously unrecognized role for OPA3 in the regulation of the PKA signaling pathway. While this function may be partially linked to cellular energy metabolism and ATP production, PLN phosphorylation appears to be particularly dependent on OPA3, pointing to a specific regulatory axis that warrants further investigation.

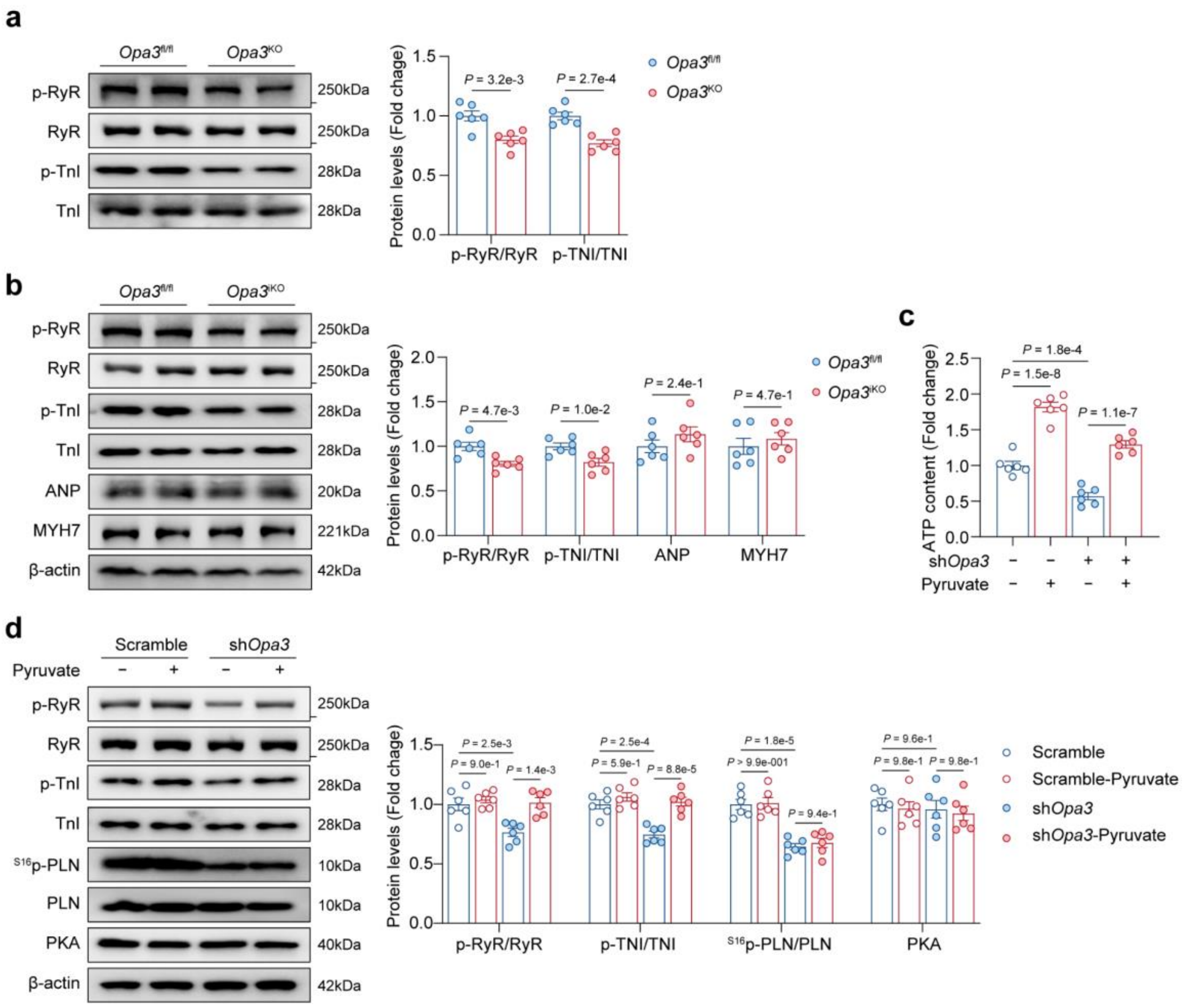

Fig. R7 The role of OPA3 in PKA signaling pathway.

a, **Left**, Representative western blots in PE-treated (50 nM for 48 h) AMCMs isolated from *Opa3*$^{fl/fl}$ and *Opa3*^{KO} mice. Right, Quantification of protein expressions in indicated groups shown in Left (n=6 per group). Data were analyzed by Student's t test. **b**, **Left**, Representative

western blots in PE-treated (50 nM for 48 h) AMCMs isolated from *Opa3*$^{fl/fl}$ and *Opa3*^{iKO} mice. *Opa3*$^{fl/fl}$ and *Opa3*^{iKO} mice were treated with tamoxifen for one week. Right, Quantification of protein expressions in indicated groups shown in Left (n=6 per group). Data were analyzed by Student's t test. **c**, ATP content was determined by ATP Assay Kit (n=6 per group). Data were analyzed by Student's *t* test. **d**, Representative western blots in indicated groups. NMCMs were infected with adenovirus encoding scramble (Scramble) or shRNA targeting *Opa3* (sh*Opa3*) for 48 h. NMCMs were then supplemented with pyruvate (20 mM) for 30 min and treated with db-cAMP (10 µM). Data were analyzed by one-way ANOVA followed by the Tukey post hoc test.

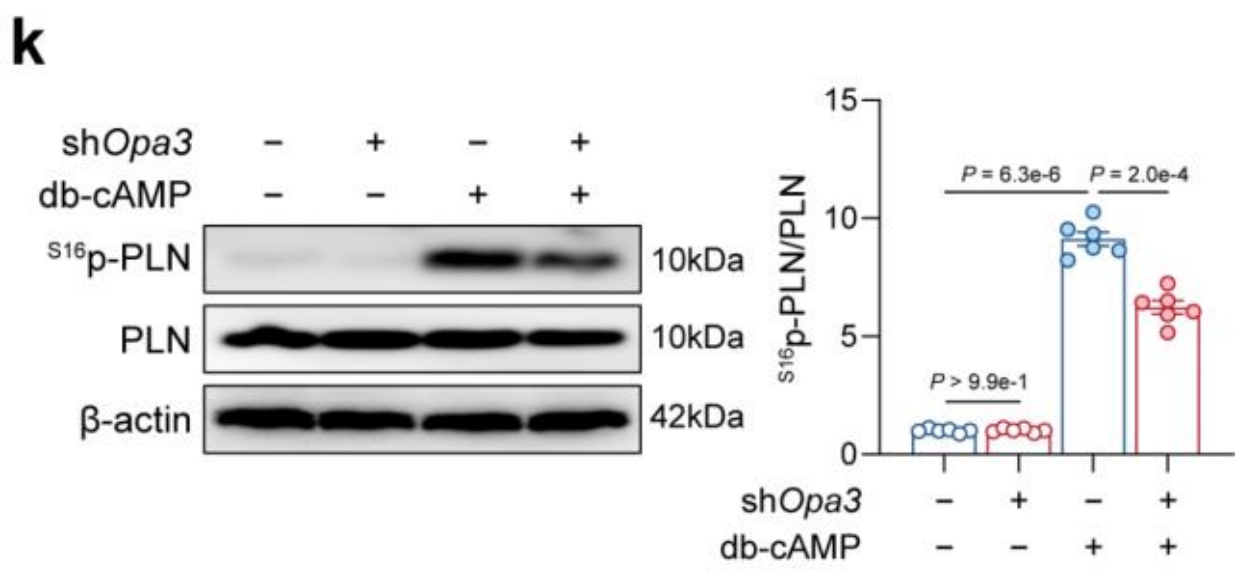

Fig. 5. OPA3 interacts with SR-located protein phospholamban (PLN).

k, **Left**, Representative western blots for PLN and its phosphorylation levels in db-cAMP-treated (10 µM) NMCMs that were infected with adenovirus encoding scramble (Scramble) or shRNA targeting *Opa3* (sh*Opa3*) for 48 h. **Right**, Quantification of protein expressions in indicated groups shown in **Left** (n=6 per group). Data were analyzed by Welch's ANOVA test followed by a post-hoc analysis using the Tamhane T2 method.

References:

1. Hasenfuss, G. et al. Influence of pyruvate on contractile performance and Ca(2+) cycling in isolated failing human myocardium. *Circulation* **105**, 194-199 (2002).

Detailed Response to the Reviewer #3

General comments:

The manuscript by Geng and colleagues describes characterization of the role of Optic atrophy 3 (OPA3) in the heart. Much of the data is derived from a mouse model in which exon 2 of the Opa3 gene is flanked by loxP sites; crossing with mre-cre-mer/Myh6 transgenic line allows cardiomyocyte specific knockout. The manuscript is well written and the extensive data generally well described.

This is a strong report which has a comprehensive data set and a number of conclusions potentially important for heart function in normal and disease settings. The complete knockout of OPA3 in cardiomyocytes has profound consequences on cardiac function suggesting a potentially important role for this protein; this approach is not subtle however, and proving its role as a driver, rather than a secondary player, in cardiomyopathy mechanisms needs further models. OPA3 is notably reduced in human cardiomyopathy samples (presumably end stage); also, interestingly c.3x fold overexpression in cardiomyocytes has significant positive effects in a DOX heart failure, suggesting therapeutic potential – these data are confined to a supplementary figure (8), the authors might consider moving this to a main figure. The mechanistic studies involving PLN are novel and of great interest, and the interaction with PLN broadly consistent with the calcium handling data measured in single cardiomyocytes.

Response:

We sincerely thank you for the thoughtful and encouraging comments. We greatly appreciate your recognition of the novelty of our study and the potential significance for understanding cardiac physiology and disease. Regarding the suggestion to move the DOX-induced heart failure data from the Supplementary Information to the main figures, we carefully considered this point during revision. Based on the collective comments from the reviewers, we have currently retained the DOX model data in the Supplementary Information. If the editors or reviewers feel that inclusion of the DOX model would strengthen the overall logic and impact of the revised manuscript, we would be very happy to reorganize the figures accordingly and move these results into the main text.

Specific points:

1. It is postulated that OPA3 multimers/tetramers and it is stated that "Multimers are sometimes linked by covalent bonds, such as disulfide bonds" as justification for the production of the series of cys-to-ala mutants. Surely it would have been straightforward to test via non-denaturing electrophoresis whether the multimers

were sensitive to reducing agent (2-mercaptoethanol, dithiothreitol etc.) as a preliminary experiment? This should be carried out and included.

__Response:__

Thank you for raising this important methodological point. We agree that testing the sensitivity of OPA3 multimers to reducing agents is a logical and direct preliminary experiment to validate our hypothesis regarding disulfide bond involvement. Accordingly, we performed non-denaturing gel electrophoresis in the presence or absence of dithiothreitol. As shown in Supplementary Fig. 20b, we observed that OPA3 exhibited a significantly reduced ability to form multimers when treated with dithiothreitol, indicating that OPA3 multimers are stabilized by disulfide bonds. We believe this additional experiment strengthens the foundation of our mechanistic study and thank you for the suggestion. These results have been added to the revised **Results** section. Please refer to the Lines 718–721.

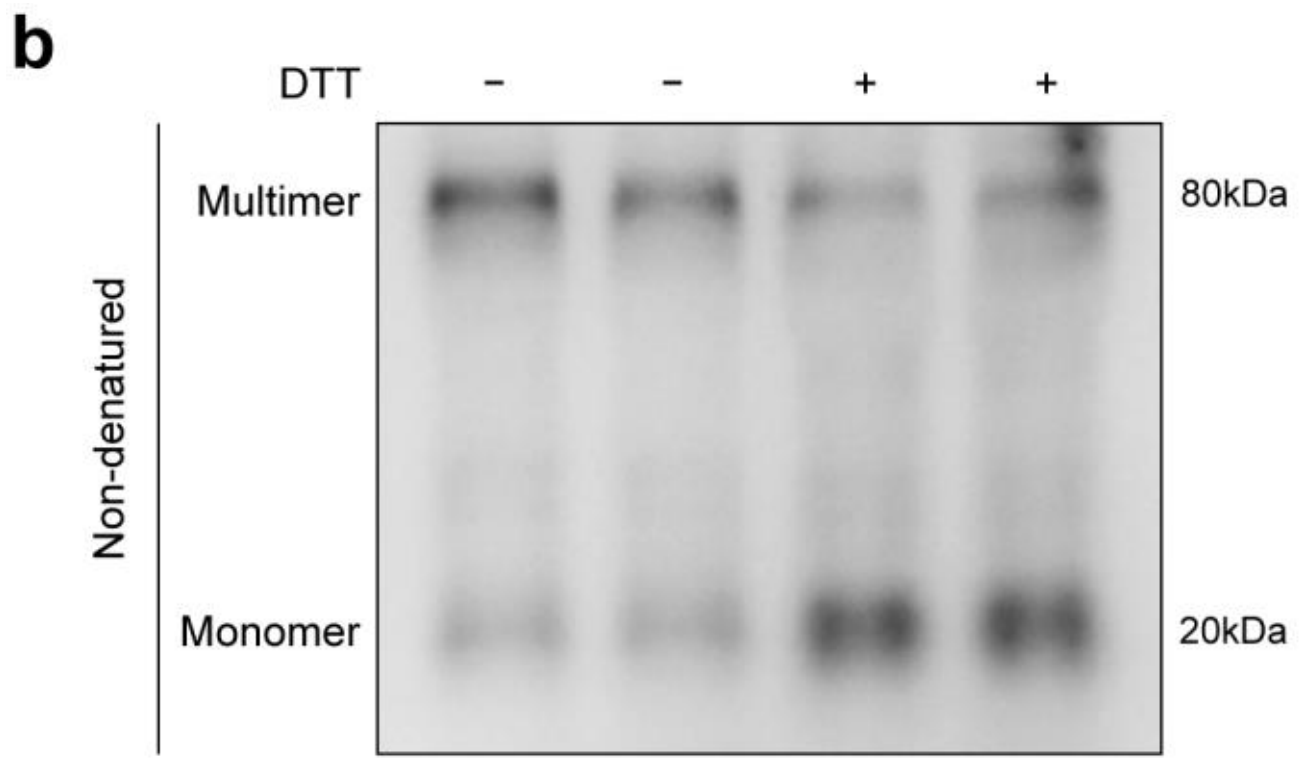

Supplementary Fig. 20. The multimerization of OPA3.

b, Representative western blots of OPA3 monomer and multimer in native protein extraction from NMCMs. Protein extracts were analyzed in the presence or absence of DTT. DTT indicates dithiothreitol; NMCM, neonatal mouse cardiomyocyte; OPA3, optic atrophy 3.

2. The subcellular localization of OPA3 is dealt with over 2 figures, 5g and 7, with the result being that the description is rather fragmented. For example, it would help if the regions in 5g where there is OPA3 staining but no PLN were described (these are presumably mitochondria); similarly, the SR should be identified in Fig 6b. Also, it is stated that "Immunoelectron microscopy demonstrated that OPA3 was mainly located in mitochondria in adult hearts (Fig. 6a)."; however, I don't think Fig6a is clear and it is difficult to draw that conclusion solely from the given figure.

__Response:__

Thank you for these critical points regarding the clarity and presentation of our

subcellular localization data. In Fig. 5, our primary focus was to illustrate the spatial relationship between OPA3 and PLN; To provide a coherent narrative, we have consolidated the description of OPA3 localization in the revised **Results** section as you suggested. We now explicitly describe the regions in Fig. 5g where OPA3 signals do not overlap with PLN as putative mitochondrial localization (Lines 636–637).

As also suggested, we attempted to identify the SR in original Fig. 6b (revised Fig. 6a) to better illustrate the distribution of OPA3 between mitochondria and the SR. However, due to technical limitations, we were unable to simultaneously visualize OPA3, mitochondria, and SR markers in adult or neonatal cardiomyocytes. In particular, transfection efficiency of the SR-EGFP probe mRNA was consistently low in both adult and neonatal cardiomyocytes in our preliminary experiments, precluding reliable co-localization analysis. To overcome this limitation, we therefore employed H9C2 cells, in which OPA3 distribution within both mitochondria and the SR could be more clearly demonstrated (Supplementary Fig. 16d). These results are now described in the revised **Results** section (Line 655–674).

Regarding the original Fig. 6a, we replaced the previous approach with cryo-immunogold electron microscopy, with optimized antibody concentrations to enhance signal specificity and spatial resolution. Using this method, we observed that OPA3 is predominantly localized to mitochondria in adult mouse hearts (revised Supplementary Fig. 16a). In addition, to provide additional evidence of OPA3 localization, we performed the co-staining experiments in H9C2 cells, which revealed a clearer and more characteristic mitochondrial pattern that co-localizes with OPA3 (Supplementary Fig. 16c). Moreover, evidence from study in non-cardiac cells consistently demonstrates that OPA3 resides in the MOM,[1] which provides additional support for our localization findings.

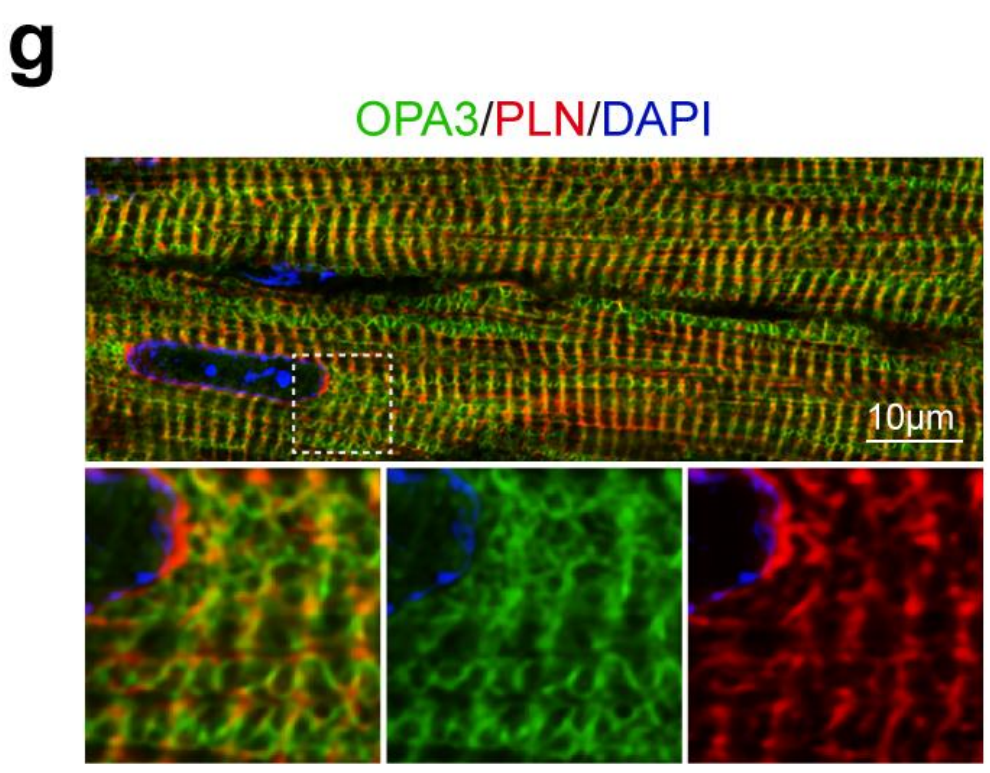

Fig. 5. OPA3 interacts with SR-located protein phospholamban (PLN).

g, Representative images of the colocalization of OPA3 (green) and PLN (red) in heart sections (scale bars, 10 μm). Nuclei were counterstained with DAPI (blue). OPA3, optic atrophy 3; PE, phenylephrine; PLN, phospholamban; SR, sarcoplasmic reticulum.

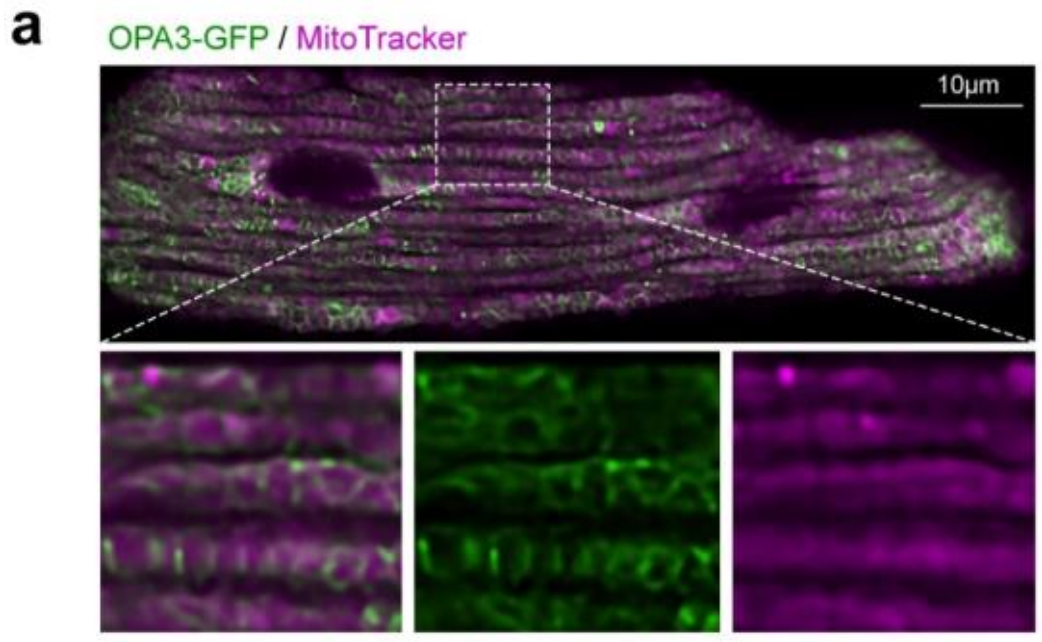

Fig. 6. OPA3 predominantly localizes to the outer mitochondrial membrane and regulates mitochondria-SR communication.

a, Representative images of MitoTraker staining in isolated AMCMs from *Opa3*$^{fl/fl}$ mice after infected with adenovirus encoding OPA3-GFP fusion protein for 48 h (scale bar, 10 µm).

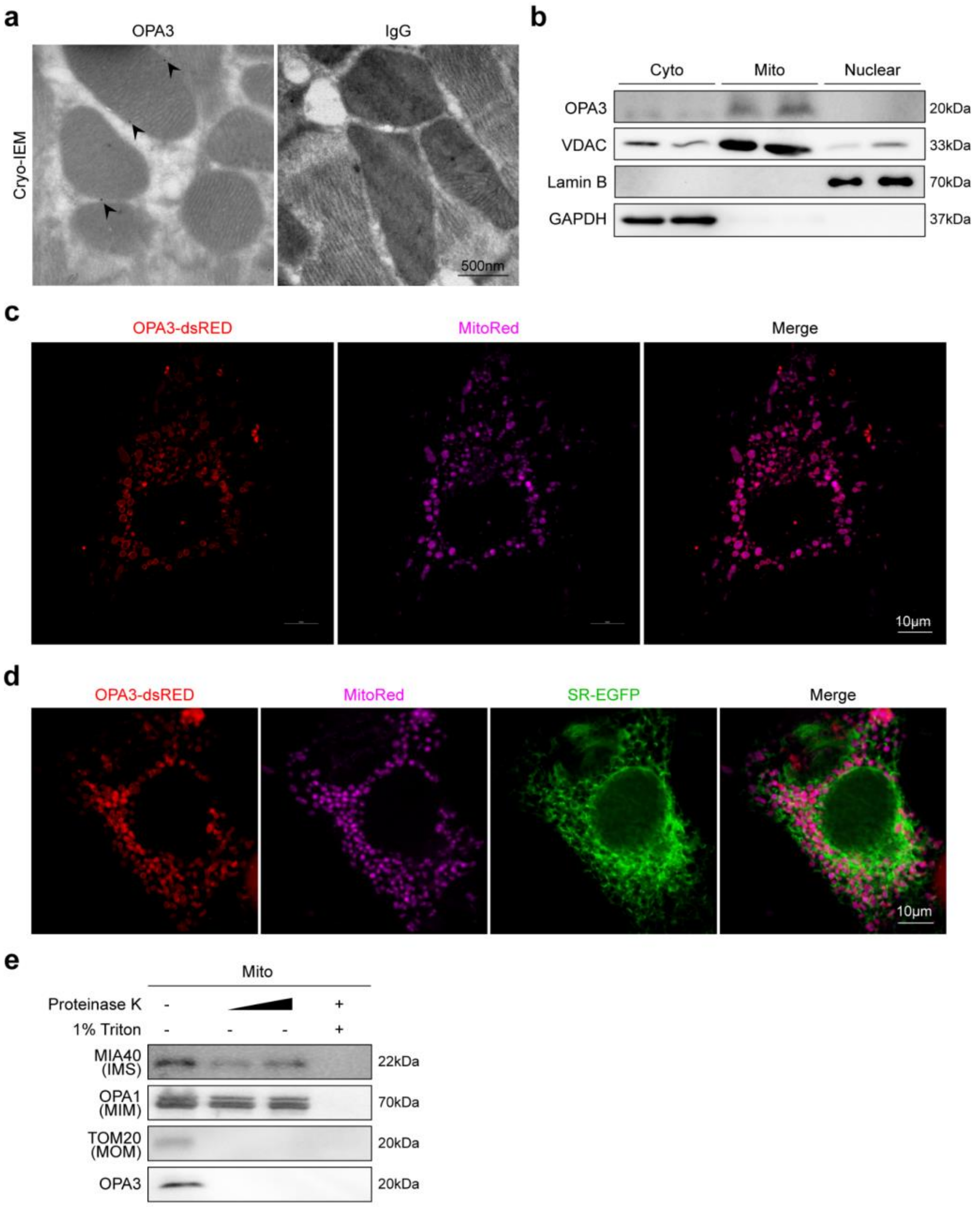

Supplementary Fig. 16. OPA3 localizes to the outer mitochondrial membrane.

a, Cryo-immunogold electron microscopy (Cryo-IEM) of mouse heart tissues was performed using 12-nm gold particles. Arrows indicate OPA3 labeling. **b**, Representative western blot for OPA3 in cytosolic (Cyto), mitochondrial (Mito), and nuclear (Nuclear) fractions from heart tissues. GAPDH, VDAC, and Lamin B were used as markers for cytosolic, mitochondrial, and nuclear loading controls, respectively. **c**, Representative images of MitoRed staining in H9C2 cells infected with adenovirus encoding OPA3-dsRED fusion protein for 48 h (scale bar, 10 μm). **d**, Representative images of MitoRed staining in H9C2 cells infected with adenovirus encoding OPA3-dsRED fusion protein and transfected with and SR-EGFP probe mRNA for 24 h (scale bar, 10 μm). **e**, Representative western blot for proteinase K protection assay. Specifically, isolated mitochondria were treated with 10 μg/ml proteinase K for 30 min in the absence or presence of 1% Triton X-100. GAPDH indicates glyceraldehyde-3-phosphate

dehydrogenase; OPA3, optic atrophy 3; SR, sarcoplasmic reticulum; VDAC, voltage-dependent anion channel.

References:

1. Ryu, S.W., Jeong, H.J., Choi, M., Karbowski, M. & Choi, C. Optic atrophy 3 as a protein of the mitochondrial outer membrane induces mitochondrial fragmentation. *Cell Mol Life Sci* **67**, 2839-2850 (2010).

3. For the general reader, please give more background/discussion on the OPA3 protein and OPA family in general. Are the different OPAs similar proteins or are they grouped together because they cause similar phenotypes? OPA1 contains a GTPase domain; is there significant homology between OPA1 and 3? If there is homology between OPA3 and other OPAs, is there anything known about multimerization in other OPAs?

Response:

Thank you for these constructive comments. The OPA gene family is typically grouped based on the shared clinical phenotype of optic atrophy.[1] However, these genes vary considerably in their molecular structure and biological function. For example, to evaluate the homology between OPA1 and OPA3, we utilized the "Align" function of the UniProt database to compare their sequences in both human and mouse. The analysis revealed that human OPA1 (UniProt ID: O60313) and OPA3 (UniProt ID: Q9H6K4), as well as their mouse orthologs (UniProt IDs: P58281 and Q505D7, respectively), share only 18.52% sequence identity (Fig. R1 and R2 shown in Review #1 response 1 and below). This limited sequence homology underscores the substantial structural differences between OPA1 and OPA3, which likely correspond to distinct biological functions. In addition, to our knowledge, no other OPA family member has been reported to exhibit multimerization.

To improve clarity for the general reader, we have added a brief description of the OPA gene family in the **Introduction** section "Optic atrophy (OPA) related gene family is grouped based on a shared clinical phenotype rather than molecular homology.[14] These genes have been identified as pivotal regulators in dominant optic atrophy, characterized by neuromuscular disorders.[14]". And a more detailed comparison of OPA1 and OPA3 in the **Discussion** section "OPA gene family primarily reflects a shared clinical phenotype rather than structural or functional homology. Indeed, OPA family members differ substantially in their molecular architecture and cellular roles. OPA1 is well-documented for its critical role in maintaining mitochondrial homeostasis and cardiac function.[16, 18, 20] OPA1 contains a GTPase domain that facilitates nucleotide-dependent dimerization, driving conformational changes and oligomer stabilization

essential for MIM fusion.[52] Imbalanced OPA1 processing leads to mitochondrial fragmentation and disruption of cardiac metabolism, which have been implicated in progression of DCM.[18] In contrast, OPA3 exhibits minimal sequence homology with OPA1 and lacks a GTPase domain, and its biological functions have remained far less understood. Our findings reveal that OPA3 also undergoes multimerization, a mechanism fundamentally distinct from the GTPase-driven oligomerization of OPA1. There was also controversy regarding the localization of OPA3 to the MIM vs. MOM in earlier studies.[24, 25] OPA3 was initially thought to be located in the MIM based on sequence predictions,[25] but our research demonstrated that OPA3 mainly localized to the MOM in cardiomyocyte mitochondria, aligning with prior observations in HeLa cells.[24] Taken together, the distinction between OPA1 and OPA3 provides important context for interpreting the role of OPA3 uncovered in our study and highlights the need to consider OPA3 as a functionally independent regulator in cardiac physiology rather than as a structural or evolutionary analogue of OPA1.". This reorganization should help readers better understand the functional and structural differences between OPA1 and OPA3. You can also refer to the revised manuscript, the Line 44–45 for **Introduction** and the Line 797–816 for **Discussion**.

Percent Identity Matrix

sp|Q9H6K4|OPA3_HUMAN	100.00%	18.52%
sp|O60313|OPA1_HUMAN	18.52%	100.00%

Fig. R1. Percent identity matrix of human OPA1 and OPA3 proteins from the UniProt database.

The matrix displays the sequence identity between human OPA1 (UniProt ID: O60313) and OPA3 (UniProt ID: Q9H6K4). OPA3 and OPA1 share 18.52% sequence identity, indicating limited sequence homology between the two proteins.

Percent Identity Matrix

sp|Q505D7|OPA3_MOUSE	100.00%	18.52%
sp|P58281|OPA1_MOUSE	18.52%	100.00%

Fig. R2. Percent identity matrix of mouse OPA1 and OPA3 proteins from the UniProt database.

The matrix displays the sequence identity between mouse OPA1 (UniProt ID: P58281) and OPA3 (UniProt ID: Q505D7). OPA3 and OPA1 share 18.52% sequence identity, indicating limited sequence homology between the two proteins.

References:

1. Lenaers, G. et al. Dominant optic atrophy. *Orphanet J. Rare Dis*. **7**, 46 (2012).

4. Has a specific cardiac phenotype been noted in any patients with dominant optic atrophy? It would be interesting to mention this if so.

Response:

Thanks for this suggestion. As highlighted in the review by Ham et al.,[1] "Some studies examined all systems including cardiac, and the presence of mitochondrial mutations in conjunction to the OPA1 mutation, whereas other smaller case studies did not. Therefore, whether individuals truly did not demonstrate cardiac or other manifestations is unknown.". Systematic cardiac assessments in ADOA patients are limited, and whether a specific cardiac phenotype is consistently present remains inconclusive. This clinical uncertainty underscores the need for deeper mechanistic investigations into the roles of OPA proteins in the heart.

References:

1. Ham, M., Han, J., Osann, K., Smith, M. & Kimonis, V. Meta-analysis of genotype-phenotype analysis of OPA1 mutations in autosomal dominant optic atrophy. *Mitochondrion* **46**, 262-269 (2018).

Detailed Response to the Reviewer #4

The work by Geng and colleagues investigates the role of OPA3 in the heart, particularly in the context of dilated cardiomyopathy and heart failure. By expliting available RNAseq datasets, the authors demonstrate that OPA3 expression is significantly downregulated in cardiomyocytes from both human patients with DCM and hypertrophic cardiomyopathy and in mouse models of heart failure. This downregulation correlates with the upregulation of classical heart failure markers, underscoring a potential role for OPA3 in cardiac pathophysiology.

Using constitutive and inducible cardiomyocyte-specific knockout models, the authors show that the absence of Opa3 leads to the development of dilated cardiomyopathy with systolic dysfunction, ventricular dilation, myocardial fibrosis, and reduced survival. In addition, pressure overload induced by transverse aortic constriction exacerbates the cardiac phenotype in Opa3-deficient mice, further highlighting the importance of OPA3 in maintaining cardiac homeostasis under stress.

At the mechanistic level, the authors find that OPA3 interacts with phospholamban (PLN), a key regulator of the sarcoplasmic reticulum (SR) Ca^{2+} ATPase SERCA2a. Loss of Opa3 reduces PLN phosphorylation, enhances the inhibitory PLN-SERCA2a interaction, impairs SR Ca^{2+} reuptake, and leads to elevated cytosolic diastolic Ca^{2+} levels. These defects caused diminished calcium transients and contractility in cardiomyocytes lacking OPA3. In addition, the authors show that OPA3 localizes predominantly to the mitochondrial outer membrane, where it appears to play a key role in coordinating mitochondrial-SR communication. Opa3 deletion increases the distance between mitochondria and SR, causes mitochondrial depolarization, promotes permeability transition pore opening, and reduces oxygen consumption and ATP production.

A interesting finding is that OPA3 works forming multimers, and that mutations disrupting cysteine residues essential for multimer formation (C42 and C96) abolish its interaction with PLN and impair its ability to support mitochondrial function. These multimer-deficient OPA3 variants fail to rescue cardiac dysfunction or mitochondrial defects in transverse aortic constriction models.

The authors also provide compelling evidence that cardiomyocyte-specific overexpression of OPA3 by AAV9 vectors improves cardiac function and mitigates pathological remodeling in both doxorubicin-induced and transverse aortic constriction-induced cardiomyopathy models. In contrast, overexpression of the multimer-deficient OPA3 mutant does not yield any functional benefit, emphasizing the importance of OPA3 structural integrity in mediating its

protective effects.

Overall, this is an interesting study which identify OPA3 as a new player in cariomypathies.
However, I have some comments that the authors should address.

Response:

We sincerely thank you for the thorough and insightful summary of our study. We also appreciate the constructive comments you have provided. Below, we address each of your concerns point by point, and we have revised the manuscript accordingly to improve clarity.

1. To the best of my knowledge, patients with mutations in OPA3 present with 3-methylglutaconic aciduria type III or optic atrophy, but no cardiac symptoms have been reported. How do the authors reconcile this with their findings?

Response:

While OPA3 mutations are primarily associated with 3-methylglutaconic aciduria type III and optic atrophy in humans, animal model studies have provided important insights into the broader physiological roles of OPA3, including its potential involvement in cardiac function. Specifically, OPA3 mutations have been shown to play a crucial role in the development of DCM in cattle.[1, 2] Our own findings also demonstrate that the ablation of *Opa3* in cardiomyocytes results in severe DCM in mice.

One possible explanation for the absence of consistent cardiac symptoms in human patients with OPA3 mutations is genotypic variation. We have identified that mutations affecting OPA3 multimerization sites, particularly at critical cysteine residues, impair OPA3 multimerization and disrupt its function in mitochondrial and SR-associated processes. These results suggest that OPA3 multimerization is pivotal for its normal function, and mutations that interfere with this process could contribute to cardiac dysfunction. In cases where the mutation does not affect multimerization or its interaction with key proteins such as PLN, cardiac symptoms may be less pronounced or difficult to detect in routine clinical assessments. Furthermore, subclinical cardiac abnormalities in human carriers of specific OPA3 mutations might have been overlooked without systematic cardiac phenotyping. Nevertheless, future studies should focus on investigating the role of OPA3 mutations in human heart pathology to fully understand the potential for cardiac involvement.

References:

1. Horiuchi, N. et al. Detection of the nonsense mutation of OPA3 gene in Holstein Friesian cattle with dilated cardiomyopathy in Japan. J. Vet. Med. Sci. 77, 1281-1283

(2015).

2. Owczarek-Lipska, M. et al. A nonsense mutation in the optic atrophy 3 gene (OPA3) causes dilated cardiomyopathy in Red Holstein cattle. Genomics 97, 51-57 (2011).

2. I am not convinced by OPA3 localization to the outer mitochondrial membrane. First, although OPA3 behaves as TOM20 i the PK experiment, it would be important to have markers also for the other mitochondrial compartments. Second, the immuno-electron microscopy images are particularly clear and better quality images should be used. Third, the immuno-EM data suggesting non-mitochondrial localization are not supported by the fractionation experiments (Extended Data Fig. 16). Fourth, the mitotracker data are again not convincing as the typical mitochondrial pattern is not recognizable. Obviously, the interaction with phospholamban requires an accessible localization. However, a solid proof evidence must be provided in order to properly intepret the data.

Response:

Thank you for the insightful comments and helpful suggestions.

First, in addition to previously demonstrated potential localization of OPA3 in the mitochondrial outer membrane (MOM) and mitochondrial inner membrane (MIM) compartments, as indicated by TOM20 and OPA1, respectively, we also examined the intermembrane space (IMS) of mitochondria using MIA40.[1] To determine the exact compartment harboring OPA3, we treated isolated mitochondria with proteinase K. Similar to the behavior of the MOM marker TOM20, proteinase K treatment resulted in the degradation of OPA3 (Supplementary Fig. 16e). In contrast, the IMS component MIA40 and the MIM protein OPA1 remained unaffected unless the membranes were solubilized with detergent. Please refer to the Line 666–674 in the **Results** section for further details.

Second, regarding the IEM images, we replaced the previous approach with cryo-immunogold electron microscopy, with optimized antibody concentrations to enhance signal specificity and spatial resolution. Using this method, we observed that OPA3 is predominantly localized to mitochondria in adult mouse hearts (Supplementary Fig. 16a). To provide a clearer presentation of OPA3 localization, we re-performed the Mitotracker co-staining experiments using higher-resolution imaging, which revealed a clearer and more characteristic mitochondrial pattern that co-localizes with OPA3 (Fig. 6a). Moreover, evidence from study in non-cardiac cells consistently demonstrates that OPA3 resides in the MOM,[2] which provides additional support for our localization findings.

Third, to further substantiate the interaction between OPA3 and phospholamban (PLN)

and confirm their spatial relationship, we performed proximity ligation assays (PLA) in conjunction with mitochondrial staining. Our results demonstrated that the OPA3-PLN interaction occurs at the mitochondrial edge (Fig. 5h), providing stronger evidence for the relevance of this interaction.

We hope these additional experiments and clarifications address your concerns and further strengthen the conclusions of our study.

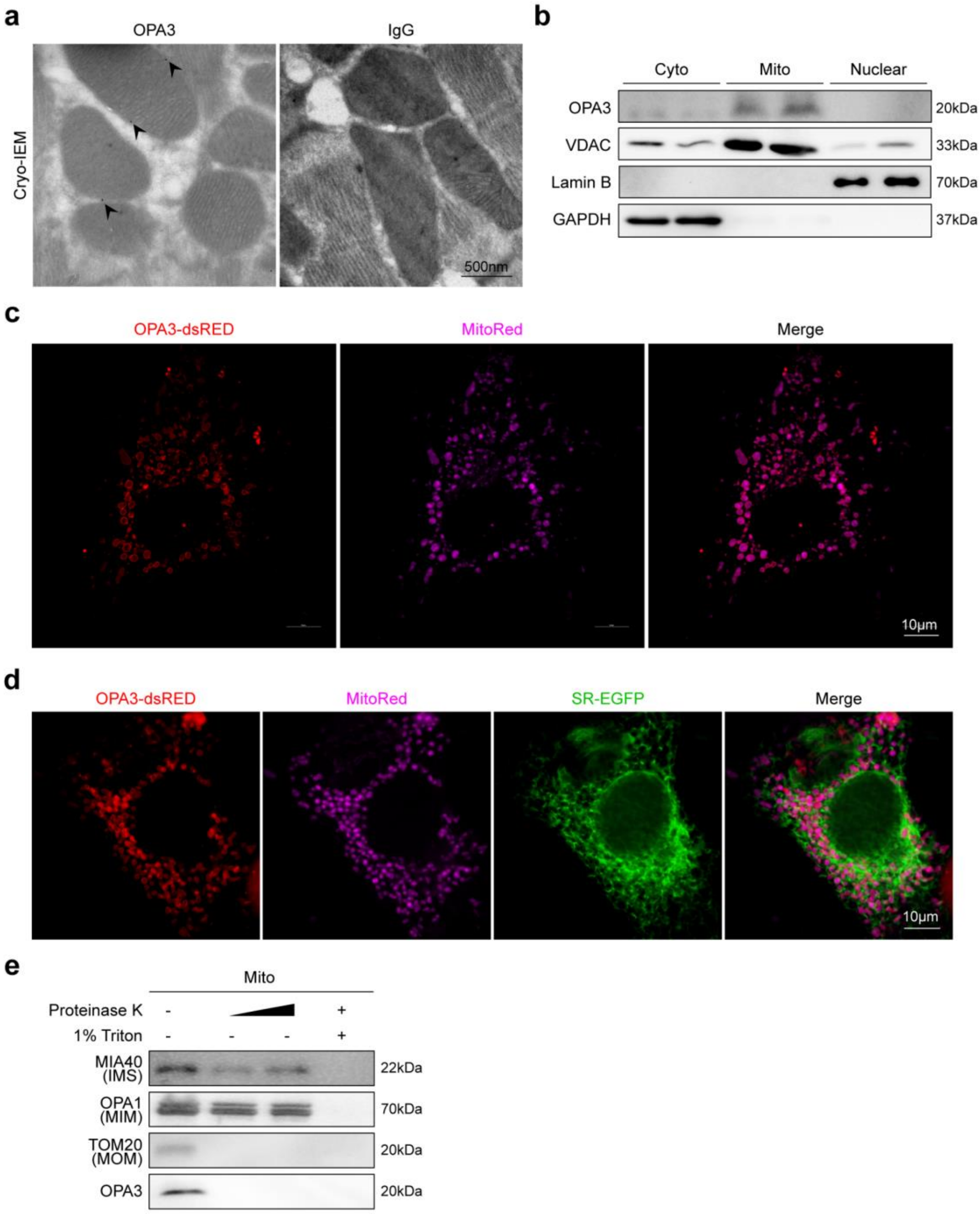

Supplementary Fig. 16. OPA3 localizes to the outer mitochondrial membrane.

a, Cryo-immunogold electron microscopy (Cryo-IEM) of mouse heart tissues was performed

using 12-nm gold particles. Arrows indicate OPA3 labeling. **b**, Representative western blot for OPA3 in cytosolic (Cyto), mitochondrial (Mito), and nuclear (Nuclear) fractions from heart tissues. GAPDH, VDAC, and Lamin B were used as markers for cytosolic, mitochondrial, and nuclear loading controls, respectively. **c**, Representative images of MitoRed staining in H9C2 cells infected with adenovirus encoding OPA3-dsRED fusion protein for 48 h (scale bar, 10 μm). **d**, Representative images of MitoRed staining in H9C2 cells infected with adenovirus encoding OPA3-dsRED fusion protein and transfected with and SR-EGFP probe mRNA for 24 h (scale bar, 10 μm). **e**, Representative western blot for proteinase K protection assay. Specifically, isolated mitochondria were treated with 10 μg/ml proteinase K for 30 min in the absence or presence of 1% Triton X-100. GAPDH indicates glyceraldehyde-3-phosphate dehydrogenase; OPA3, optic atrophy 3; SR, sarcoplasmic reticulum; VDAC, voltage-dependent anion channel.

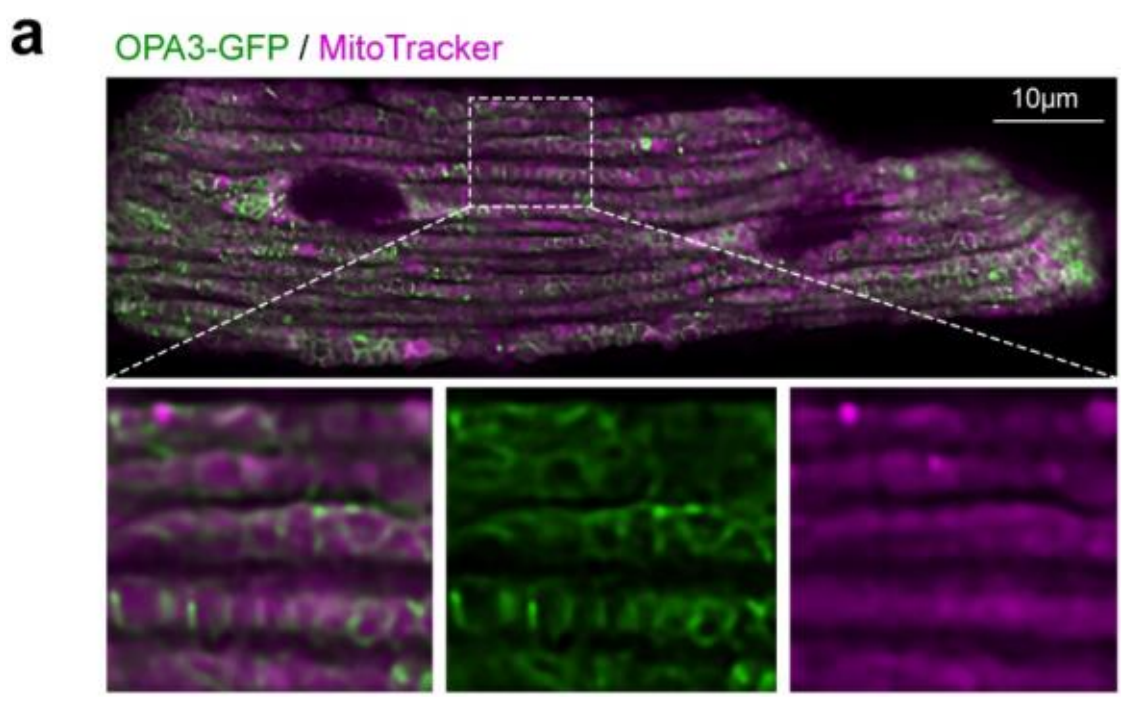

Fig. 6 OPA3 predominantly localizes to the outer mitochondrial membrane and regulates mitochondria-SR communication.

a, Representative images of MitoTraker staining in isolated AMCMs from *Opa3*$^{fl/fl}$ mice after infected with adenovirus encoding OPA3-GFP fusion protein for 48 h (scale bar, 10 μm).

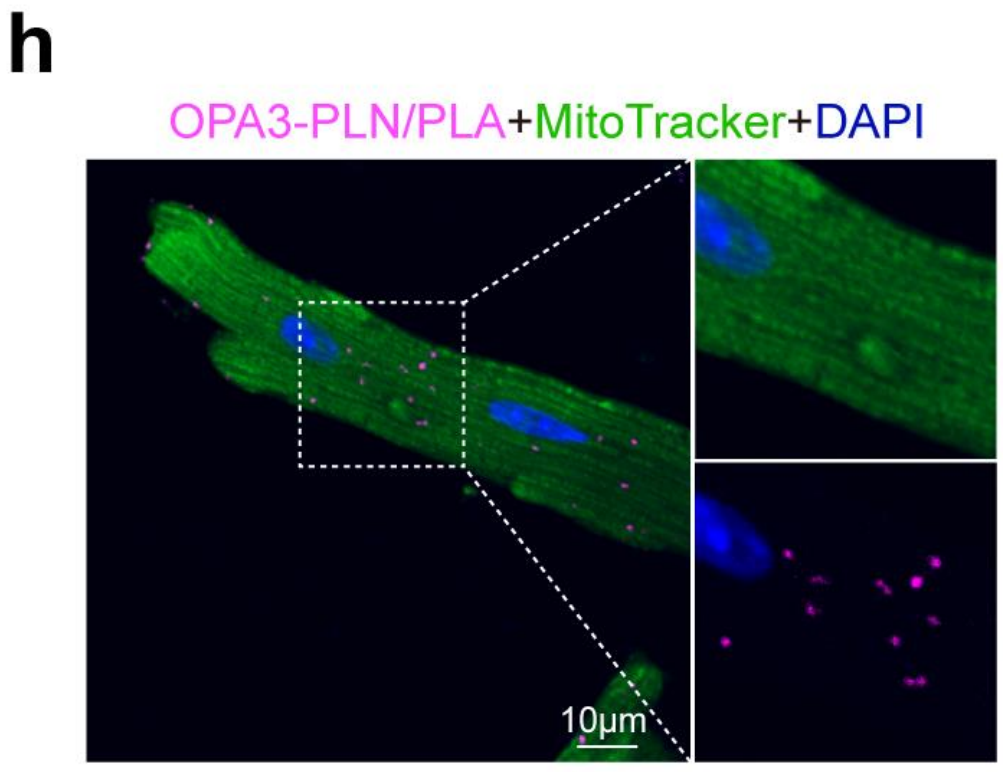

Fig. 5. OPA3 interacts with SR-located protein phospholamban (PLN).

h, The interaction between OPA3 and PLN was assessed by proximity ligation assay (PLA). Mitochondria were labeled with MitoTracker, and nuclei were counterstained with DAPI (blue).

References:

1. McMinimy, R. et al. Reactive oxygen species control protein degradation at the

mitochondrial import gate. *Mol. Cell* **84** (2024).

2. Ryu, S.W., Jeong, H.J., Choi, M., Karbowski, M. & Choi, C. Optic atrophy 3 as a protein of the mitochondrial outer membrane induces mitochondrial fragmentation. *Cell Mol Life Sci* **67**, 2839-2850 (2010).

3. Also the analysis of mitochondrial-SR contacts is not very convincing, and better quality EM images should be used, that should also evaluate cristae morphology.

<u>Response:</u>

Thank you for this helpful comment. In the revised manuscript, we have provided higher-resolution TEM images to more accurately assess mitochondrial ultrastructure and mitochondria-SR contacts. Using these improved images, we carefully assessed cristae morphology and found no significant alterations in *Opa3*-deficient hearts compared with controls (Fig. 6b). Please refer to the Line 679 in the **Results** section.

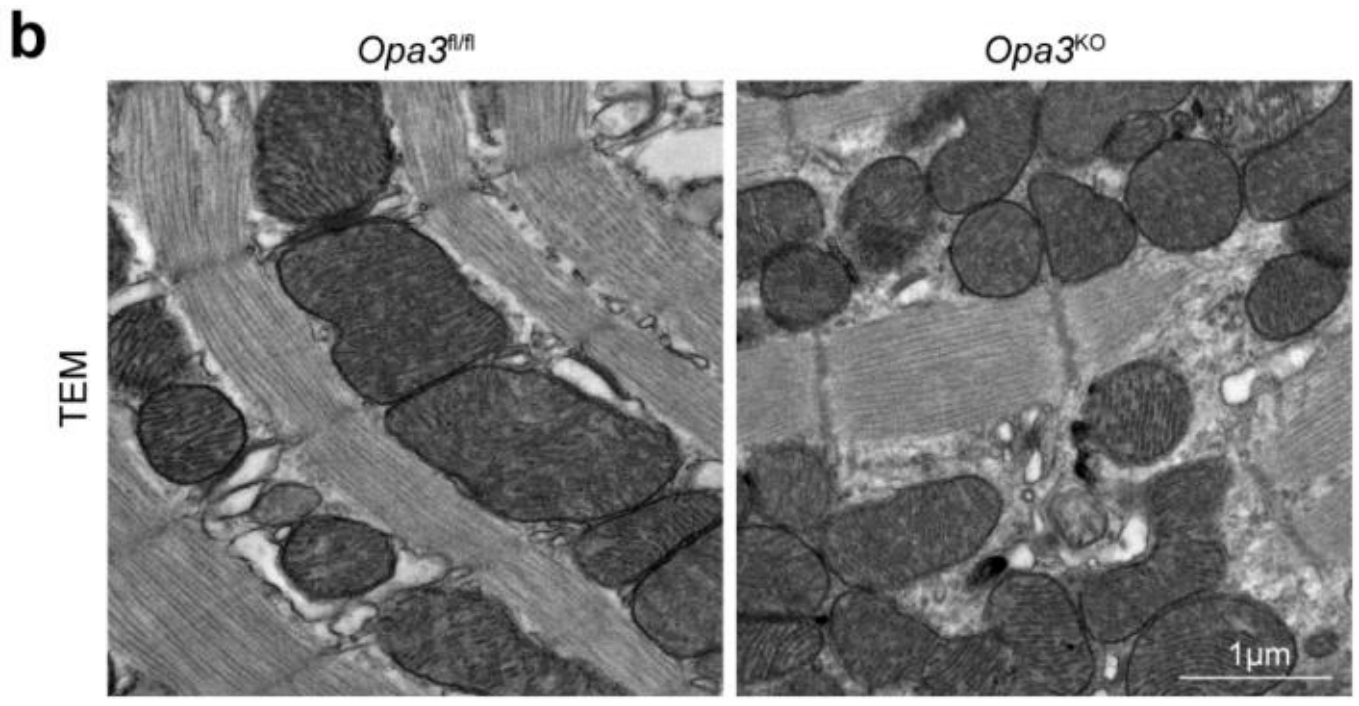

Fig. 6. OPA3 predominantly localizes to the outer mitochondrial membrane and regulates mitochondria-SR communication.

b, Transmission electron micrographs (TEM) of ventricular tissue from *Opa3*$^{fl/fl}$ and *Opa3*^{KO} mice at 8 weeks of age (scale bars, 1 μm).

4. The experiments to assess the mitochondrial membrane potential lack a key control, ie the use of an uncoupler such as FCCP. Without this it is impossible to properly evaluate the changes observed with the JC1 dye.

<u>Response:</u>

Thanks. In the revised manuscript, we have included FCCP treatment as a positive control to confirm reliability of the observed JC-1 fluorescence changes (Supplementary Fig. 18a, b).

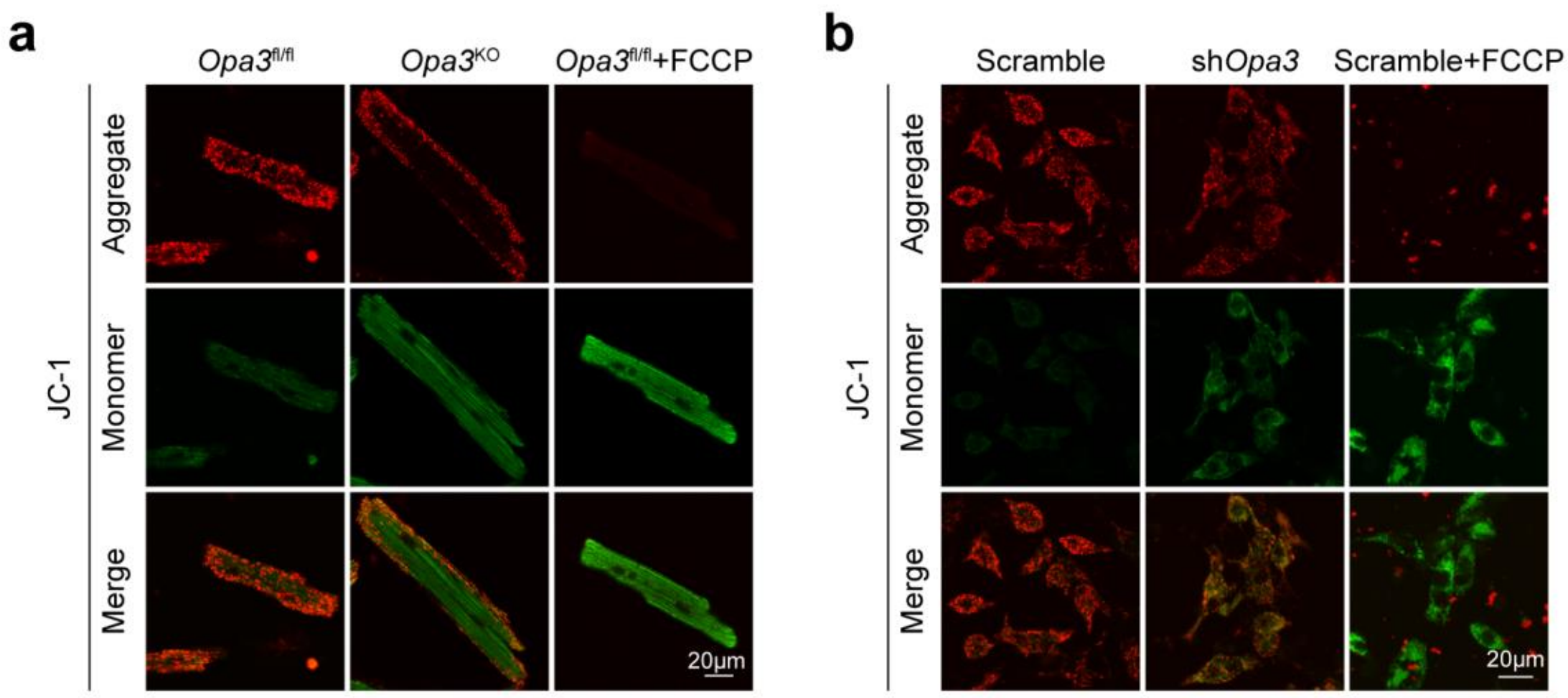

Supplementary Fig. 18. The role of OPA3 in mitochondrial ΔΨm.

a, Representative images of mitochondrial ΔΨm assessed by JC-1 staining in isolated AMCMs from *Opa3*$^{fl/fl}$ and *Opa3*^{KO} mice (scale bar, 20 μm). FCCP was used as a positive control. **b**, Representative images of mitochondrial ΔΨm assessed by JC-1 staining in isolated NMCMs (scale bar, 20 μm). Specifically, the NMCMs were infected with adenovirus encoding scramble (Scramble) or shRNA targeting *Opa3* (sh*Opa3*) for 48 h. FCCP was used as a positive control.

5. The authors observe an increase in PTP: does cyclosporin A improves the phenotype of the mice?

Response:

Thanks. We treated the the *Opa3*-deficient mice with cyclosporin A or vehicle according to the previous study (25 mg/kg, subcutaneously for 24 days).[1] Our preliminary data indicated that pharmacological inhibition of the mPTP using cyclosporin A (CsA) partially ameliorates the impaired cardiac function observed in *Opa3*-deficient mice (Fig. R8). We agree that these findings are valuable for strengthening the manuscript. We included these preliminary results as supplementary data and are prepared to conduct a more comprehensive set of experiments (e.g., assessing mitochondrial function and cell death directly after CsA treatment) in a future study to fully elucidate the mechanism. We will revise the manuscript accordingly if you consider this addition appropriate.

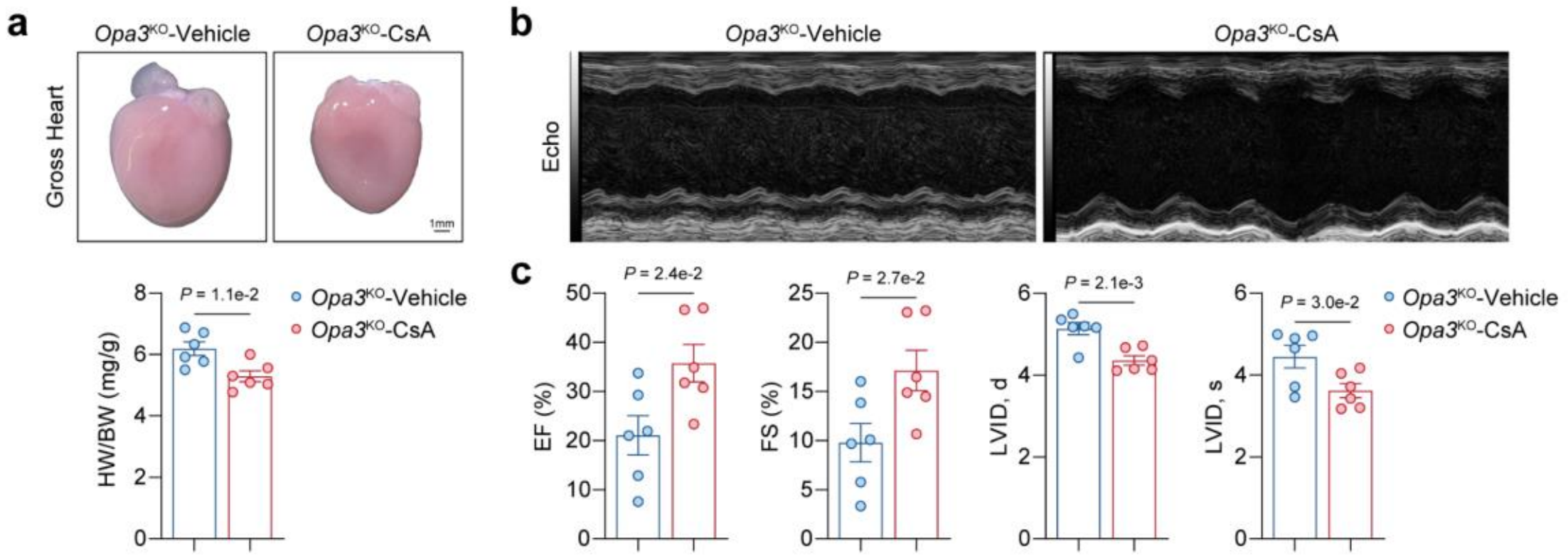

Fig. R8. Cyclosporin A administration attenuated cardiac dysfunction in *Opa3* knockout mice.

a, **Top**, Representative gross images of hearts from indicated groups (scale bar, 1 mm). **Bottom**, Ratios of heart weight to body weight (HW/BW) in indicated groups (n=8 per group). Data were analyzed by Student's *t* test. **b** and **c**, Representative M-mode echocardiographic images (**b**) and corresponding measurements of EF, FS, LVID,d, and LVID,s (**c**) in indicated groups (n=8 per group). Data were analyzed by Student's *t* test.

<u>**References:**</u>

1. Meguro, T. et al. Cyclosporine attenuates pressure-overload hypertrophy in mice while enhancing susceptibility to decompensation and heart failure. *Circ. Res.* **84**, 735-740 (1999).

6. Page 15 line 458: to improve readibility what CM1/2/3 mean should be included here.

<u>**Response:**</u>

Thank you for pointing this out. CM1, CM2, and CM3 refer to cardiomyocyte subclusters identified by our snRNA-seq analysis. To improve readability, we have now defined these abbreviations in the main text and corresponding figure legends.

Detailed Response to the Reviewer #1

I believe the authors have done a commendable job in addressing the reviewers' comments. The additional supplementary figures are convincing and strengthen the manuscript. I have no further comments or questions.

Response:

We are grateful for your constructive comments.

Detailed Response to the Reviewer #2

The authors have responded favorably to my previous concerns.

Response:

Thanks for your constructive suggestions.

Only one issue requires attention. The images shown in supplementary Fig 13 does not have any detectable Ca sparks. Please replace with images where Ca sparks are clearly visible. Maybe eliminating the portion of the ca transients will allow enough contrast to see them.

Response:

In the revised manuscript, we have eliminated the portion of the Ca^{2+} transients and enhanced contrast as you suggested (see revised Supplementary Fig.13).

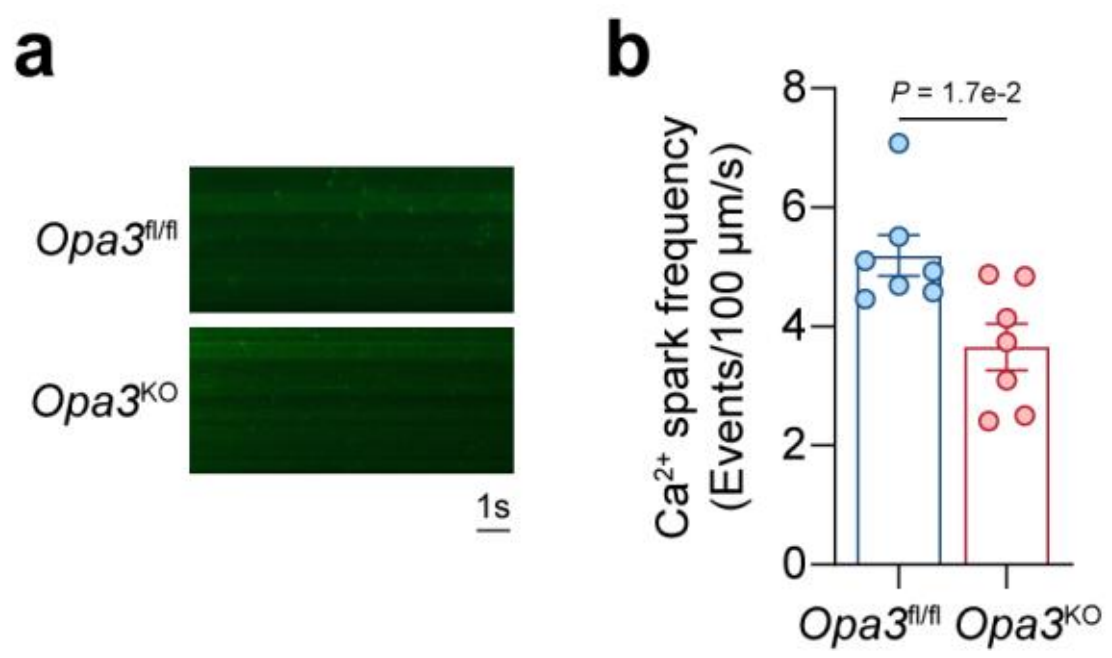

Supplementary Fig. 13. Deficiency of *Opa3* decreases SR Ca^{2+} leak.

a, Line scan images show Ca^{2+} sparks of indicated groups after 2 Hz stimulation. **b**, Quantification of Ca^{2+} sparks frequency in (**a**). Data were analyzed by two-sided Mann-Whitney *U* test. Data are expressed as mean ± SEM. Opa3 indicates optic atrophy 3; SR, sarcoplasmic reticulum.

I failed to see which RyR2 residue's phosphorylation was studied in the new experiments. These data should be added to the new version, either in the supplementary figure, or in the text.

Response:

The phosphorylation of RyR2 at Ser2808 was detected in our previous experiments, and

the corresponding results have been added to the manuscript. Please refer to the updated **Results** section for these data.

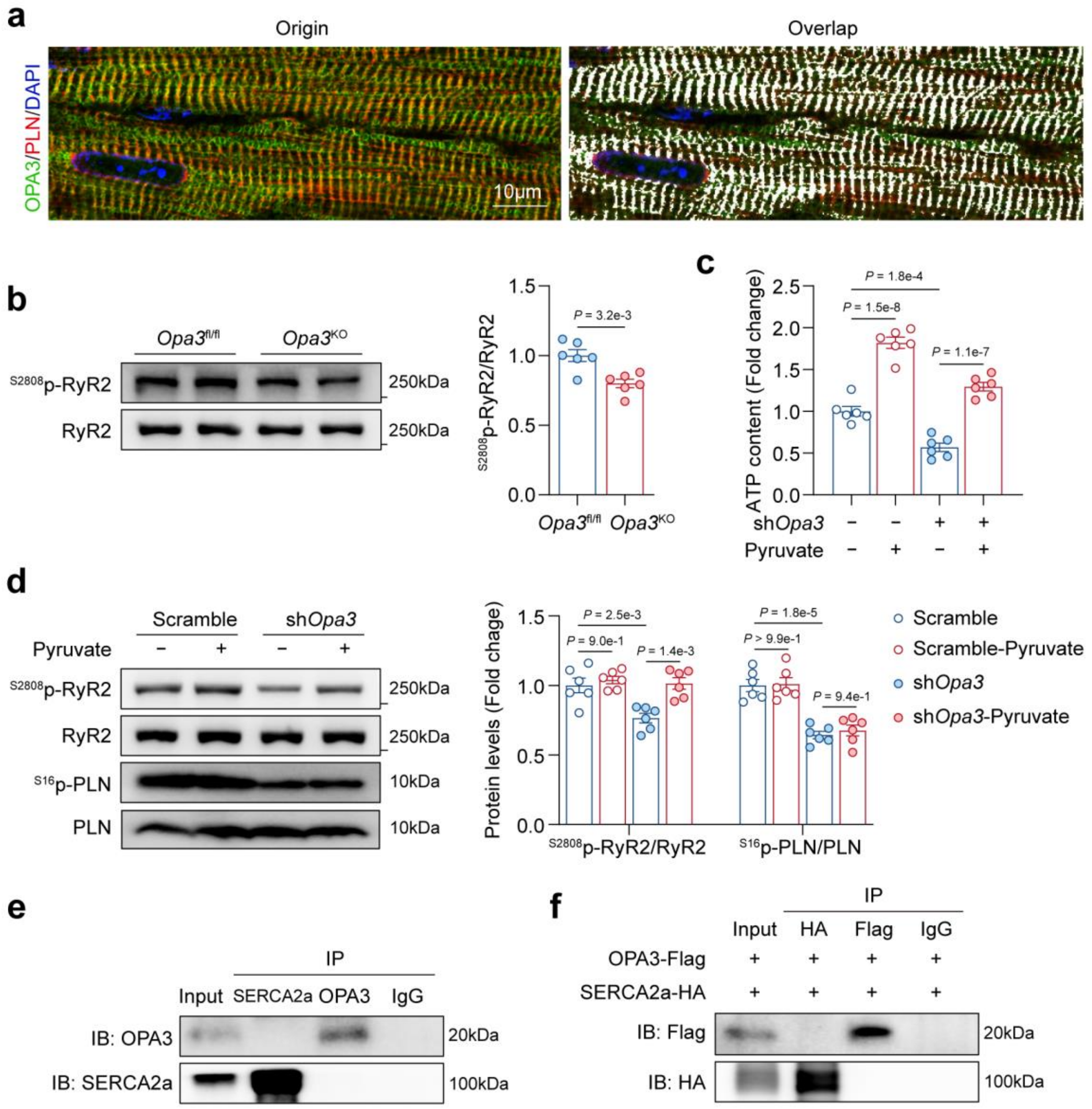

Supplementary Fig. 15. The role of OPA3 in PKA signaling pathway.

a, Representative images of the colocalization of OPA3 (green) and PLN (red) in heart sections (scale bars, 10 μm). Nuclei were counterstained with DAPI (blue). The upper panels show the original fluorescence images, and the lower panels display the colocalized OPA3-PLN regions (white) identified using *FIJI* software. Colocalized OPA3-PLN signals were quantified and normalized to total OPA3 fluorescence intensity to estimate the fraction of OPA3 associated with PLN *in situ*. **b**, Representative western blots and quantification of RyR2 and its phosphorylation in PE-treated (50 nM for 48 h) AMCMs isolated from *Opa3*$^{fl/fl}$ and *Opa3*^{KO} mice (n=6 per group). **c**, ATP content was determined by ATP Assay Kit (n=6 per group). **d**, Representative western blots in indicated groups. NMCMs were infected with adenovirus encoding scramble (Scramble) or shRNA targeting *Opa3* (sh*Opa3*) for 48 h. NMCMs were then supplemented with pyruvate (20 mM) for 30 min and treated with db-cAMP (10 μM). **e**, Coimmunoprecipitation of OPA3 and SERCA2a in heart tissues. Endogenous OPA3 or SERCA2a was immunoprecipitated by anti-

OPA3 antibody or anti-SERCA2a antibody, respectively, using IgG as a negative control. **f**, Coimmunoprecipitation of OPA3 and SERCA2a in HEK293T cells co-transfected with OPA3-Flag and SERCA2a-HA plasmids. Exogenous OPA3-Flag or SERCA2a-HA was immunoprecipitated by anti-Flag antibody or anti-HA antibody, respectively, using IgG as a negative control. Data are expressed as mean ± SEM. Data in **b** were analyzed by two-sided Student's *t* test. Data in **c**, **d** were analyzed by one-way ANOVA followed by the Tukey post hoc test. AMCMs indicates adult mouse cardiomyocytes; NMCMs, neonatal mouse cardiomyocytes; OPA3, optic atrophy 3; PKA, protein kinase A; PLN, phospholamban; RyR2, ryanodine receptor 2; SERCA, sarcoplasmic/endoplasmic reticulum Ca^{2+}-ATPase.

Detailed Response to the Reviewer #3

The authors have responded well to each of my points raised in the first review, carrying out additional experiments and altering the text. They also seem to have responded positively to the other reviewers' comments, and I believe have produced an improved manuscript.

Response:

Thank for your insightful suggestions.

Detailed Response to the Reviewer #4

I appreciate the effort the authors have made to address my concerns and comments. I have no futher comments.

Response:

Thank for your constructive comments.
