# Supplementary material for: Mitochondrial protein OPA3 sustains cardiac function by regulating calcium handling in male mice
Source: Nat Commun. 2026 Jun 17;17:7665. doi: 10.1038/s41467-026-73991-4 (PMC13434718; doi:10.1038/s41467-026-73991-4)

Full unedited gel for Fig. 1d

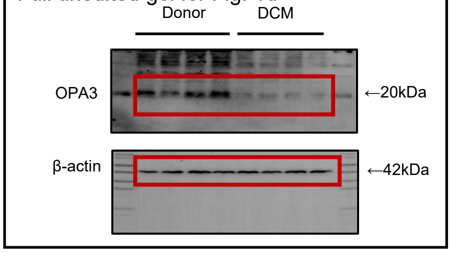

Full unedited gel for Fig. 1e

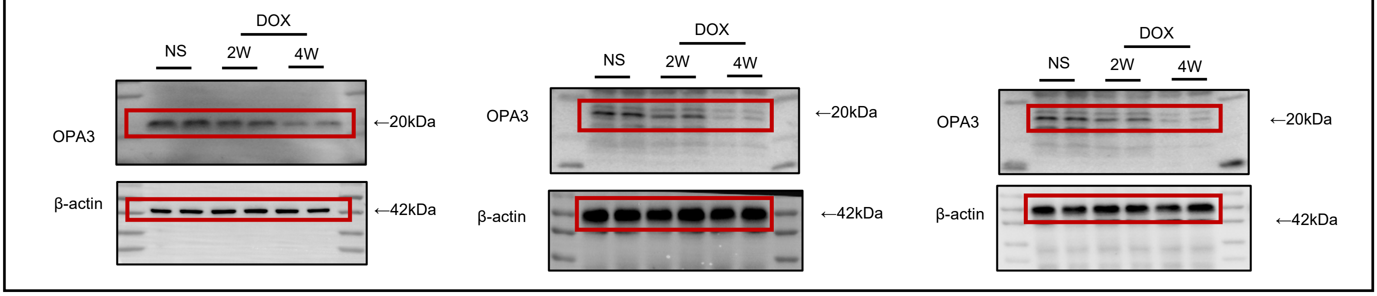

Full unedited gel for Fig. 1f

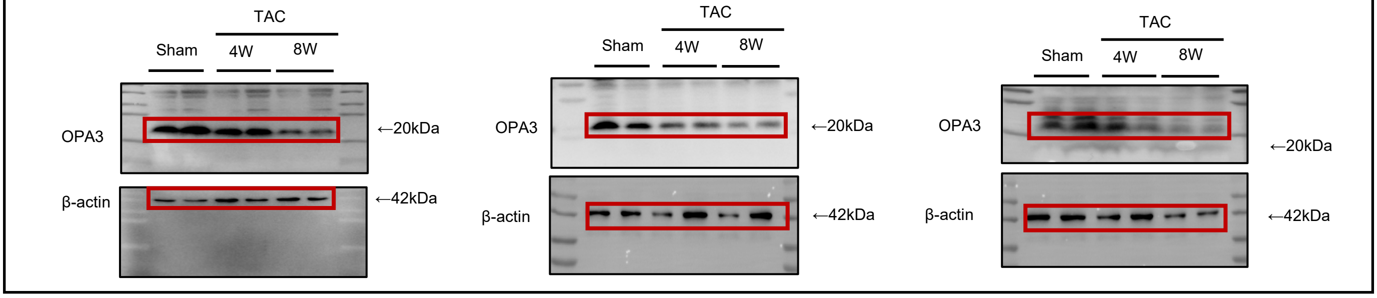

Full unedited gel for Fig. 2b

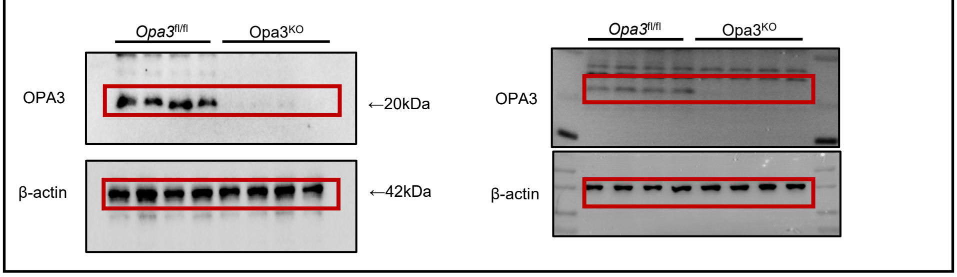

Full unedited gel for Fig. 5e

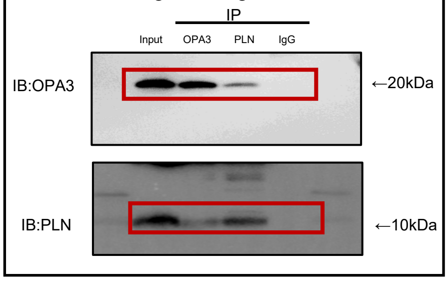

Full unedited gel for Fig. 5f

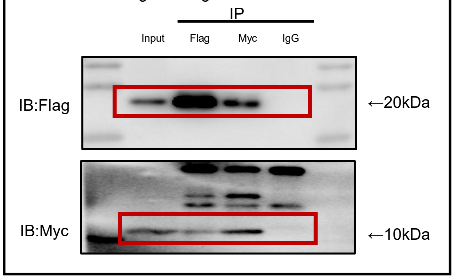

Full unedited gel for Fig. 5i

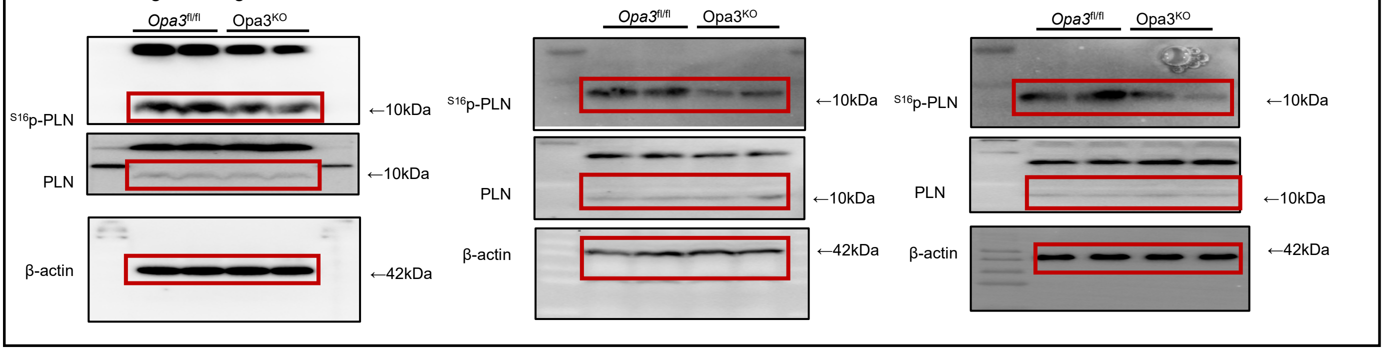

Full unedited gel for Fig. 5j

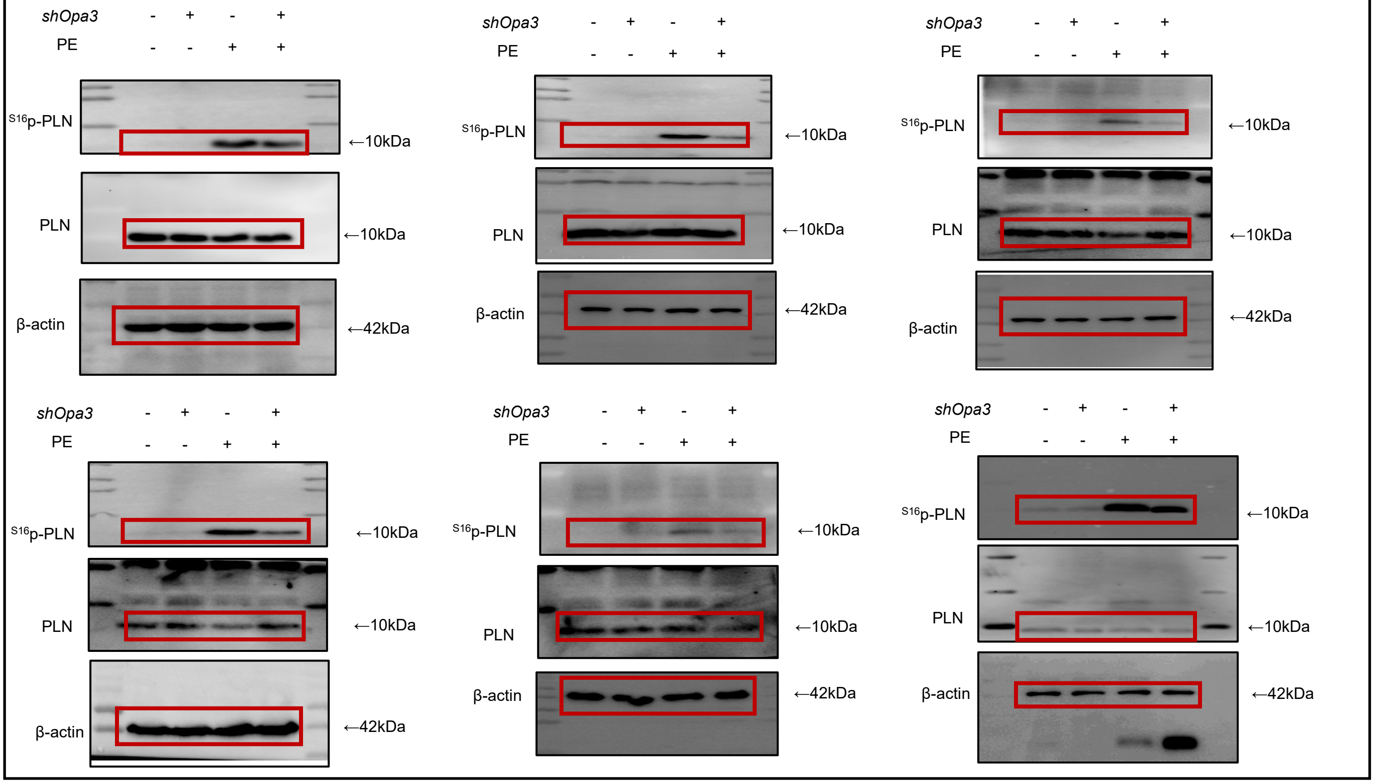

Full unedited gel for Fig. 5k

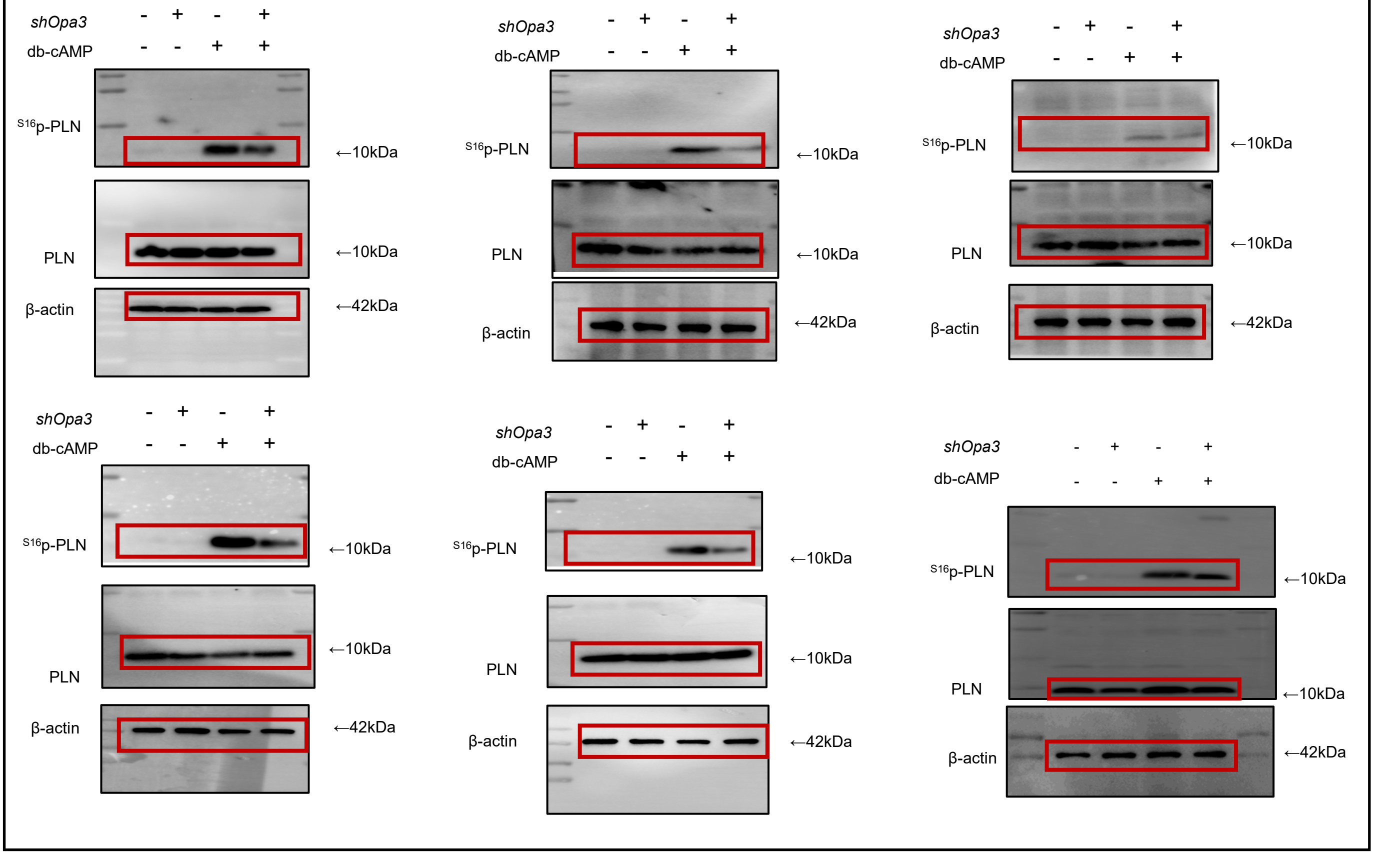

Full unedited gel for Fig. 5l

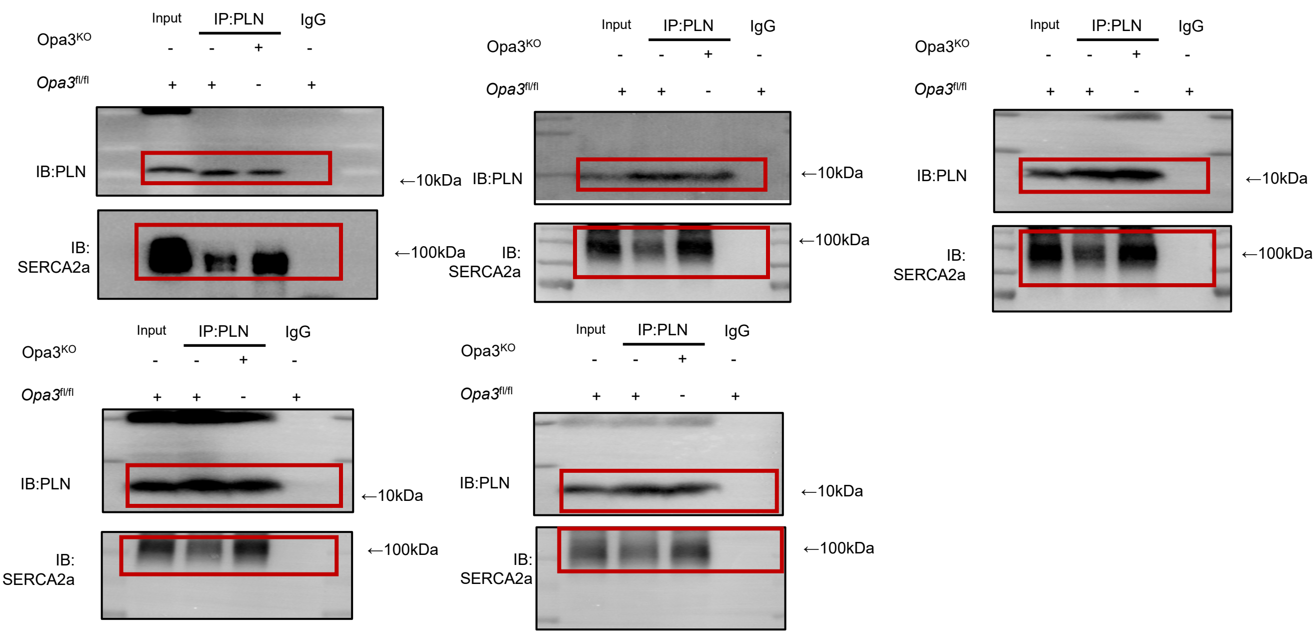

Full unedited gel for Fig. 7a

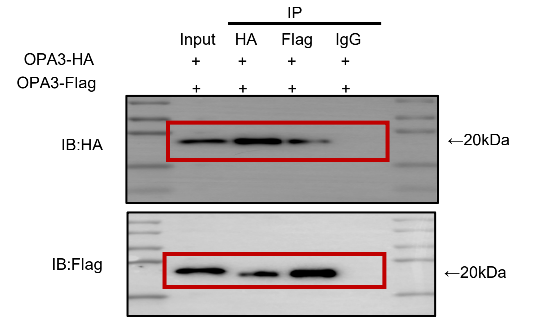

Full unedited gel for Fig. 7b

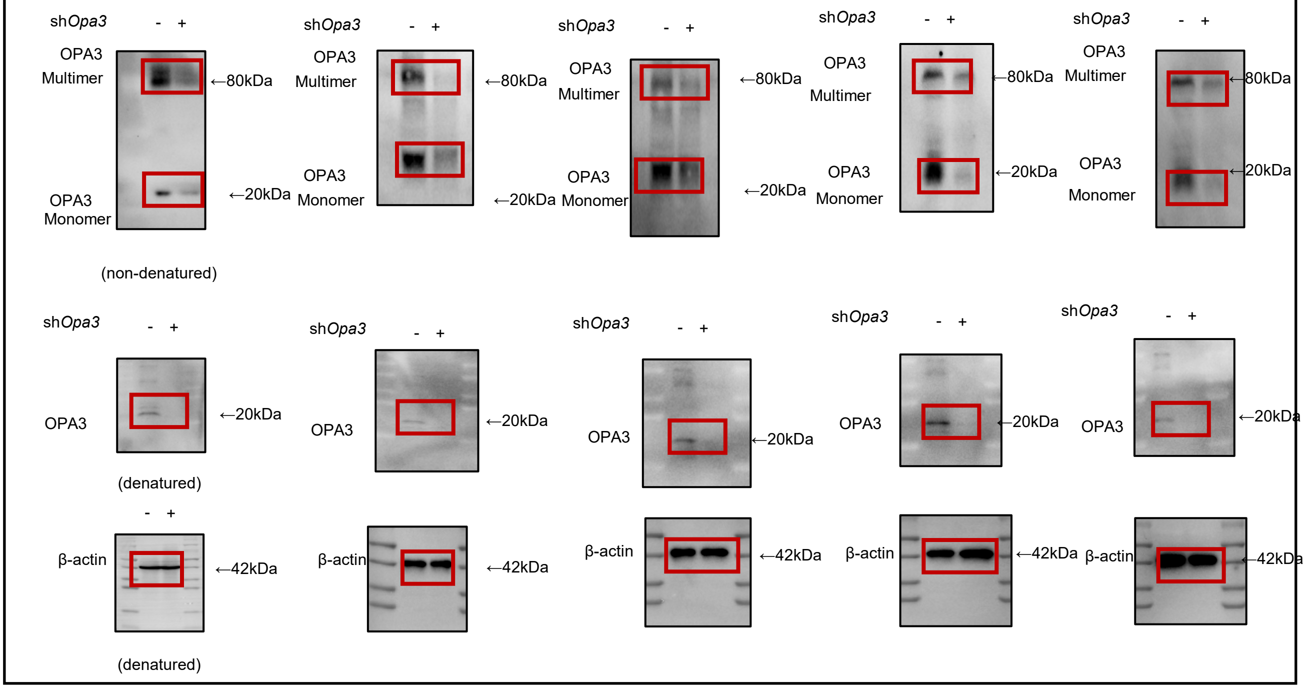

Full unedited gel for Fig. 7d

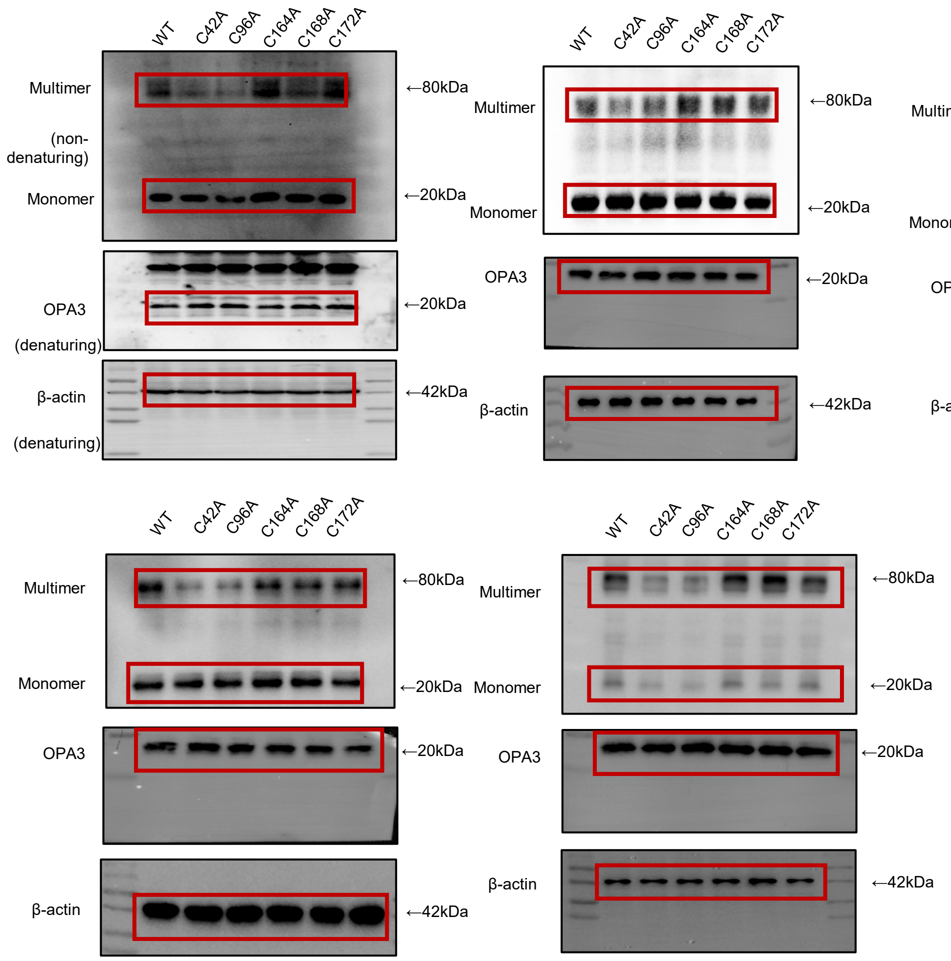

Full unedited gel for Fig. 7e

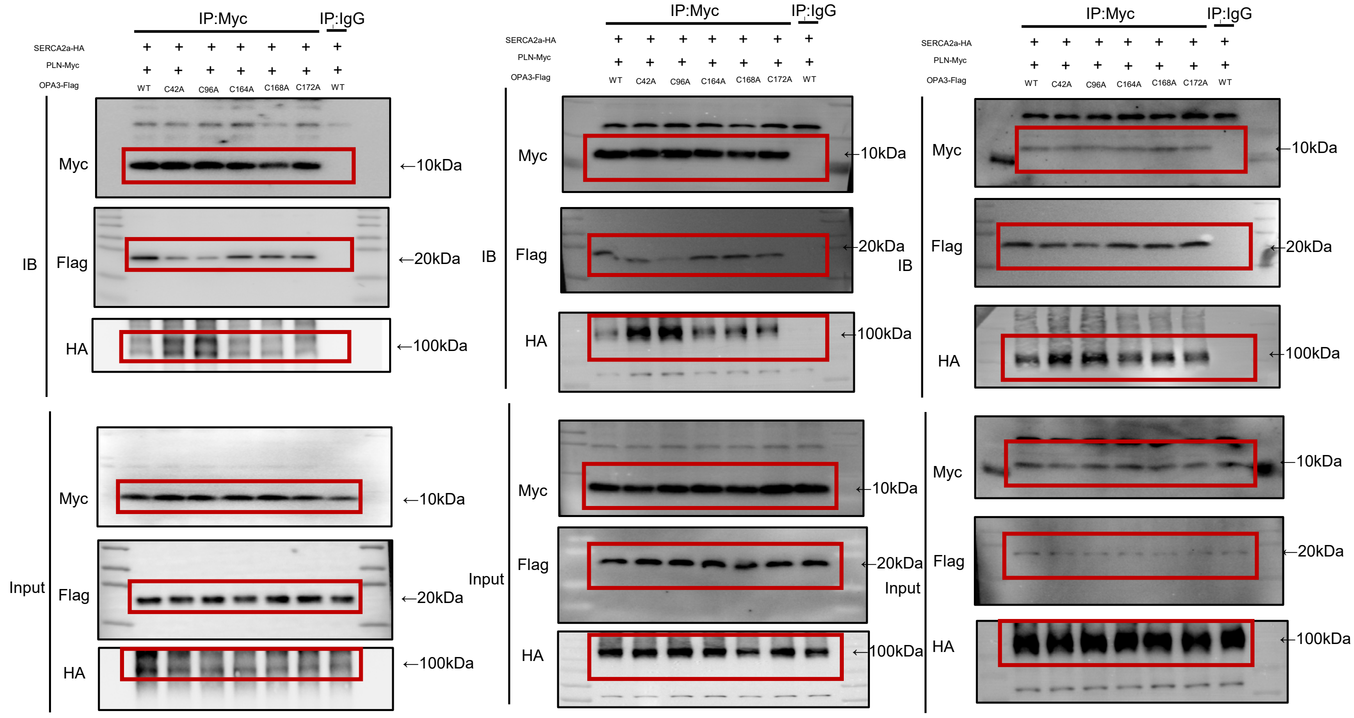

Full unedited gel for Fig. 7e

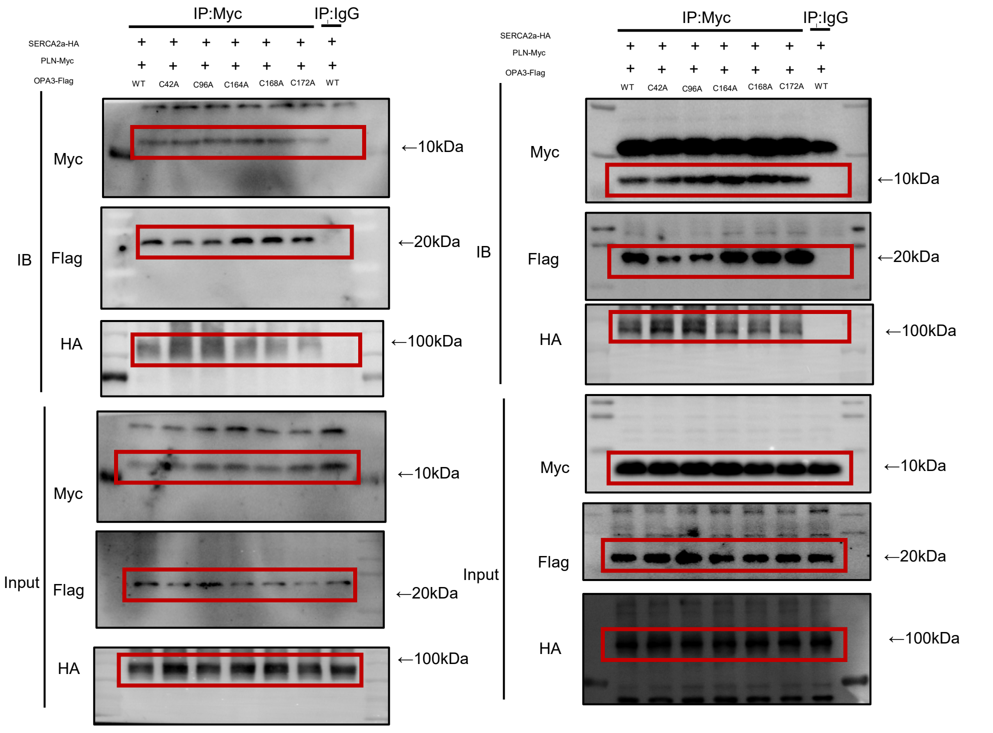

Full unedited gel for Fig. 8j

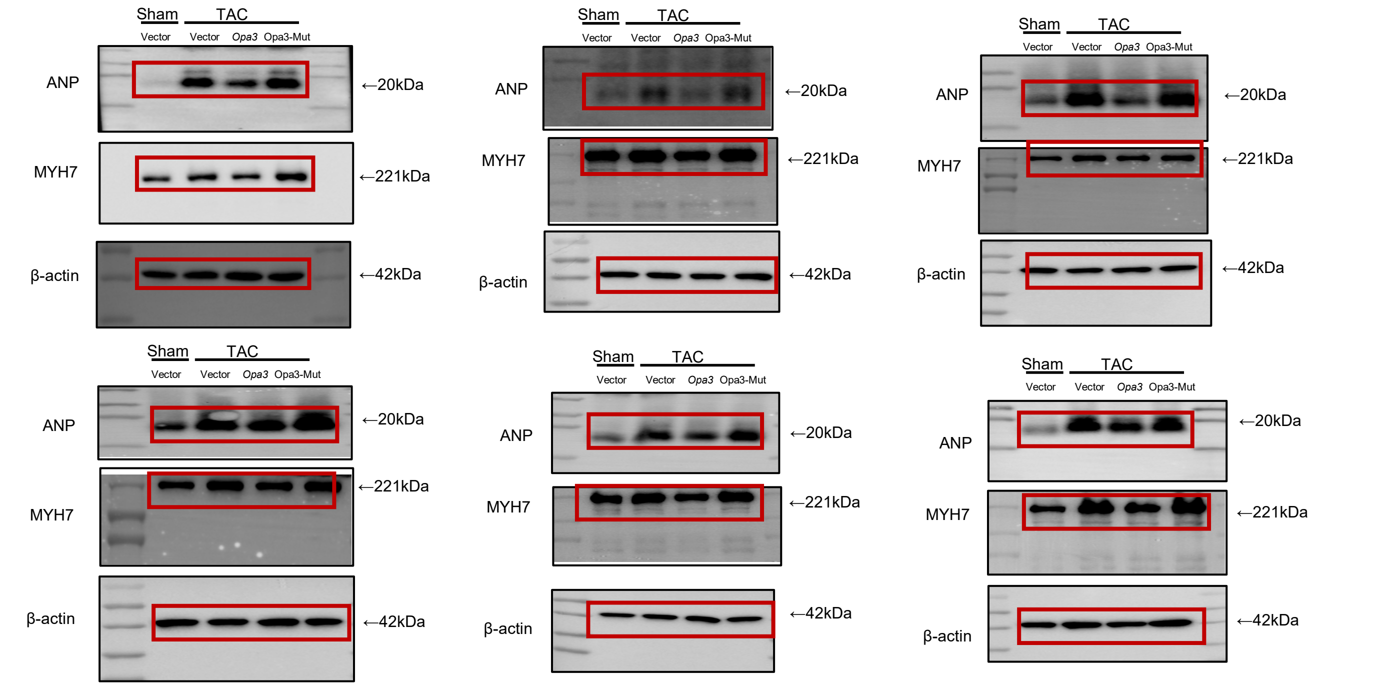

Full unedited gel for Supplementary Fig. 4f

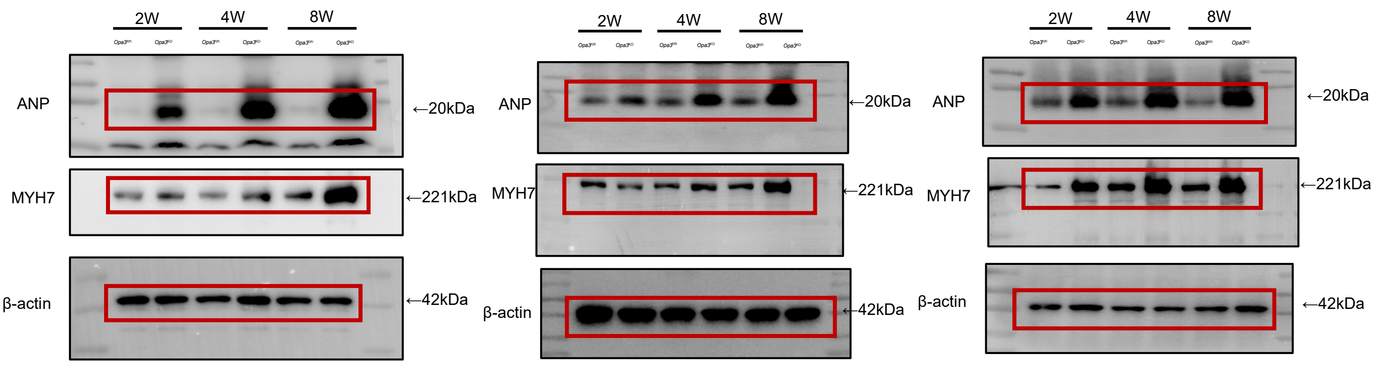

Full unedited gel for Supplementary Fig. 4f

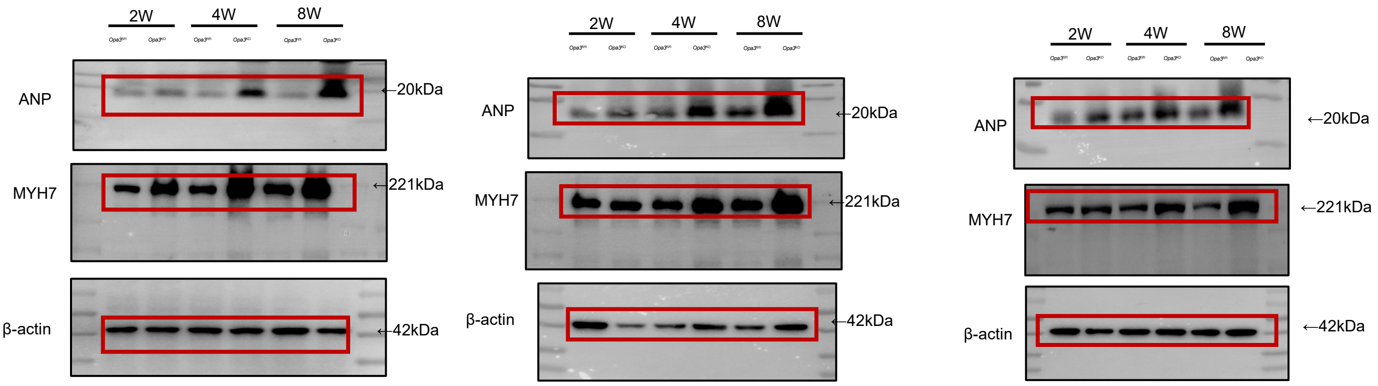

Full unedited gel for Supplementary Fig. 5c

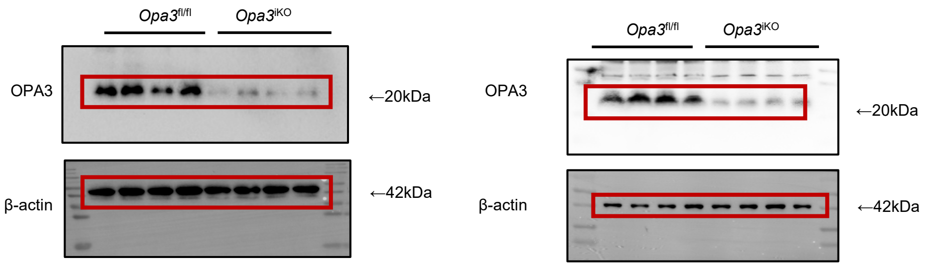

Full unedited gel for Supplementary Fig. 6l

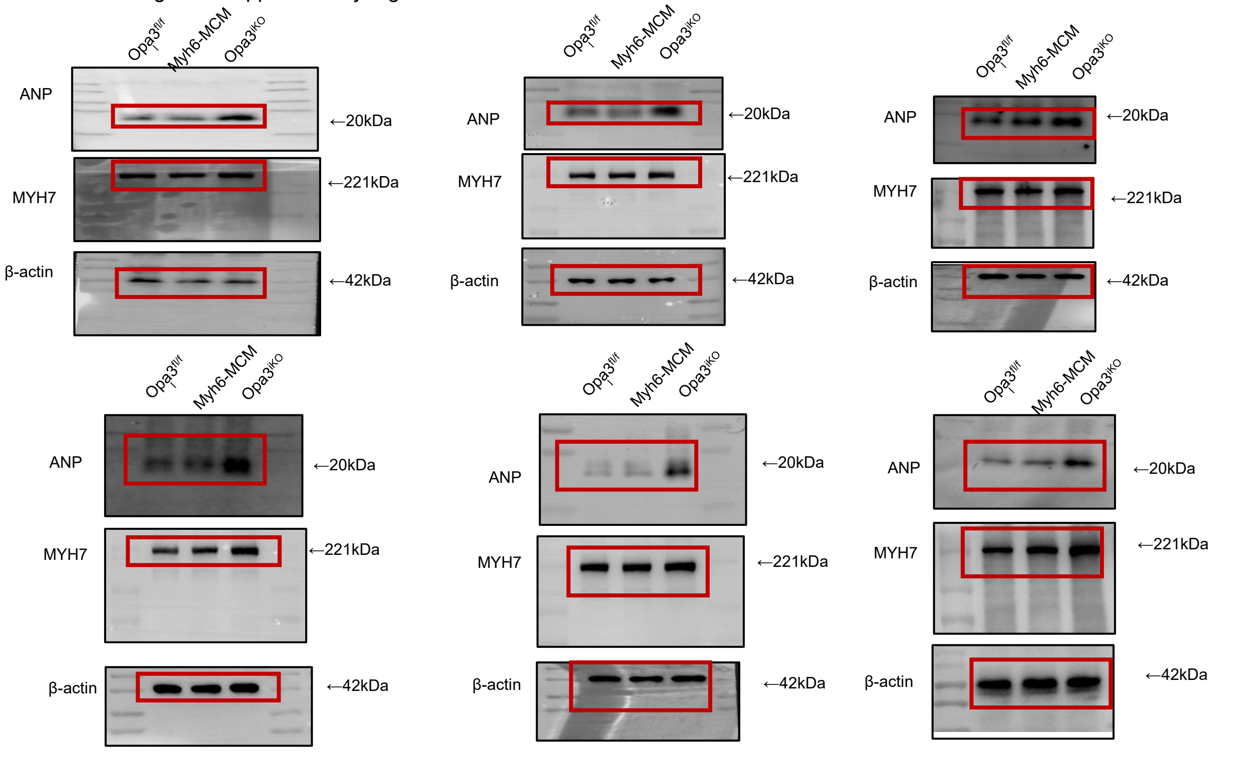

Full unedited gel for Supplementary Fig. 7j

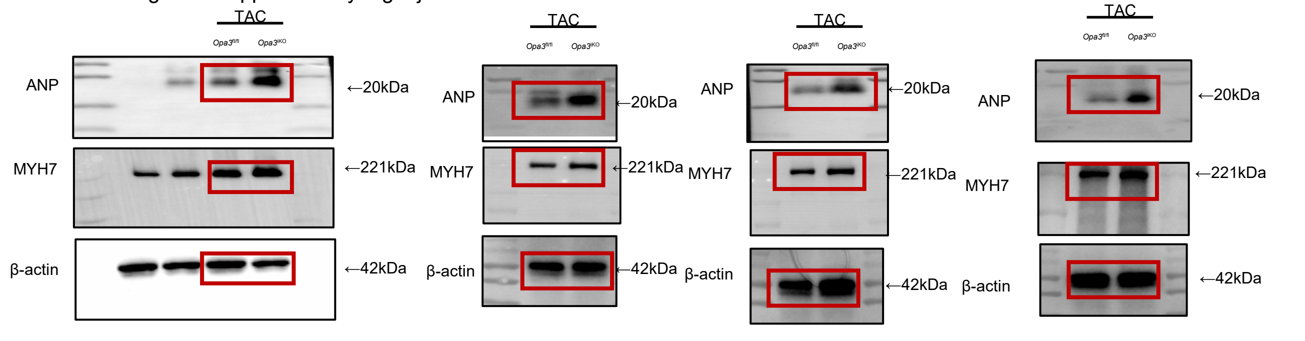

Full unedited gel for Supplementary Fig. 7j

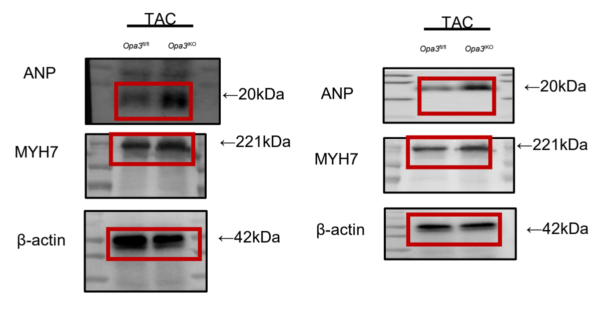

Full unedited gel for Supplementary Fig. 8b

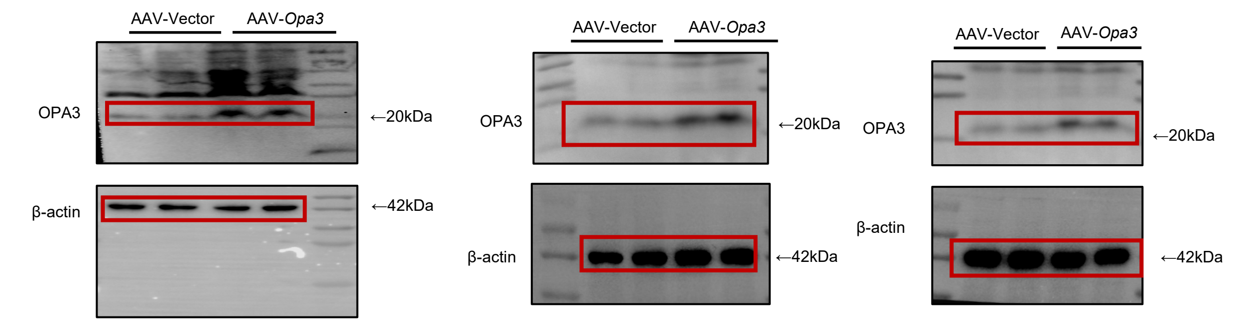

Full unedited gel for Supplementary Fig. 8l

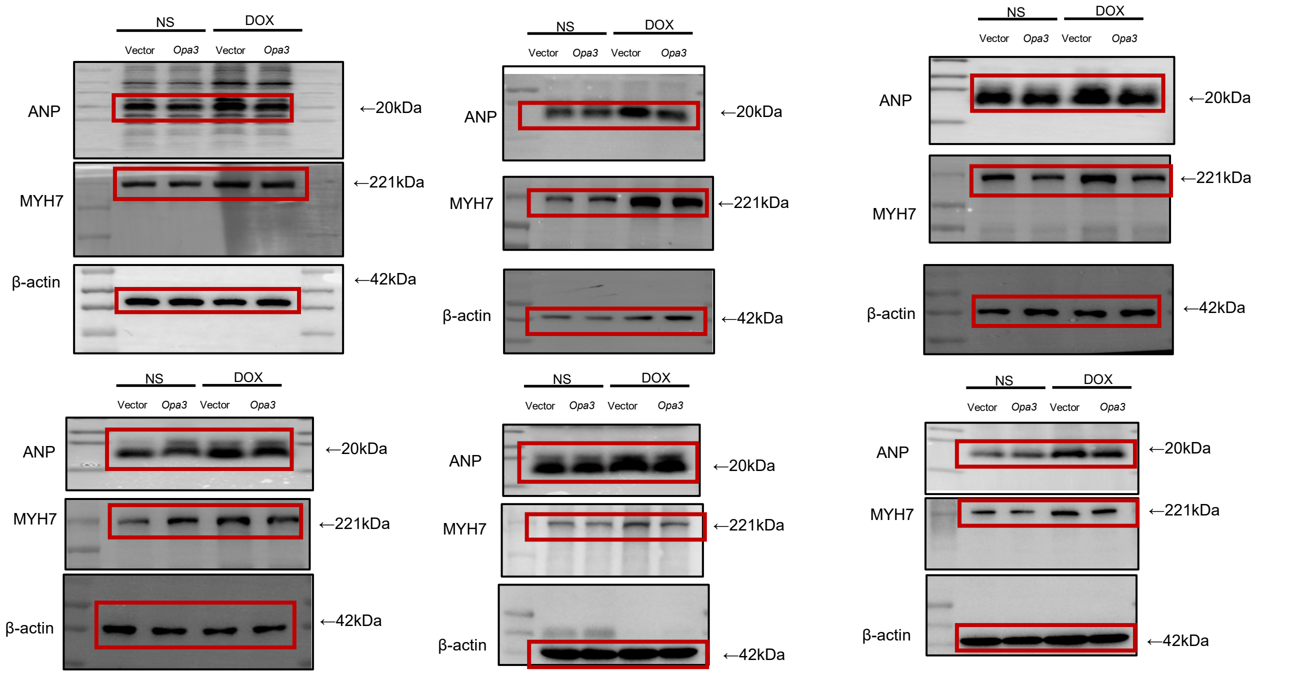

Full unedited gel for Supplementary Fig. 11a

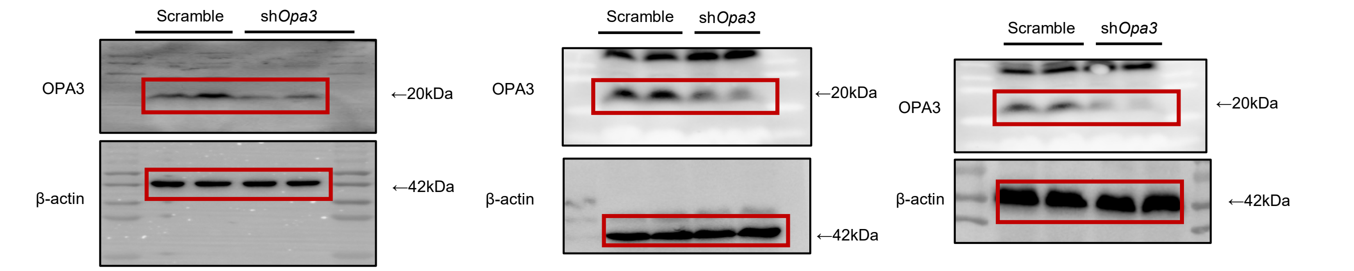

Full unedited gel for Supplementary Fig. 15b

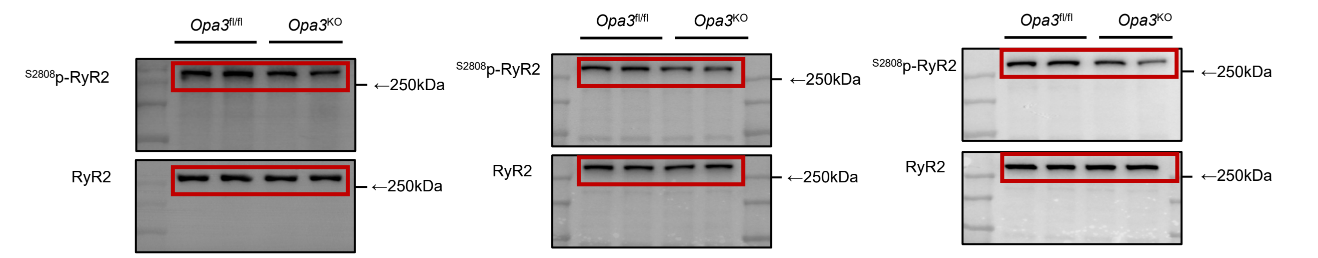

Full unedited gel for Supplementary Fig. 15d

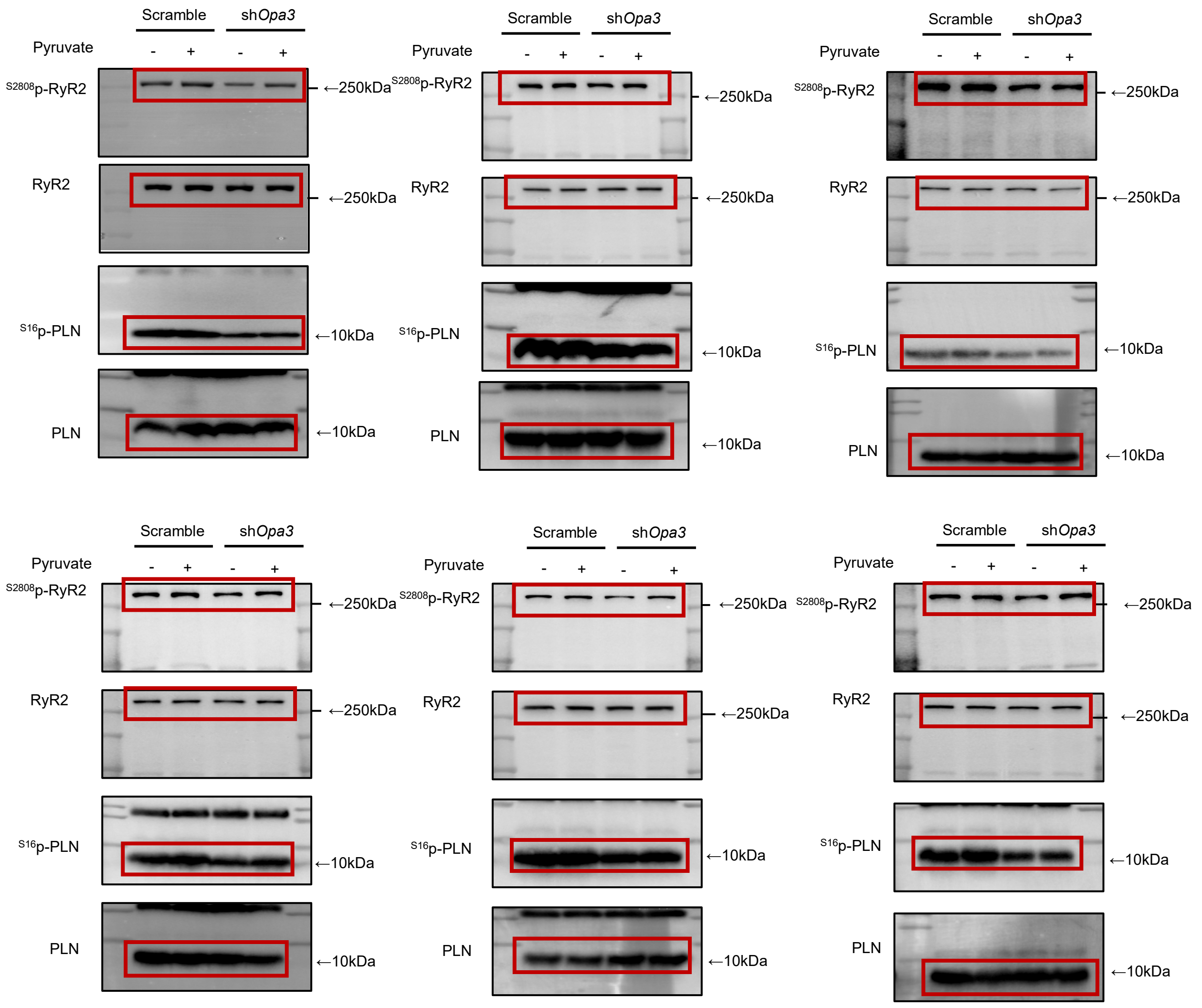

Full unedited gel for Supplementary Fig. 15e

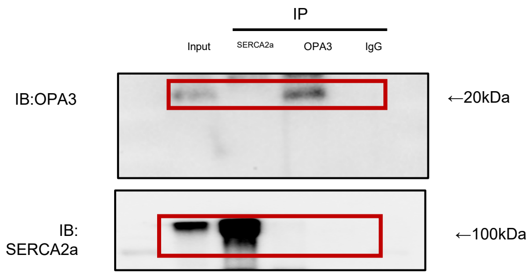

Full unedited gel for Supplementary Fig. 15f

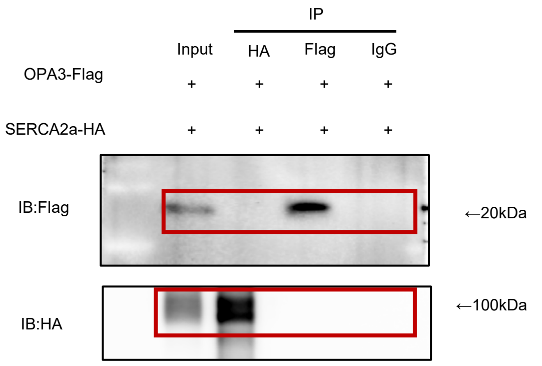

Full unedited gel for Supplementary Fig. 16b

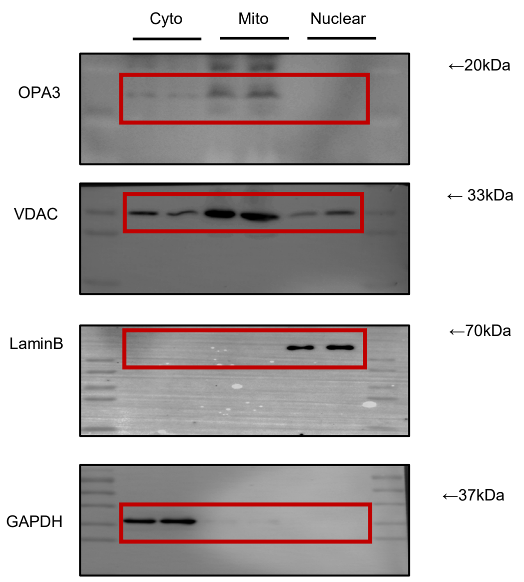

Full unedited gel for Supplementary Fig. 16e

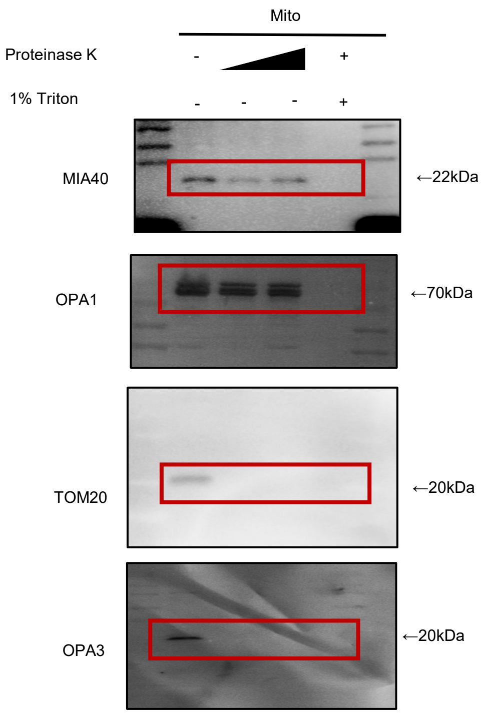

Full unedited gel for Supplementary Fig. 17

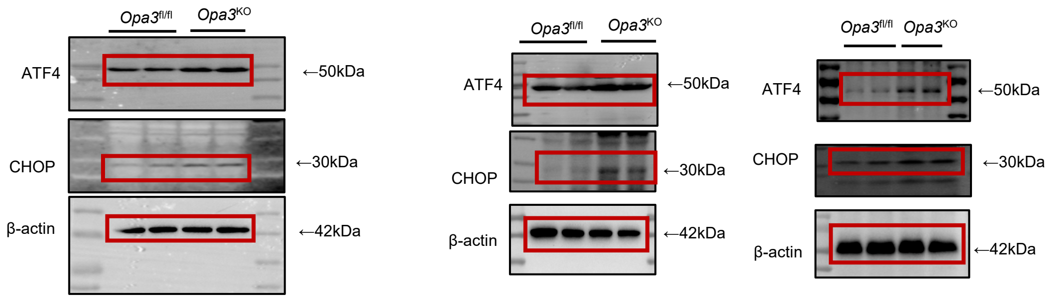

Full unedited gel for Supplementary Fig. 20

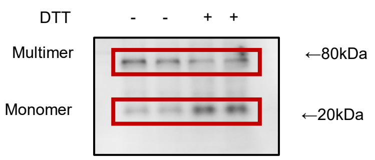

Full unedited gel for Supplementary Fig. 21

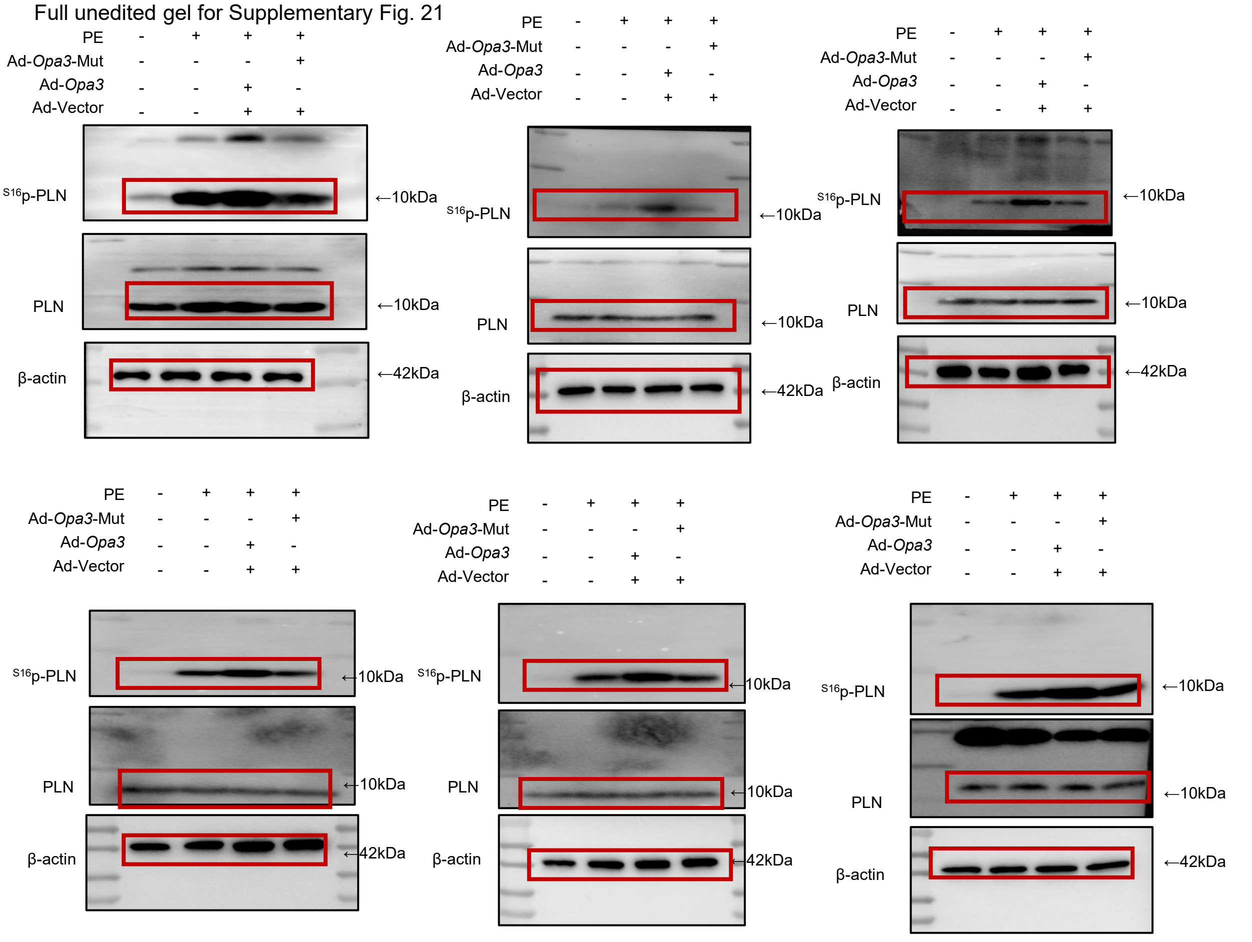

Full unedited gel for Supplementary Fig. 23

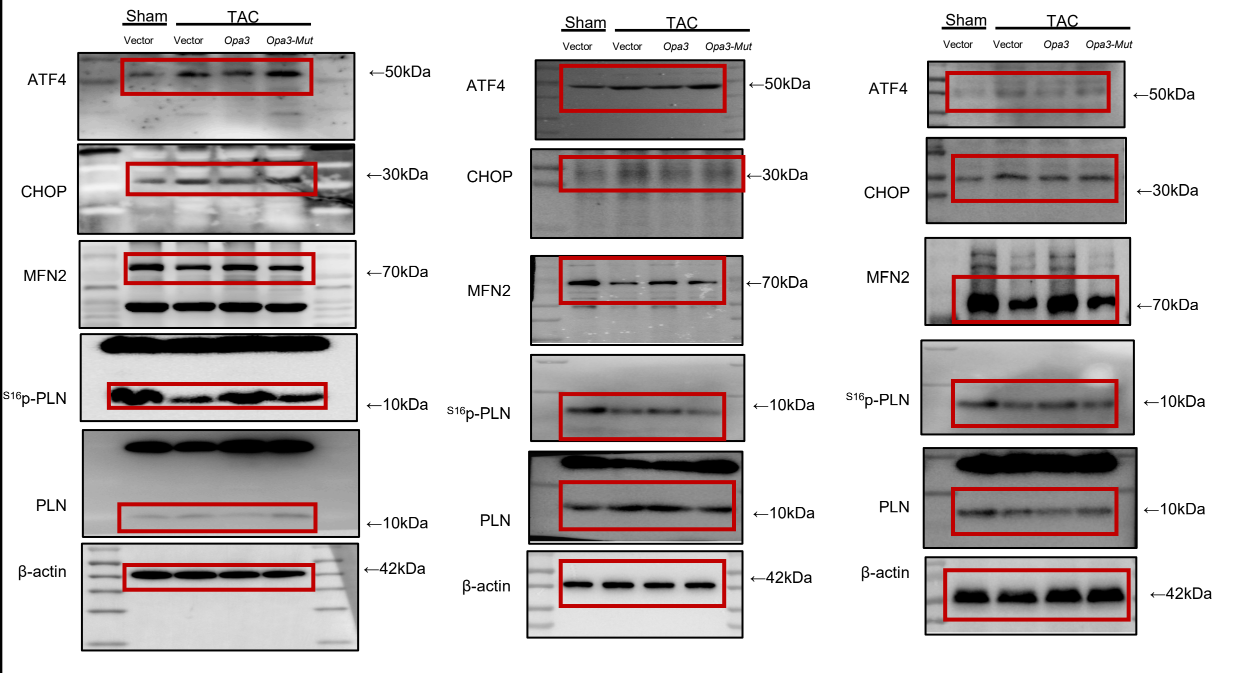

Full unedited gel for Supplementary Fig. 23

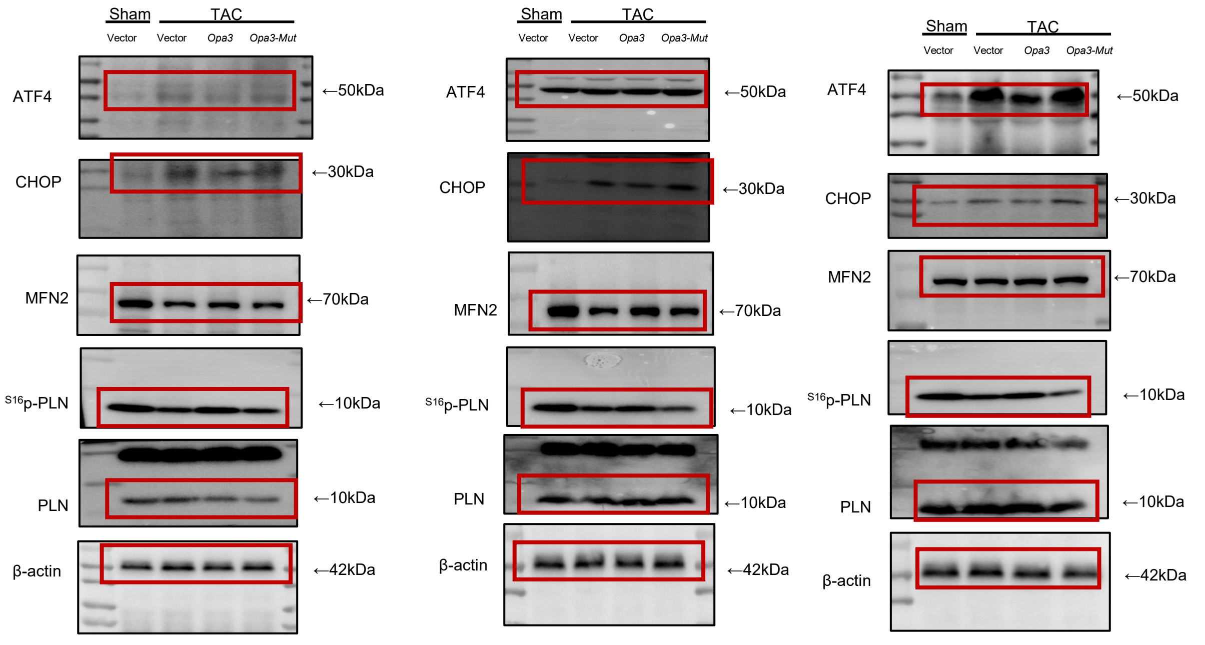

Supplement: Supplementary file 4 [file 41467_2026_73991_MOESM4_ESM.zip › Source data/Unedited immunoblots.pdf]
